# Multitopic Corannulene–Porphyrin Hosts for Fullerenes: A Three-Layer Scaffold for Precisely Designed Supramolecular Ensembles

**DOI:** 10.1021/acs.orglett.4c04385

**Published:** 2024-12-20

**Authors:** Nerea Álvarez-Llorente, Anton J. Stasyuk, Alberto Diez-Varga, Sergio Ferrero, Miquel Solà, Héctor Barbero, Celedonio M. Álvarez

**Affiliations:** † Institut de Química Computacional and Departament de Química, Universitat de Girona, C/Maria Aurèlia Capmany 69, 17003 Girona, Spain; ‡ GIR MIOMeT, IU CINQUIMA/Química Inorgánica, Facultad de Ciencias, Universidad de Valladolid, Valladolid E47011, Spain

## Abstract

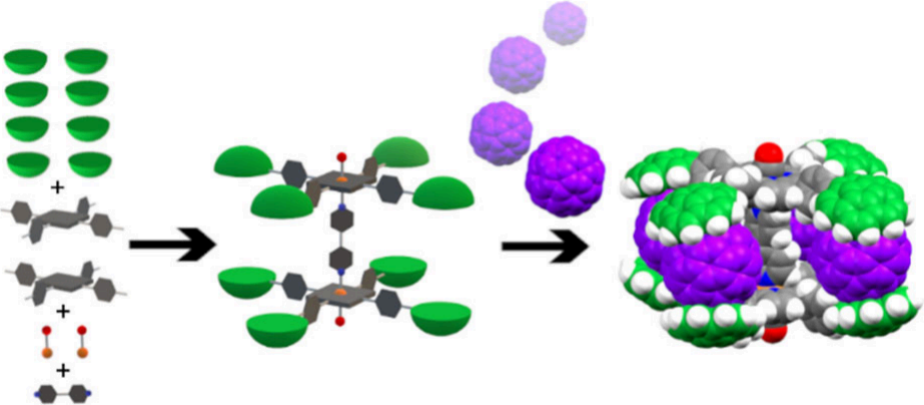

A method to synthesize cofacial dimeric porphyrins bearing eight corannulene units has been developed. It relies on the stability of octahedral CO-capped Ru(II) complexes linked by N-donor ligands. This specific arrangement provides an optimal scaffold to accommodate fullerenes by imposing corannulene groups at a precise distance and relative orientation. Their capabilities for C_60_ recognition have been thoroughly assessed, revealing that each system can encapsulate up to four guests, giving rise to a compact supramolecular van der Waals complex echoing a discrete donor–acceptor–donor trilayer offering significant potential properties for further exploitation.

Organic-based materials comprising small molecule entities with potential applications in many fields of the chemical sciences require a certain degree of order in the relative location and orientation of their constituents. Properties such as the size of the excitons, optical gap, mobility, and redox potentials critically depend on these features.^1^ The distance in donor–acceptor (DA) junctions directly impacts electron transfer processes and must be carefully engineered to provide the most efficient electron transfer kinetics.^1e^ Specific host–guest recognition in supramolecular adducts is an excellent strategy to fulfill these requirements because interacting electron-active units self-assemble in ordered structures.^1d^

[5]Circulene (corannulene) is a nonplanar aromatic hydrocarbon exhibiting versatile applications as organic devices.^2^ One of the most interesting properties is the supramolecular recognition of fullerenes^3^ due to the concave/convex complementarity between their topologies. However, a single unit of corannulene is insufficient to establish strong interactions. This limitation has led to the development of various strategies to enhance these interactions such as π-extension^4^ or the design of molecular tweezers, where two corannulene moieties cooperate to bind fullerenes.^5^ However, increasing the number of corannulene units in flexible systems does not unequivocally enhance affinity.^6^ This suggests that multitopic receptors may not fully utilize all available binding sites, except in polymeric frameworks.^7^ Additionally, porphyrins have shown remarkable proficiency in fullerene recognition,^8^ paving the way for the exploration of emergent properties in resulting DA adducts.^9^ Our investigations into porphyrin–corannulene ensembles demonstrate their synergistic recognition capabilities.^10^ Nonetheless, a multitopic receptor has never been achieved (Figure 1a–c). We therefore aimed to develop a platform in which more than two corannulene moieties are preorganized, using porphyrin primarily as an anchoring scaffold rather than an active recognition motif. By grafting a Ru(II)–CO fragment onto a free-base porphyrin, we could explore the sixth coordination position using a quasilinear N-donor bidentate ligand with the appropriate stoichiometry. This approach might furnish a dimer consisting of two octahedral complexes with inherent thermodynamic and kinetic inertness.^11^ Such a porphyrin dimer would render an arrangement in which eight corannulenes are placed in a pairwise manner at the appropriate distance, solely dictated by the ligand. With regard to N-donor ligands, we opted to investigate 4,4′-bipyridyl (bpy) and 1,4-di(pyridin-4-yl)benzene (dpyb), which typically exhibit N–N distances of 7.06 and 11.41 Å, respectively. Given their proximity to the diameter of C_60_ (7.07 Å), the resulting dimeric hosts are expected to strongly interact with it. This design holds the potential to accommodate up to four sites for fullerene recognition (Figure 1d).

**Figure 1:**
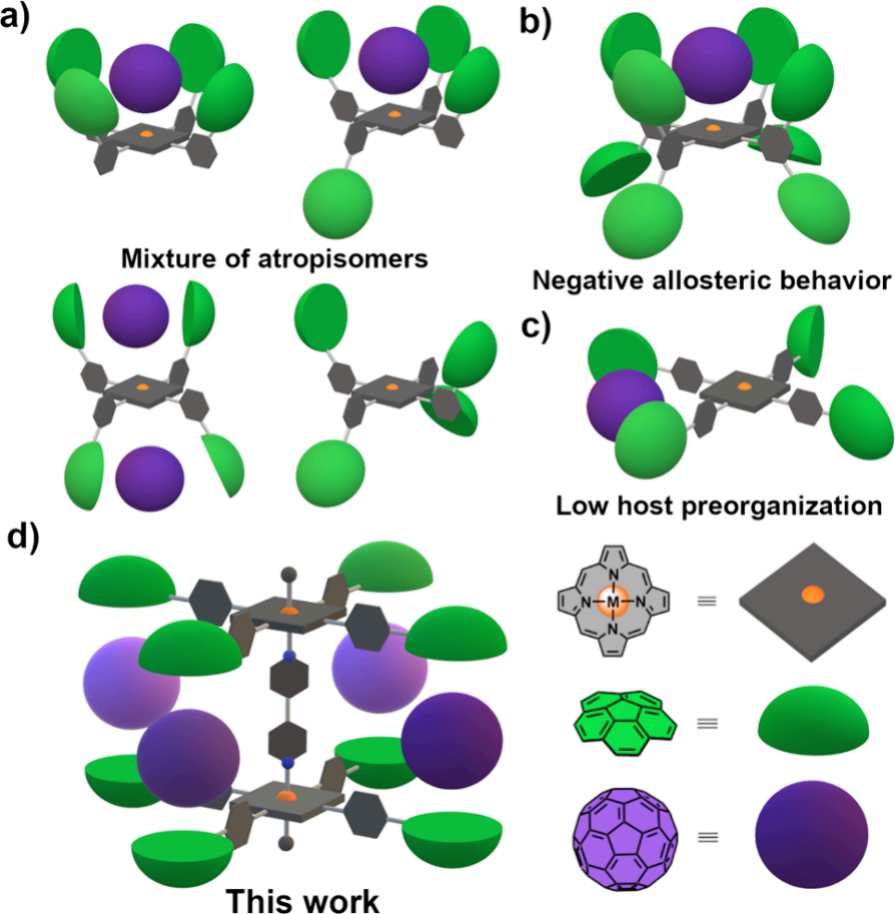
Cartoon depiction of previously reported multicorannulene porphyrin-based hosts. (a) Mixture of nonfunctional atropisomers.^10a^ (b) Negative allosteric induction due to excellent synergy in the first recognition step.^10b^ (c) Neither porphyrin contribution nor host preorganization.^10c^ (d) Four-fold hosts reported herein.

The synthetic strategy (Scheme 1) starts with free-base porphyrin **2HP-Br** that readily reacts with trimer [Ru_3_(CO)_12_] in excess furnishing complex **RuP-Br** in good yield (79%). The next step consisted of a dimerization via addition of 0.5 equiv of the corresponding bidentate N-donor ligand. This process furnished complexes **(RuP-Br)**_**2**_**·bpy** and **(RuP-Br)**_**2**_**·dpyb** in nearly quantitative yield. Finally, a multi-Suzuki C–C cross-coupling between the parent brominated complex and an excess of the boronate ester of corannulene was carried out. An octa-Suzuki reaction has been previously achieved^10b^ and can be readily performed in toluene under microwave irradiation with ^*t*^BuONa as the base and [PdCl_2_(dppf)] as the catalyst. The procedure gave rise to final complexes **(RuP-cor)**_**2**_**·bpy** and **(RuP-cor)**_**2**_**·dpyb** in good yields (64% and 60%, respectively).^13^ Compound **RuP-cor·py** (see Scheme 1) was also prepared and will be used as a monomeric reference system.^14^

**Scheme 1:**
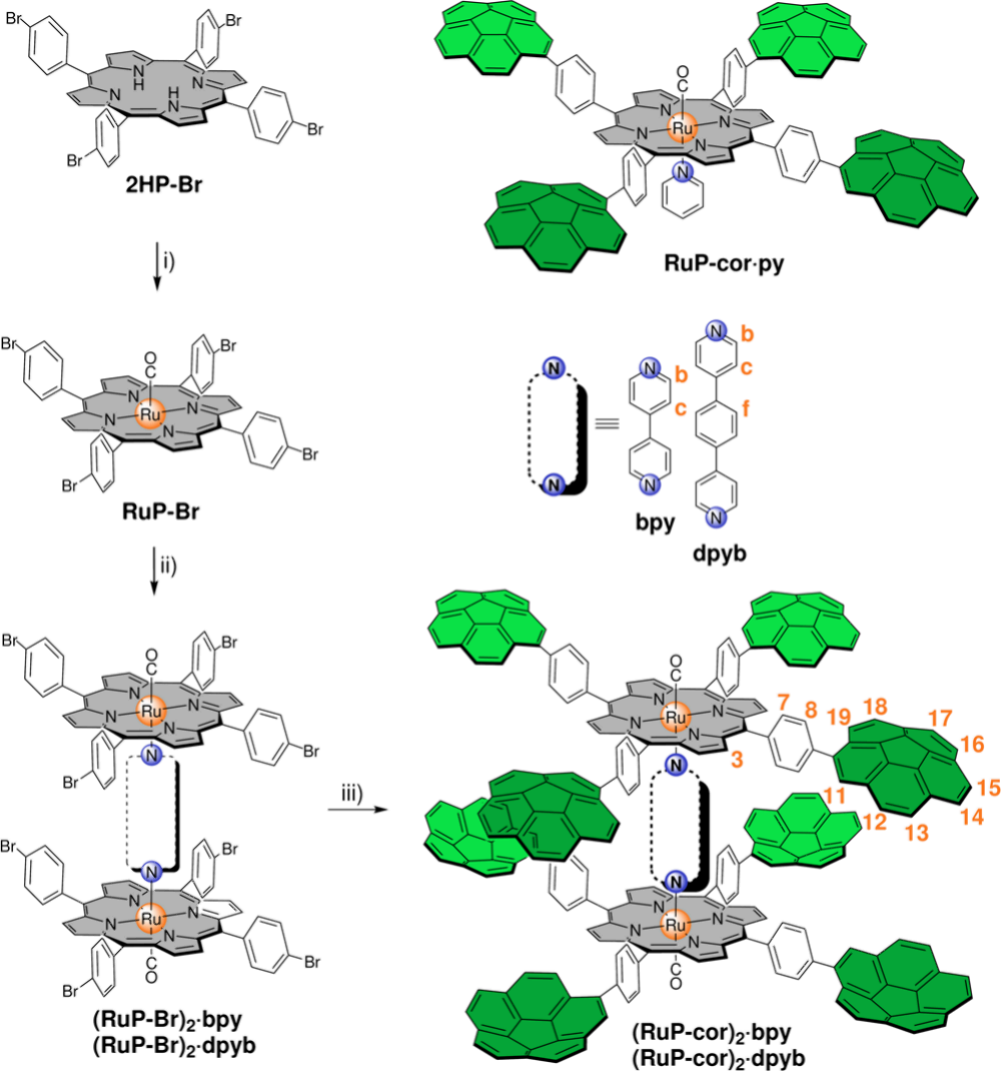
Synthetic Strategy to Prepare Porphyrin Complexes **(RuP-cor)**_**2**_**·bpy** and **(RuP-cor)**_**2**_**·dpyb** with Atom Numbering^12^, Reagents and conditions: (i) Ru_3_(CO)_12_, toluene, reflux; (ii) 0.5 equiv of bidentate ligand, DCM, rt; (iii) Bpin-cor, [PdCl_2_(dppf)], ^*t*^BuONa, toluene, microwave irradiation, 135°C. **RuP-cor·py** is also shown.

In general, ^1^H nuclear magnetic resonance (NMR) spectra are relatively simple due to the symmetric nature of the systems as well as the free rotation of the porphyrins along the OC–Ru–N(bpy or dpyb) axis (Figure 2a). Signature β-pyrrole chemical shifts (H^3^) are the most deshielded nuclei at 8.75 and 8.83 ppm, whereas corannulene protons resonate between 8.5 and 7.7 ppm. Aromatic protons pertaining to bridging ligands (H^b^–H^f^) experience an outstanding upfield shift (6.32 to 1.36 ppm) that is less pronounced, as the nuclei are located farther from Ru(II). This is a consequence of the strong magnetic field imposed by the porphyrin π-ring current, clearly indicating axial coordination (blue signals in Figure 2a). Absorption UV–vis spectra show the expected set of signals corresponding to π–π* transitions [strong Soret band at 415 nm and two weak Q bands at 534 and 568 nm (Figure 2b)], typical of coordinated meso-substituted porphyrins according to the four-orbital Gouterman model.^15^ The reduction in the number of Q bands (from four to two) arises from the degeneration of the HOMO and HOMO–1 due to metalation.^16^ Attaching corannulene groups to the scaffold in both complexes minimally alters the absorption features, with Soret and Q bands showing slight bathochromic shifts of 6 and 3 nm, respectively, on average. This suggests weak electron coupling between the porphyrin core and the nonplanar aromatic groups, likely due to a dihedral angle of ∼34°.^17^ In terms of emission, two distinct bands can be discerned at ∼660 and ∼725 nm (Figure 2c). The first band possesses fluorescent character, whereas the second band demonstrates phosphorescence, evidenced by a marked enhancement in intensity under deaerated conditions (Figure 2c, blue), proving its ^3^MLCT (Metal to Ligand Charge Transfer) nature due to the presence of a closed-shell heavy metal favoring spin–orbit coupling.^15a,18^

**Figure 2:**
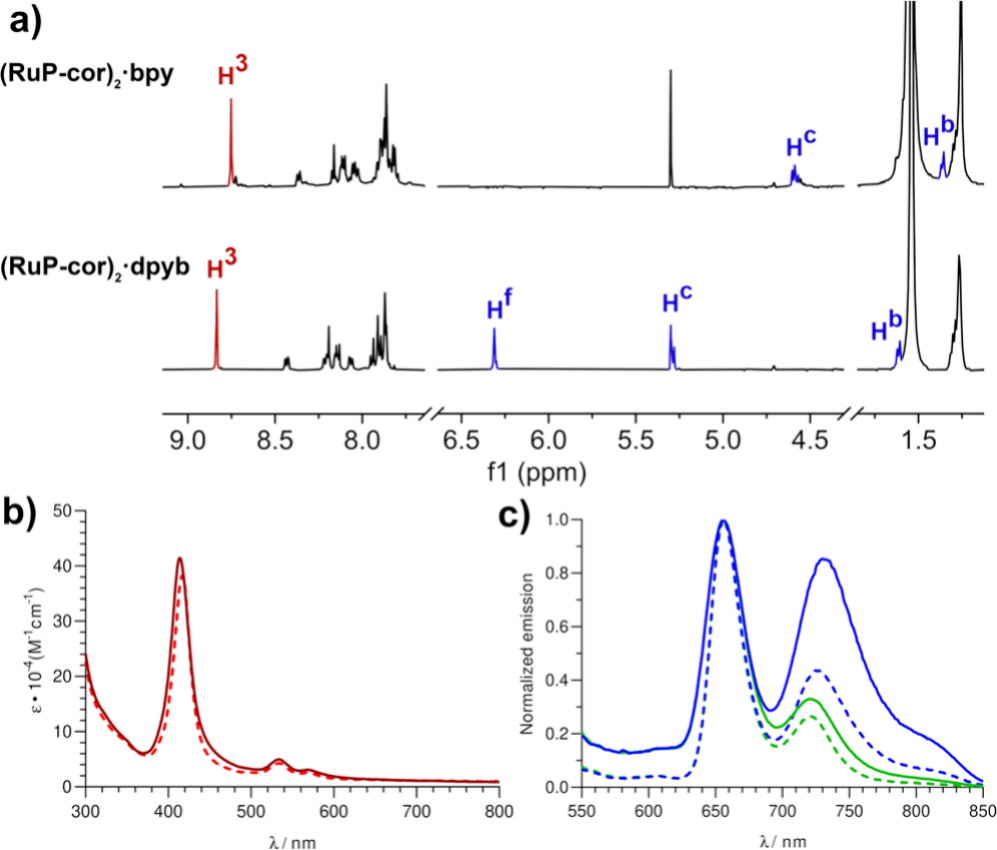
(a) Partial ^1^H NMR spectrum (500 MHz, CDCl_3_) with key signals colored red (β-pyrrole) and blue (bridging ligand). (b) UV–vis spectrum (DCM) of compounds **(RuP-cor)**_**2**_**·bpy** (solid line) and **(RuP-cor)**_**2**_**·dpyb** (dashed line). (c) Emission spectra (DCM; λ_ex_ = 516 nm) of the same dimers (green lines) and those under deareated conditions (blue lines).

To evaluate fullerene recognition capabilities of synthesized dimers, a series of titrations were conducted at room temperature in toluene-*d*_8_ and monitored by NMR. Monomer **RuP-cor·py** was subjected to the same protocol. Despite significant chemical shift changes observed during titration, most signals broadened after the initial additions, likely due to the deceleration of porphyrin rotation, precluding precise analysis, even at high temperatures (Figures S121 and S122). Interestingly, control host **RuP-cor·py** did not suffer from these inconveniences (Figure S119). With regard to absorption experiments, a very small hypsochromic shift (4 nm) of the Soret band, concomitant with a mild enhancement of the intensity of Q bands, was observed, indicating that the ground-state electronic properties of the porphyrin remain in the supramolecular adduct. In other words, the porphyrin core is not involved in the recognition event, and therefore, it takes place within the cavities imposed by pairs of corannulenes. Moreover, no significant charge transfer (CT) band was detected (Figure S118). This is likely due to (1) the dominance of dispersion forces in the supramolecular interaction and (2) the low solvent polarity, which does not support CT complex stabilization.^19^ Conversely, emission experiments proved to be highly effective for monitoring supramolecular adduct formation. The fluorescence band of all corannulene-based hosts was efficiently quenched upon fullerene addition at a constant host concentration (Figure 3a).^14^ This suggests the involvement of corannulene-localized molecular orbitals in the ^1^MLCT state.^20^ This strategy has previously been successful in other molecular tweezers based on a corannulene motif.^21^ Given the complexity of fullerene binding, we applied nonlinear regression analysis to fit the fluorescence intensity decay across a series of models ranging from 1:1 to 1:4 stoichiometries following Thordarson and Miyake’s analysis.^14,22^ It was conducted under the assumption of static quenching and a non-emissive guest (Figure 3a, inset).^23^ The host concentration was kept constant and low (10^–6^ M) so that the absorption of the species at the excitation wavelength (516 nm, Q-band) lies below 0.05.^23b^ Control host **RuP-cor·py** was analyzed using the same protocol, revealing a dominant 1:1 stoichiometry with an association constant of 373 M^–1^. This value aligns closely with the result from NMR (362 M^–1^)^14^ and a previously reported Zn-based porphyrin host (Figure 1c, 273 M^–1^).^10c^ This consistency validates the method used, confirms that emission decay is due to adduct formation (static quenching), and verifies that host **RuP-cor·py** binds in a tweezer-like arrangement.

**Figure 3:**
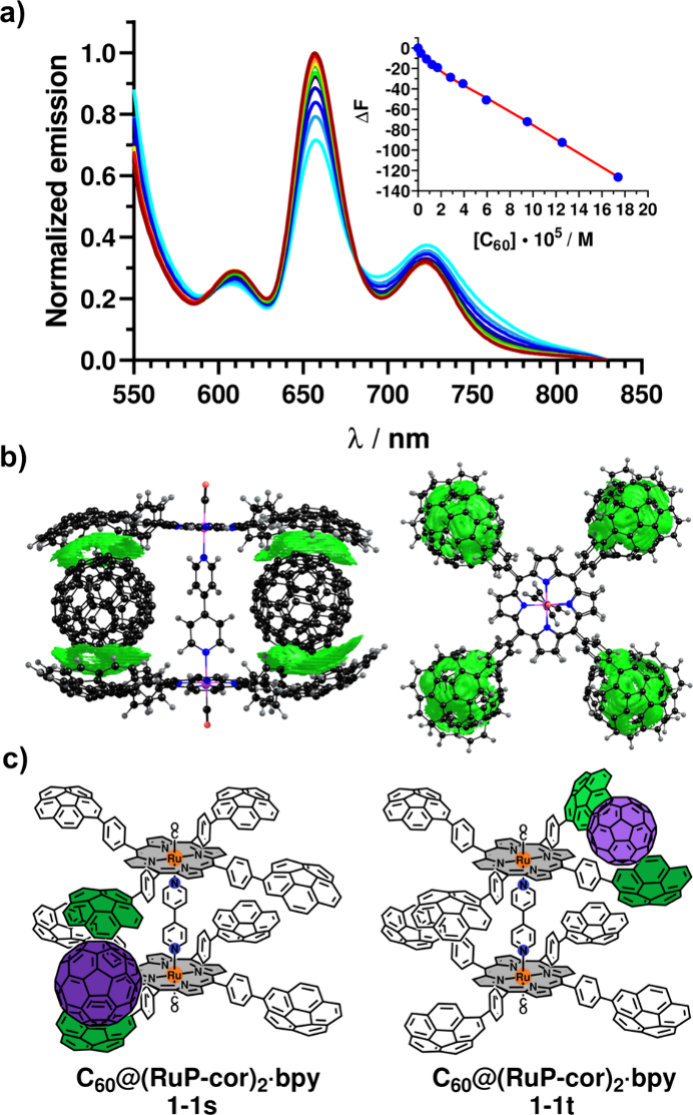
(a) Normalized emission spectra (toluene; λ_ex_ = 517 nm) of complex **(RuP-cor)**_**2**_**·bpy** upon addition of C_60_ at room temperature. The inset shows the fluorescence quenching binding isotherm at 657 nm. Blue dots are experimental data, and the red line is a nonlinear regression fit using a 1:4 noncooperative model. (b) NCI isosurfaces showing vdW interactions in the **(C**_**60**_**)**_**4**_**@(RuP-cor)**_**2**_**·bpy** assembly. (c) Depiction of adduct **C**_**60**_**@(RuP-cor)**_**2**_**·bpy** with two arrangements: sandwich-like (s) and tweezer-like (t). Corannulene units involved in recognition are colored green.

With regard to dimers **(RuP-cor)**_**2**_**·bpy** and **(RuP-cor)**_**2**_**·dpyb**, the optimal fit was a noncooperative 1:4 binding model, showing low residuals and a high covfit factor (≤10.5).^22,23b^ Macroscopic association constants are listed in Table 1. Despite allosteric effects observed in double-decker systems,^24^ the noncooperative model dominates, as initial binding does not change the host structure to facilitate subsequent binding. Thus, the first values (*K*_1_, in M^–1^) are comparable to benchmarks such as rigid Sygula’s Buckycatchers I and II (2.8 × 10^3^ and 8.5 × 10^4^, respectively)^5b,25^ and Chen’s helicene (2.8 × 10^3^),^21^ despite the energy penalty arising from free rotation of porphyrin cores.

**Table 1:** Stepwise Association Constants (M^–1^) for Hosts with C_60_

host	*K*_1_	*K*_2_	*K*_3_	*K*_4_
**RuP-cor·py**	(3.73 ± 0.06) × 10^2^	–	–	–
**(RuP-cor)_2_·bpy**^a^	(2.12 ± 0.12) × 10^4^	(7.96 ± 0.45) × 10^3^	(3.54 ± 0.20) × 10^3^	(1.33 ± 0.08) × 10^3^
**(RuP-cor)_2_·dpyb**^a^	(3.08 ± 0.29) × 10^4^	(1.16 ± 0.11) × 10^4^	(5.14 ± 0.48) × 10^3^	(1.93 ± 0.18) × 10^3^

a Uncertainties estimated with Monte Carlo simulations.^23c^

However, they perform worse than Buckycatcher III (5 × 10^4^ in chlorobenzene, yet in a 2:1 adduct).^5a^ Moreover, the association constants of both dimers are higher than those of previous atropisomeric porphyrins (Figure 1a, 5.4 × 10^3^ on average)^10a^ and are comparable to those of the octapodal porphyrin with negative allosteric binding (Figure 1b, 2.7 × 10^4^).^10b^ Importantly, these dimers do not benefit from the porphyrin core assistance in binding. Overall, both hosts outperform the control porphyrin by 2 orders of magnitude (Table 1), with host **(RuP-cor)**_**2**_**·dpyb** showing a slight advantage [log β_**(RuP-cor)_2_·bpy**_ = 14.9 vs log β_**(RuP-cor)_2_·dpyb**_ = 15.5]. Therefore, the ligand length within this range has minimal impact.

To elucidate the most likely structures of the supramolecular complexes in solution, the geometries of inclusion complexes **(RuP-cor)**_**2**_**·bpy** and **(RuP-cor)**_**2**_**·dpyb** were optimized at the GFN2-xTB^26a^ level. Noncovalent interaction (NCI) analysis^26b^ indicated extended regions of weak π···π interactions between corannulenes and C_60_ (Figure 3b and Figure S124). Morokuma-like energy decomposition analysis (EDA)^26c^ showed that dispersion interactions (Δ*E*_disp_) constitute ∼58% of the total interaction energy, followed by electrostatic attraction (Δ*E*_elstat_ ≤ 28%) and orbital interactions [Δ*E*_oi_ < 15% (Table S8)]. The interaction energy (Δ*E*_int_), calculated at the BLYP(D3BJ)/TZP//GFN2-xTB level,^14^ for assembly **(C**_**60**_**)**_**4**_**@(RuP-cor)**_**2**_**·bpy** is −176.9 kcal/mol (Table S8), nearly 4 times higher than that for adduct **C**_**60**_**@(RuP-cor)**_**2**_**·bpy** (see below). Fullerene center distances range from 14.7 to 15.6 Å (Figure S125), exceeding the sum of a C_60_ diameter and twice the van der Waals (vdW) radius of carbon. Thus, the addition of each new fullerene to the complex is energetically equivalent. These findings align with experimental association constants, confirming noncooperative binding and a lack of interactions between fullerenes.

The binding mechanism is convoluted and is not directly accessible experimentally. However, the first recognition event can be ventured knowing that fullerene binding by control host **RuP-cor·py** involves a pincer-like interaction between two adjacent corannulene groups as discussed above. For porphyrin dimers, two possible binding modes might exist: a tweezer-like (**1-1t**) or a sandwich-like (**1-1s**) arrangement (Figure 3c). Complexity significantly increases with 1:2 and 1:3 stoichiometries (Scheme S2). Optimized structures of sandwich-like (**1-1s**) and tweezer-like (**1-1t**) assemblies were obtained using the same computational protocol (Figure S124), furnishing Δ*E*_int_ values of −44.4 and −43.1 kcal/mol, respectively. The deformation energies (Δ*E*_def_), i.e., the energy penalty for host reorganization to bind the guest, were 1.9 and 6.7 kcal/mol, respectively. The higher Δ*E*_def_ for **1-1t** suggests that the formation of **1-1s** is energetically more favorable (Table S8). This is supported by experimental data as *K*_1_ for porphyrin dimers is 2 orders of magnitude higher than *K* for the control host (Table 1), suggesting that the binding mechanism likely involves sequential sandwich-like assemblies (Scheme S3).

In summary, a suitable synthetic protocol for obtaining porphyrin dimers based on Ru–N coordination bearing eight corannulene units has been developed. They show excellent capabilities for C_60_ recognition, accommodating up to four guests within their structure in solution. The overall topology resembles a triple layer of DA adducts, paving the way for exploring higher fullerenes, potential photoinduced electron transfer processes, and possible hierarchical self-assembly into highly ordered materials.

## Data Availability

The data underlying this study are available in the published article and its Supporting Information.

## Supporting Information Available

Detailed experimental procedures; synthetic protocols; NMR, HRMS, FT-IR, and UV–vis absorption and emission spectra; X-ray crystallographic data; details of supramolecular titrations (fluorescence, UV–vis, and ^1^H NMR); variable-temperature (VT) ^1^H NMR experiments; and details of computational calculations (PDF)
