# Supplementary material for: Multitopic Corannulene–Porphyrin Hosts for Fullerenes: A Three-Layer Scaffold for Precisely Designed Supramolecular Ensembles
Source: Org Lett. 2024 Dec 20;27(1):357–62. doi: 10.1021/acs.orglett.4c04385 (PMC11731351; doi:10.1021/acs.orglett.4c04385)
Supplement: Supplementary file 1 — ol4c04385_si_001.pdf [file ol4c04385_si_001.pdf]

Multitopic corannulene-porphyrin hosts for fullerenes: a three-layer scaffold for precisely designed supramolecular ensembles

Nerea Álvarez-Llorente,[1,‡] Anton J. Stasyuk,[2] Alberto Diez-Varga,[1,‡] Sergio Ferrero,[1] Miquel Solà,[2,*] Héctor Barbero,[1,*] Celedonio M. Álvarez[1,*]

[1] GIR MIOMeT, IU CINQUIMA / Química Inorgánica, Facultad de Ciencias, Universidad de Valladolid, Valladolid, E47011, Spain

2 Institut de Química Computacional and Departament de Química, Universitat de Girona, C/ Maria Aurèlia Capmany 69, Girona, E17003, Spain

‡These authors contributed equally.

Table of contents

Experimental procedures ... 2

General methods ... 2

Synthetic procedures ... 3

Discussion about the synthetic strategy ... 8

NMR spectra ... 9

High Resolution Mass Spectrometry ... 68

FT-IR spectra ... 77

UV-Vis absorption and emission spectra ... 79

X-ray Crystallographic Tables ... 88

Supramolecular titrations ... 90

Fluorescence titrations ... 90

UV-Vis titrations ... 97

^{1}H NMR titrations ... 97

Variable temperature (VT) ^{1}H NMR experiments ... 99

Computational Calculations details ... 102

Cartesian Coordinates ... 105

References ... 124

Experimental procedures

General methods

All reagents were purchased from commercial sources and used without further purification. 1-Bromocorannulene was acquired from Synoi Chemicals (http://synoichemicals.uva.es/). Solvents were of analytical grade or spectrophotometric grade. They were either used as purchased or dried according to procedures described elsewhere.[1] Microwave reactions were carried out with an Anton Paar Monowave 300 Reactor using sealed G30, G10 and G4 reaction vessels (for volumes up to 30 mL, 10 mL and 4 mL, respectively) specially designed for the apparatus. The temperature was monitored by IR. When necessary, reactions were performed under an inert atmosphere with standard Schlenk techniques. Such techniques were also used as a preliminary step for degassing microwave flasks when an inert atmosphere was necessary in microwave reactions. Column chromatography separations were carried out using Silica gel 60 (particle size 0.040-0.063 mm; 230-400 mesh; Merck, Germany) or neutral alumina (particle size 0.040-0.300 mm, 60 A, Thermo Scientific) as the stationary phase, and TLC was performed on precoated silica gel plates (0.25 mm thick, 60 F254, Merck, Germany) and observed under UV light. Purifications by centrifugation were performed in a Nahita 2600. The NMR spectra were recorded on a 400 MHz Agilent NMR, a 500 MHz Agilent DD2 instrument equipped with a OneNMR probe, a 500 MHz Agilent DD2 instrument equipped with a cold probe, or a 500 MHz Bruker AVANCE NEO instrument equipped with an i-Probe. ^{1}H and ^{13}C chemical shifts (δ) are reported in parts per million (ppm) and are referenced to tetramethylsilane (TMS) using the residual solvent peak as an internal reference. Coupling constants (*J*) are reported in Hz. Standard abbreviations are used to indicate multiplicity: s, singlet; d, doublet; t, triplet; dd, doublet of doublets; and m, multiplet. ^{1}H and ^{13}C peak assignments were performed using 2D NMR methods (^{1}H-^{1}H gCOSY, ^{1}H-^{1}H-gDQFCOSY, ^{1}H-^{1}H NOESY, ^{1}H-^{1}H ROESYAD, ^{1}H-^{13}C gHSQCAD, ^{1}H-^{13}C gHMBCAD, ^{1}H-^{13}C gc2hsqc, ^{1}H-^{13}C gc2hmbc, band selective ^{1}H-^{13}C gHSQCAD, band selective ^{1}H-^{13}C HMBC). Due to the low solubility, some carbon signals were detected indirectly via ^{1}H-^{13}C HSQC/HMBC experiments (C-in). High resolution mass spectra were recorded at mass spectrometry service of the Laboratory of Instrumental Techniques of the University of Valladolid (L.T.I., www.laboratoriotecnicasinstrumentales.es). A MALDI-TOF system (Bruker Autoflex Speed; N_2 laser 337 nm, pulse energy 100 μJ, 1 ns, acceleration voltage 19 kV) was used in reflector or linear positive modes. *Trans*-2-[3-(4-tert-butylphenyl)-2-methyl-2-propenylidene]malonitrile (DCTB) and 1,8-dihydroxy-9(10H)-anthracenone (dithranol) were used as matrixes. IR spectra in solid were recorded with a Frontier Perkin Elmer Spectrum RX I FT-IR instrument. Steady-state UV-Vis absorption spectroscopy was carried out on a Perkin Elmer Lambda 265 spectrophotometer, whereas emission spectroscopy was performed on a Cary Eclipse Fluorescence Spectrophotometer (Agilent), equipped with a single cell peltier for temperature control, using quartz cuvettes with a path length of 1 cm in DCM or toluene as the solvent. Diffraction data were collected using an Oxford Diffraction Supernova diffractometer equipped with an Atlas CCD area detector and a four-circle kappa goniometer. For the data collection, Mo or Cu micro-focused sources with multilayer optics were used. When necessary, crystals were mounted directly from solution using perfluorohydrocarbon oil to prevent atmospheric oxidation, hydrolysis, and solvent loss. Data integration, scaling, and empirical absorption correction were performed using the CrysAlisPro software package. The structure was solved by direct methods and refined by full-matrix-least-squares against F2 with SHELX in

OLEX2. Non-hydrogen atoms were refined anisotropically, and hydrogen atoms were placed at idealized positions and refined using the riding model. Graphics were made using OLEX2 and MERCURY. 5,10,15,20-tetrakis(4-bromophenyl)porphyrin (*2HP-Br*), 1,4-bis(4-pyridyl)benzene (*dpyb*), 1-pinacol pyreneboronate (*Bpin-pyr*) and 1-pinacol corannuleneboronate (*Bpin-cor*) were synthesized following reported methods.[2–5]

Synthetic procedures

Synthesis overview

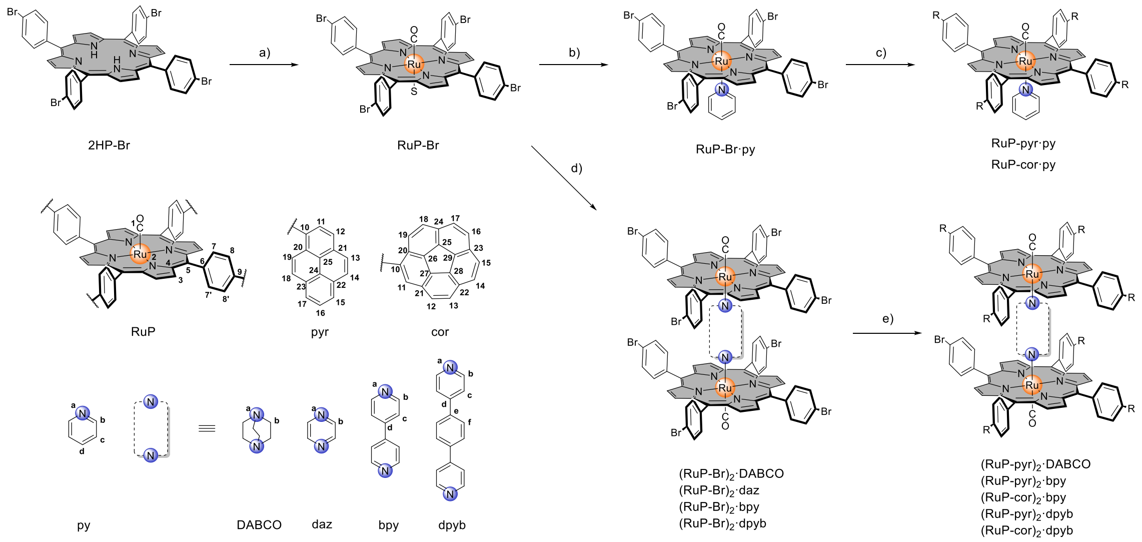

Scheme S 1. Synthesis overview to achieve the complexes prepared in this work. Reagents and conditions: a) $Ru_3(CO)_{12}$, toluene, reflux. b) 1 eq. of pyridine, DCM, rt. c) 4 eq. Bpin-PAH, $[PdCl_2(dppf)]$, ^{t}BuONa, toluene, MW, 135°C. d) 0.5 eq. of ligand, DCM, rt. e) 8 eq. Bpin-PAH, $[PdCl_2(dppf)]$, ^{t}BuONa, toluene, MW, 135°C.

RuP-Br. A variation of a method described elsewhere[6] was followed. *2HP-Br* (100 mg, 0.107 mmol) and $Ru_3(CO)_{12}$ (137 mg, 0.215 mmol) were dissolved in 5 mL of dry toluene. The mixture was heated under reflux until no absorption bands of *2HP-Br* were observed by UV/Vis. Then it was allowed to cool to room temperature and the solvent was removed under reduced pressure. The crude was purified by column chromatography on alumina (gradient hexane, 2:1 hexane/DCM, DCM, THF) to give the pure compound as a garnet solid (93 mg, 79% yield). ^{1}H NMR (400 MHz, $CDCl_3$) δ 8.68 (s, 8H, H^3), 8.07 (d, J = 8.1 Hz, 4H, H^7), 8.00 (d, J = 8.1 Hz, 4H, $H^{7'}$), 7.88 (t, J = 7.6 Hz, 8H, H^8 + $H^{8'}$). ^{13}C{^{1}H} NMR (126 MHz, $CDCl_3$) δ 144.0 (C^4), 141.3 (C^6), 135.9 (C^7), 135.3 ($C^{7'}$), 132.0 (C^3), 130.1 (C^8), 130.0 ($C^{8'}$), 122.4 (C^9), 120.9 (C^5). FT-IR (cm^{-1}) 1967 ($\tilde{\nu}_{CO}$). UV-Vis (DCM) λ (nm) 410 (ε = 180 817), 526 (ε = 17 642), 561 (ε = 3 589). HRMS (MALDI-TOF +, DCTB) m/z: $[M - CO]^+$ Calcd for $C_{44}H_{24}Br_4N_4Ru$ 1029.7755; found 1029.7692. m/z: $[M]^+$ 1057.9989 Calcd for $C_{45}H_{24}Br_4N_4ORu$ 1057.7704; found 1057.7622.

General method for the synthesis of *RuP-Br* complexes with nitrogen ligands

RuP-Br and the corresponding ligand were dissolved in DCM and the resulting mixture was stirred at room temperature for 30 minutes. The crude was concentrated and then *n*-hexane

was added to the mixture. The corresponding complex precipitates as a garnet solid, which was separated from the solution by centrifugation and dried under vacuum.

RuP-Br·py. General method was followed using *RuP-Br* (50 mg, 0.046 mmol), DCM (10 mL) and pyridine (10 μL, 0.070 mmol). *RuP-Br·py* was isolated as a garnet solid (51 mg, 98% yield). ^{1}H NMR (500 MHz, $CDCl_3$) δ 8.60 (s, 8H, H^3), 8.06 (dd, *J* = 8.0, 2.1 Hz, 4H, H^7), 7.91 (dd, *J* = 8.0, 2.1 Hz, 4H, $H^{7'}$), 7.87 (dd, *J* = 8.0, 2.0 Hz, 4H, H^8), 7.82 (dd, *J* = 8.0, 2.0 Hz, 4H, $H^{8'}$), 6.10 (t, *J* = 7.6 Hz, 1H, H^d), 5.20 (t, *J* = 7.0 Hz, 2H, H^c), 1.47 (d, *J* = 5.2 Hz, 2H, H^b). $^{13}C\{^{1}H\}$ NMR (101 MHz, $CDCl_3$) δ 179.5 (C^1), 143.9 (C^b), 143.6 (C^4), 141.5 (C^6), 135.8 (C^7), 135.4 ($C^{7'}$), 134.6 (C^d), 132.0 (C^3), 130.0 (C^8), 129.7 ($C^{8'}$), 122.2 (C^9), 121.8 (C^c), 120.6 (C^5). FT-IR (cm^{-1}) 1956 ($\tilde{\nu}_{CO}$). UV-Vis (DCM) λ (nm) 410 (ε = 132 195), 528 (ε = 19 263), 570 (ε = 3 946). HRMS (MALDI-TOF +, DCTB) m/z: [M – CO - py]$^+$ Calcd for $C_{44}H_{24}Br_4N_4Ru$ 1029.7755; found 1029.7677. m/z: [M – py]$^+$ Calcd for $C_{45}H_{24}Br_4N_4ORu$ 1057.7704; found 1057.7605. m/z: [M – CO]$^+$ Calcd for $C_{49}H_{29}Br_4N_5Ru$ 1108.8178; found 1108.8124.

(RuP-Br)₂·DABCO. General method was followed using *RuP-Br* (40 mg, 0.037 mmol), triethylenediamine (2 mg, 0.018 mmol) and DCM (10 mL). *(RuP-Br)₂·DABCO* was isolated as a garnet solid (39 mg, 95% yield). ^{1}H NMR (500 MHz, $CDCl_3$) δ 8.30 (s, 16H, H^3), 7.82 (dd, *J* = 7.9, 2.1 Hz, 8H, $H^{8'}$), 7.78 (dd, *J* = 7.9, 2.0 Hz, 8H, H^8), 7.74 (dd, *J* = 7.9, 2.0 Hz, 8H, H^7), 7.39 (dd, *J* = 7.9, 2.1 Hz, 8H, $H^{7'}$), -5.43 (s, 12H, H^b). ^{13}C (in) δ 135.5 (C^7), 134.7 ($C^{7'}$), 132.0 (C^3), 130.2 (C^8), 129.7 ($C^{8'}$), 38.9 (C^b). FT-IR (cm^{-1}) 1985 ($\tilde{\nu}_{CO}$). UV-Vis (DCM) λ (nm) 409 (ε = 137 886), 527 (ε = 16 414), 560 (ε = 4 707). HRMS (MALDI-TOF +, DCTB) m/z: [M – 2CO – DABCO – RuP-Br]$^+$ Calcd for $C_{44}H_{24}Br_4N_4Ru$ 1029.7755; found 1029.7732. m/z [M – 2CO – RuP-Br]$^+$ Calcd for $C_{50}H_{36}Br_4N_6Ru$ 1141.8757; found 1141.8722.

(RuP-Br)₂·daz. General method was followed using *RuP-Br* (50 mg, 0.046 mmol), pyrazine (2 mg, 0.023 mmol) and DCM (10 mL). *(RuP-Br)₂·daz* was isolated as a garnet solid (50 mg, 100% yield). ^{1}H NMR (500 MHz, $CDCl_3$) δ 8.33 (s, 16H, H^3), 7.81 (s, 16H, H^7 + H^8), 7.74 (d, *J* = 7.9 Hz, 8H, $H^{8'}$), 7.33 (d, *J* = 7.9 Hz, 8H, $H^{7'}$), -0.62 (s, 4H, H^b). $^{13}C\{^{1}H\}$ NMR (126 MHz, $CDCl_3$) δ 142.9 (C^4), 140.5 (C^6), 136.6 (C^b), 135.6 (C^7), 135.0 ($C^{7'}$), 131.8 (C^3), 130.2 (C^8), 129.8 ($C^{8'}$), 122.6 (C^9), 120.3 (C^5). FT-IR (cm^{-1}) 1959 ($\tilde{\nu}_{CO}$). UV-Vis (DCM) λ (nm) 409 (ε = 237 779), 529 (ε = 31 004), 562 (ε = 7 206). HRMS (MALDI-TOF +, DCTB) m/z: [M – 2CO – daz – RuP-Br]$^+$ Calcd for $C_{44}H_{24}Br_4N_4Ru$ 1029.775; found 1029.7711. m/z [M – COM -daz – RuP-Br]$^+$ Calcd for $C_{45}H_{24}Br_4N_4ORu$ 1057.7704; found 1057.7735.

(RuP-Br)₂·bpy. General method was followed using *RuP-Br* (50 mg, 0.046 mmol), 4,4'-bipyridine (3.6 mg, 0.023 mmol) and DCM (10 mL). *(RuP-Br)₂·bpy* was isolated as a garnet solid (49 mg, 95% yield). ^{1}H NMR (500 MHz, $CDCl_3$) δ 8.44 (s, 16H, H^3), 7.94 (dd, *J* = 8.0, 2.2 Hz, 8H, H^7), 7.80 (dd, *J* = 8.0, 2.2 Hz, 8H, H^8), 7.70 (dd, *J* = 7.9, 2.2 Hz, 8H, $H^{8'}$), 7.62 (dd, *J* = 7.9, 2.2 Hz, 1H, $H^{7'}$), 4.39 (d, *J* = 7.2 Hz, 4H, H^c), 1.07 (d, *J* = 7.2 Hz, 4H, H^b). $^{13}C\{^{1}H\}$ NMR (101 MHz, $CDCl_3$) δ 144.1 (C^b), 143.2 (C^4), 140.9 (C^6 + C^d), 135.6 (C^7), 134.9 ($C^{7'}$), 131.7 (C^3), 129.8 (C^8), 129.4 ($C^{8'}$), 122.1 (C^9), 120.2 (C^5), 118.1 (C^c). FT-IR (cm^{-1}) 1995 ($\tilde{\nu}_{CO}$). UV-Vis (DCM) λ (nm) 408 (ε = 276 155), 529 (ε = 36 173), 566 (ε = 11 081). HRMS (MALDI-TOF +, DCTB) m/z: [M – 2CO – bpy – RuP-Br]+ Calcd for $C_{44}H_{24}Br_4N_4Ru$ 1029.7755; found 1029.7672. m/z: [M – 2CO – RuP-Br]$^+$ Calcd for $C_{54}H_{32}Br_4N_6Ru$ 1185.8445; found 1185.8415.

(RuP-Br)₂·dpyb. General method was followed using *RuP-Br* (50 mg, 0.046 mmol), 1,4-di(pyridine-4-yl)benzene (5 mg, 0.023 mmol) and DCM (10 mL). *(RuP-Br)₂·dpyb* was isolated as a garnet solid (53 mg, 98% yield). ^{1}H NMR (500 MHz, $CDCl_3$) δ 8.53 (s, 16H, H^3), 8.01 (dd, *J* = 7.9, 2.0 Hz, 8H, H^7), 7.83 (dd, *J* = 7.9, 2.0 Hz, 8H, H^8), 7.81 (dd, *J* = 7.9, 2.0 Hz, 8H, $H^{7'}$), 7.73 (dd, *J* = 7.9, 2.0 Hz, 8H, $H^{8'}$), 6.21 (s, 4H, H^f), 5.16 (d, *J* = 7.0 Hz, 4H, H^c), 1.36 (d, *J* = 7.0 Hz, 4H, H^b). $^{13}C\{^1H\}$ NMR (101 MHz, $CDCl_3$) δ 144.7 (C^d-in), 144.1 (C^b), 143.5 (C^4), 141.3 (C^6), 136.1 (C^e-in) 135.8 (C^7), 135.3 ($C^{7'}$), 132.0 (C^3), 130.0 (C^8), 129.6 ($C^{8'}$), 126.4 (C^f), 122.2 (C^9), 120.5 (C^5), 119.1 (C^c). FT-IR (cm^{-1}) 1965 ($\tilde{\nu}_{CO}$). UV-Vis (DCM) λ (nm) 410 (ε = 335 460), 531 (ε = 35 000), 564 (ε = 9 478). HRMS (MALDI-TOF +, DCTB) m/z: [M – 2CO – dpyb – RuP-Br]$^+$ Calcd for $C_{44}H_{24}Br_4N_4Ru$ 1029.775; found 1029.7677. m/z [M – 2CO – RuP-Br]$^+$ Calcd for $C_{60}H_{36}Br_4N_6Ru$ 1261.8760; found 1261.8769.

General method for the Suzuki coupling

The corresponding *RuP-Br* complex, the polycyclic aromatic hydrocarbon boronic ester, [$PdCl_2$(dppf)] and ^tBuONa were mixed in a microwave flask under inert atmosphere. Dry toluene was then added. The resulting solution was irradiated in a microwave reactor at 135°C for 30 minutes. The solvent was removed under vacuum and the crude was extracted with DCM/H_2O, the organic layer was concentrated, and *n*-hexane was added to precipitate the complex as a garnet solid which was separated from the solution by centrifugation, washed with portions of *n*-hexane and dried under vacuum.

RuP-pyr·py. General method was followed using *RuP-Br·py* (10 mg, 0.009 mmol), Bpin-pyr (12 mg, 0.037 mmol), [$PdCl_2$(dppf)] (2.6 mg, 0.004 mmol), ^tBuONa (10 mg, 0.108 mmol) and dry toluene (3.5 mL). *RuP-pyr·py* was isolated as a garnet solid (8 mg, 53% yield). ^1H NMR (500 MHz, $CDCl_3$) δ 8.99 (s, 8H, H^3), 8.66 (d, *J* = 9.2 Hz, 4H, H^{19}), 8.54 (dd, *J* = 7.6, 1.8 Hz, 4H, H^7), 8.42 (d, *J* = 7.8 Hz, 4H, H^{12}), 8.39 (d, *J* = 7.8 Hz, 4H, H^{11}), 8.35 (dd, *J* = 7.6, 1.8 Hz, 4H, $H^{7'}$), 8.28 (d, *J* = 7.6 Hz, 8H, H^{15} + H^{17}), 8.24 (d, *J* = 9.2 Hz, 4H, H^{18}), 8.22 – 8.17 (m, 8H, H^{13} + H^{14}), 8.11 – 8.06 (m, 8H, H^8 + H^{16}), 8.03 (dd, *J* = 7.6, 1.8 Hz, 4H, $H^{8'}$), 6.19 (tt, *J* = 7.4, 1.6 Hz, 1H, H^d), 5.33 (t, *J* = 7.4 Hz, 2H, H^c), 1.78 – 1.74 (m, 2H, H^b). $^{13}C\{^1H\}$ NMR (126 MHz, $CDCl_3$) δ 144.3 (C^b), 144.0 (C^4), 141.8 (C^6), 140.3 (C^9), 137.9 (C^{10}), 134.7 (C^7), 134.6 (C^d), 134.4 ($C^{7'}$), 132.3 (C^3), 131.8 (C^{22}), 131.3 (C^{23}), 131.0 (C^{21}), 129.1 (C^8), 128.9 (C^{20}), 128.8 ($C^{8'}$), 128.2 (C^{11}), 127.9 (C^{18}), 127.7 (C^{13} + C^{14}), 126.3 (C^{16}), 125.7 (C^{19}), 125.41 (C^{25}), 125.39 (C^{15} or C^{17}), 125.2 (C^{24}), 125.13 (C^{15} or C^{17}), 125.06 (C^{12}), 121.8 (C^5), 121.7 (C^c). FT-IR (cm^{-1}) 1945 ($\tilde{\nu}_{CO}$). UV-Vis (DCM) λ (nm) 416 (ε = 191 257), 531 (ε = 19 299), 566 (ε = 7 530). HRMS (MALDI-TOF +, DCTB) m/z: [M – CO – py]$^+$ Calcd for $C_{108}H_{60}N_4Ru$ 1514.3892; found 1514.3902. m/z: [M – py]$^+$ Calcd for $C_{109}H_{60}N_4ORu$ 1542.3841; found 1542.3827. LRMS (MALDI-TOF +. DCTB) m/z: [M – CO]$^+$ Calc. for $C_{113}H_{65}N_5Ru$ 1593.43; found 1593.30. m/z: [M]$^+$ Calcd for $C_{114}H_{65}N_5ORu$ 1621.43; found 1621.30.

(RuP-pyr)₂·DABCO. General method was followed using *(RuP-Br)₂·DABCO* (20 mg, 0.013 mmol), Bpin-pyr (37 mg, 0.110 mmol), [$PdCl_2$(dppf)] (8 mg, 0.011 mmol), ^tBuONa (30 mg, 0.312 mmol) and dry toluene (2.3 mL). *(RuP-pyr)₂·DABCO* was isolated as a garnet solid (13 mg, 32% yield). ^1H NMR (500 MHz, $CDCl_3$) δ 8.72 (s, 16H, H^3), 8.48 (d, *J* = 9.3 Hz, 8H, H^{19}), 8.32 (dd, *J* = 7.4, 1.9 Hz, 8H, H^7), 8.17 (dd, *J* = 7.1, 1.2 Hz, 8H, H^{pyr}), 8.09 – 8.05 (m, 24H, $H^{8'}$ + 2H^{pyr}), 7.99 (d, *J* = 9.3 Hz, 8H, H^{18}), 7.95 (dd, *J* = 7.4, 1.9 Hz, 8H, H^8), 7.91 – 7.86 (m, 24H, 3Hpyr), 7.84 – 7.80 (m, 16H, $H^{7'}$ + H^{pyr}), -5.09 (s, 12H, H^b). C (in) δ 134.1 ($C^{7'}$), 133.6 (C^7), 132.2 (C^3), 129.1 (C^8), 128.5 ($C^{8'}$), 127.6 (C^{18}), 127.5 (C^{pyr}), 127.4 (2C^{pyr}), 125.9 (C^{pyr}), 125.1 (C^{pyr}), 124.9 (C^{19}), 124.8 (C^{pyr}), 124.7 (C^{pyr}), 39.0 (C^b).

FT-IR (cm^{-1}) 1946 ($\tilde{\nu}_{CO}$). UV-Vis (DCM) λ (nm) 414 (ε = 106 653), 531 (ε = 8 943), 564 (ε = 4 245). HRMS (MALDI-TOF +, DCTB) m/z: [M – 2CO – DABCO – RuP-pyr]$^+$ Calcd for $C_{108}H_{60}N_4Ru$ 1514.3892; found 1514.3902. m/z: [M – CO – DABCO – RuP-pyr]$^+$ Calcd for $C_{109}H_{60}N_4ORu$ 1542.3841; found 1542.3827. m/z: [M – 2CO – RuP-pyr]$^+$ Calcd for $C_{114}H_{72}N_6Ru$ 1626.4894; found 1626.4873.

(RuP-pyr)$_2$·bpy. General method was followed using *(RuP-Br)$_2$·bpy* (25 mg, 0.011 mmol), Bpin-pyr (31 mg, 0.094 mmol), [PdCl$_2$(dppf)] (6 mg, 0.009 mmol), ^{t}BuONa (25 mg, 0.264 mmol) and dry toluene (2.4 mL). *(RuP-pyr)$_2$·bpy* was isolated as a garnet solid (20 mg, 57% yield). ^{1}H NMR (500 MHz, $CDCl_3$) δ 8.81 (s, 16H, H^3), 8.52 (d, J = 9.1 Hz, 8H, H^{19}), 8.39 (d, J = 7.3 Hz, 8H, H^7), 8.31 (d, J = 7.7 Hz, 8H, H^{12}), 8.27 (d, J = 7.7 Hz, 8H, H^{11}), 8.24 (d, J = 7.3 Hz, 8H, H^{17}), 8.17 (d, J = 7.3 Hz, 8H, H^{15}), 8.15 (s, 16H, H^{13} + H^{14}), 8.14 (d, J = 9.1 Hz, 8H, H^{18}) 8.08 (d, J = 7.3 Hz, 8H, $H^{7'}$), 8.02 (t, J = 7.3 Hz, 8H, H^{16}), 7.98 (d, J = 7.3 Hz, 8H, H^8), 7.87 (d, J = 7.3 Hz, 8H, $H^{8'}$), 4.59 (d, J = 7.0 Hz, 4H, H^c), 1.39 (d, J = 7.0 Hz, 4H, H^b). C (in) δ 144.4 (C^b), 143.6 (C^4), 141.4 (C^6), 141.1 (C^d), 140.1 (C^9), 137.5 (C^{10}), 134.5 (C^7), 134.0 ($C^{7'}$), 132.0 (C^3), 131.5 (C^{22}), 131.0 (C^{23}), 130.7 (C^{21}) 128.9 (C^8), 128.6 (C^{20}), 128.5 ($C^{8'}$), 127.9 (C^{11}), 127.7 (C^{18}), 127.5 (C^{13} + C^{14}), 126.1 (C^{16}), 125.3 (C^{17} + C^{19}), 125.1 (C^{25}), 124.9 (C^{15}), 124.8 (C^{12}), 121.5 (C^5) 118.1 (C^c). FT-IR (cm^{-1}) 1940 ($\tilde{\nu}_{CO}$). UV-Vis (DCM) λ (nm) 412 (ε = 292 522), 530 (ε = 33 336), 565 (ε = 14 072). HRMS (MALDI-TOF +, DCTB) m/z: [M – 2CO – bpy – RuP-pyr]$^+$ Calcd for $C_{108}H_{60}N_4Ru$ 1514.3892; found 1514.3836. m/z: [M – CO – bpy – RuP-pyr]$^+$ Calcd for $C_{109}H_{60}N_4ORu$ 1542.3841; found 1542.3805. LRMS (MALDI-TOF +, DCTB) m/z: [M – 2CO – RuP-pyr]$^+$ Calcd for $C_{118}H_{68}N_6Ru$ 1670.46; found 1671.47. m/z: [M – CO – RuP-pyr]$^+$ Calcd for $C_{119}H_{68}N_6ORu$ 1698.45; found 1697.47.

(RuP-pyr)$_2$·dpyb. General method was followed using *(RuP-Br)$_2$·dpyb* (10 mg, 0.004 mmol), Bpin-pyr (12 mg, 0.035 mmol), [PdCl$_2$(dppf)] (3 mg, 0.003 mmol), ^{t}BuONa (10 mg, 0.102 mmol) and dry toluene (1 mL). *(RuP-pyr)$_2$·dpyb* was isolated as a garnet solid (9 mg, 65% yield). ^{1}H NMR (500 MHz, $CDCl_3$) δ 8.91 (s, 16H, H^3), 8.58 (d, J = 9.2 Hz, 8H, H^{19}), 8.48 (dd, J = 7.5, 1.8 Hz, 8H, H^7), 8.37 (d, J = 7.6 Hz, 8H, H^{12}), 8.33 (d, J = 7.6 Hz, 8H, H^{11}), 8.28 – 8.22 (m, 24H, $H^{7'}$ + H^{15} + H^{17}), 8.21 – 8.15 (m, 24H, H^{13} + H^{14} + H^{18}), 8.06 (t, J = 7.4 Hz, 8H, H^{16}), 8.04 (dd, J = 7.5, 1.8 Hz, 8H, H^8) 7.92 (dd, J = 7.4, 1.8 Hz, 8H, $H^{8'}$), 6.32 (s, 4H, H^f), 5.30 (d, J = 7.5 Hz, 4H, H^c), 1.65 (d, J = 7.5 Hz, 4H, H^b). $^{13}C\{^1H\}$ NMR (126 MHz, $CDCl_3$) δ 144.3 (C^b), 143.8 (C^4), 141.6 (C^6), 140.1 (C^9-in), 137.6 (C^{10}), 136.3 (C^d or C^e-in), 134.5 (C^7), 134.2 ($C^{7'}$), 132.1 (C^3), 131.6 (C^{22}), 131.1 (C^{23}), 130.8 (C^{21}), 128.9 (C^8), 128.7 (C^{20}), 128.5 ($C^{8'}$), 127.9 (C^{11}), 127.7 (C^{18}), 127.6 (C^{13} + C^{14}), 126.3 (C^f), 126.1 (C^{16}), 125.4 (C^{19}), 125.3 (C^{15} or C^{17}), 125.2 (C^{25}), 125.1 (C^{24}), 125.0 (C^{15} or C^{17}), 124.9 (C^{12}), 121.5 (C^5), 119.0 (C^c). FT-IR (cm^{-1}) 1951 ($\tilde{\nu}_{CO}$). UV-Vis (DCM) λ (nm) 416 (ε = 230 604), 533 (ε = 21 516), 567 (ε = 8 996). HRMS (MALDI-TOF +, DCTB) m/z: [M – CO – dpyb -RuP-pyr]$^+$ Calcd for $C_{109}H_{60}N_4ORu$ 1542.3841; found 1542.3855. m/z: [M – 2CO – RuP-pyr]$^+$ Calc. for $C_{124}H_{72}N_6Ru$ 1746.4896; found 1746.4863. LRMS (MALDI-TOF +, DCTB) m/z: [M – CO -RuP-pyr]$^+$ Calcd for $C_{125}H_{72}N_6ORu$ 1774.48; found 1774.25. m/z: [M – CO]$^+$ Calcd for $C_{233}H_{132}N_{10}ORu_2$ 3289.88; found 3288.35. m/z: [M]$^+$ Calcd for $C_{234}H_{132}N_{10}O_2Ru_2$ 3317.87; found 3314.43.

RuP-cor·py. General method was followed using *RuP-Br·py* (14 mg, 0.013 mmol), Bpin-cor (20 mg, 0.053 mmol), [PdCl$_2$(dppf)] (4 mg, 0.005 mmol), ^{t}BuONa (15 mg, 0.154 mmol) and dry toluene (5 mL). *RuP-cor·py* was isolated as a garnet solid (18 mg, 82% yield). ^{1}H NMR (500 MHz, $CDCl_3$) δ 8.92 (s, 8H, H^3), 8.50 (dd, J = 7.7, 1.8 Hz, 4H, H^7), 8.32 (dd, J = 7.6, 1.8 Hz, 4H, $H^{7'}$), 8.26 (s, 4H, H^{11}), 8.22 (d, J = 8.7 Hz, 4H, H^{19}), 8.21 (dd, J = 7.7, 1.8 Hz, 4H, H^8), 8.17 (dd, J = 7.6, 1.8 Hz, 4H, $H^{8'}$), 8.00 (d, J = 8.6 Hz, 4H, H^{12}), 7.97 (d, J = 8.7 Hz, 4H, H^{18}), 7.94 (d, J = 8.6 Hz, 4H, H^{13}), 7.92

– 7.87 (m, 16H, H^{14} + H^{15} + H^{16} + H^{17}), 6.21 – 6.17 (m, 1H, H^{d}), 5.32 (t, *J* = 7.2 Hz, 2H, H^{c}), 1.72 (m, 2H, H^{b}). $^{13}C\{^{1}H\}$ NMR (126 MHz, $CDCl_3$) *δ* 144.1 (C^{b}), 143.8 (C^{4}), 142.1 (C^{10}), 141.7 (C^{6}), 138.7 (C^{9}), 136.5 (C^{26}), 136.3 (C^{29}), 136.0 (C^{25}), 135.6 (C^{28}), 135.5 (C^{27}), 134.9 (C^{7}), 134.7 ($C^{7'}$), 134.4 (C^{d}-in) 132.1 (C^{3}), 131.1 (C^{21}), 131.0 (C^{24}), 130.9 (C^{22}), 129.9 (C^{20}), 128.2 (C^{8}), 128.0 ($C^{8'}$), 127.6 (C^{18}), 127.5 (C^{13}), 127.4 (C^{15} or C^{16}), 127.3 (C^{19}), 127.2 (C^{14}), 127.12 (C^{12}), 127.08 (C^{15} or C^{16}), 127.0 (C^{17}), 126.2 (C^{11}), 121.7 (C^{c}), 121.4 (C^{5}). FT-IR (cm^{-1}) 1939 ($\tilde{\nu}_{CO}$). UV-Vis (DCM) λ (nm) 417 (ε = 204 081), 533 (ε = 20 947), 568 (ε = 7 853). HRMS (MALDI-TOF +, DCTB) m/z: $[M – CO – py]^+$ Calcd for $C_{124}H_{60}N_4Ru$ 1706.3895; found 1706.3825. m/z: $[M – py]^+$ Calcd for $C_{125}H_{60}N_4ORu$ 1734.3845; found 1734.3815. LRMS (MALDI-TOF +, DCTB) m/z: $[M – CO]^+$ Calc for $C_{129}H_{65}N_5Ru$ 1785.43; found 1785.20. m/z: $[M]^+$ Calcd for $C_{130}H_{65}N_5ORu$ 1813.20; found 1813.43. C_{60}@RuP-cor·py: LRMS (MALDI-TOF +, DCTB) m/z: $[M – CO – py]^+$ Calcd for $C_{184}H_{60}N_4Ru$ 2427.39; found 2430.83. m/z: $[M – CO]^+$ Calcd for $C_{189}H_{65}N_5Ru$ 2506.43; found 2509.10. m/z: $[M]^+$ Calcd for $C_{190}H_{65}N_5ORu$ 2534.43; found 2532.50.

***(RuP-cor)₂·bpy*.** General method was followed using *(RuP-Br)₂·bpy* (14 mg, 0.006 mmol), Bpin-cor (20 mg, 0.053 mmol), [$PdCl_2$(dppf)] (4 mg, 0.005 mmol), ^{t}BuONa (15 mg, 0.154 mmol) and dry toluene (2.5 mL). *(RuP-cor)₂·bpy* was isolated as a garnet solid (14 mg, 64% yield). ^{1}H NMR (500 MHz, $CDCl_3$) *δ* 8.75 (s, 16H, H^{3}), 8.36 (d, *J* = 7.5 Hz, 8H, H^{7}), 8.16 (s, 8H, H^{11}), 8.13 – 8.08 (m, 16H, H^{8} + H^{19}), 8.06 – 8.02 (m, 16H, $H^{7'}$ + $H^{8'}$), 7.92 – 7.79 (m, 56H, H^{12} - H^{18}), 4.60 (d, *J* = 7.2 Hz, 4H, H^{c}), 1.36 (d, *J* = 7.2 Hz, 4H, H^{b}). C (in) *δ* 144.4 (C^{b}), 143.6 (C^{4}), 141.7 (C^{6} + C^{10}), 138.6 (C^{9}), 136.6 (C^{26}), 135.5 (C^{27}), 134.7 (C^{7}), 134.3 ($C^{7'}$), 130.9 (C^{24}), 129.7 (C^{20}), 128.2 (C^{8}), 127.9 – 126.6 (C^{13} – C^{18}), 127.8 ($C^{8'}$), 127.13 (C^{19}), 127.08 (C^{12}), 126.2 (C^{11}), 118.2 (C^{c}). FT-IR (cm^{-1}) 1947 ($\tilde{\nu}_{CO}$). UV-Vis (DCM) λ (nm) 415 (ε = 380 291), 534 (ε = 32 945), 568 (ε = 16 448). HRMS (MALDI-TOF +, DCTB) m/z: $[M – 2CO – bpy – RuP\text{-}cor]^+$ Calcd for $C_{124}H_{60}N_4Ru$ 1706.3895; found 1706.3865. LRMS (MALDI-TOF +, DCTB) m/z: $[M – 2CO – RuP\text{-}cor]^+$ Calcd for $C_{134}H_{68}N_6Ru$ 1862.46; found 1861.58. m/z: $[M – CO – RuP\text{-}cor]^+$ Calcd for $C_{135}H_{68}N_6ORu]^+$ 1890.45; found 1891.29.

***(RuP-cor)₂·dpyb*.** General method was followed using *(RuP-Br)₂·dpyb* (15 mg, 0.006 mmol), Bpin-cor (20 mg, 0.053 mmol), [$PdCl_2$(dppf)] (4 mg, 0.005 mmol), ^{t}BuONa (15 mg, 0.154 mmol) and dry toluene (2.5 mL). *(RuP-cor)₂·dpyb* was isolated as a garnet solid (13 mg, 60% yield). ^{1}H NMR (500 MHz, $CDCl_3$) *δ* 8.83 (s, 16H, H^{3}), 8.43 (dd, *J* = 7.5, 1.8 Hz, 8H, H^{7}), 8.21 (dd, *J* = 7.6, 2.2 Hz, 8H, $H^{7'}$), 8.19 (s, 8H, H^{11}), 8.15 (dd, *J* = 7.5, 1.8 Hz, 8H, H^{8}), 8.14 (d, *J* = 8.6 Hz, 8H, H^{19}), 8.07 (dd, *J* = 7.6, 2.2 Hz, 8H, $H^{8'}$), 7.95 (d, *J* = 8.6 Hz, 8H, H^{12}), 7.90 (d, *J* = 8.6 Hz, 16H, H^{13} + H^{18}), 7.87 (m, 32H, H^{14} – H^{17}), 6.32 (s, 4H, H^{f}), 5.29 (d, *J* = 7.6 Hz, 4H, H^{c}), 1.61 (d, *J* = 7.6 Hz, 4H, H^{b}). $^{13}C\{^{1}H\}$ NMR (126 MHz, $CDCl_3$) *δ* 144.2 (C^{b}), 143.7 (C^{4}), 142.0 (C^{6}), 141.6 (C^{10}), 138.6 (C^{9}), 136.5 (C^{26}), 136.3 (C^{e}), 136.0 (C^{25}), 135.50 (C^{27} or C^{28}), 135.47 (C^{27} or C^{28}), 134.8 (C^{7}), 134.4 ($C^{7'}$), 132.0 (C^{3}), 131.1 (C^{23}), 131.0 (C^{21} + C^{24}), 130.8 (C^{22}), 129.8 (C^{20}), 128.14(C^{8}), 127.9 ($C^{8'}$), 127.6 (C^{13} or C^{18}), 127.5 (C^{13} or C^{18}), 127.4 (C^{15} or C^{16}), 127.2 (C^{14} or C^{17}), 127.1 (C^{12} + C^{15} or C^{16}), 127.0 (C^{14} or C^{17}), 126.3 (C^{f}), 126.2 (C^{11}), 121.4 (C^{5}), 119.0 (C^{c}). FT-IR (cm^{-1}) 1941 ($\tilde{\nu}_{CO}$). UV-Vis (DCM) λ (nm) 416 (ε = 316 439), 533 (ε = 26 131), 568 (ε = 10 585). HRMS (MALDI-TOF +, DCTB) m/z: $[M – 2CO – dpyb – RuP\text{-}cor]^+$ Calcd for $C_{124}H_{60}N_4Ru$ 1706.3895; found 1706.3872. m/z: $[M – CO – dpyb – RuP\text{-}cor]^+$ Calcd for $C_{125}H_{60}N_4ORu$ 1734.3845; found 1734.3865. LRMS (MALDI-TOF +, DCTB) m/z: $[M – 2CO – RuP\text{-}cor]^+$ Calcd for $C_{140}H_{72}N_6Ru$ 1938.49; found 1940.86. m/z: $[M – CO]^+$ Calcd for $C_{265}H_{132}N_{10}ORu_2$ 3673.88; found 3674.23.

Discussion about the synthetic strategy

The global synthetic protocol relies on multi–Suzuki C-C cross couplings between the parent brominated complex and a boronate ester of corannulene. Although it appears more pragmatic to first carry out such a reaction on the intermediate **RuP-Br** to yield porphyrin **RuP-cor** and subsequently form the dimer by adding 0.5 equiv. of the corresponding bidentate ligand (Figure S 1a, Pathway A), this method proved cumber-some due to competition with coordinated cosolvent used in corannulene coupling (either DMSO or DMF). Consequently, this resulted in the non-negligible presence of monomer **RuP-cor·bpy** (or **·dpyb**) at the correct stoichiometry (Figure S 1b). Conversely, the dimerization of compound **RuP-Br** occurred readily, prompting the adoption of an octuple Suzuki reaction on **(RuP-Br)₂·bpy** and **(RuP-Br)₂·dpyb** homobimetallic complexes (Figure S 1a, Pathway B) furnishing targeted final compounds **(RuP-cor)₂·bpy** and **(RuP-cor)₂·dpyb** in good yields (64% and 60%, respectively). The same observations were also made for pyrene derivatives **RuP-pyr**.

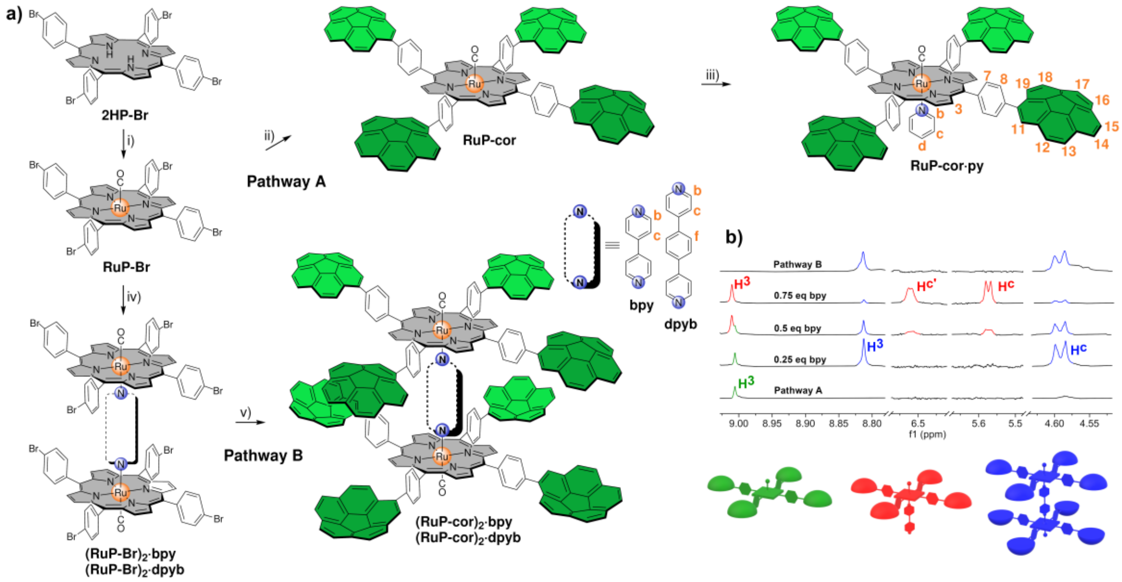

Figure S 1. (a) Synthetic pathways to prepare porphyrin complexes **RuP-cor·py**, **(RuP-cor)$_2$·bpy**, and **(RuP-cor)$_2$·dpyb** with atom numbering. Reagents and conditions: *i*) $Ru_3(CO)_{12}$, toluene, reflux; *ii*) 4 equiv. Bpin-cor, $[PdCl_2(dppf)]$, ^{t}BuONa, toluene/DMSO (or DMF) 10:1, MW, 135°C; *iii*) 1 equiv. py, DCM, rt; *iv*) 0.5 equiv. bidentate ligand, DCM, rt; *v*) 8 equiv. Bpin-cor, $[PdCl_2(dppf)]$, ^{t}BuONa, toluene, MW, 135°C. (b) ^{1}H-NMR spectra (500 MHz, $CDCl_3$) of relevant protons during dimer **(RuP-cor)$_2$·bpy** formation via bpy titration after **RuP-cor** preparation (Pathway A) compared with the targeted compound after octa-Suzuki C-C cross coupling (Pathway B). Color coding: **RuP-cor** (DMF or DMSO coordinated, green), **RuP-cor·bpy** (red), **(RuP-cor)$_2$·bpy** (blue). Note: **RuP-cor·py** can also be prepared via Pathway B.

NMR spectra

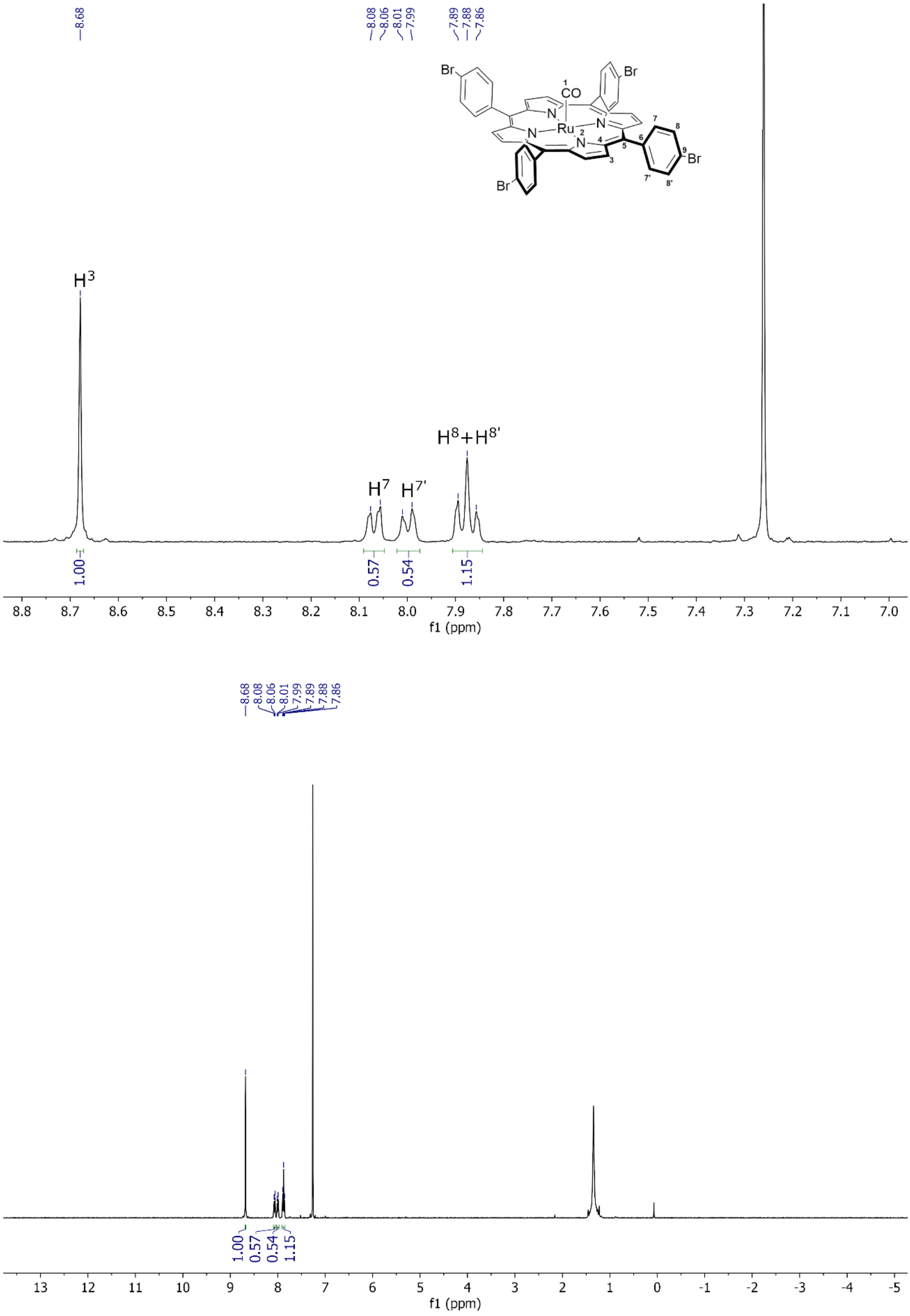

Figure S 2. ^{1}H NMR spectrum of compound **RuP-Br** (400 MHz, $CDCl_3$); selected regions (above) and full spectrum (below).

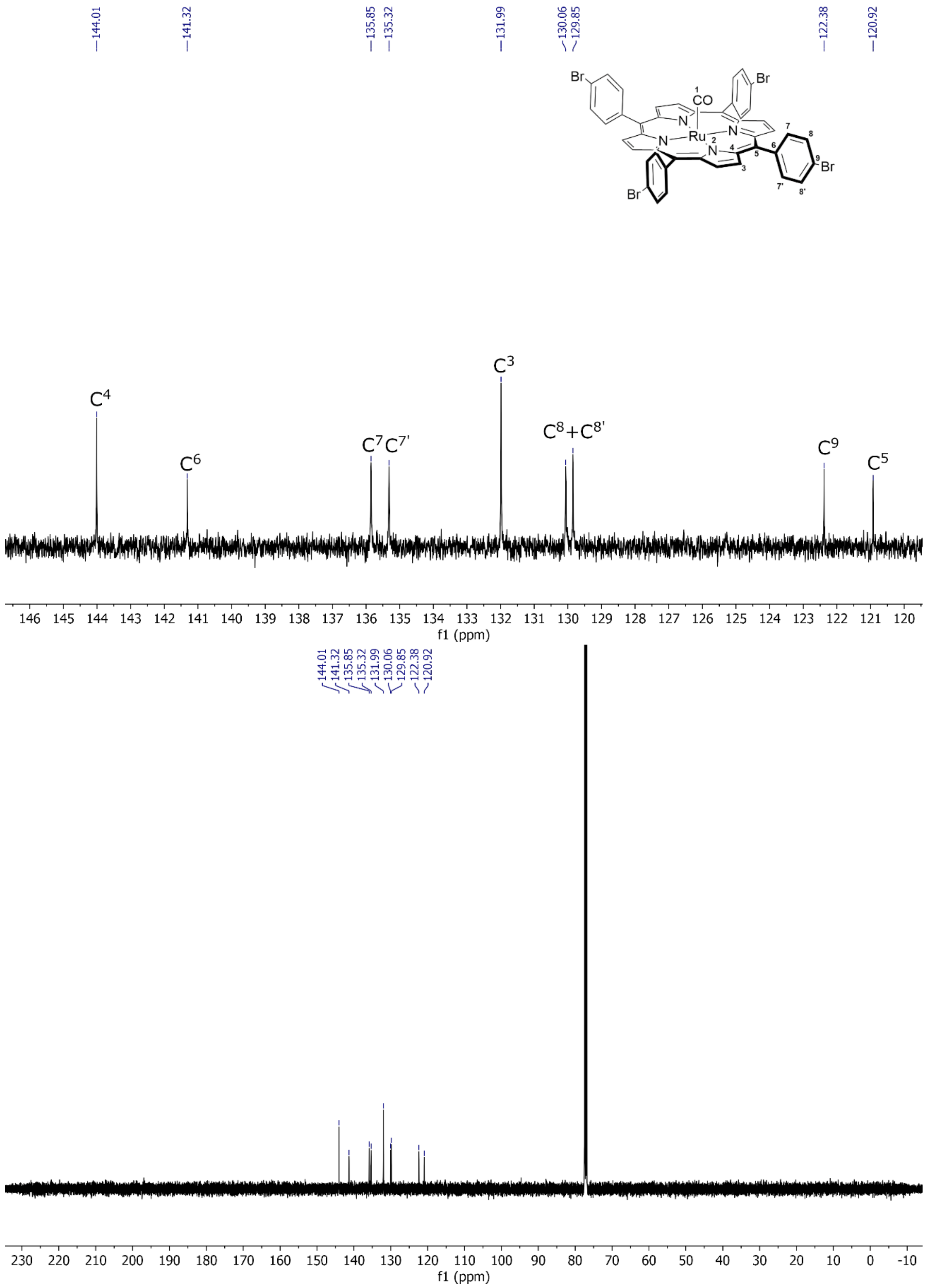

Figure S 3. $^{13}C\{^{1}H\}$ NMR spectrum of compound **RuP-Br** (126 MHz, $CDCl_3$); selected regions (above) and full spectrum (below).

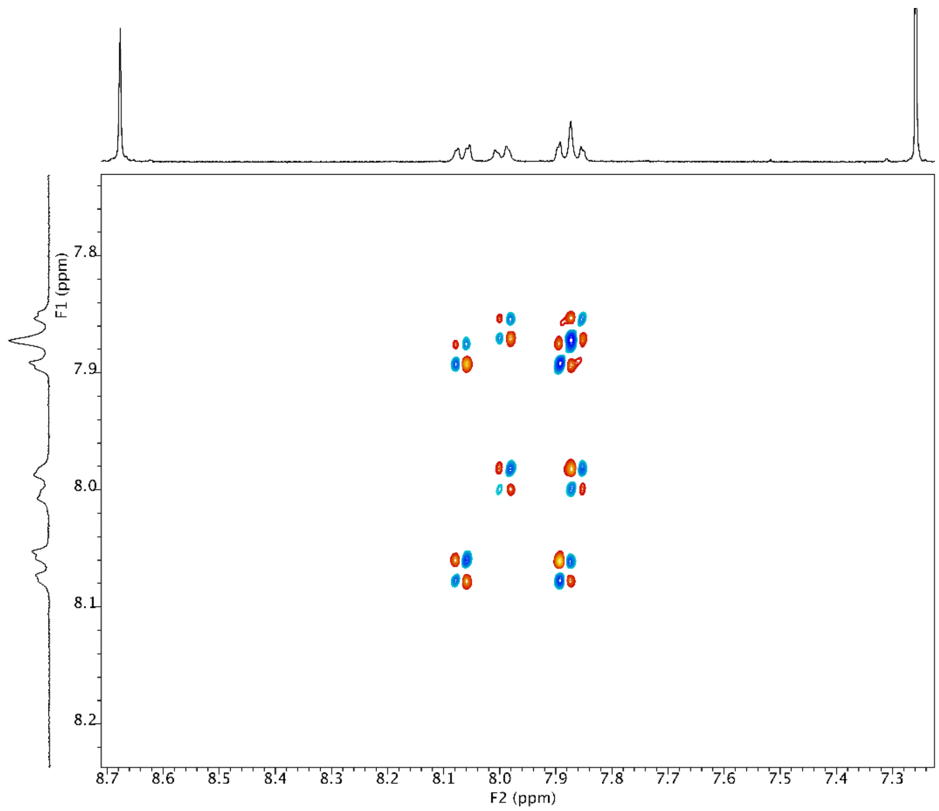

Figure S 4. ^{1}H-^{1}H gDQFCOSY spectrum of compound **RuP-Br** (400 MHz, $CDCl_3$).

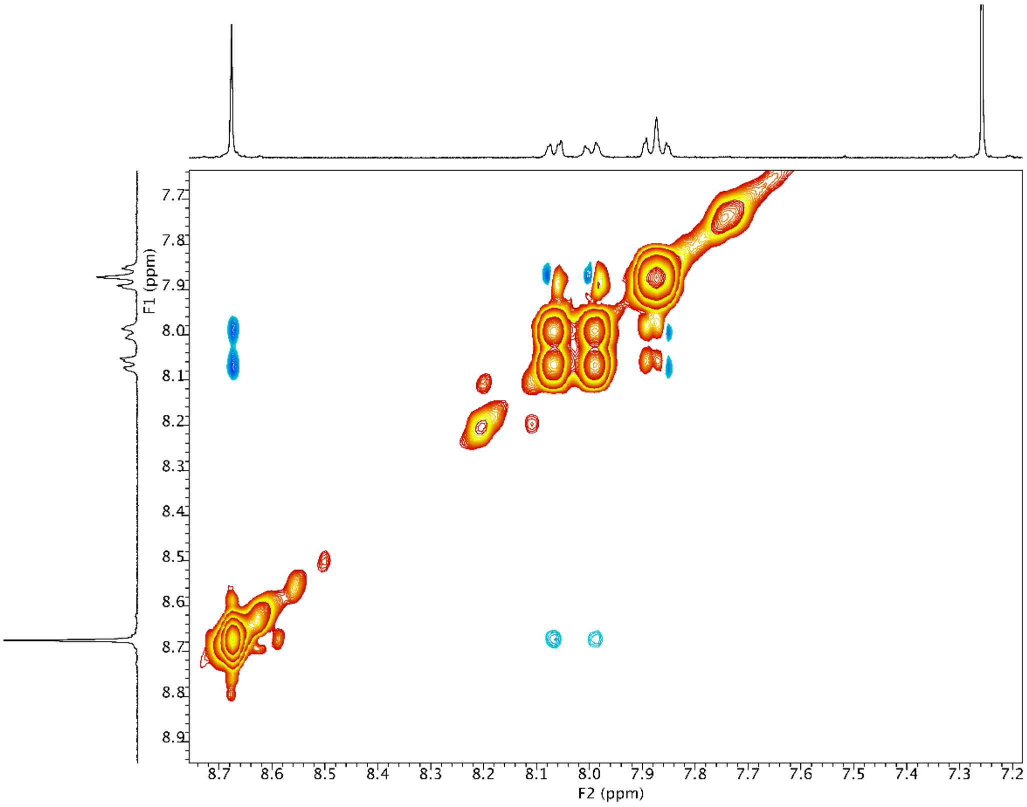

Figure S 5. ^{1}H-^{1}H NOESY spectrum of compound **RuP-Br** (500 MHz, $CDCl_3$).

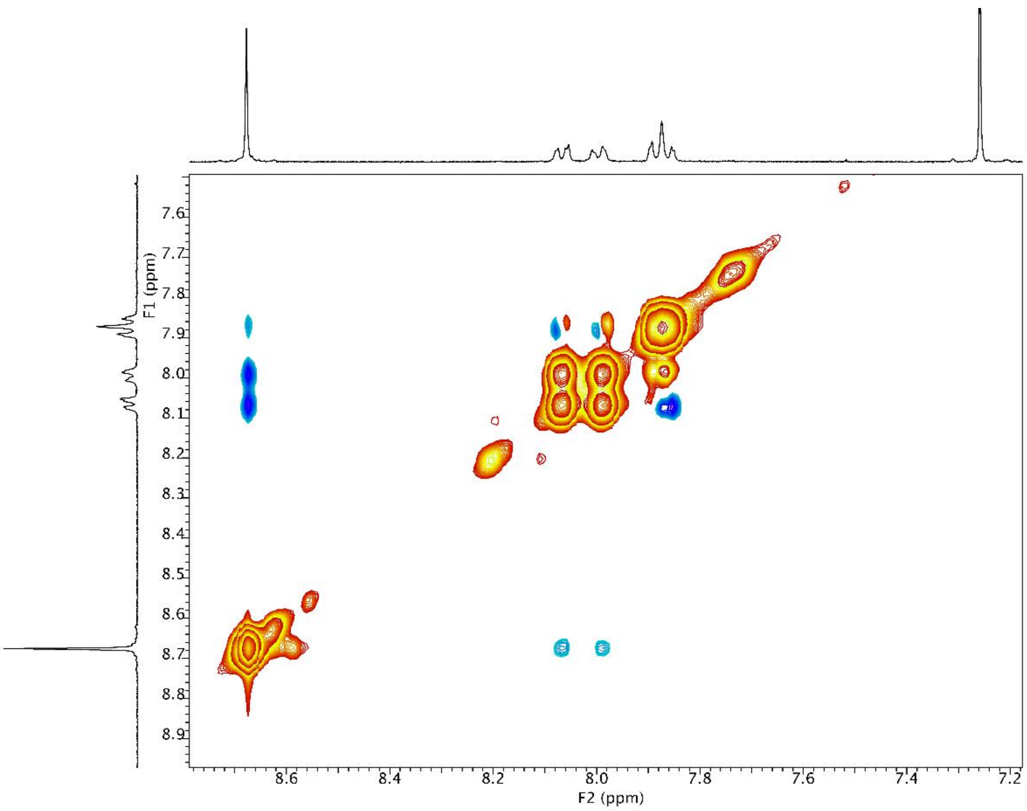

Figure S 6. ^{1}H-^{1}H ROESYAD spectrum of compound **RuP-Br** (500 MHz, $CDCl_3$).

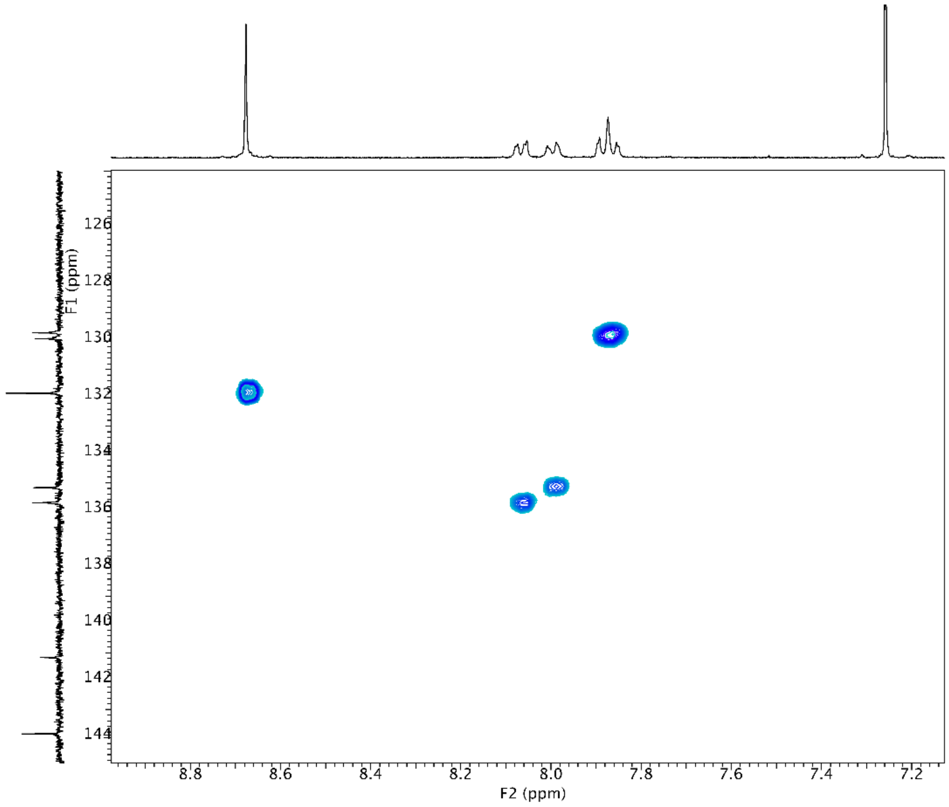

Figure S 7. ^{1}H-^{13}C gHSQCAD spectrum of compound **RuP-Br** (400 MHz, $CDCl_3$).

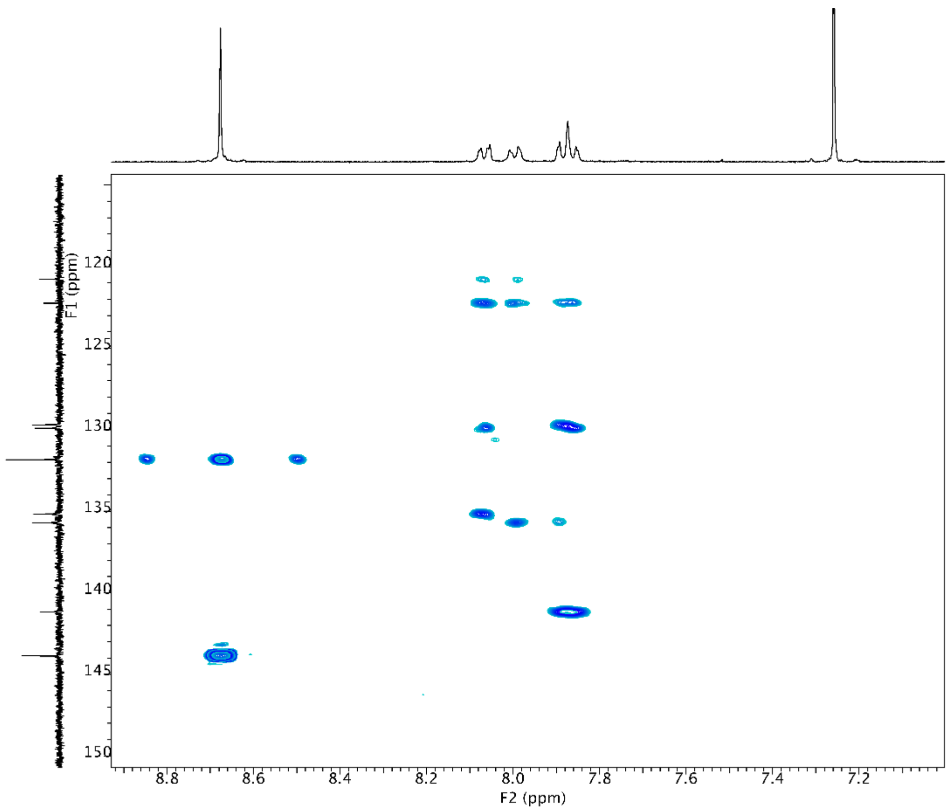

Figure S 8. ^{1}H-^{13}C gHMBCAD spectrum of compound **RuP-Br** (500 MHz, $CDCl_3$).

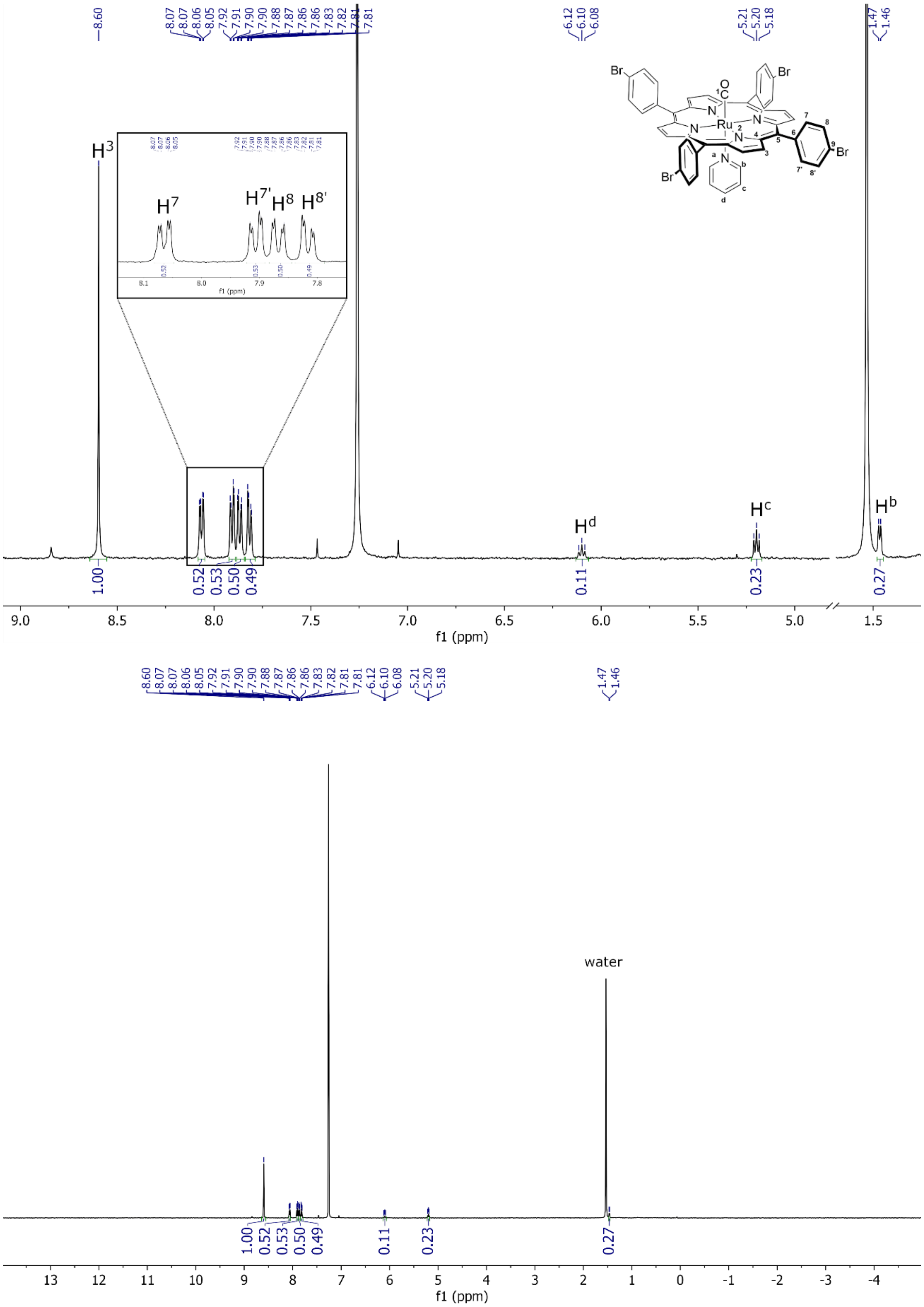

Figure S 9. ^{1}H NMR spectrum of compound **RuP-Br·py** (500 MHz, $CDCl_3$); selected regions (above) and full spectrum (below).

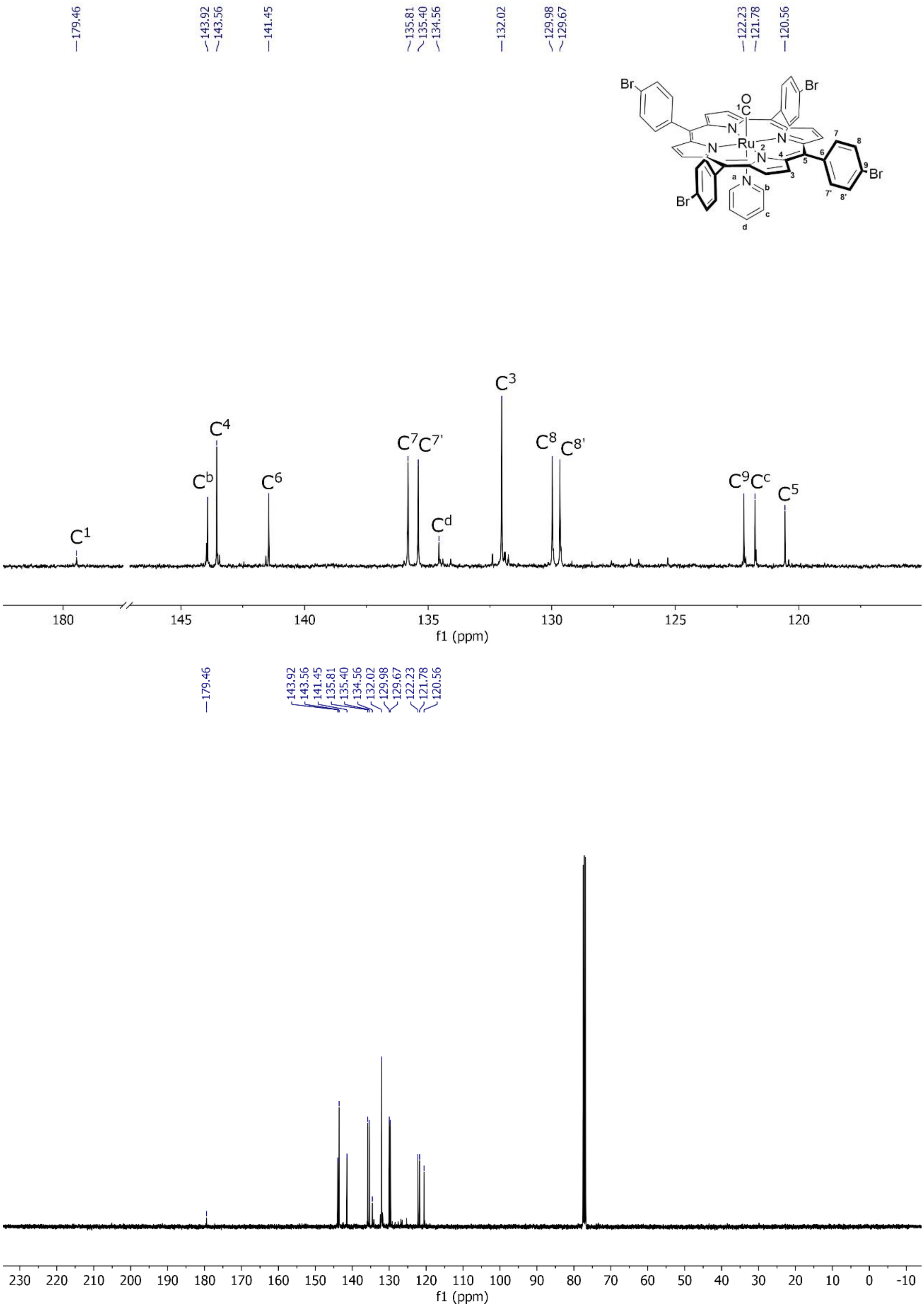

Figure S 10. $^{13}C\{^{1}H\}$ NMR spectrum of compound **RuP-Br·py** (101 MHz, $CDCl_3$); selected regions (above) and full spectrum (below).

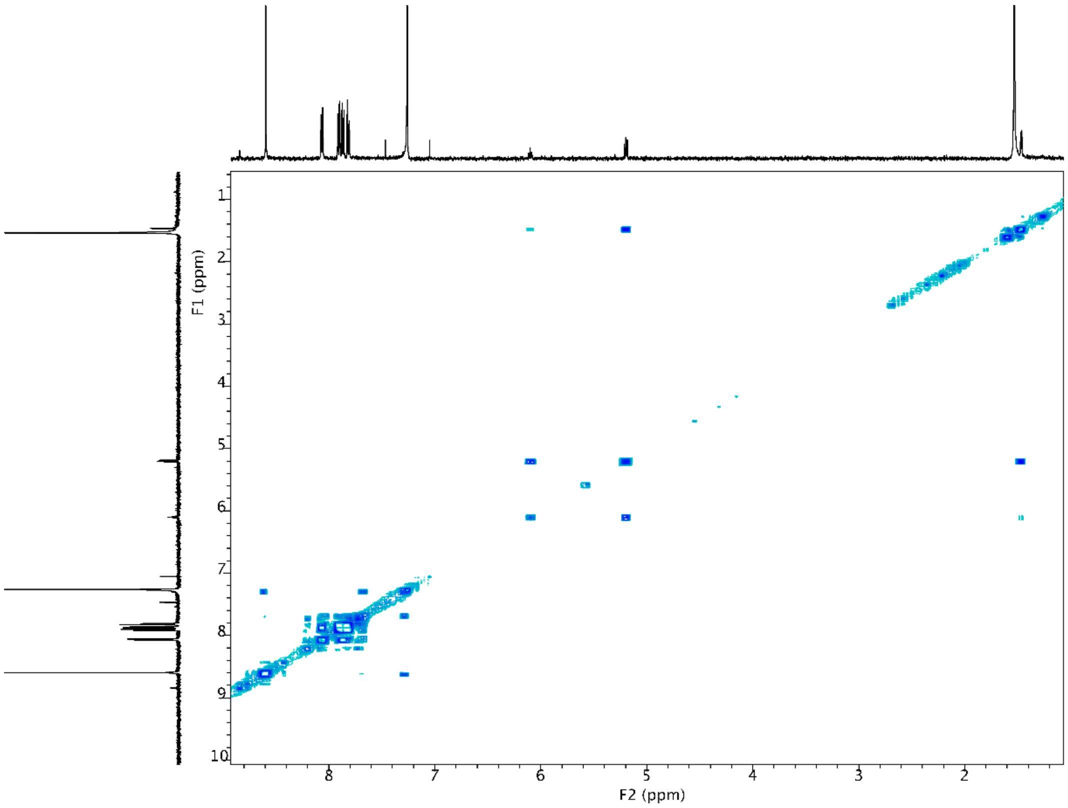

Figure S 11. ^{1}H-^{1}H gCOSY spectrum of compound **RuP-Br·py** (500 MHz, $CDCl_3$).

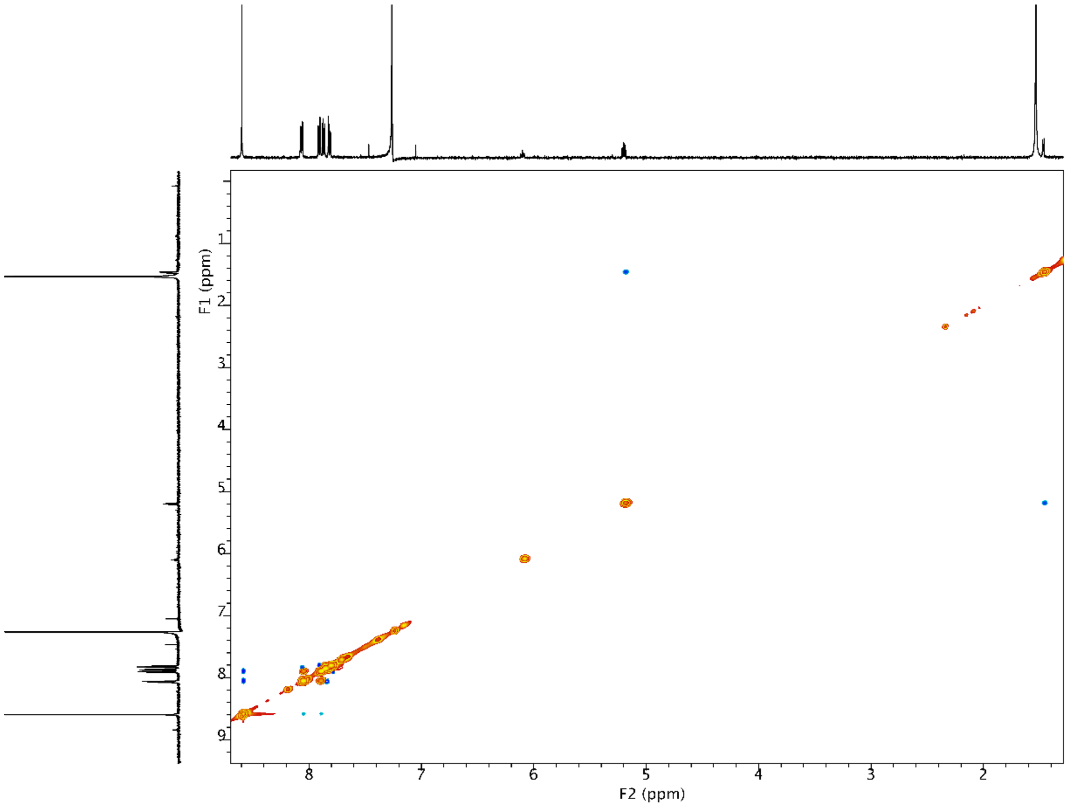

Figure S 12. ^{1}H-^{1}H NOESY spectrum of compound **RuP-Br·py** (400 MHz, $CDCl_3$).

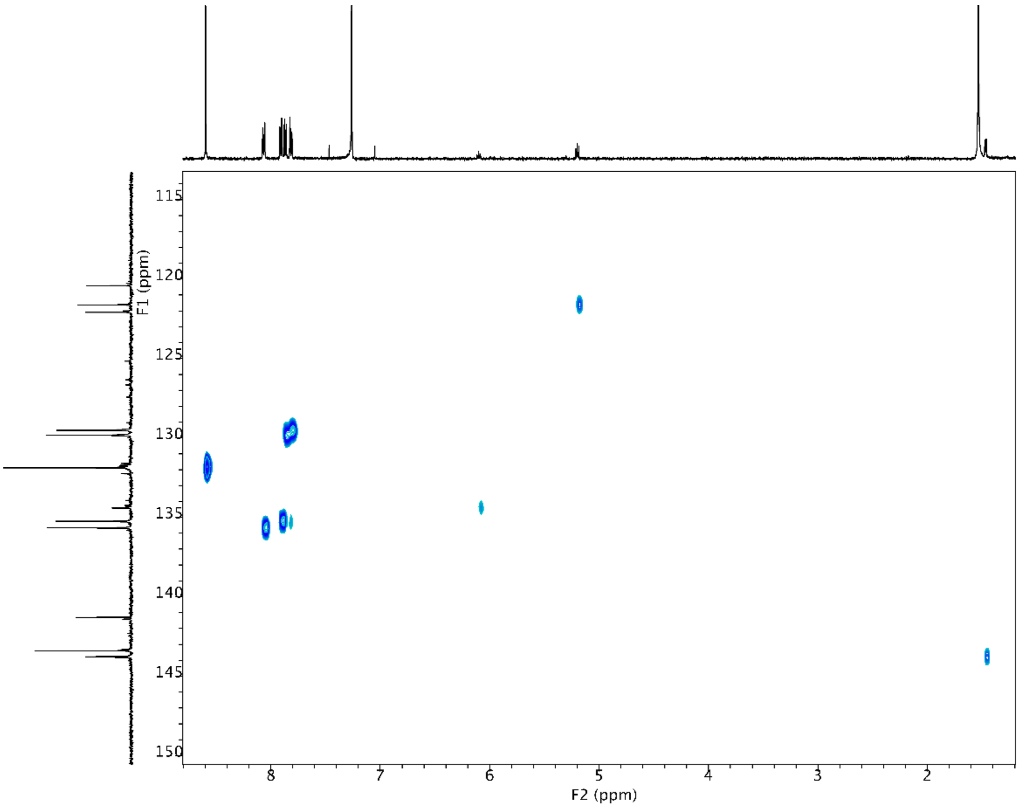

Figure S 13. ^{1}H-^{13}C gHSQCAD spectrum of compound **RuP-Br·py** (400 MHz, $CDCl_3$).

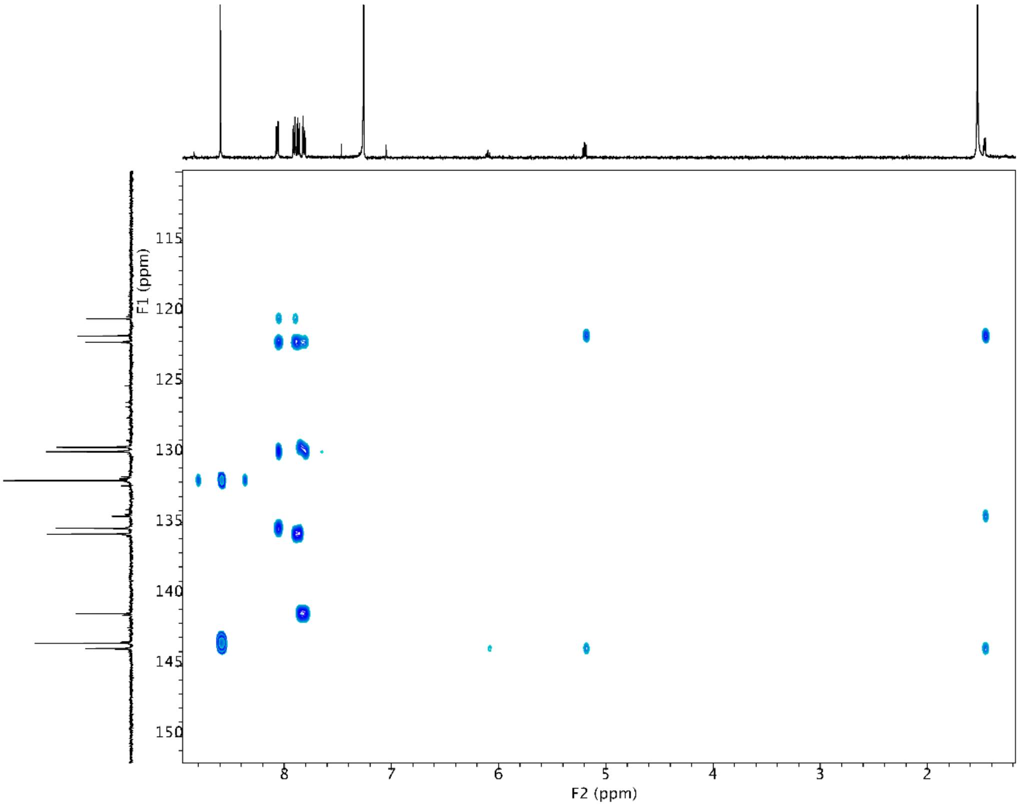

Figure S 14. ^{1}H-^{13}C gHMBCAD spectrum of compound **RuP-Br·py** (400 MHz, $CDCl_3$).

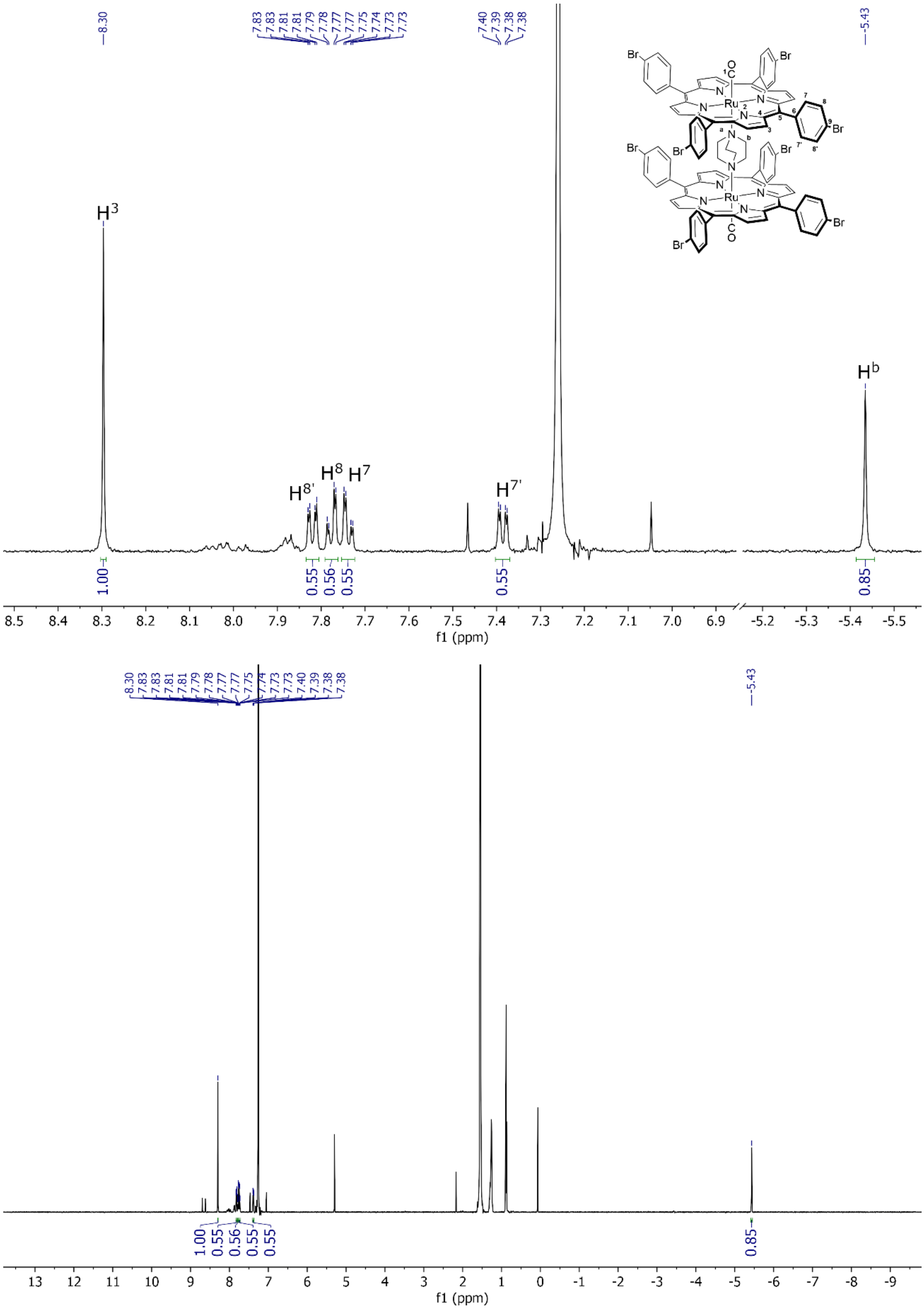

Figure S 15. ^{1}H NMR spectrum of compound **(RuP-Br)$_2$·DABCO** (500 MHz, $CDCl_3$); selected regions (above) and full spectrum (below).

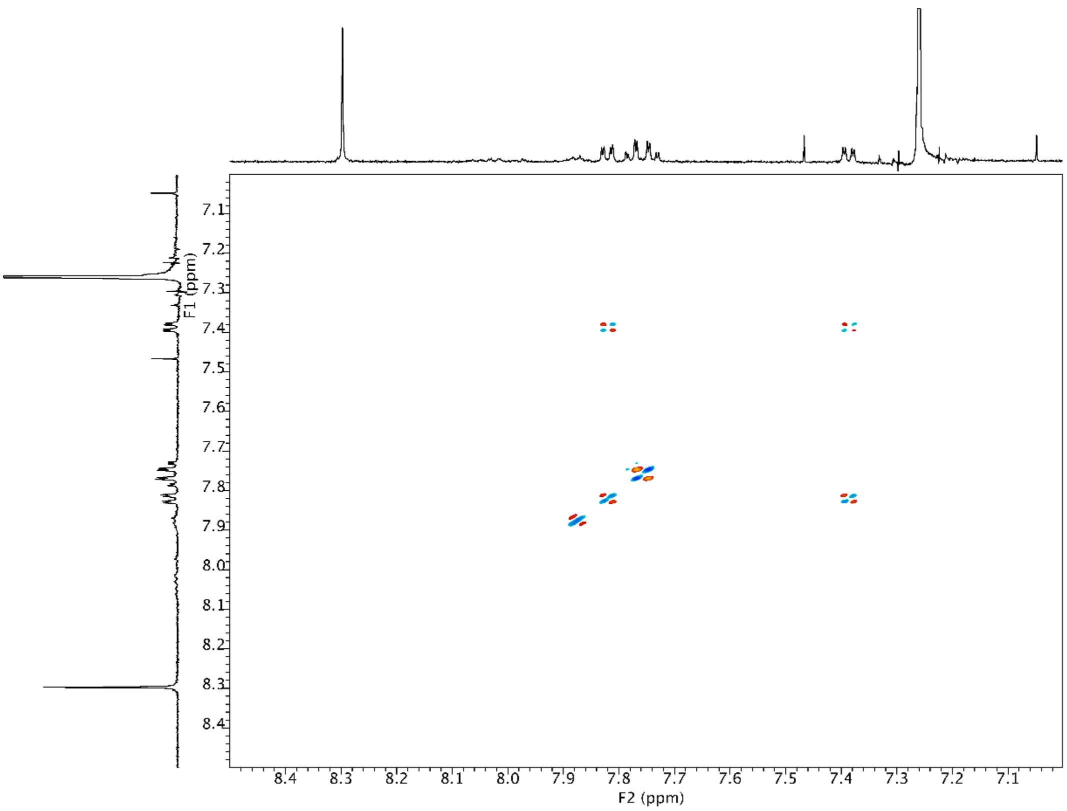

Figure S 16. ^1H-^1H gDQFCOSY spectrum of compound **(RuP-Br)$_2$·DABCO** (500 MHz, $CDCl_3$).

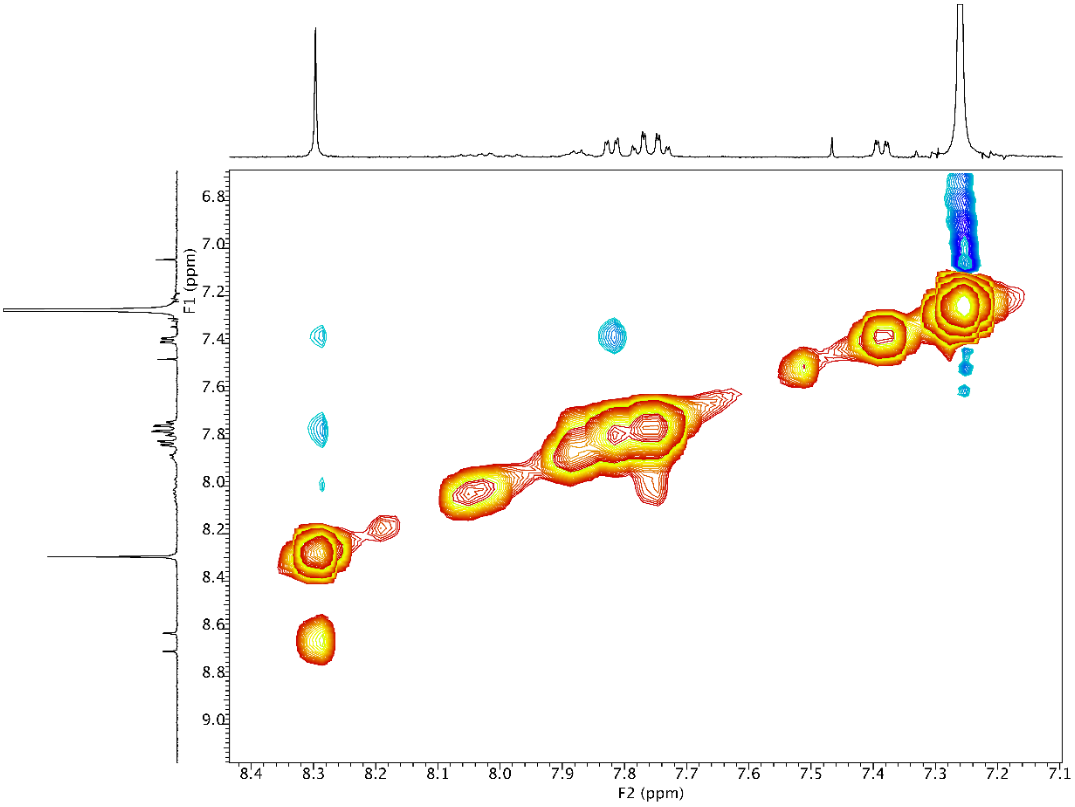

Figure S 17. ^1H-^1H ROESYAD spectrum of compound **(RuP-Br)$_2$·DABCO** (400 MHz, $CDCl_3$).

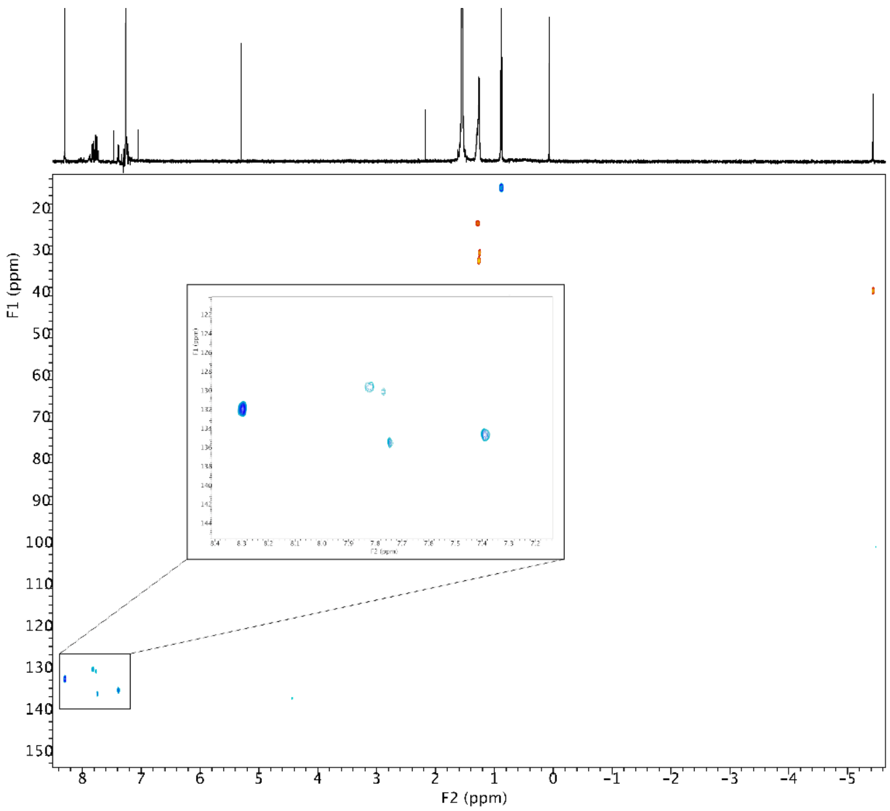

Figure S 18. ^{1}H-^{13}C gc2hsqc spectrum of compound **(RuP-Br)$_2$·DABCO** (500 MHz, $CDCl_3$).

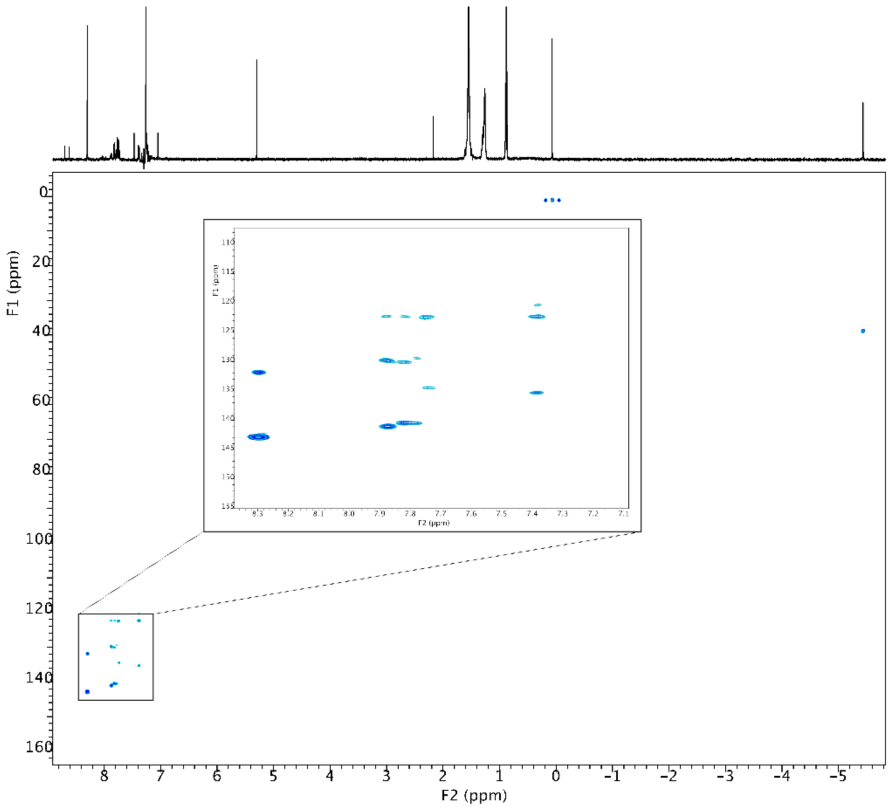

Figure S 19. ^{1}H-^{13}C gc2hmbc spectrum of compound **(RuP-Br)$_2$·DABCO** (500 MHz, $CDCl_3$).

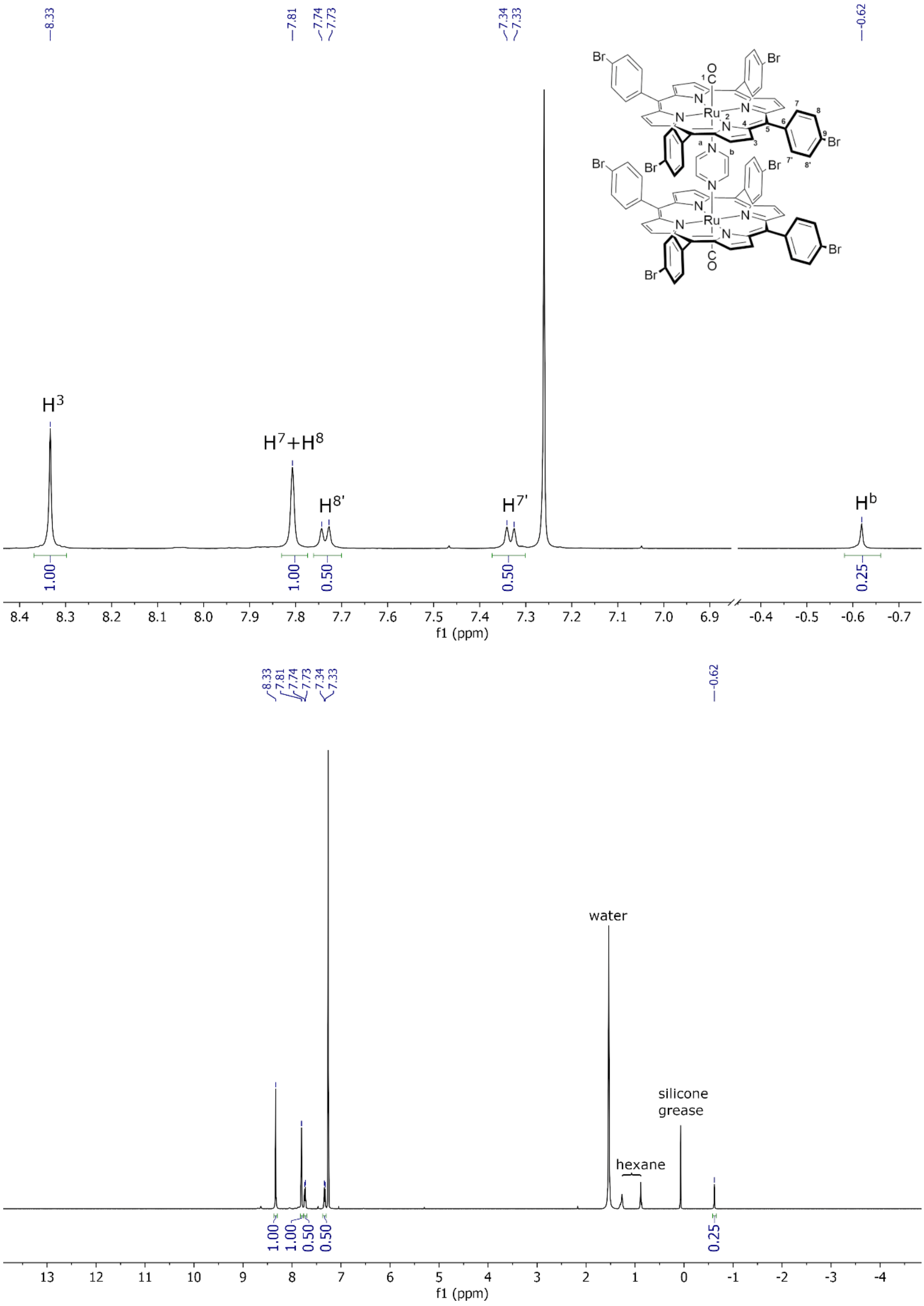

Figure S 20. ^{1}H NMR spectrum of compound **(RuP-Br)$_2$·daz** (500 MHz, $CDCl_3$); selected regions (above) and full spectrum (below).

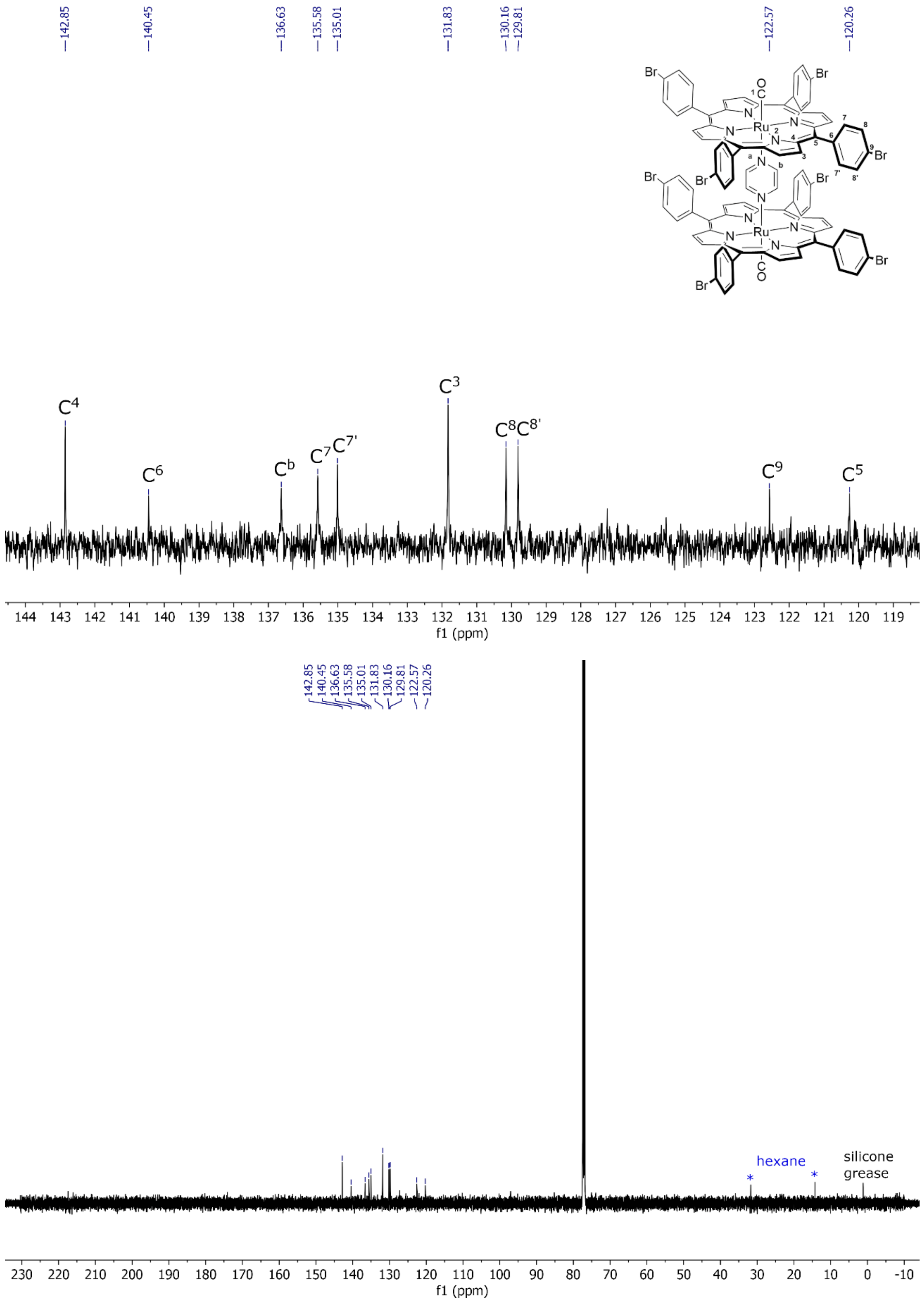

Figure S 21. $^{13}C\{^{1}H\}$ NMR spectrum of compound **(RuP-Br)₂·daz** (126 MHz, $CDCl_3$); selected regions (above) and full spectrum (below).

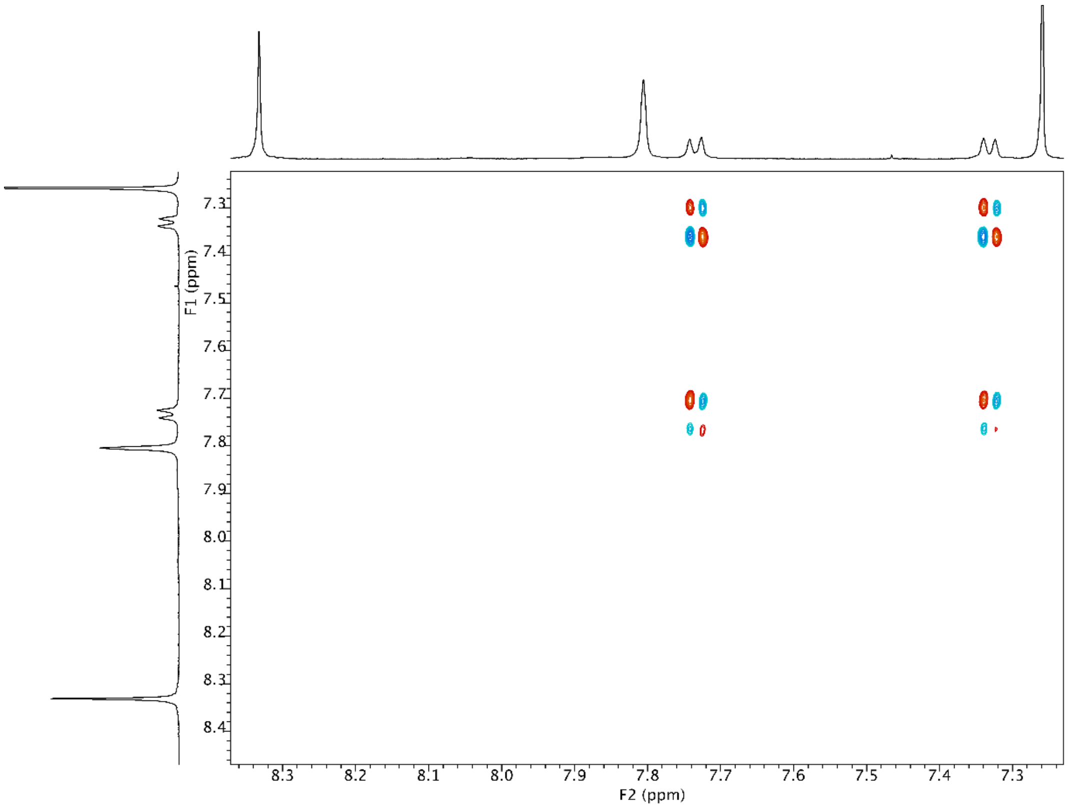

Figure S 22. ^{1}H-^{1}H gDQFCOSY spectrum of compound **(RuP-Br)$_2$·daz** (500 MHz, $CDCl_3$).

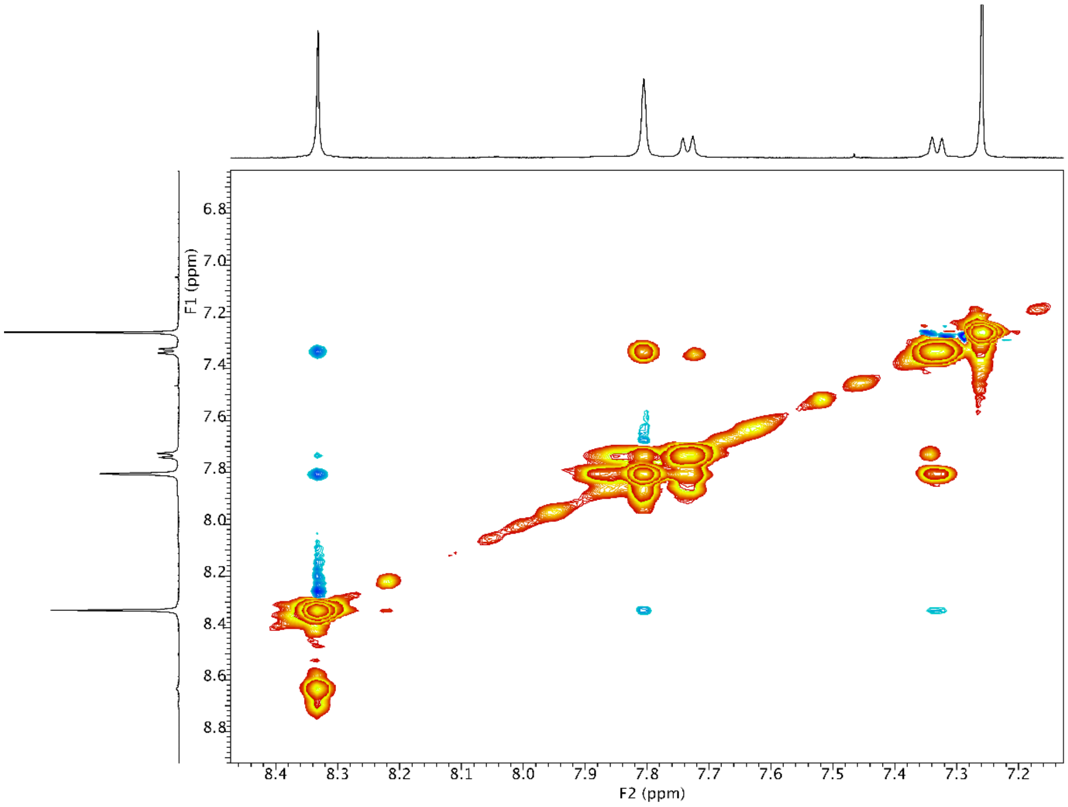

Figure S 23. ^{1}H-^{1}H NOESY spectrum of compound **(RuP-Br)$_2$·daz** (400 MHz, $CDCl_3$).

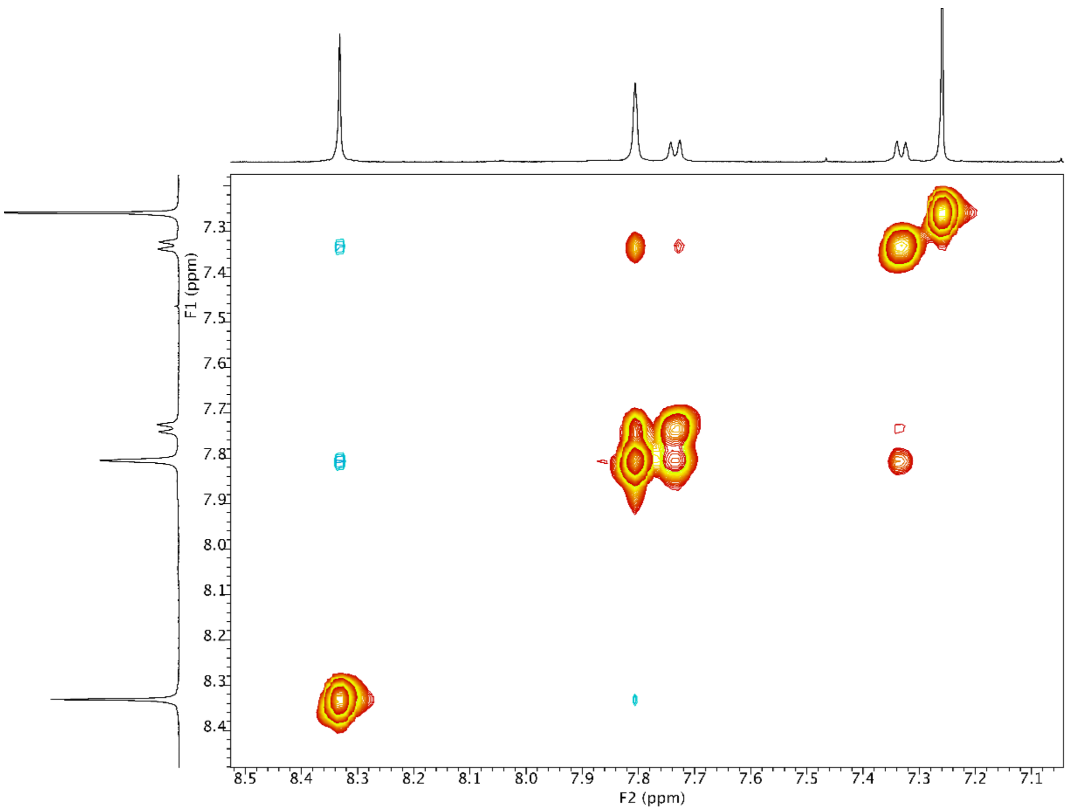

Figure S 24. ^{1}H-^{1}H ROESYAD spectrum of compound **(RuP-Br)$_2$·daz** (400 MHz, $CDCl_3$).

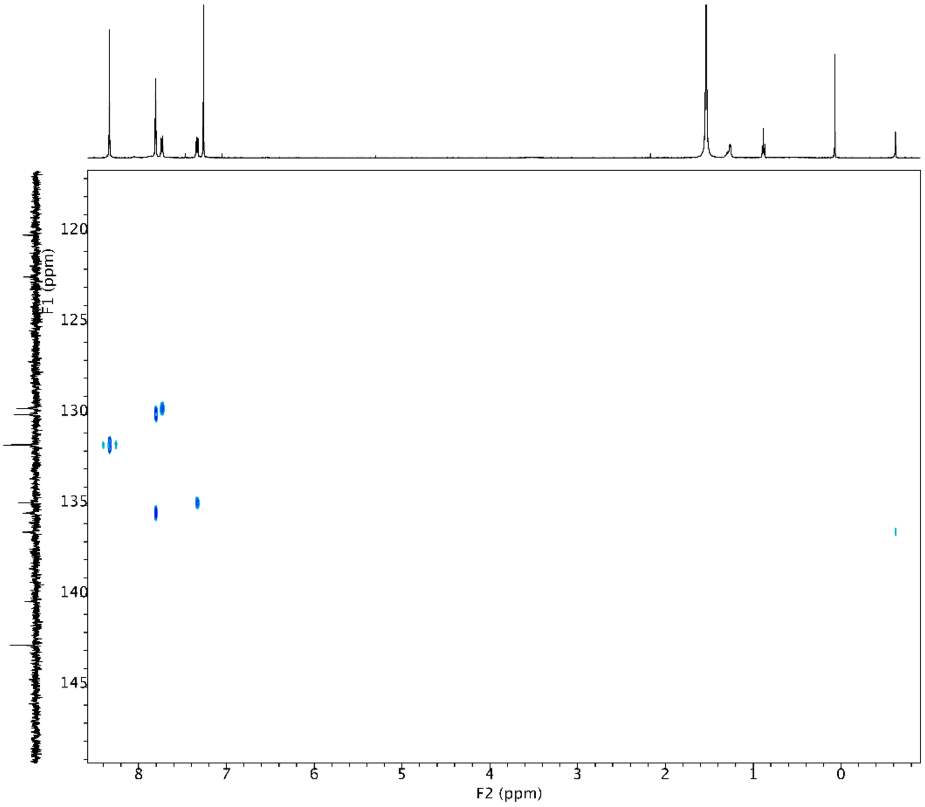

Figure S 25. ^{1}H-^{13}C gHSQCAD spectrum of compound **(RuP-Br)$_2$·daz** (500 MHz, $CDCl_3$).

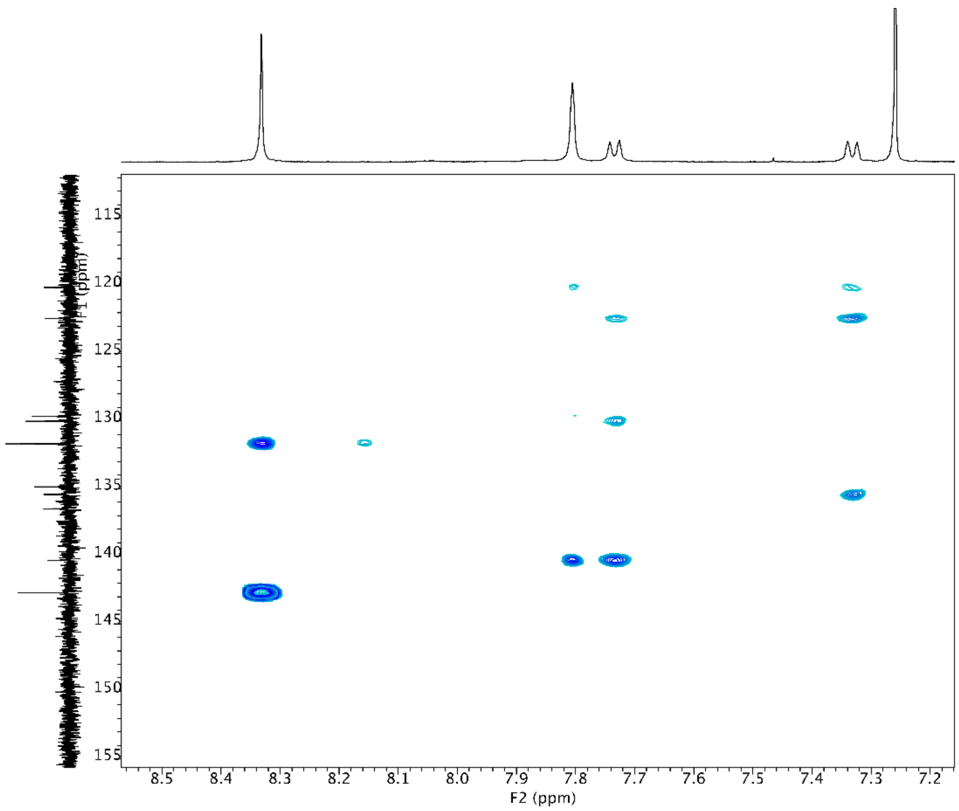

Figure S 26. ^{1}H-^{13}C gHMBCAD spectrum of compound **(RuP-Br)$_2$·daz** (500 MHz, $CDCl_3$).

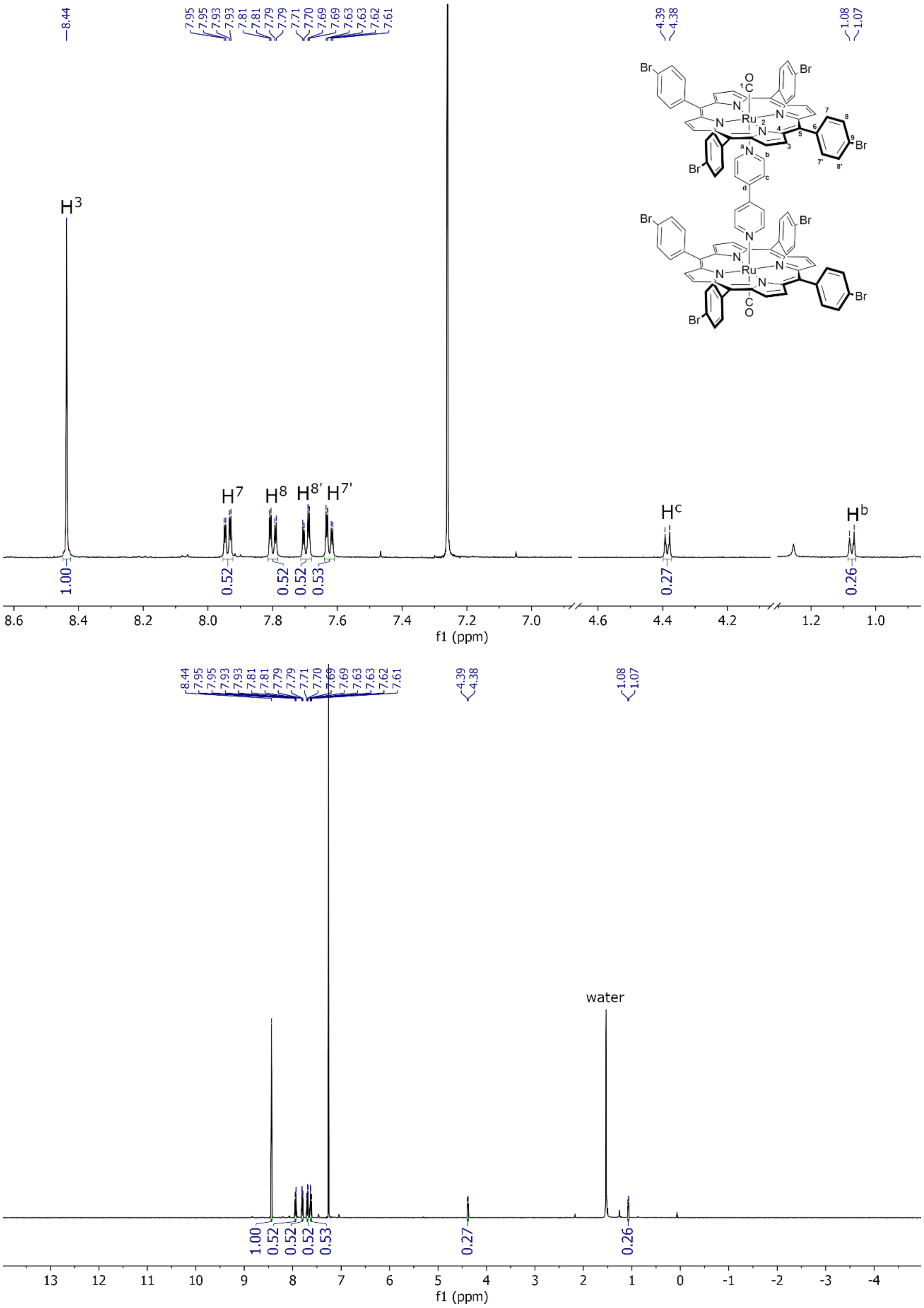

Figure S 27. ^{1}H NMR spectrum of compound **(RuP-Br)$_2$·bpy** (500 MHz, $CDCl_3$); selected regions (above) and full spectrum (below).

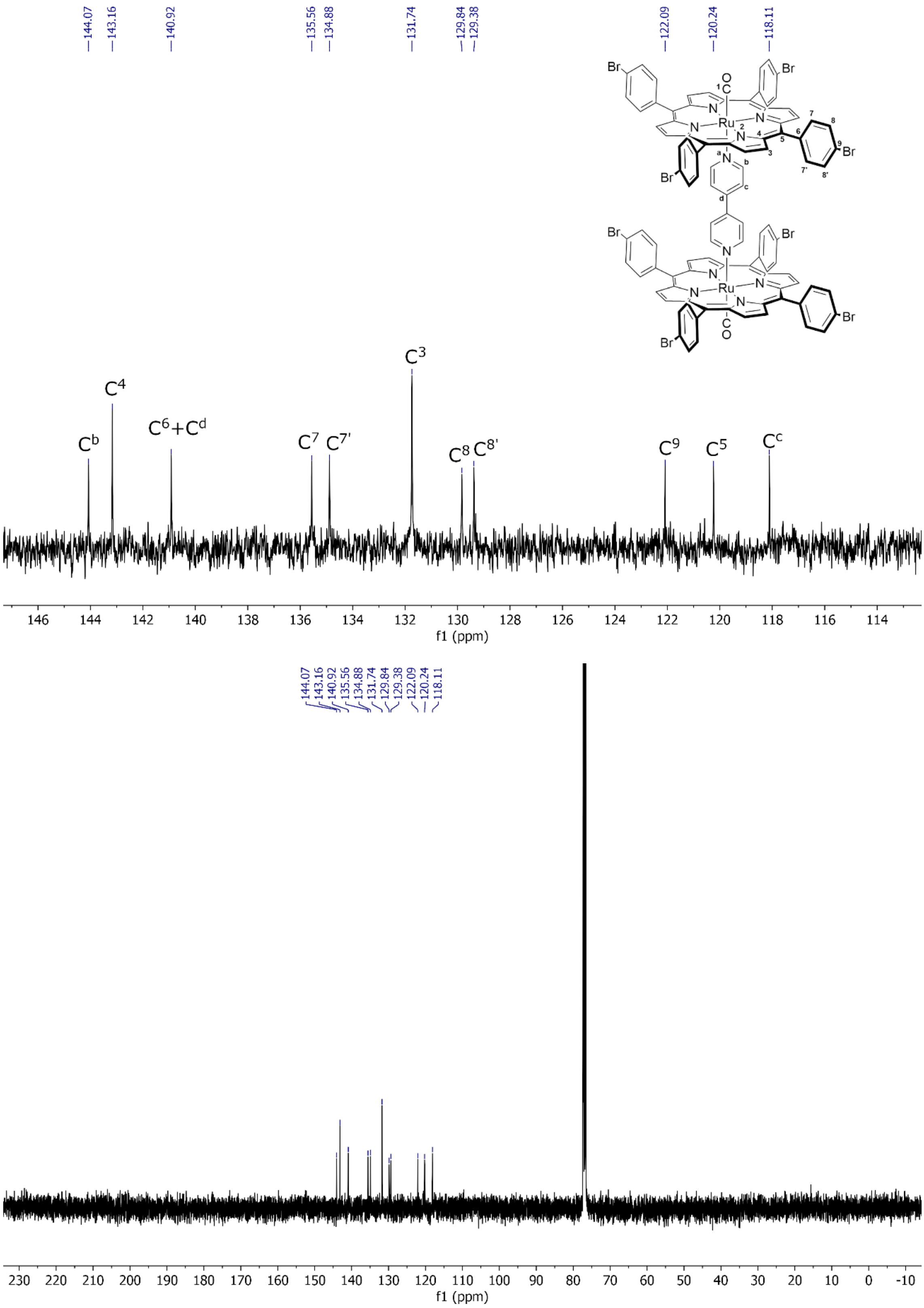

Figure S 28. $^{13}C\{^{1}H\}$ NMR spectrum of compound **(RuP-Br)$_2$·bpy** (101 MHz, $CDCl_3$); selected regions (above) and full spectrum (below).

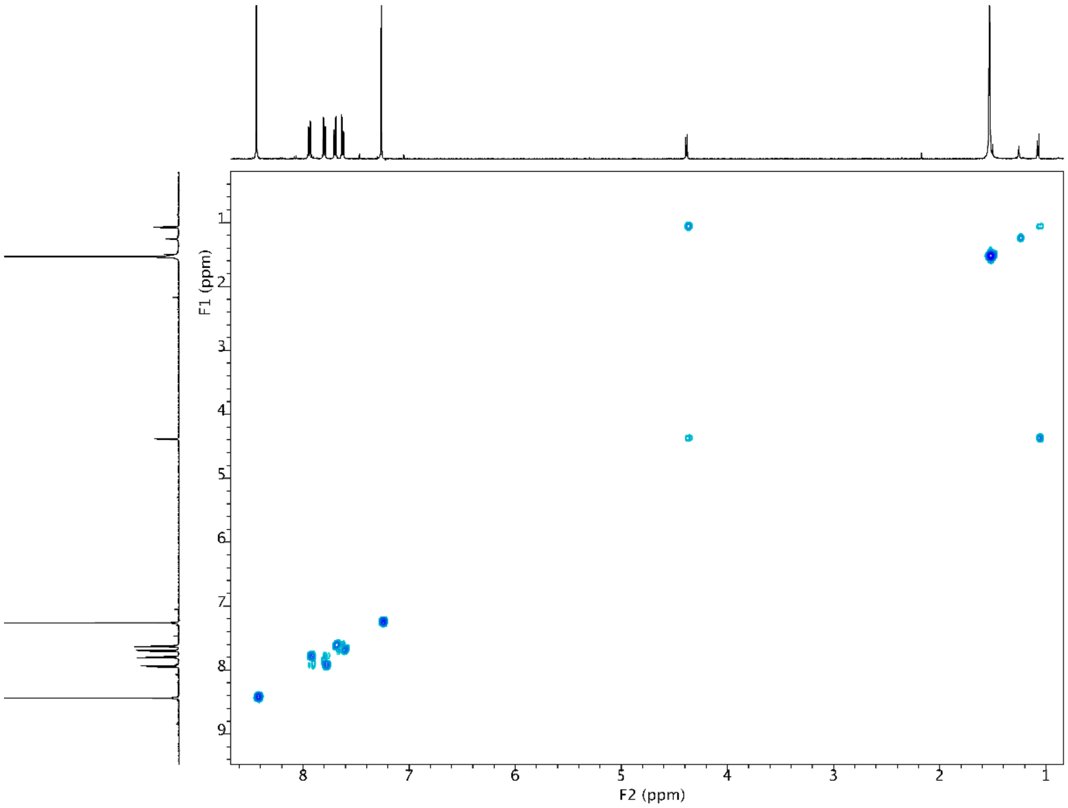

Figure S 29. ^{1}H-^{1}H gCOSY spectrum of compound **(RuP-Br)$_2$·bpy** (500 MHz, $CDCl_3$).

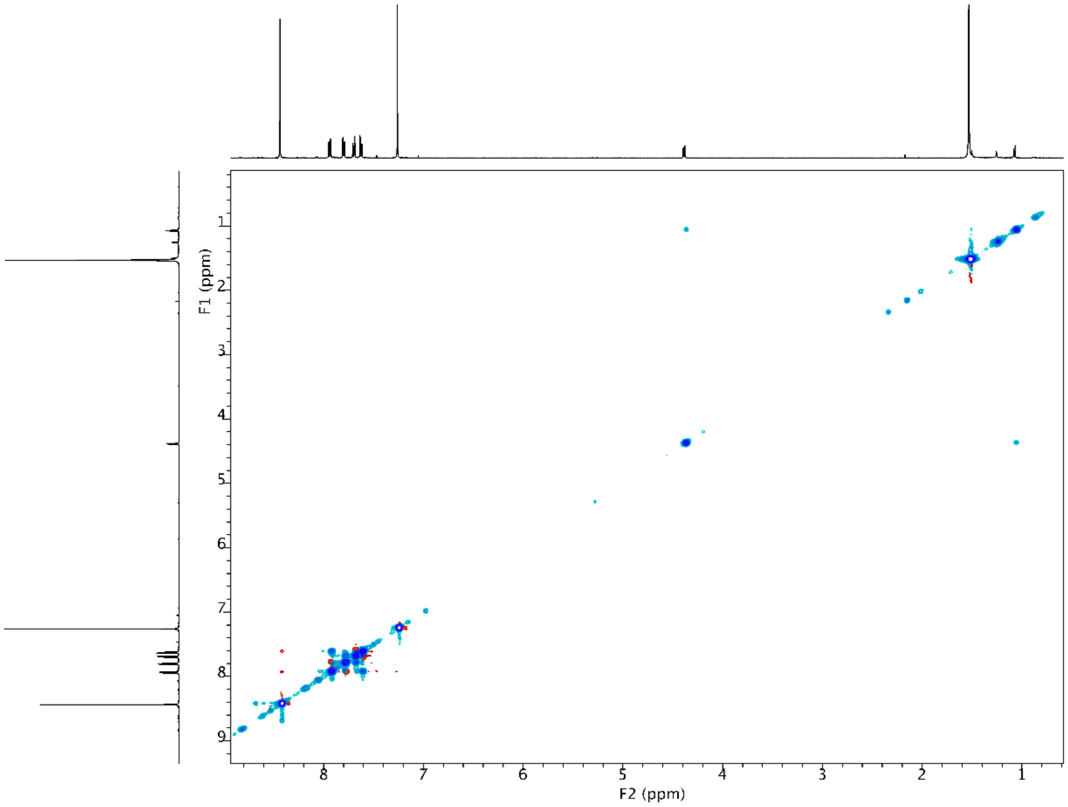

Figure S 30. ^{1}H-^{1}H NOESY spectrum of compound **(RuP-Br)$_2$·bpy** (400 MHz, $CDCl_3$).

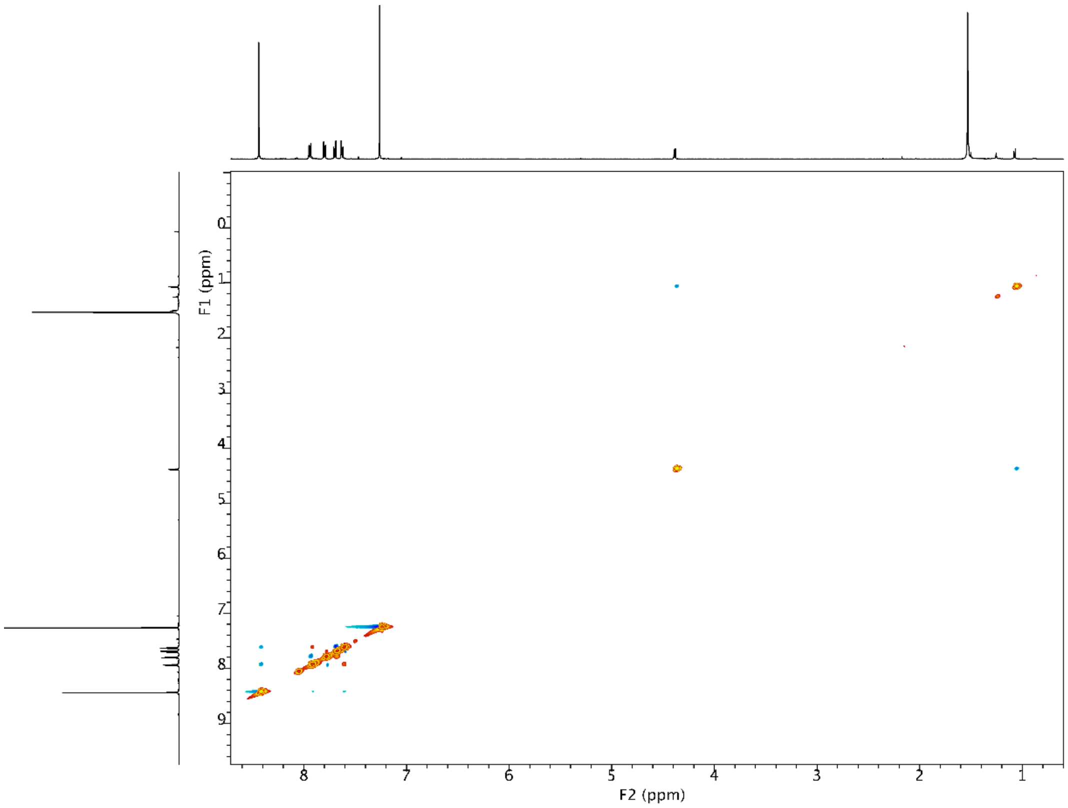

Figure S 31. ^{1}H-^{1}H ROESYAD spectrum of compound **(RuP-Br)$_2$·bpy** (400 MHz, $CDCl_3$).

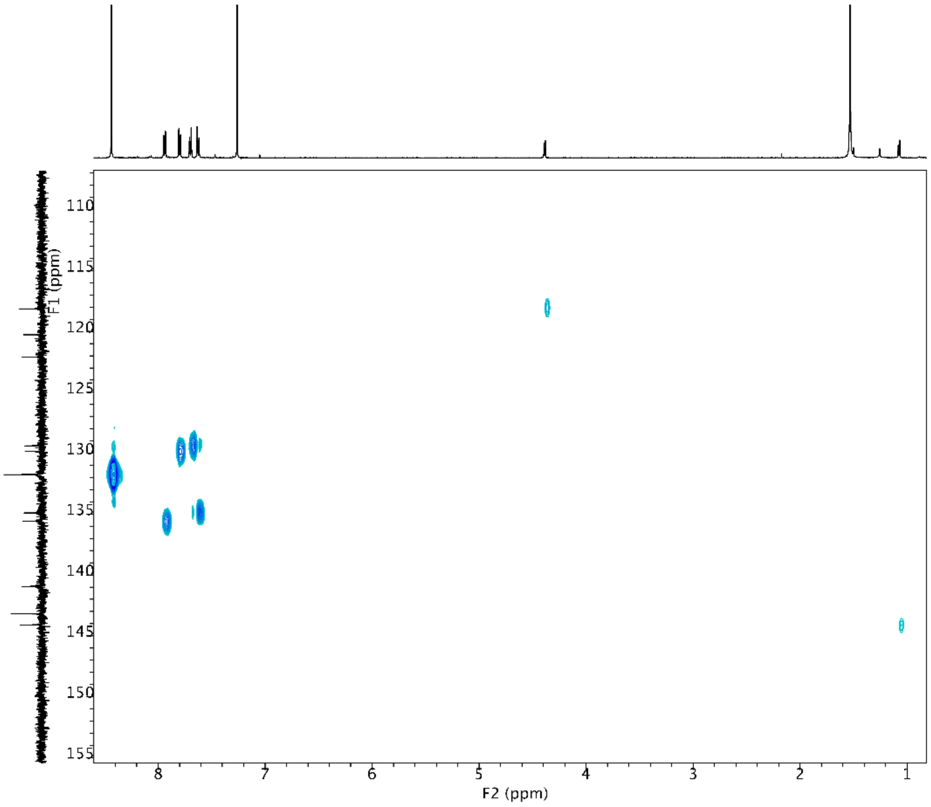

Figure S 32. ^{1}H-^{13}C gHSQCAD spectrum of compound **(RuP-Br)$_2$·bpy** (400 MHz, $CDCl_3$).

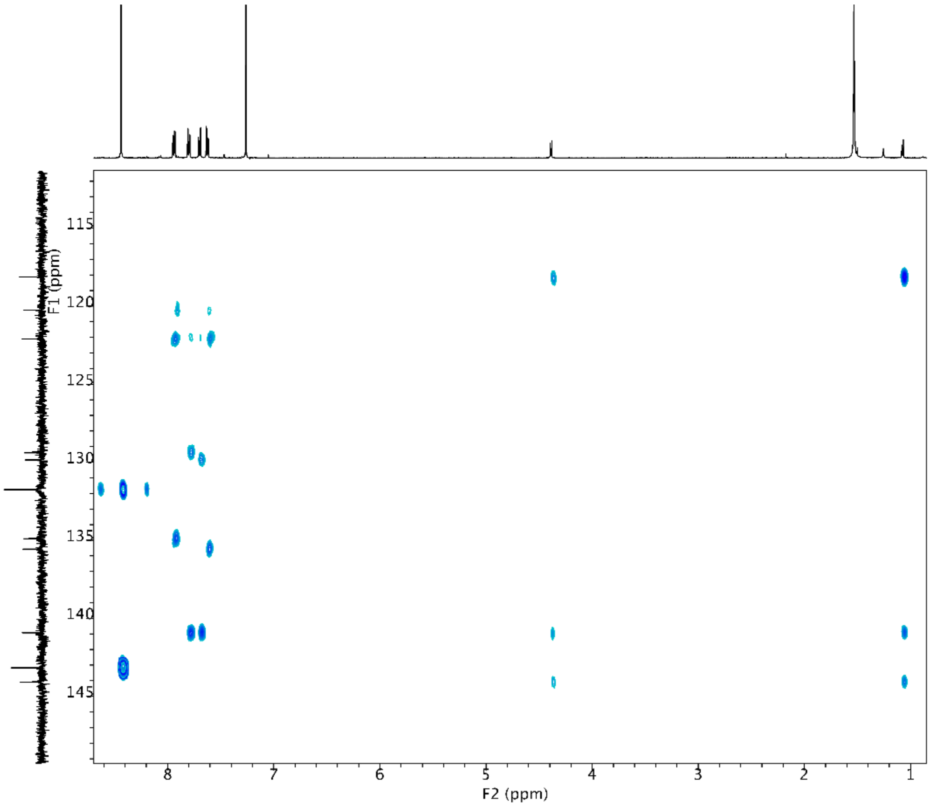

Figure S 33. ^{1}H-^{13}C gHMBCAD spectrum of compound **(RuP-Br)$_2$·bpy** (400 MHz, $CDCl_3$).

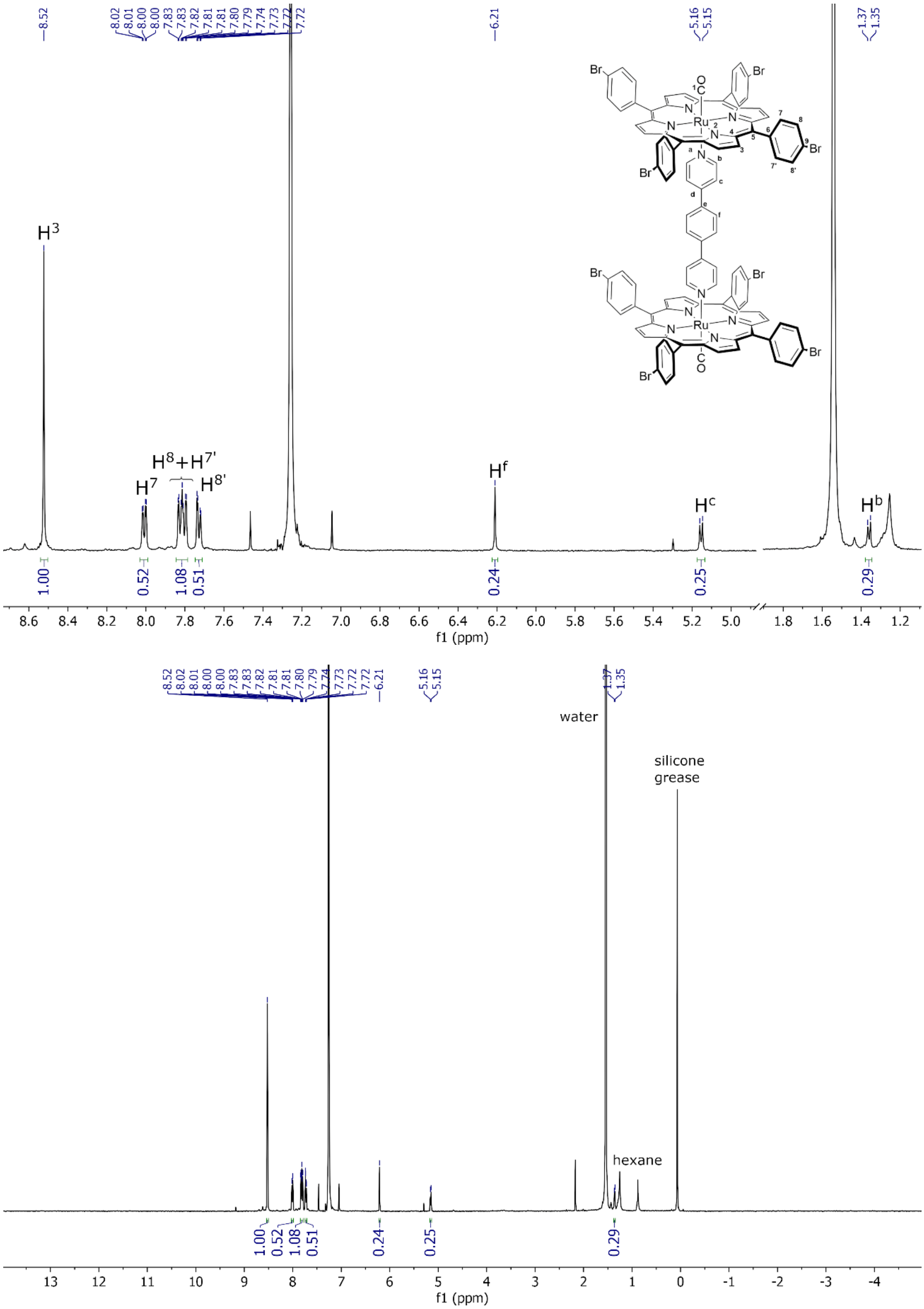

Figure S 34. ^1H NMR spectrum of compound **(RuP-Br)$_2$·dpyb** (500 MHz, $CDCl_3$); selected regions (above) and full spectrum (below).

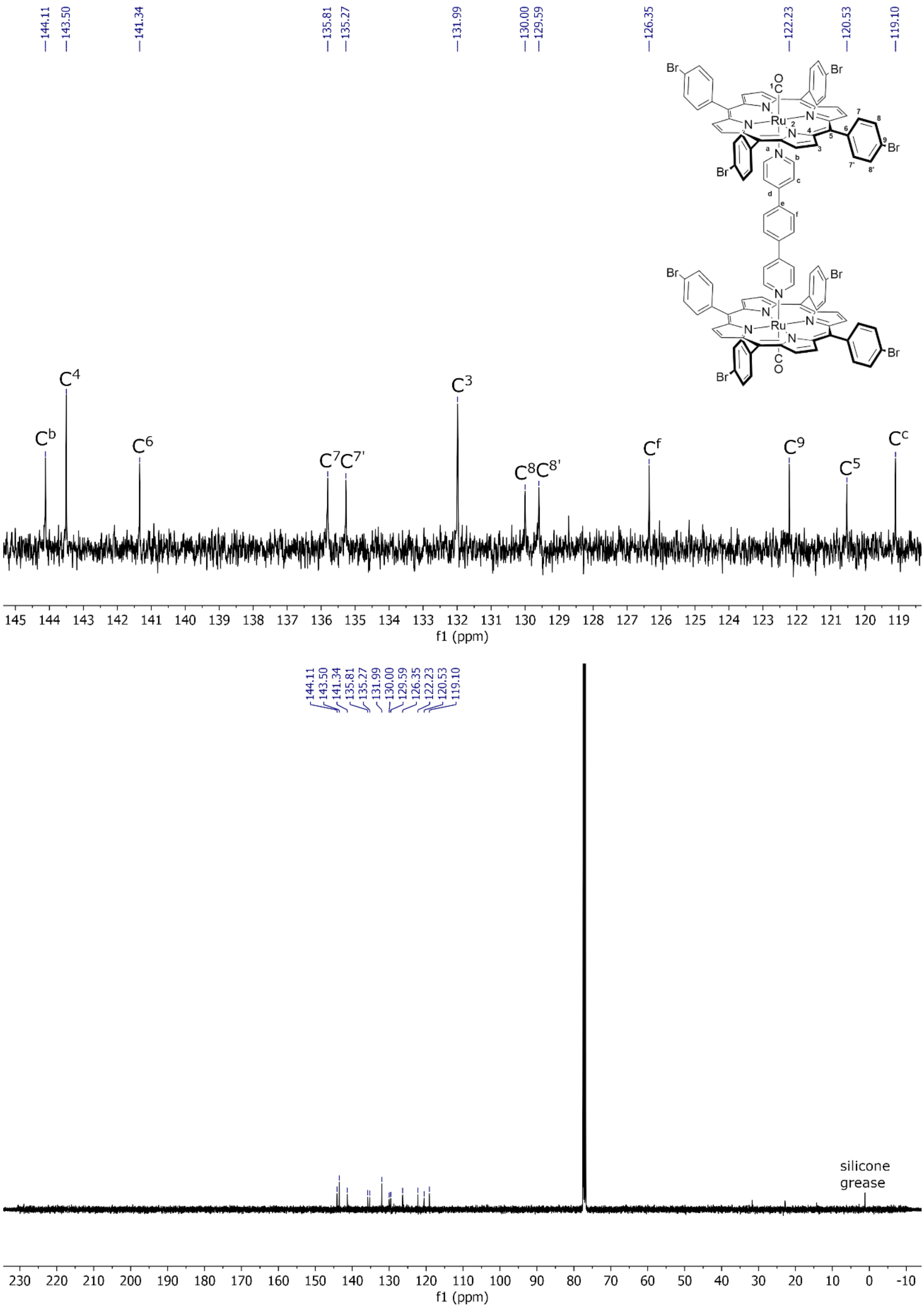

Figure S 35. $^{13}C\{^1H\}$ NMR spectrum of compound **(RuP-Br)$_2$·dpyb** (101 MHz, $CDCl_3$); selected regions (above) and full spectrum (below).

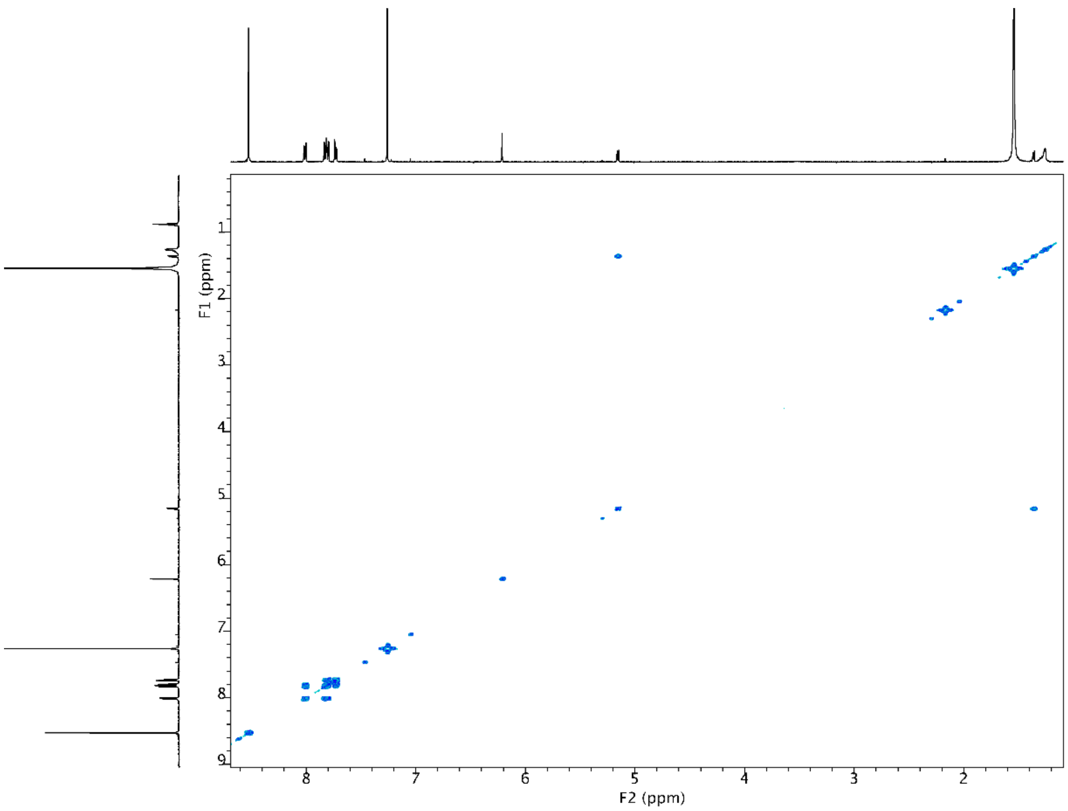

Figure S 36. ^{1}H-^{1}H gCOSY spectrum of compound **(RuP-Br)$_2$·dpyb** (500 MHz, $CDCl_3$).

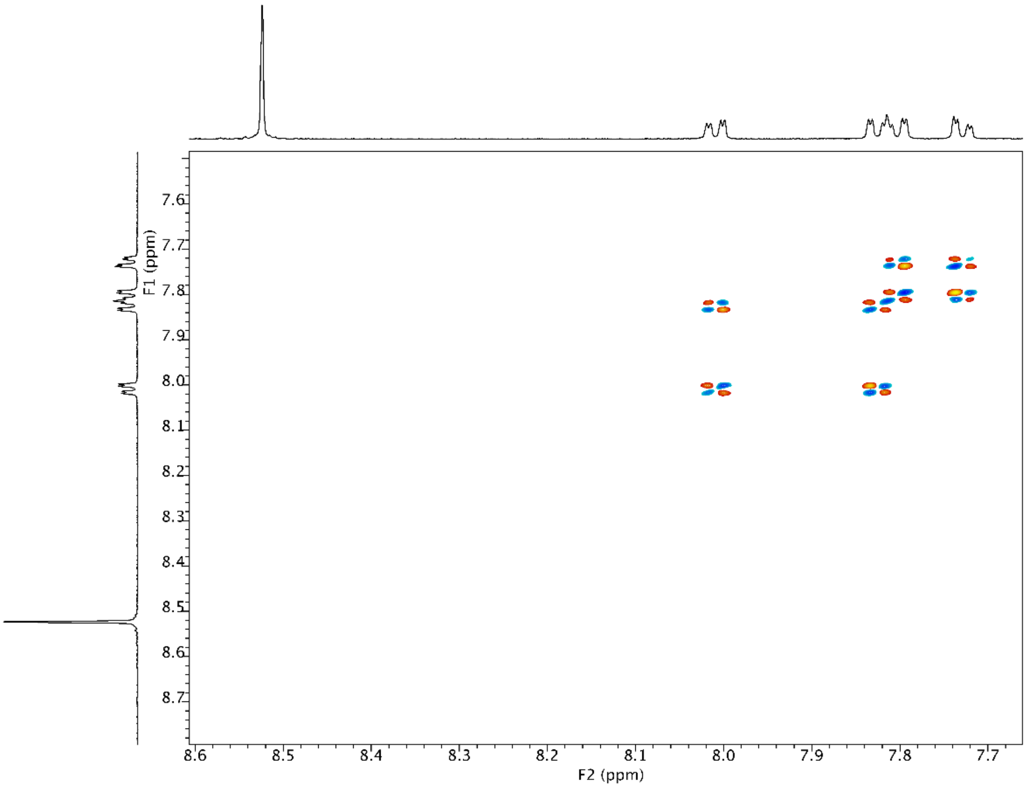

Figure S 37. ^{1}H-^{1}H gDQFCOSY spectrum of compound **(RuP-Br)$_2$·dpyb** (500 MHz, $CDCl_3$).

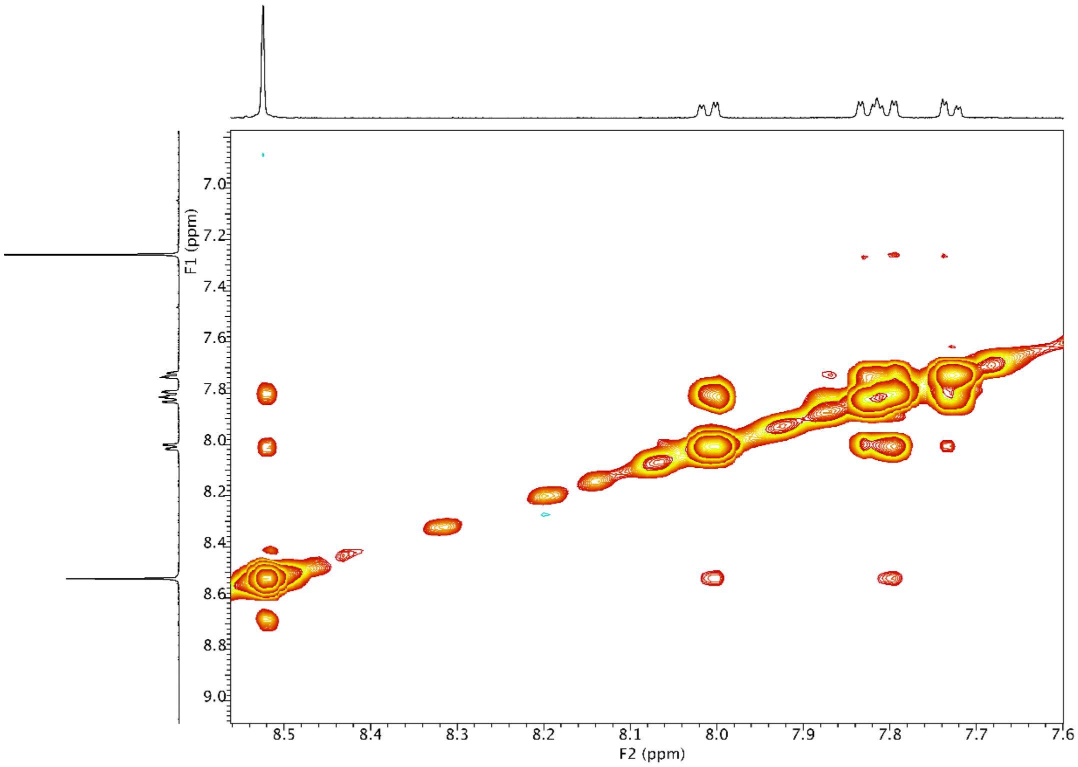

Figure S 38. ^{1}H-^{1}H NOESY spectrum of compound **(RuP-Br)$_2$·dpyb** (500 MHz, $CDCl_3$).

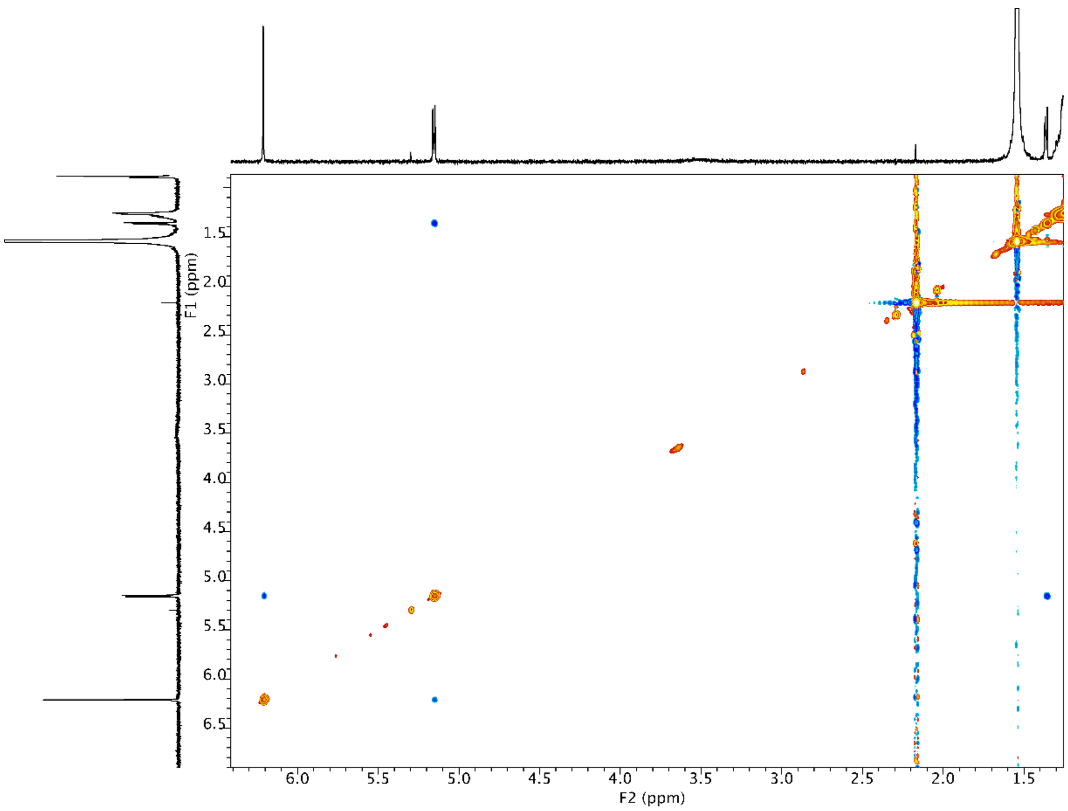

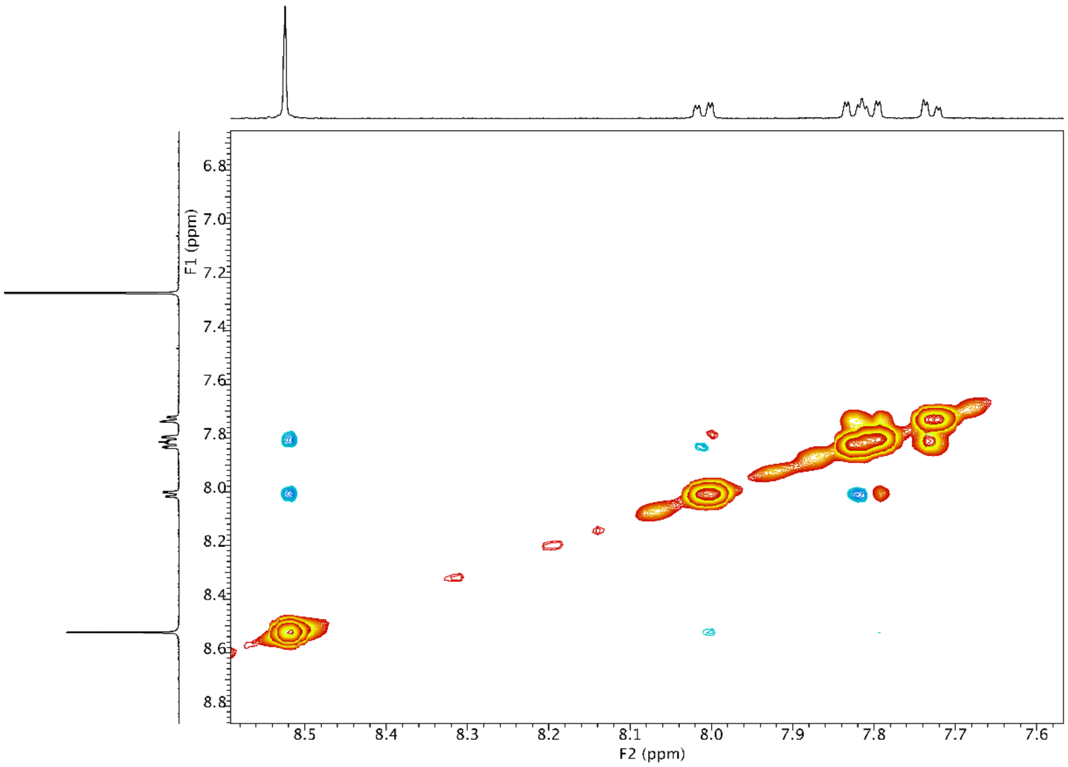

Figure S 39. ^{1}H-^{1}H ROESYAD selected regions of the spectrum of the compound **(RuP-Br)$_2$·dpyb** (500 MHz, $CDCl_3$); aliphatic region (above) and aromatic region (below).

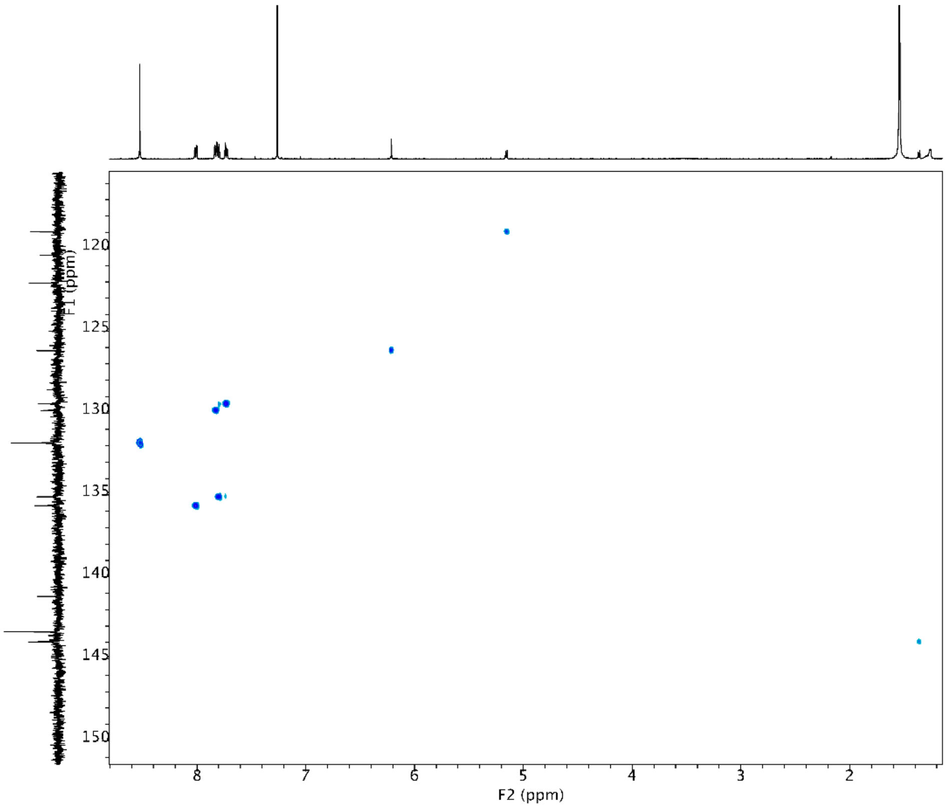

Figure S 40. ^{1}H-^{13}C gHSQCAD spectrum of compound **(RuP-Br)$_2$·dpyb** (400 MHz, $CDCl_3$).

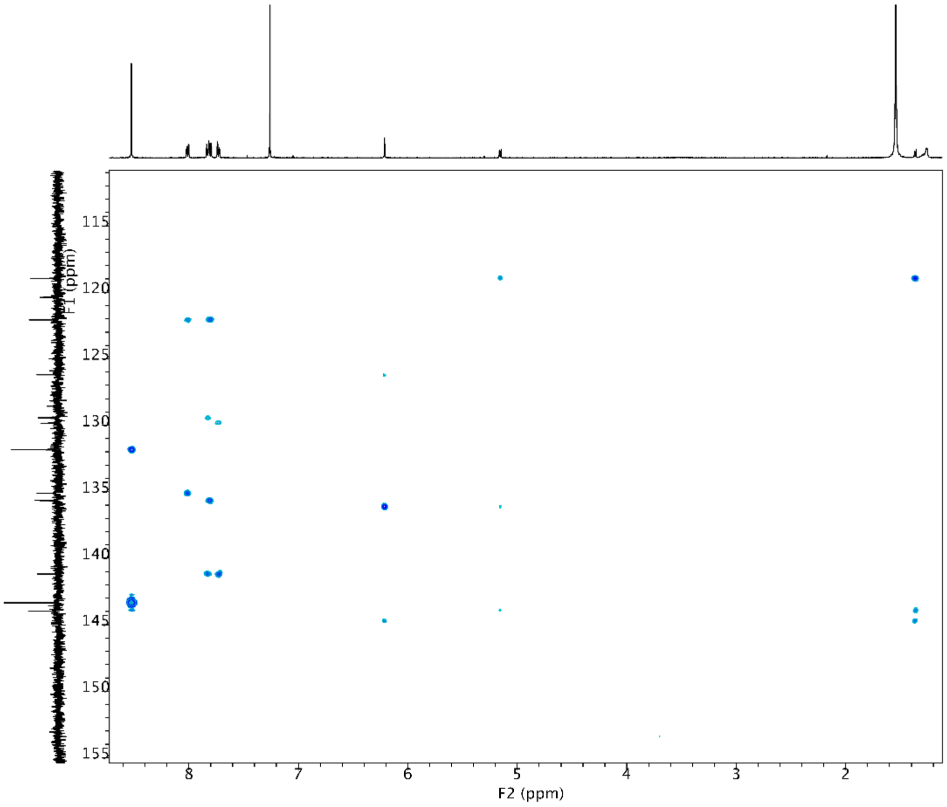

Figure S 41. ^{1}H-^{13}C gHMBCAD spectrum of compound **(RuP-Br)$_2$·dpyb** (400 MHz, $CDCl_3$).

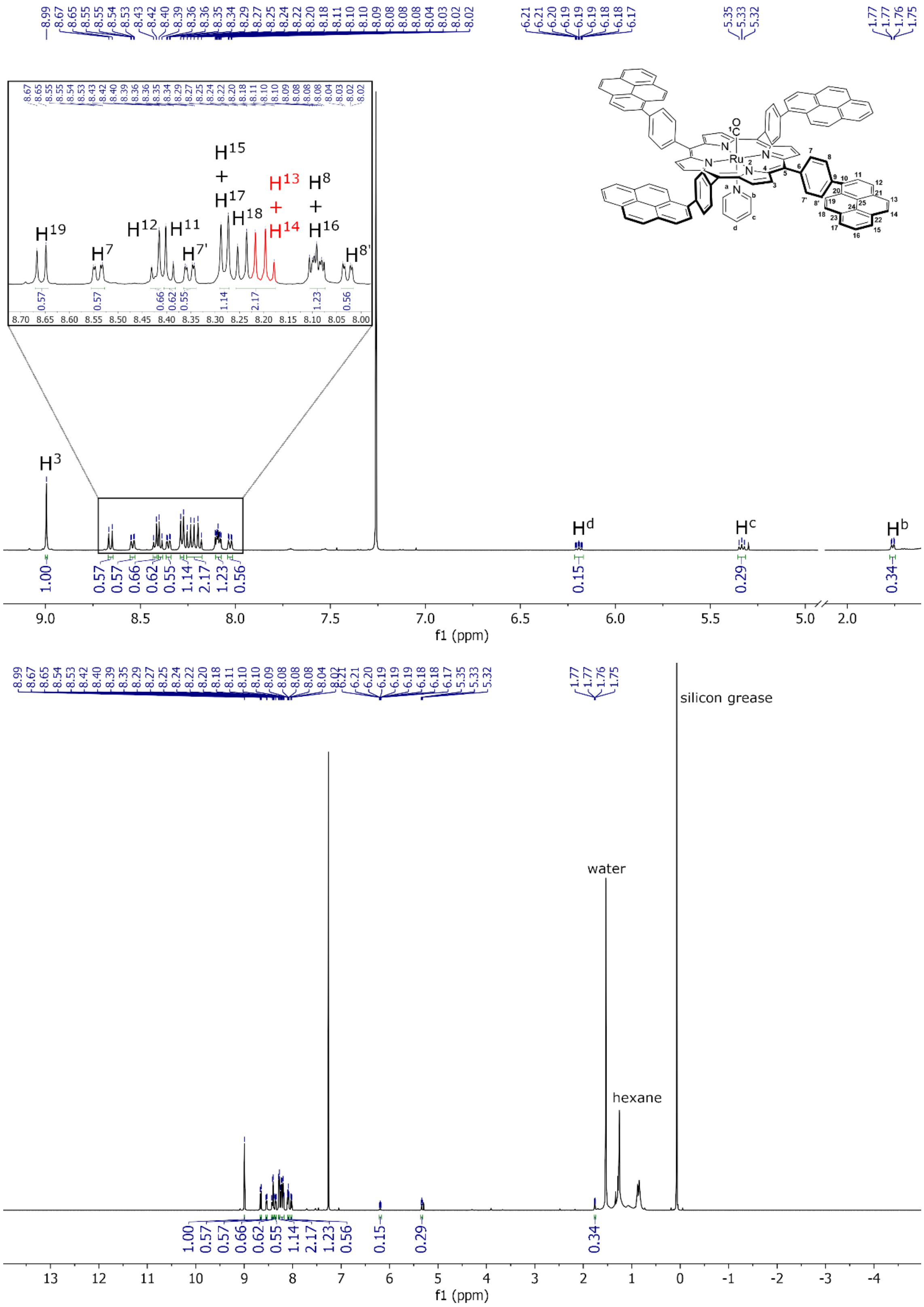

Figure S 42. ^{1}H NMR spectrum of compound **RuP-pyr·py** (500 MHz, $CDCl_3$); selected regions (above) and full spectrum (below). Some signals are depicted in different colors for clarity purposes.

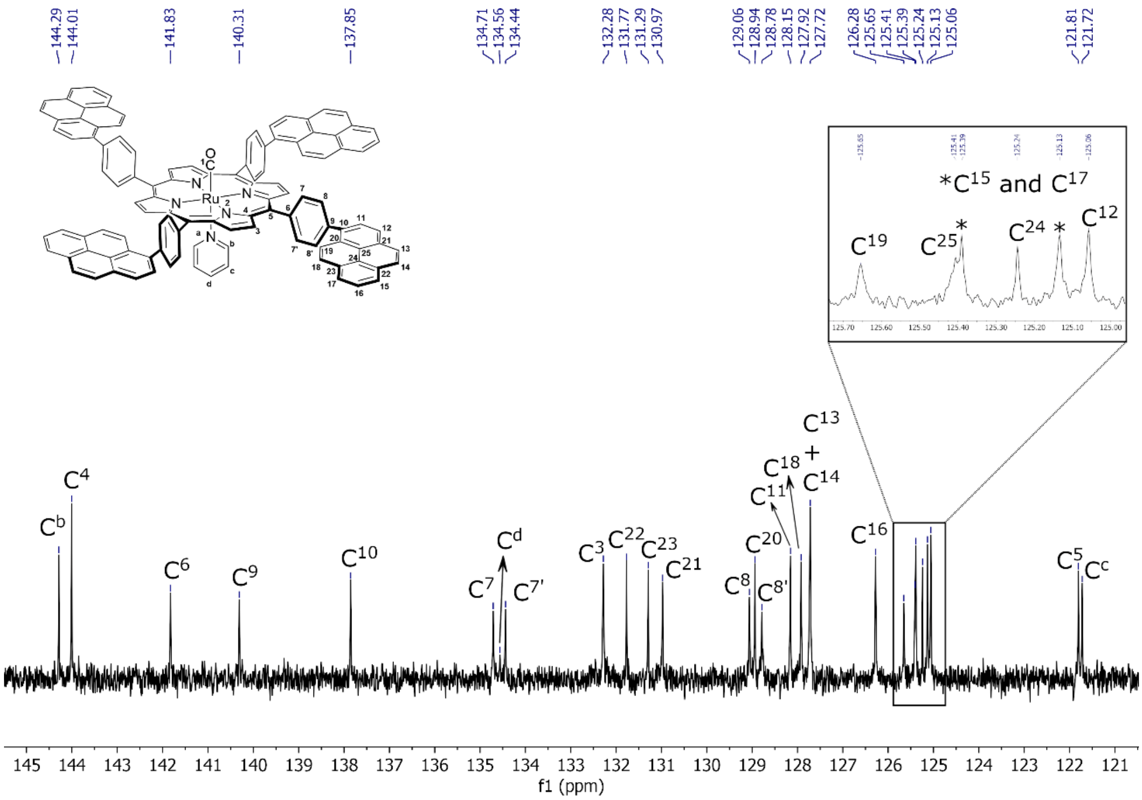

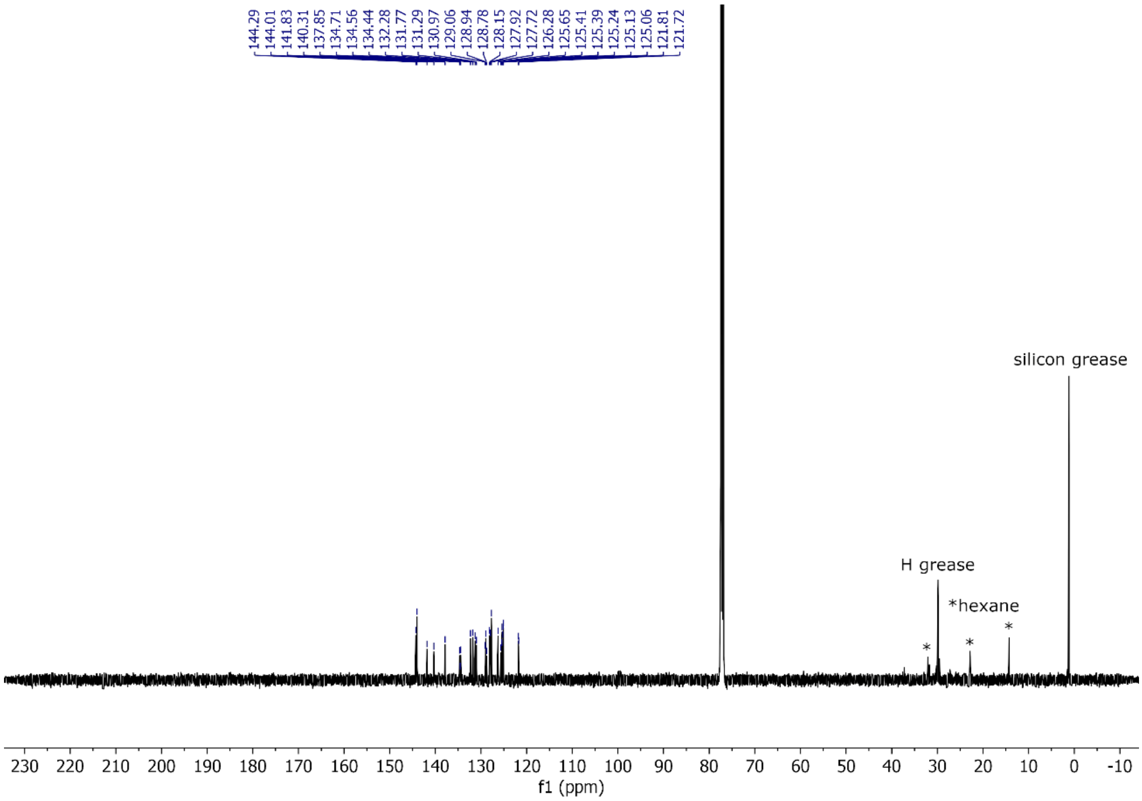

Figure S 43. $^{13}C\{^{1}H\}$ NMR spectrum of compound **RuP-pyr·py** (126 MHz, $CDCl_3$); selected regions (above) and full spectrum (below).

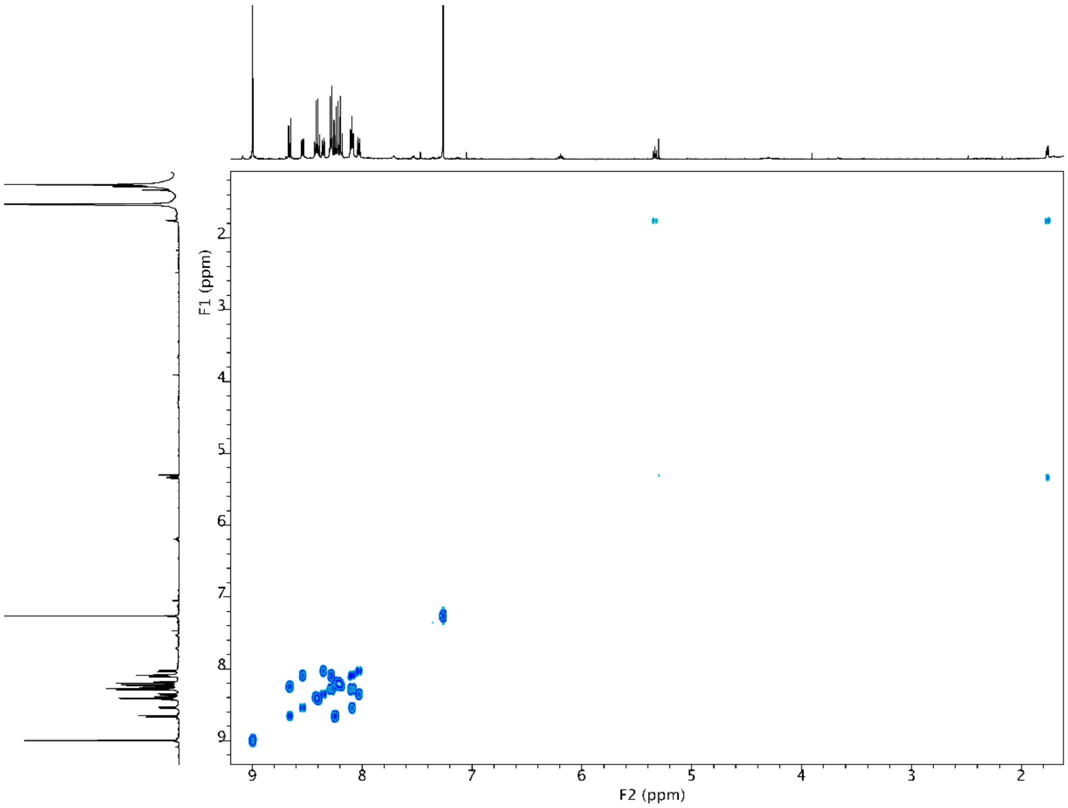

Figure S 44. ^{1}H-^{1}H gCOSY spectrum of compound **RuP-pyr·py** (500 MHz, $CDCl_3$).

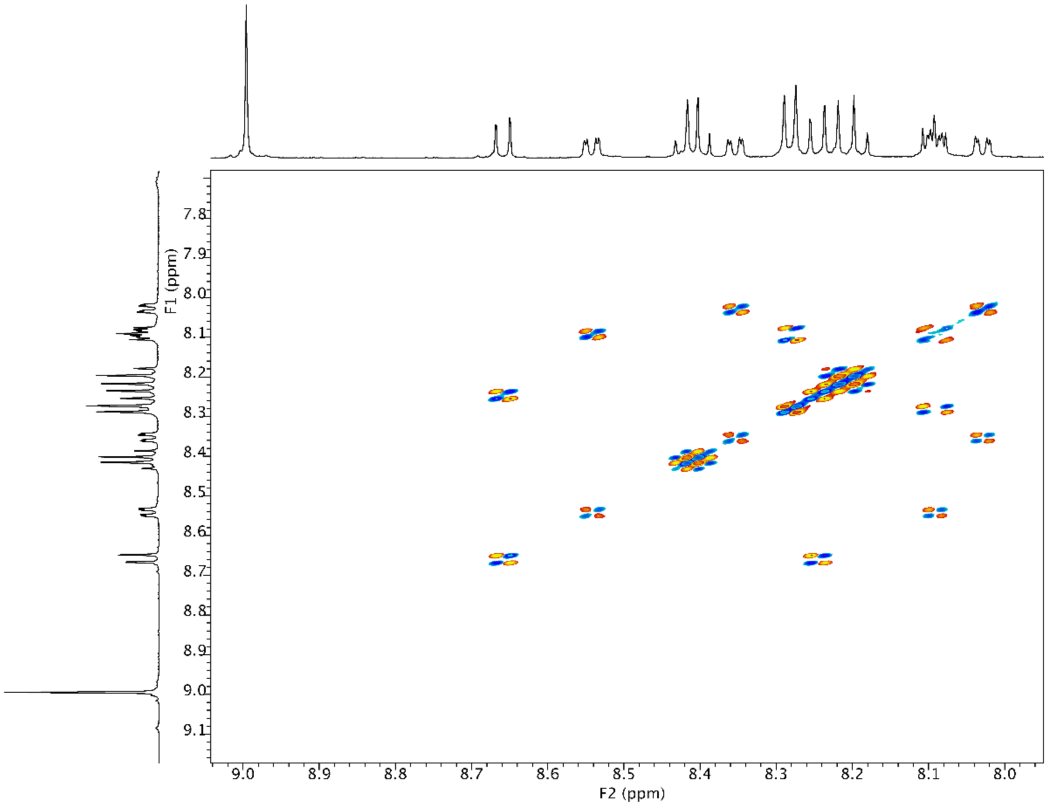

Figure S 45. ^{1}H-^{1}H gDQFCOSY spectrum of compound **RuP-pyr·py** (500 MHz, $CDCl_3$).

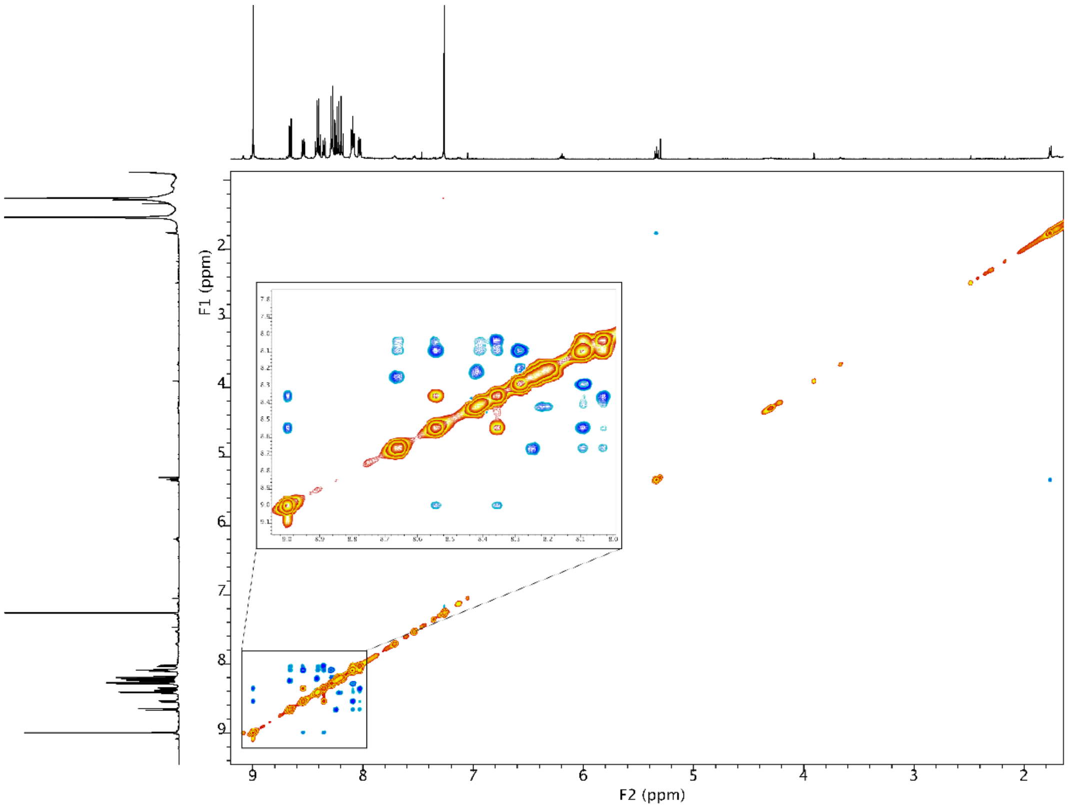

Figure S 46. ^{1}H-^{1}H ROESYAD spectrum of compound **RuP-pyr·py** (500 MHz, $CDCl_3$).

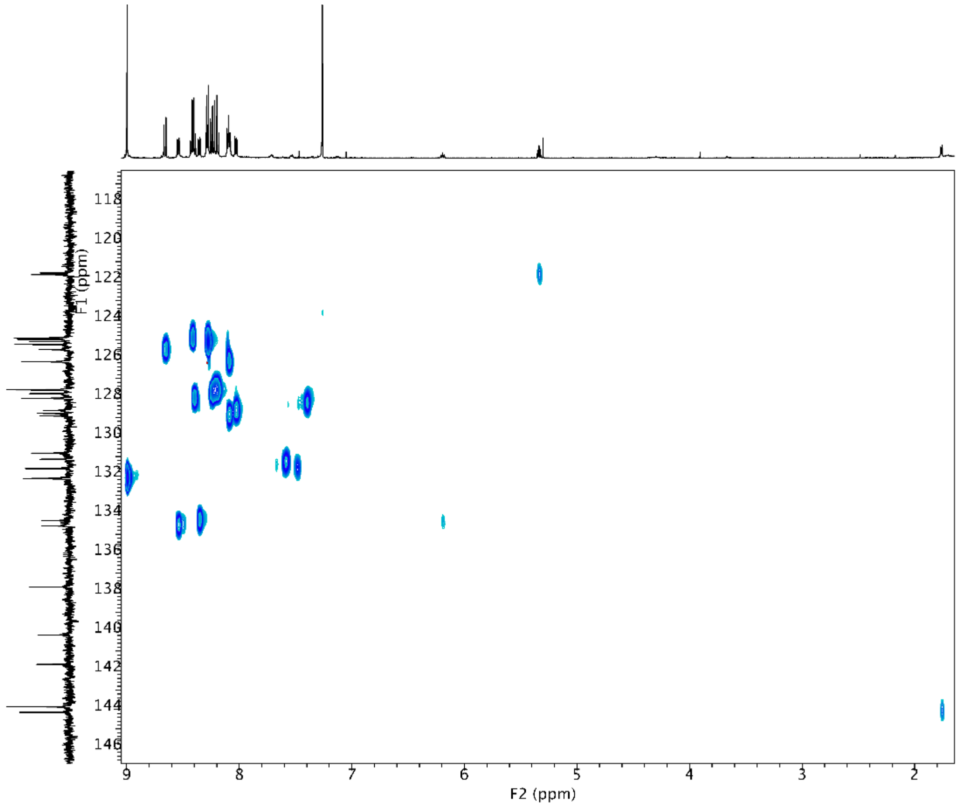

Figure S 47. ^{1}H-^{13}C gc2hsqc spectrum of compound **RuP-pyr·py** (500 MHz, $CDCl_3$).

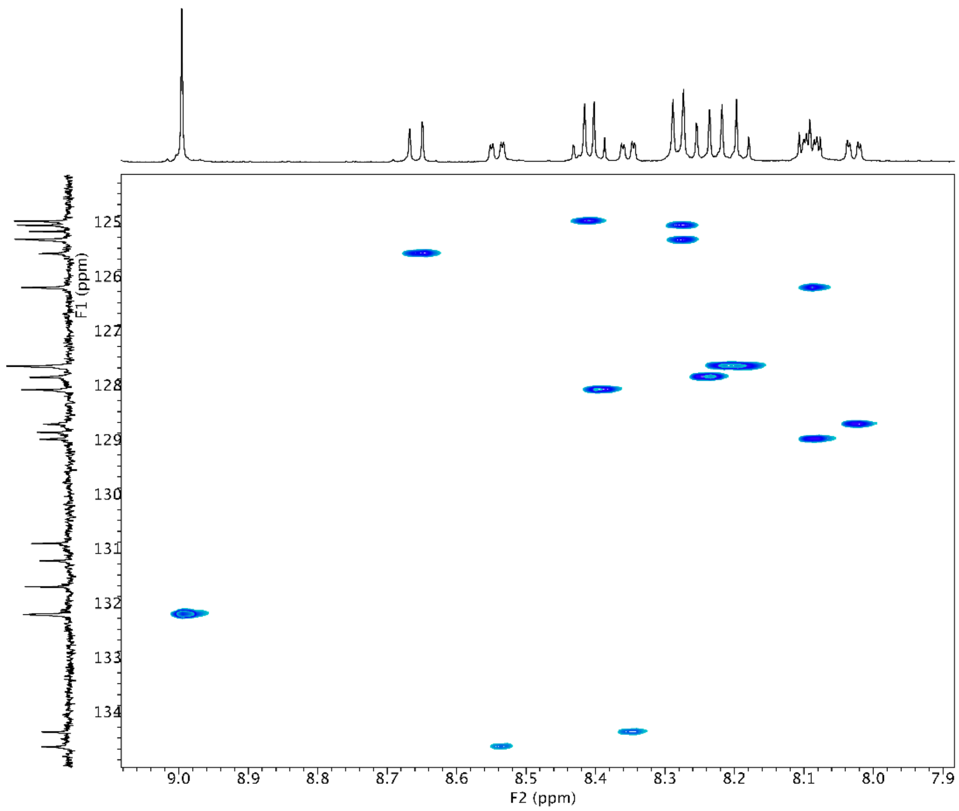

Figure S 48. ^{1}H-^{13}C bsgHSQCAD spectrum of compound **RuP-pyr·py** (500 MHz, $CDCl_3$).

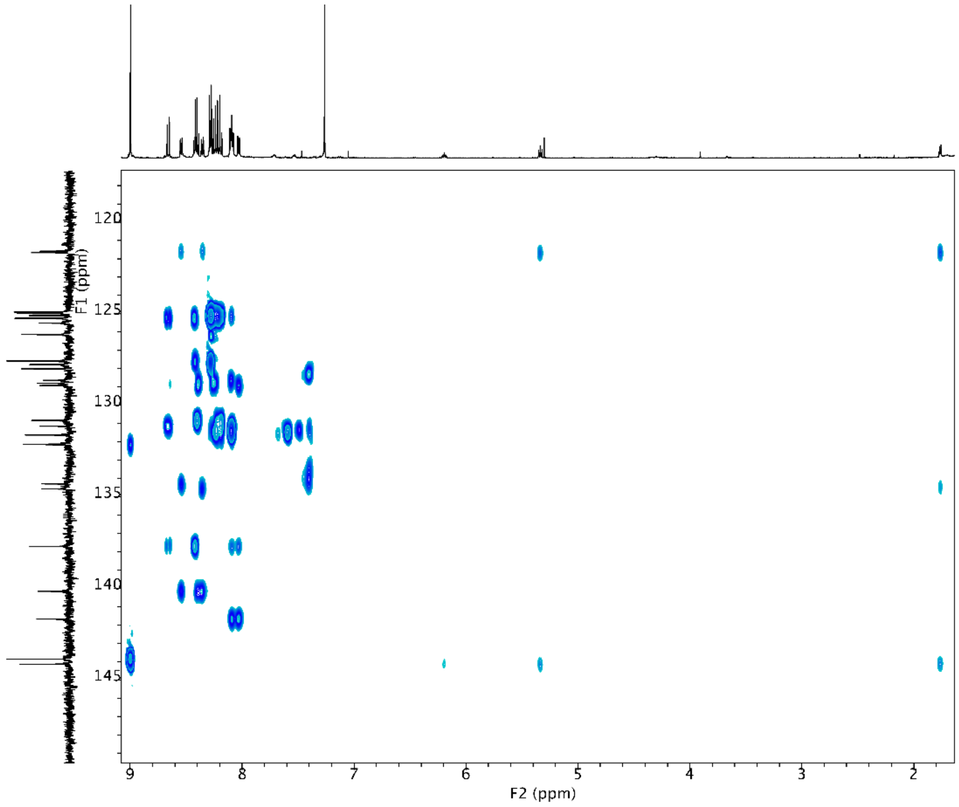

Figure S 49. ^{1}H-^{13}C gc2hmbc spectrum of compound **RuP-pyr·py** (500 MHz, $CDCl_3$).

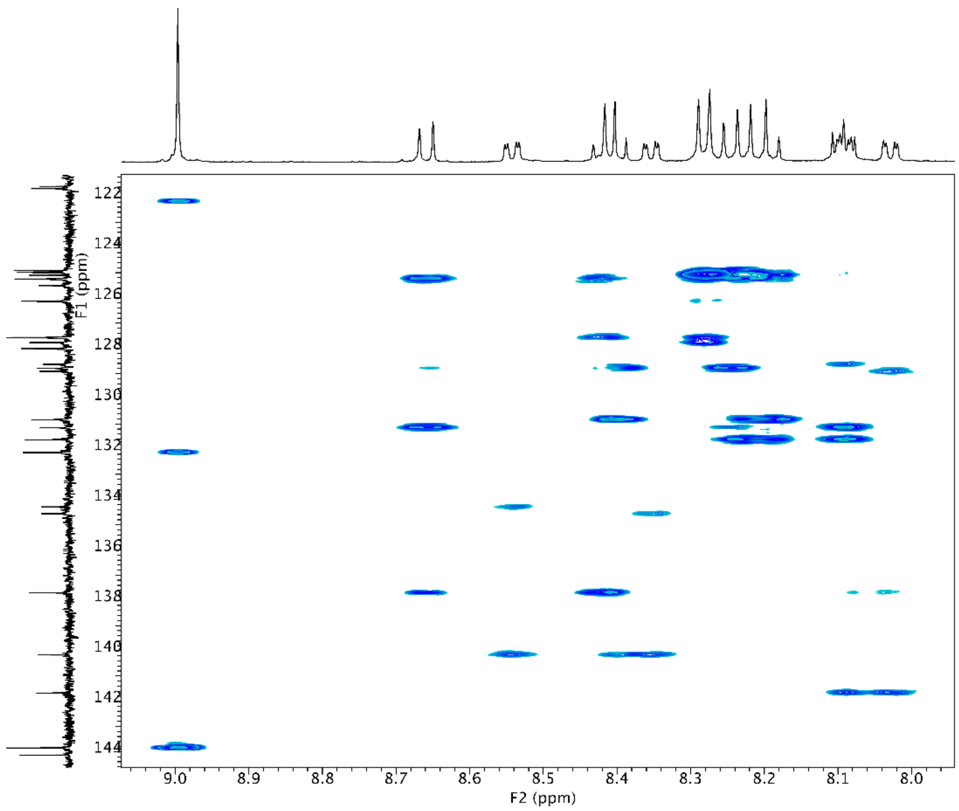

Figure S 50. ^{1}H-^{13}C bsgHMBC spectrum of compound **RuP-pyr·py** (500 MHz, $CDCl_3$).

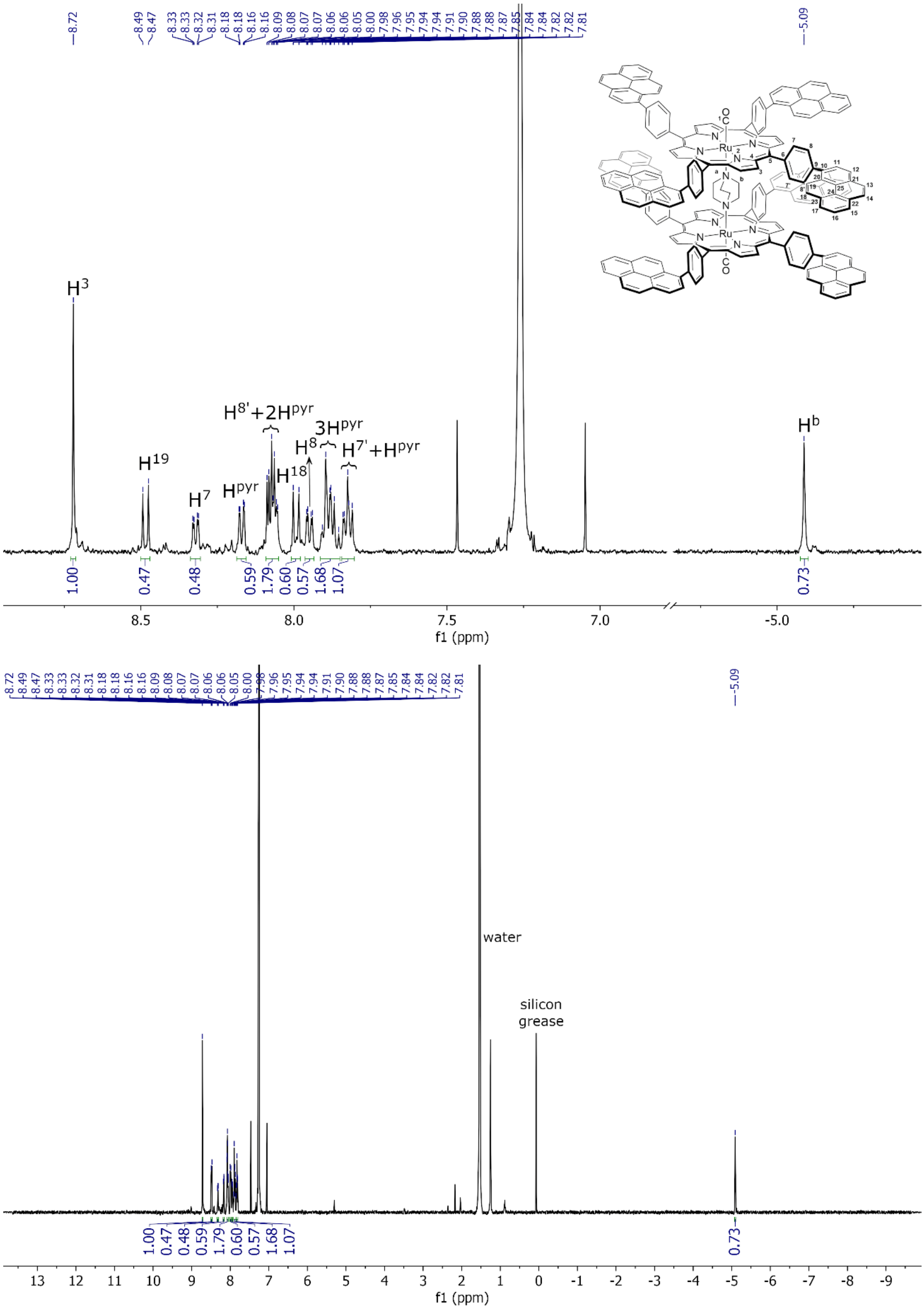

Figure S 51. ^{1}H NMR spectrum of compound **(RuP-pyr)$_2$·DABCO** (500 MHz, $CDCl_3$); selected regions (above) and full spectrum (below).

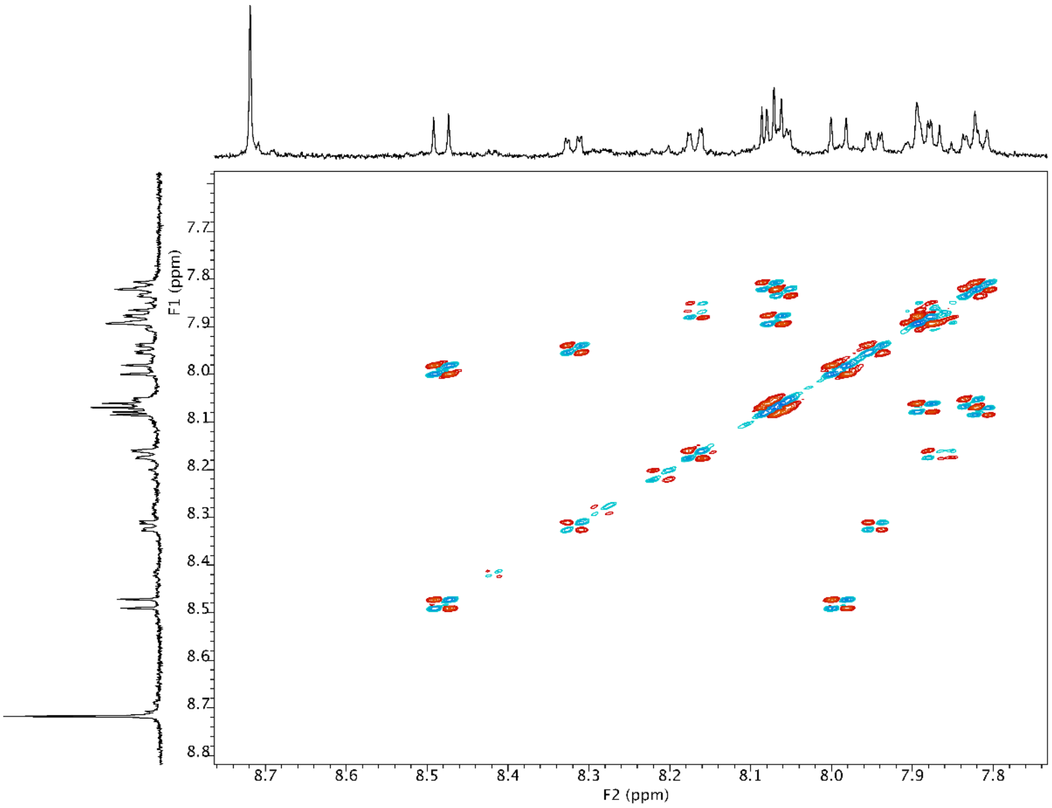

Figure S 52. ^{1}H-^{1}H gDQFCOSY spectrum of compound **(RuP-pyr)$_2$·DABCO** (500 MHz, $CDCl_3$).

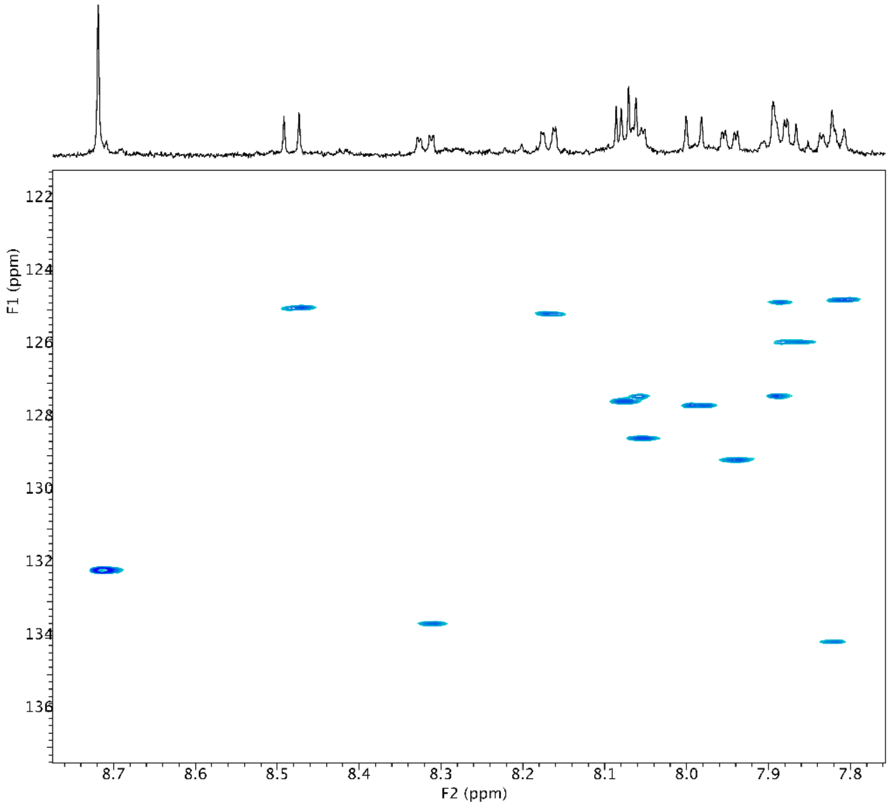

Figure S 53. ^{1}H-^{13}C bsgHSQCAD spectrum of compound **(RuP-pyr)$_2$·DABCO** (500 MHz, $CDCl_3$).

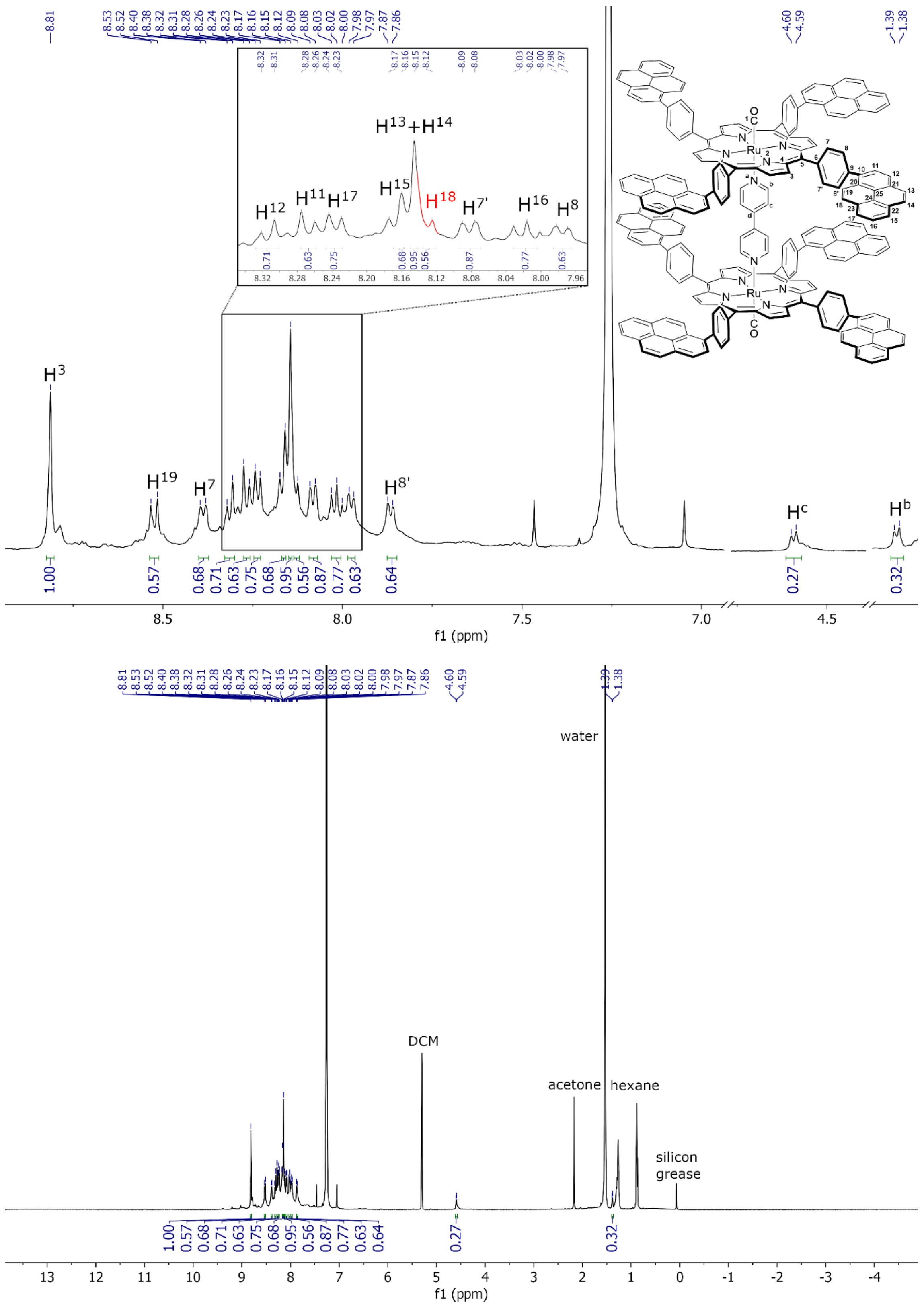

Figure S 54. ^{1}H NMR spectrum of compound **(RuP-pyr)$_2$·bpy** (500 MHz, $CDCl_3$); selected regions (above) and full spectrum (below). Some signals are depicted in different colors for clarity purposes.

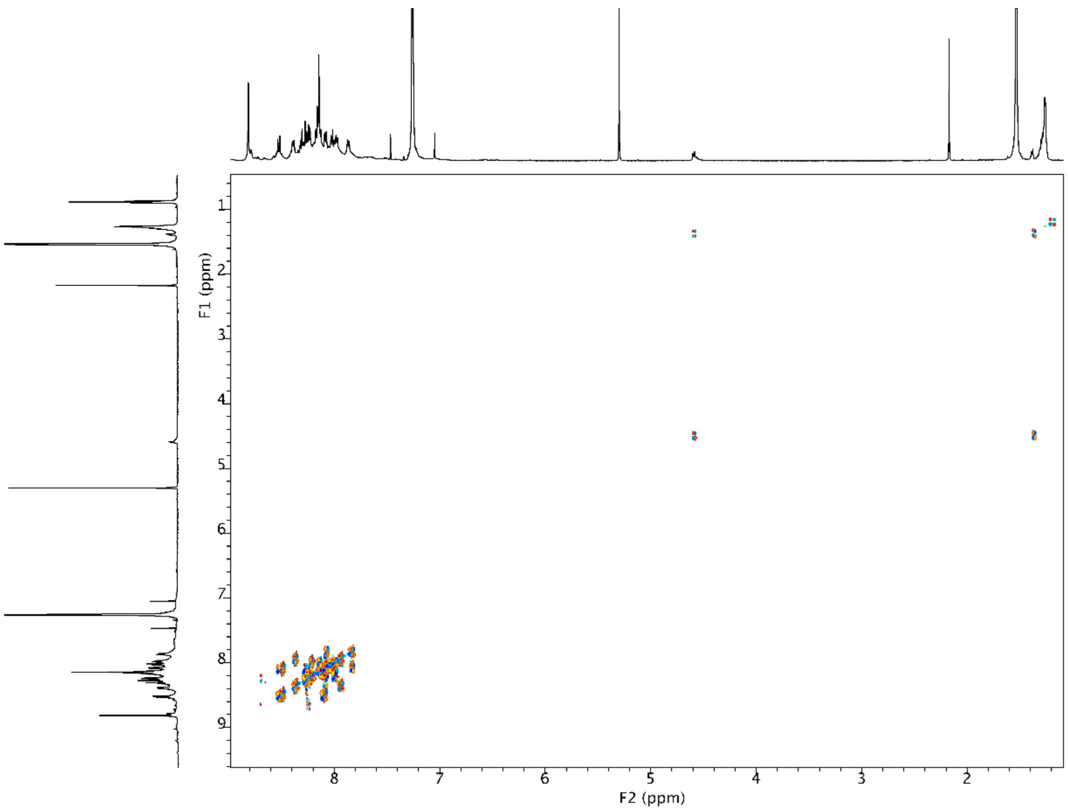

Figure S 55. ^{1}H-^{1}H gDQFCOSY spectrum of compound **(RuP-pyr)$_2$·bpy** (500 MHz, $CDCl_3$).

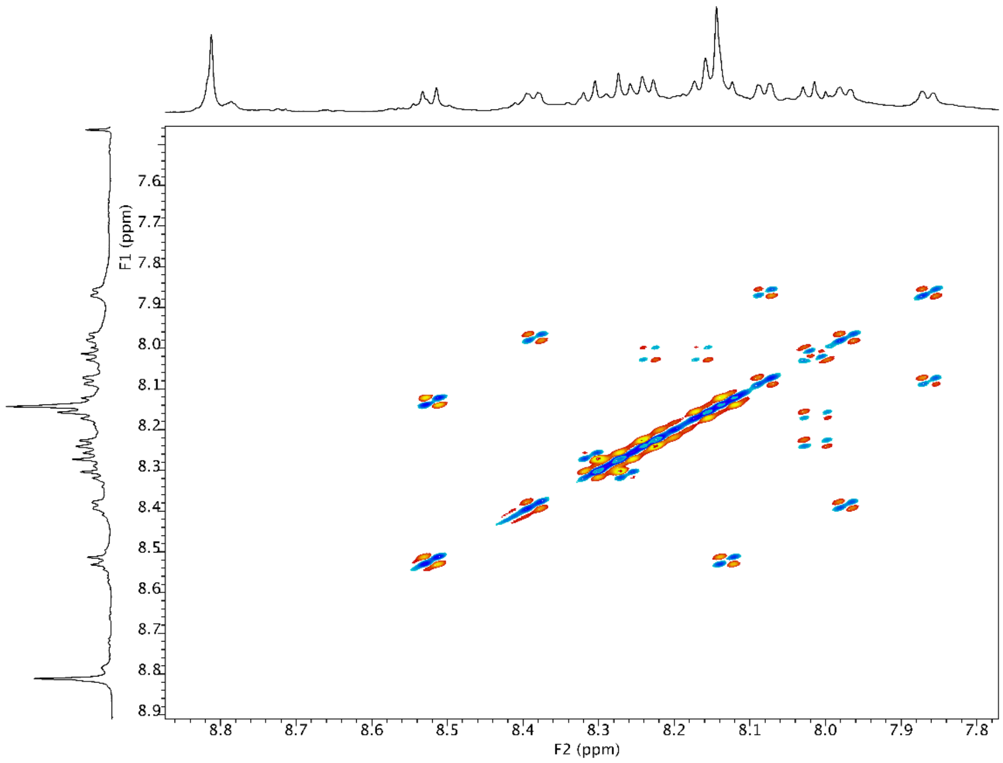

Figure S 56. ^{1}H-^{1}H selective gDQFCOSY spectrum of compound **(RuP-pyr)$_2$·bpy** (500 MHz, $CDCl_3$).

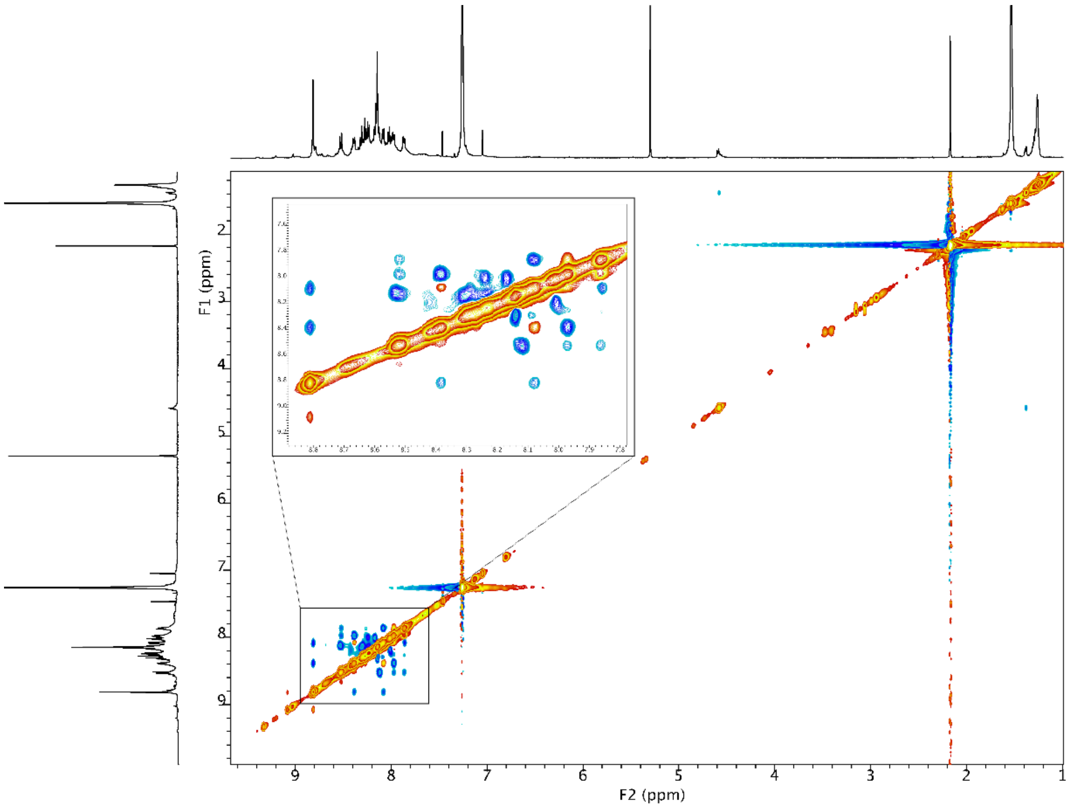

Figure S 57. ^{1}H-^{1}H ROESYAD spectrum of compound **(RuP-pyr)$_2$·bpy** (500 MHz, $CDCl_3$).

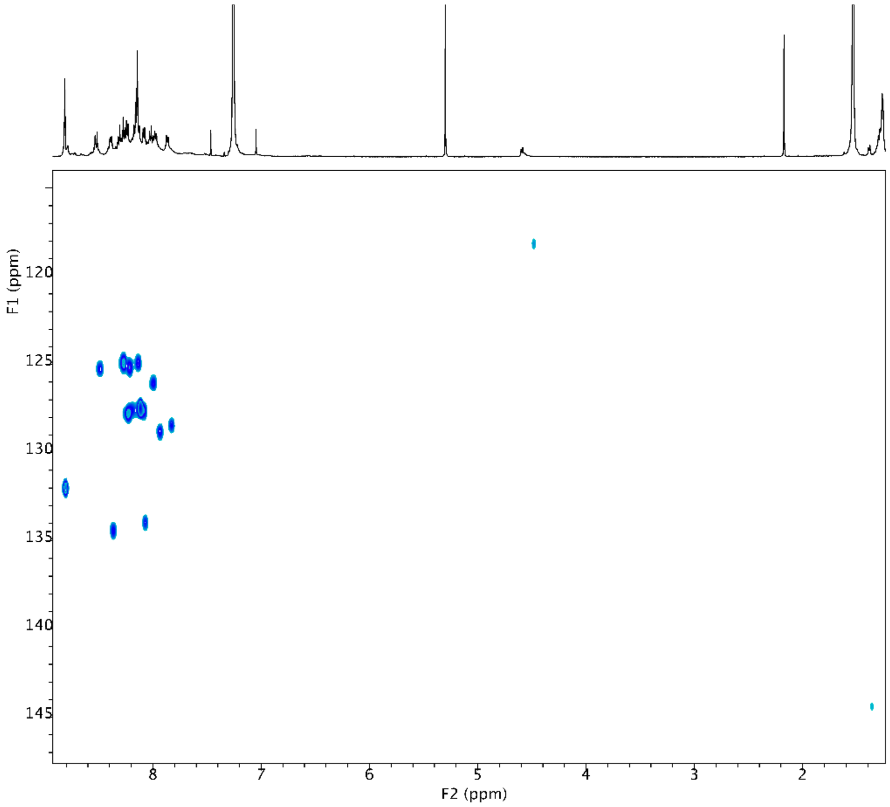

Figure S 58. ^{1}H-^{13}C gc2hsqc spectrum of compound **(RuP-pyr)$_2$·bpy** (500 MHz, $CDCl_3$).

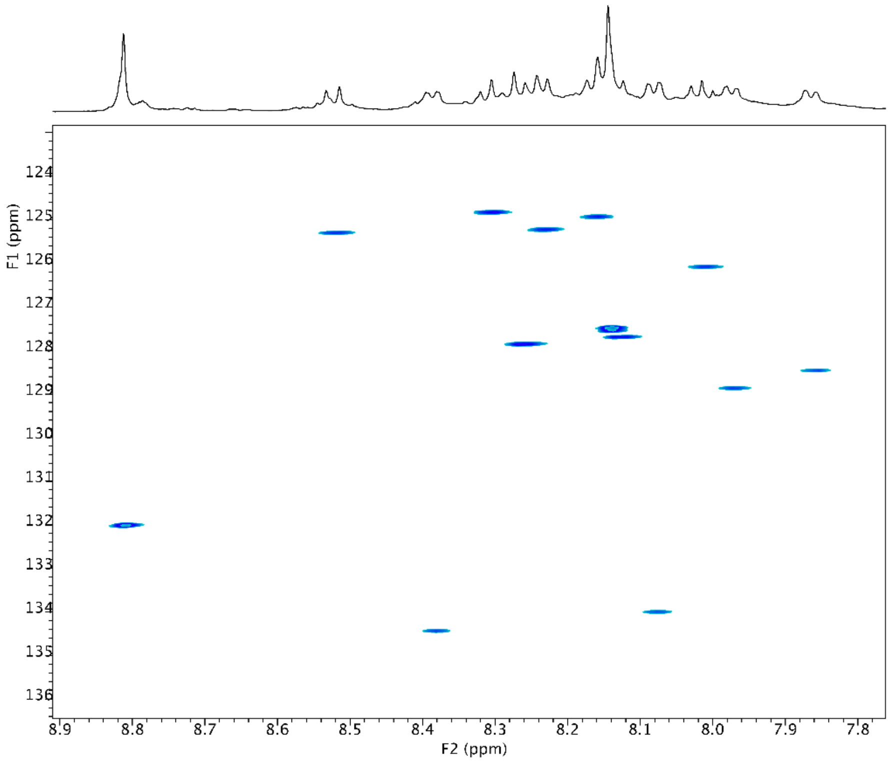

Figure S 59. ^{1}H-^{13}C selective gc2hsqc spectrum of compound **(RuP-pyr)$_2$·bpy** (500 MHz, $CDCl_3$).

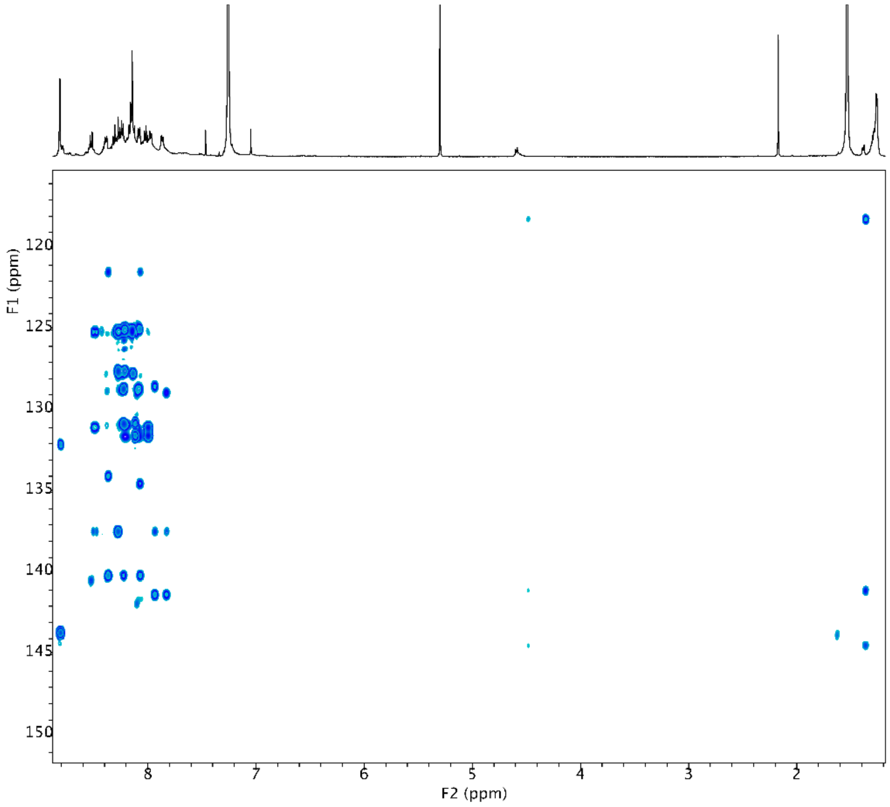

Figure S 60. ^{1}H-^{13}C gc2hmbc spectrum of compound **(RuP-pyr)$_2$·bpy** (500 MHz, $CDCl_3$).

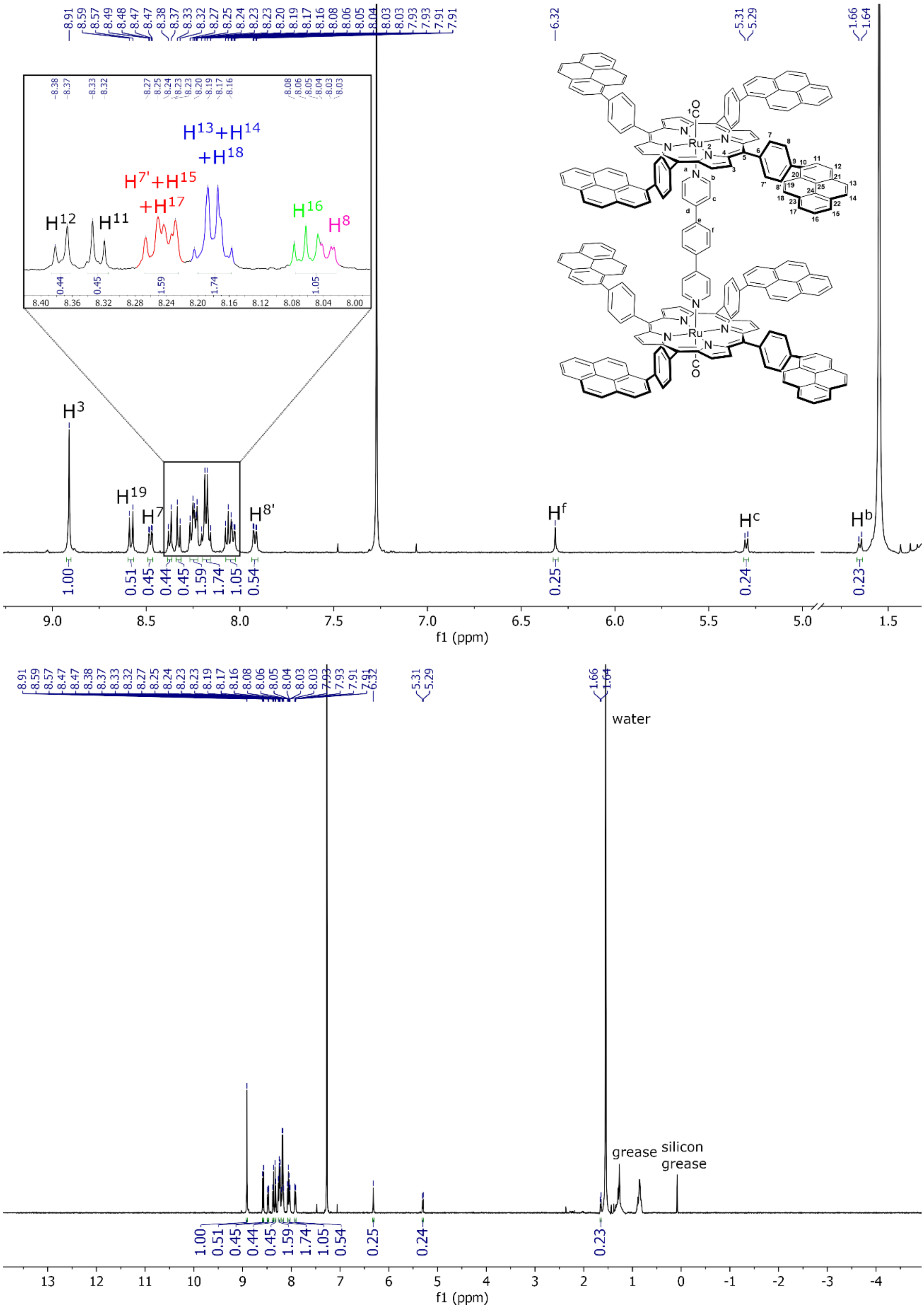

Figure S 61. ^{1}H NMR spectrum of compound **(RuP-pyr)$_2$·dpyb** (500 MHz, $CDCl_3$); selected regions (above) and full spectrum (below). Some signals are depicted in different colors for clarity purposes.

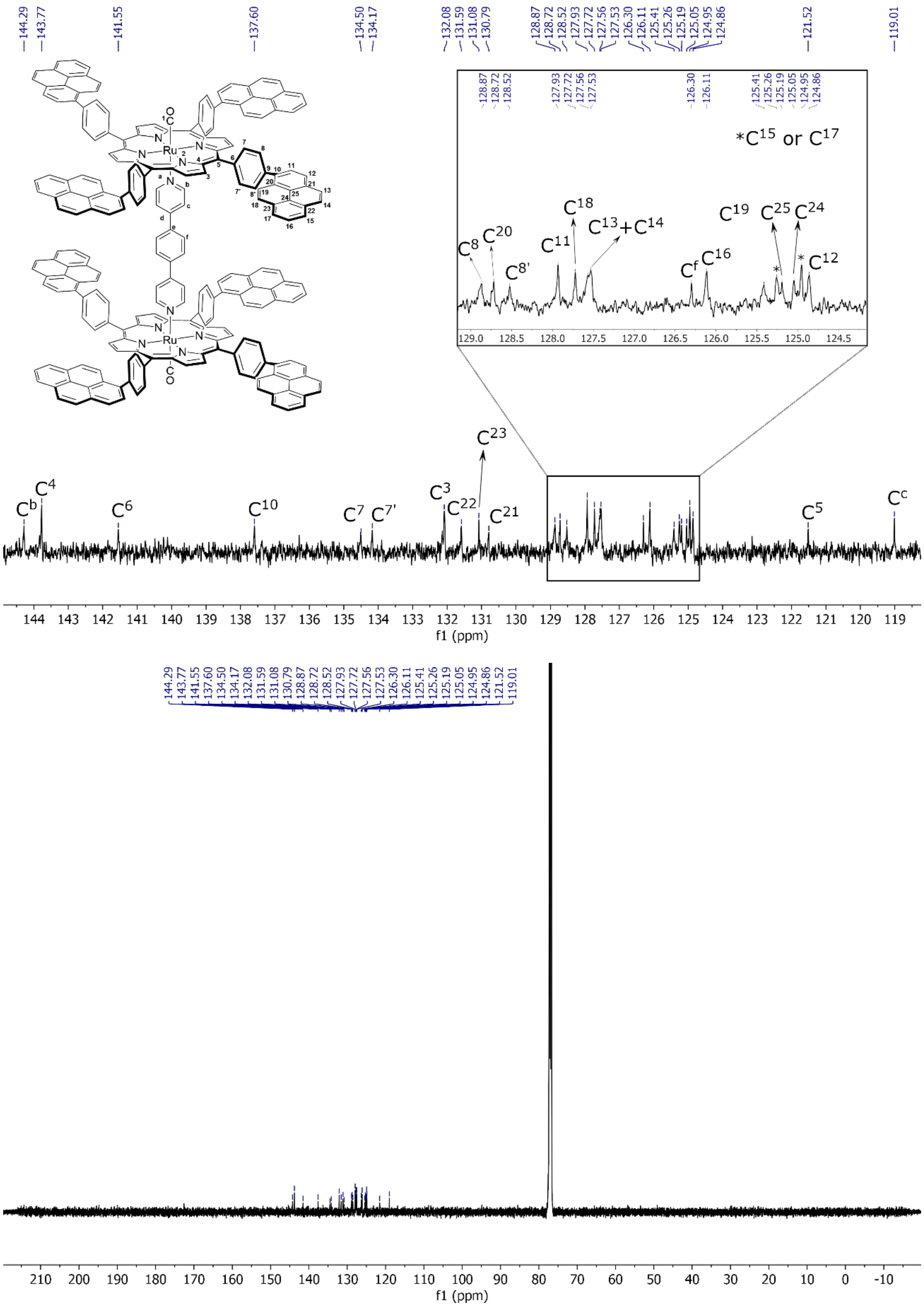

Figure S 62. $^{13}C\{^{1}H\}$ spectrum of compound **(RuP-pyr)₂·dpyb** (126 MHz, $CDCl_3$); selected regions (above) and full spectrum (below).

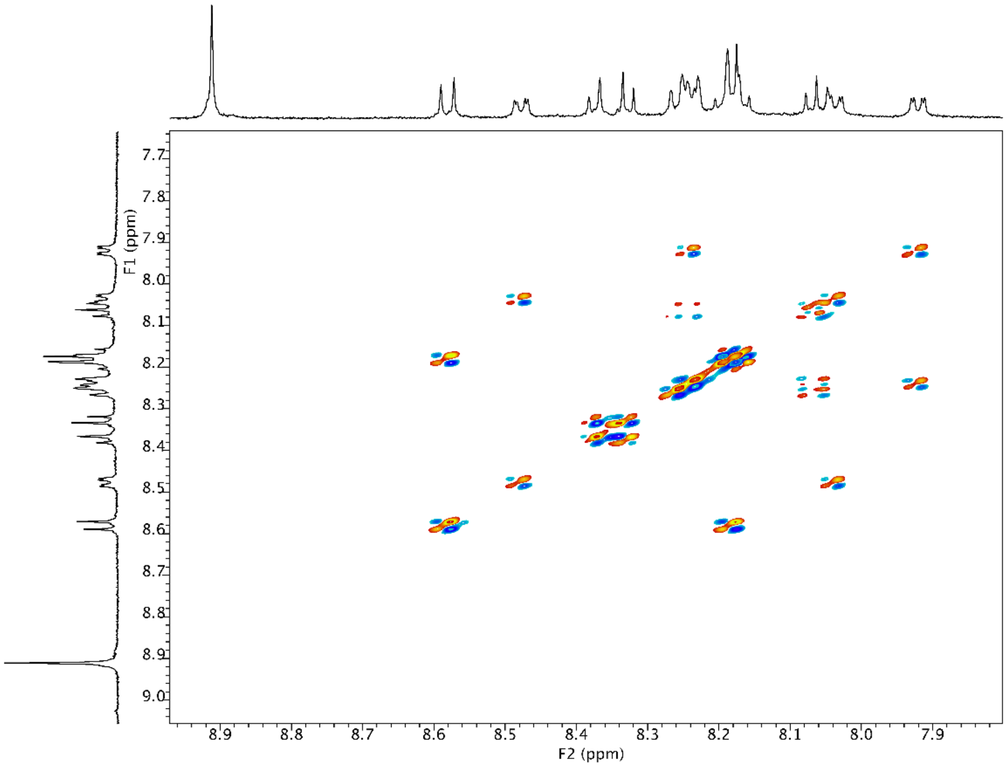

Figure S 63. ^{1}H-^{1}H gDQFCOSY spectrum of compound **(RuP-pyr)$_2$·dpyb** (500 MHz, $CDCl_3$).

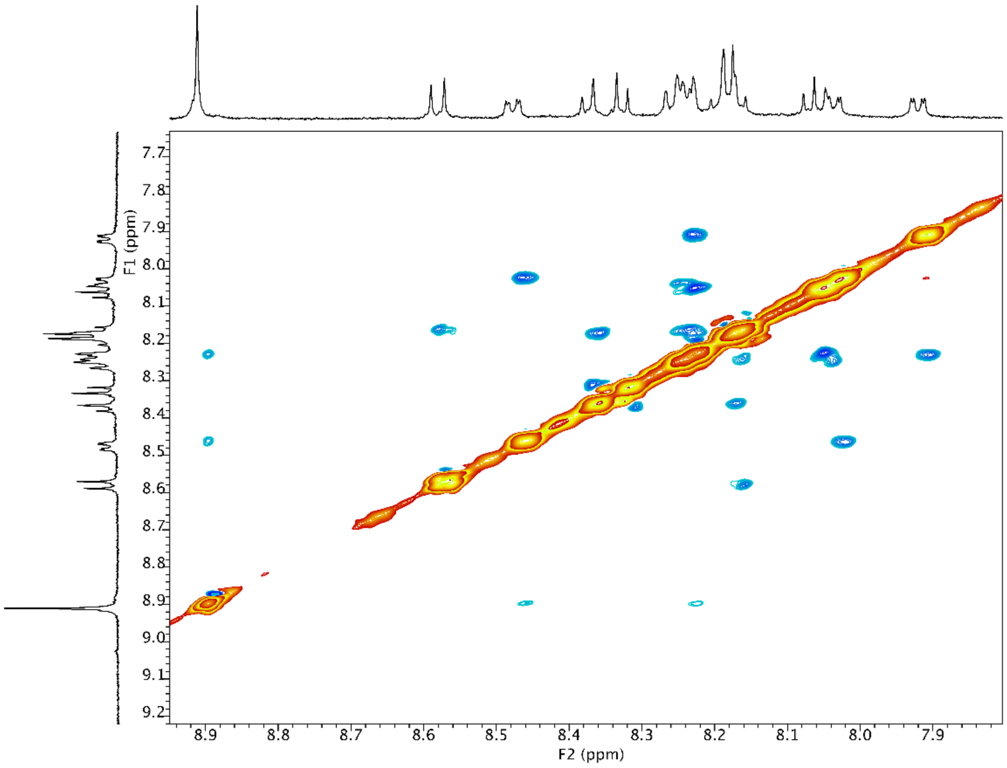

Figure S 64. ^{1}H-^{1}H ROESYAD spectrum of compound **(RuP-pyr)$_2$·dpyb** (500 MHz, $CDCl_3$).

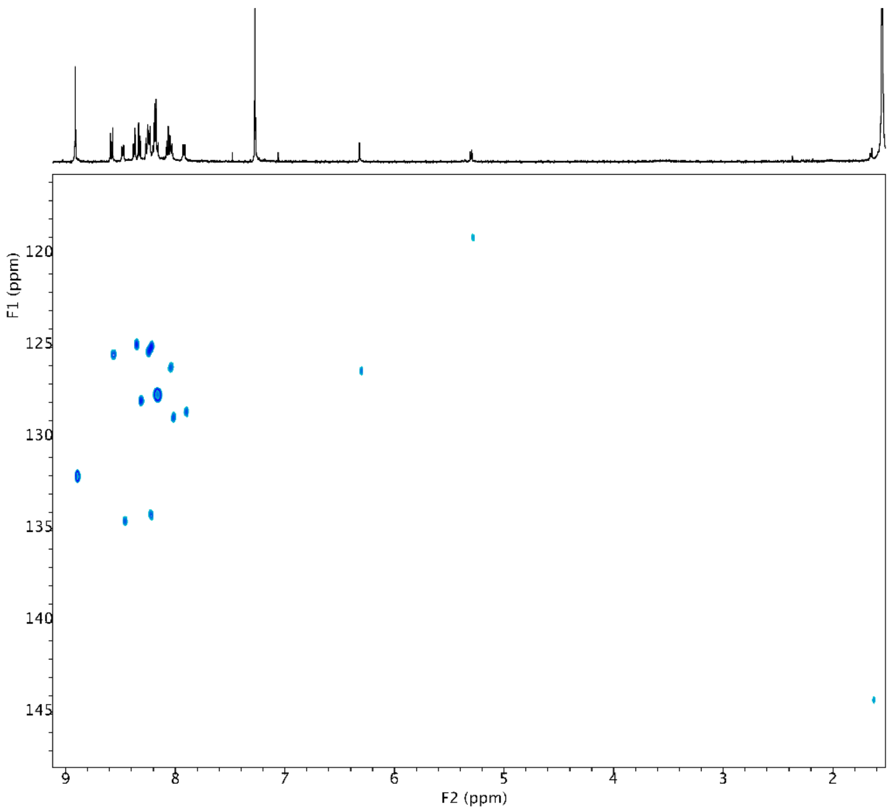

Figure S 65. ^{1}H-^{13}C gc2hsqc spectrum of compound **(RuP-pyr)$_2$·dpyb** (500 MHz, $CDCl_3$).

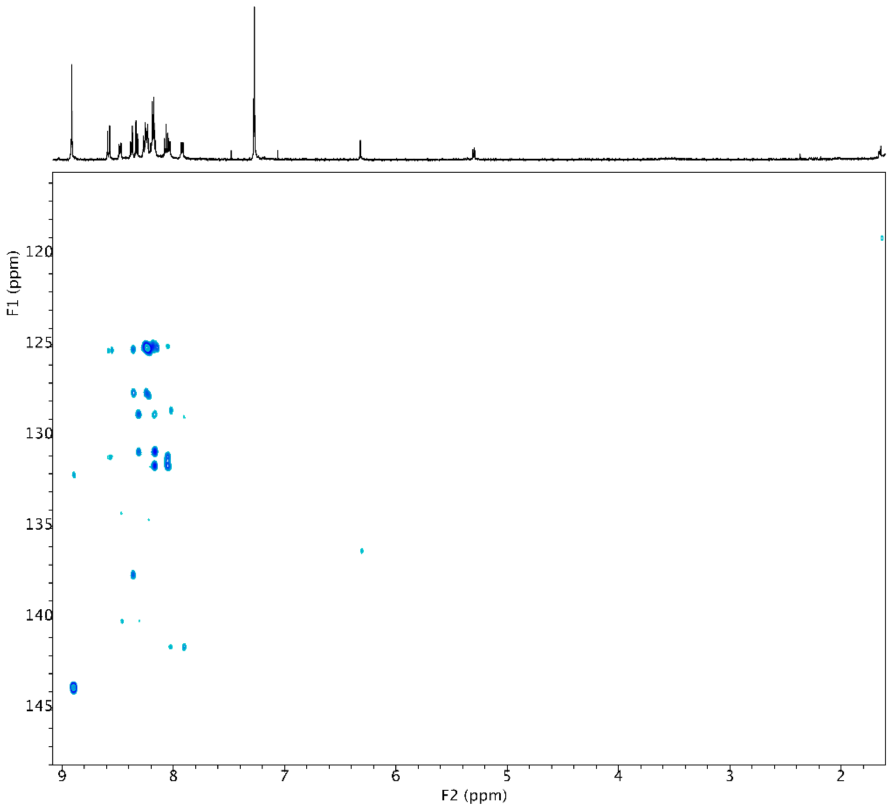

Figure S 66. ^{1}H-^{13}C gc2hmbc spectrum of compound **(RuP-pyr)$_2$·dpyb** (500 MHz, $CDCl_3$).

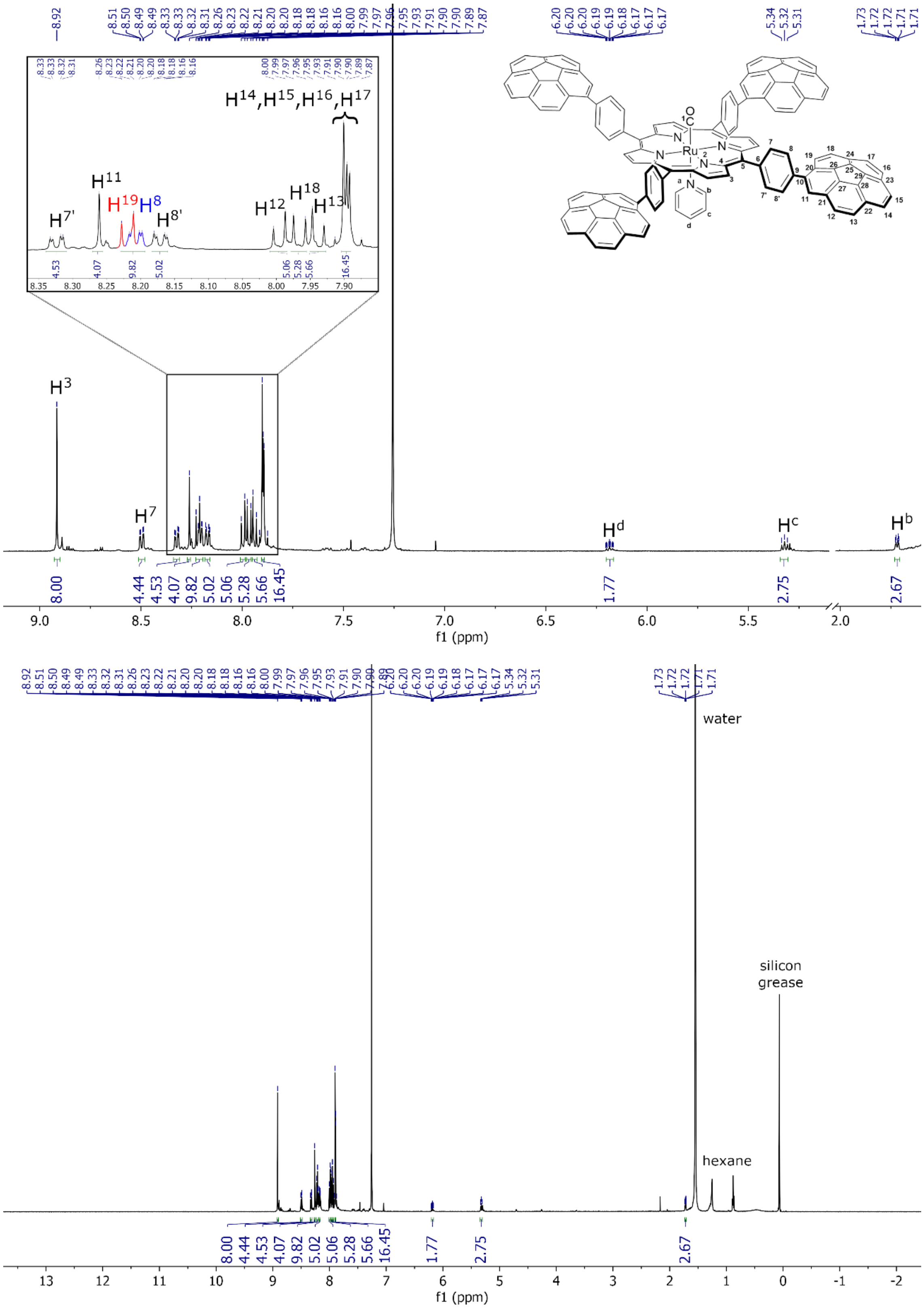

Figure S 67. ^{1}H NMR spectrum of compound **RuP-cor·py** (500 MHz, $CDCl_3$); selected regions (above) and full spectrum (below). Some signals are depicted in different colors for clarity purposes.

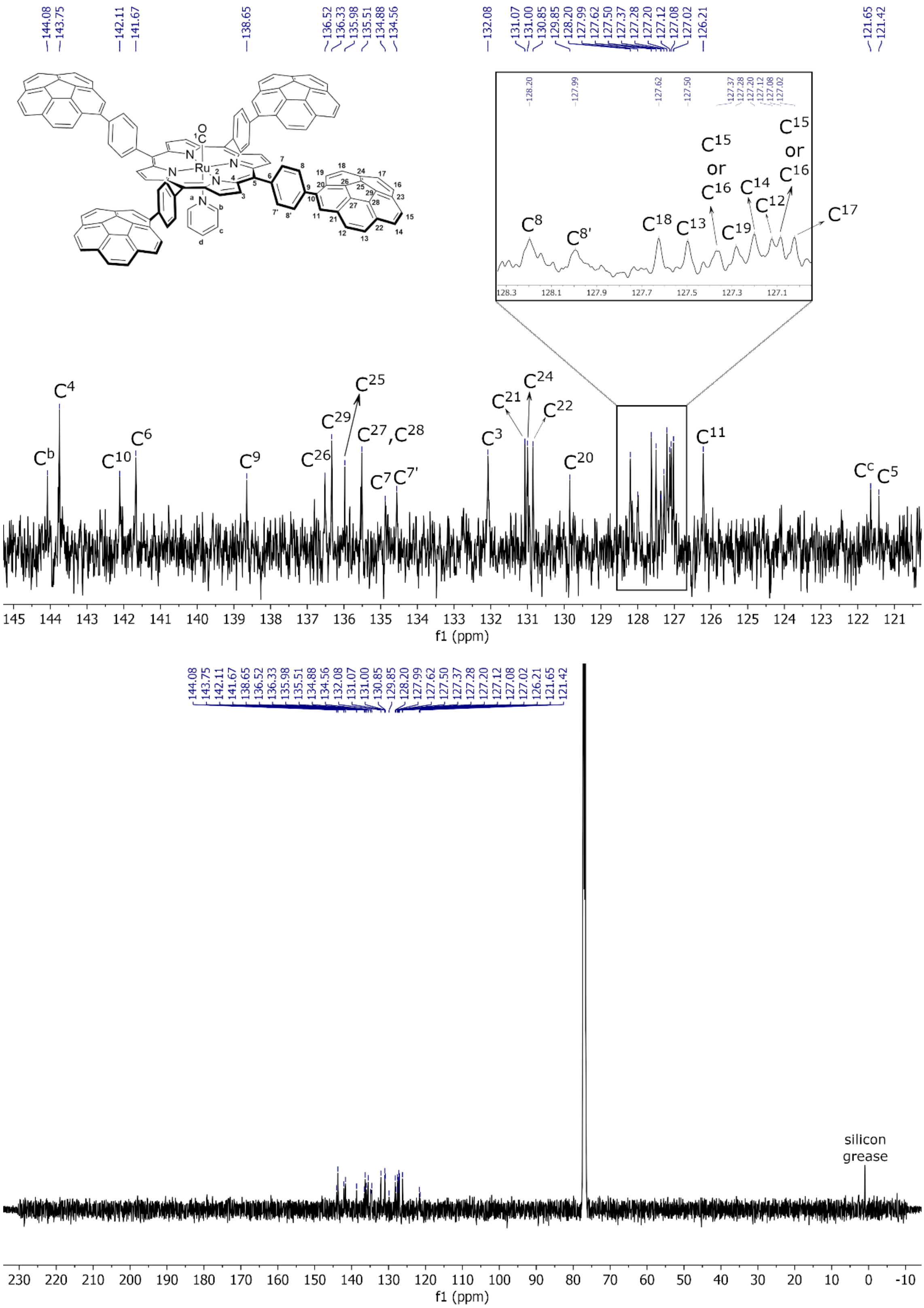

Figure S 68. $^{13}C\{^{1}H\}$ NMR spectrum of compound **RuP-cor·py** (126 MHz, $CDCl_3$); selected regions (above) and full spectrum (below).

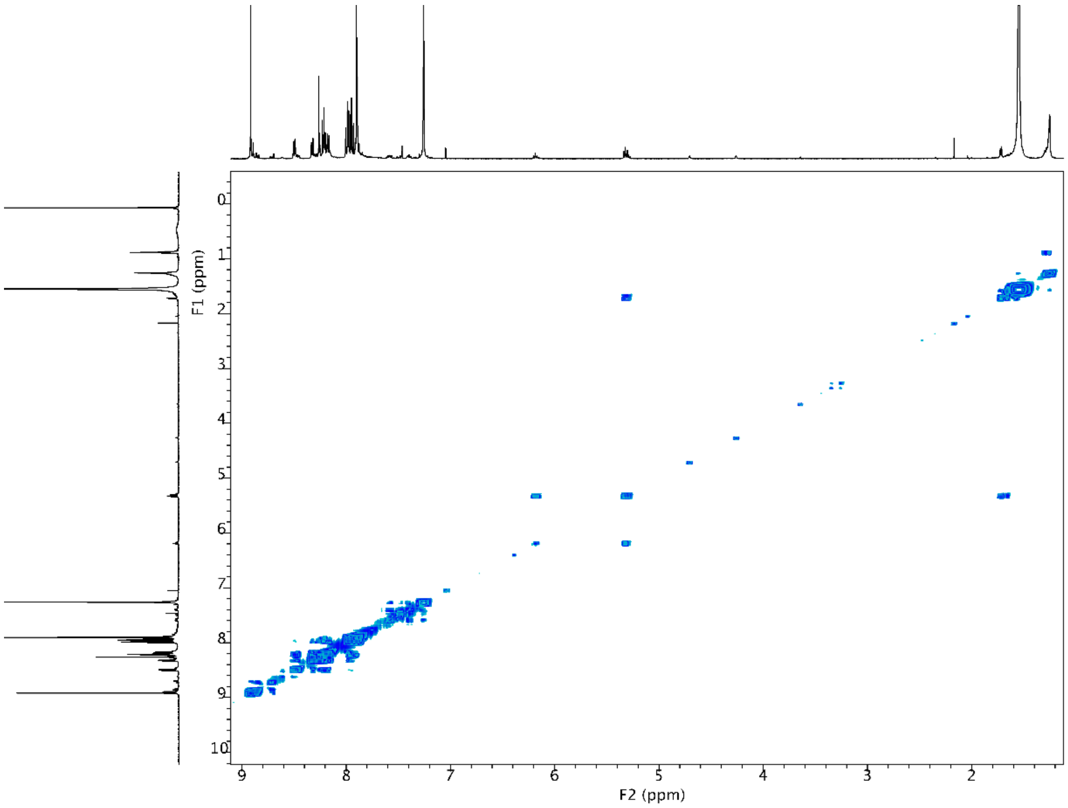

Figure S 69. ^{1}H-^{1}H gCOSY spectrum of compound **RuP-cor·py** (500 MHz, $CDCl_3$).

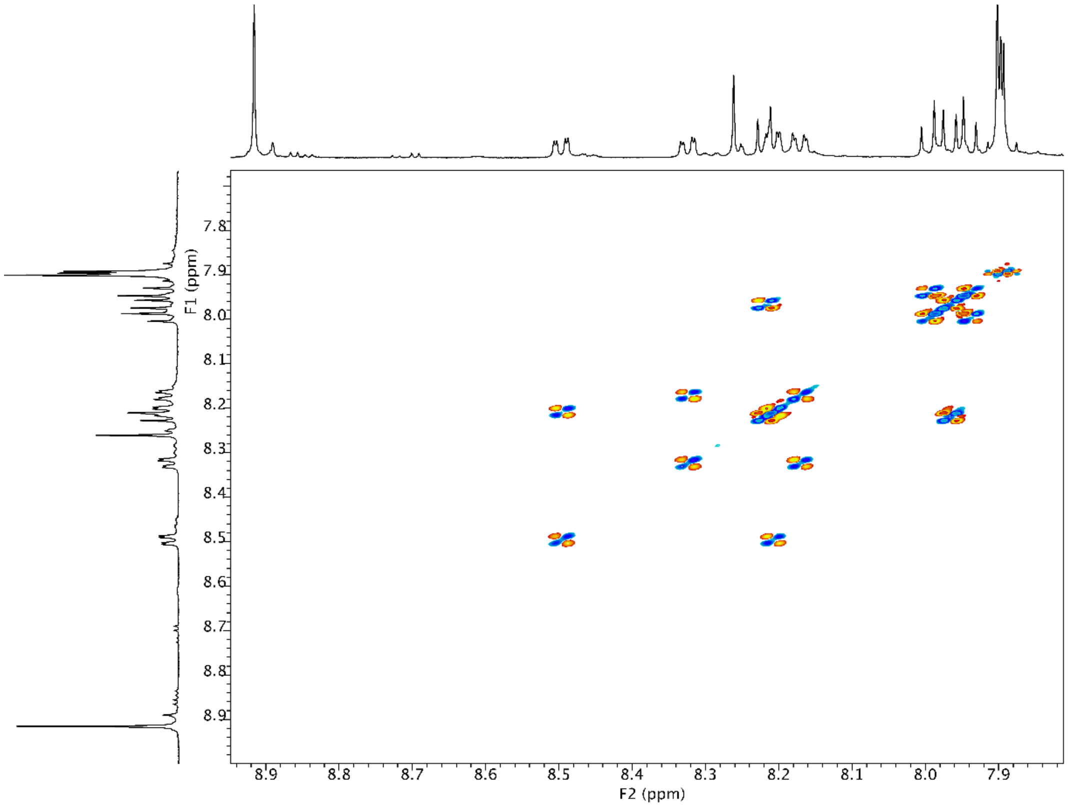

Figure S 70. ^{1}H-^{1}H gDQFCOSY spectrum of compound **RuP-cor·py** (500 MHz, $CDCl_3$).

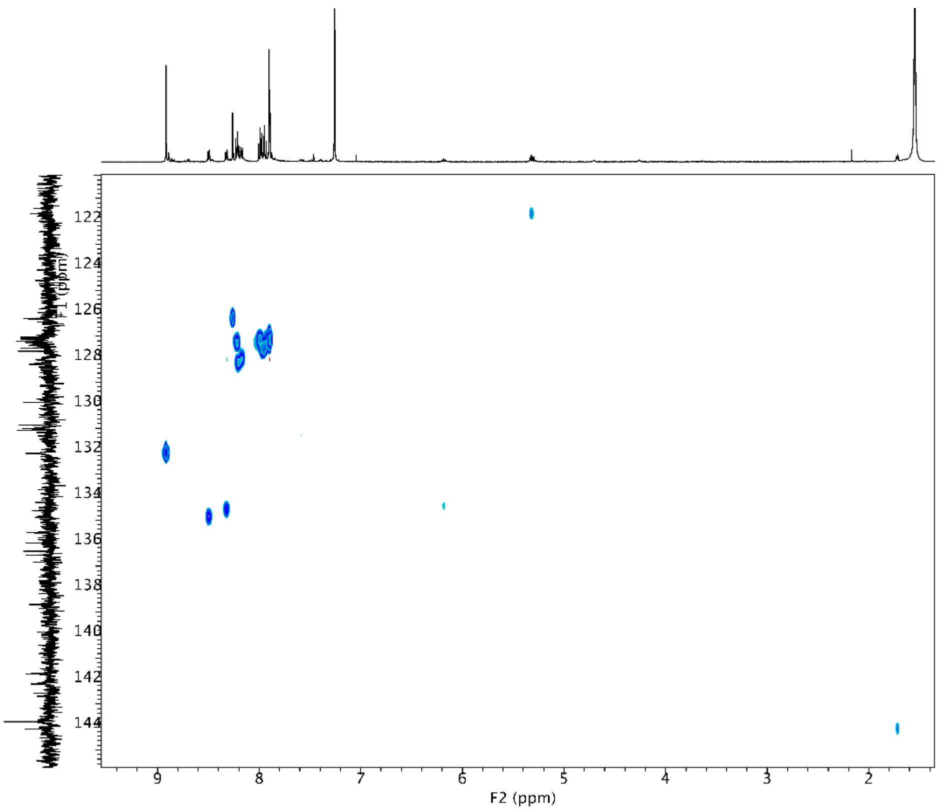

Figure S 71. ^{1}H-^{13}C gc2hsqc spectrum of compound **RuP-cor·py** (500 MHz, $CDCl_3$).

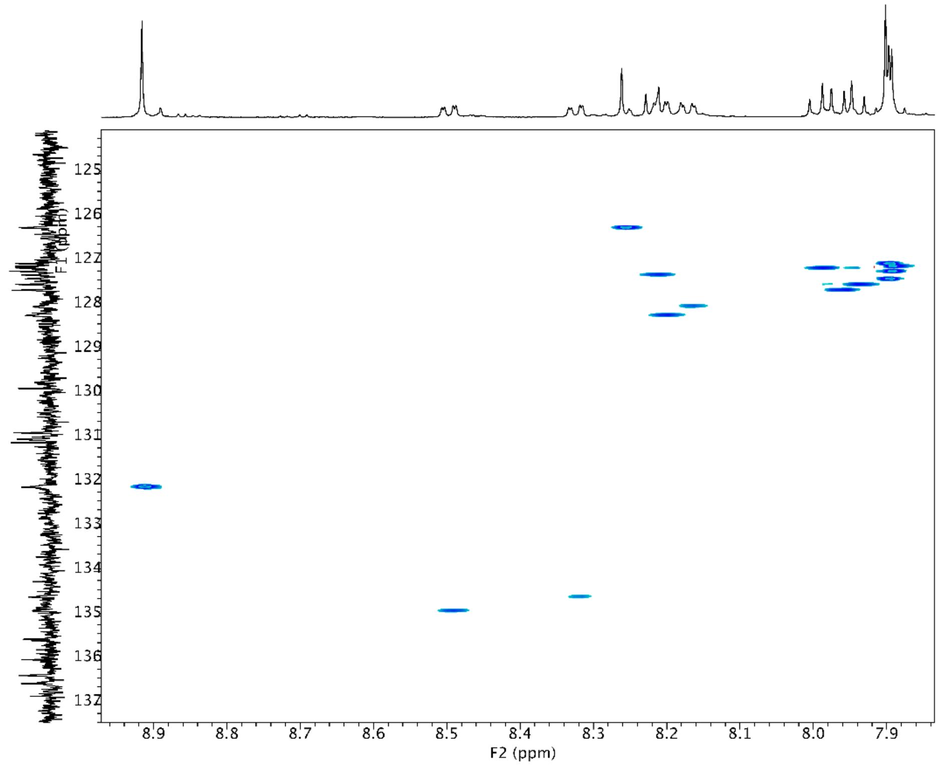

Figure S 72. ^{1}H-^{13}C bsgHSQCAD spectrum of compound **RuP-cor·py** (500 MHz, $CDCl_3$).

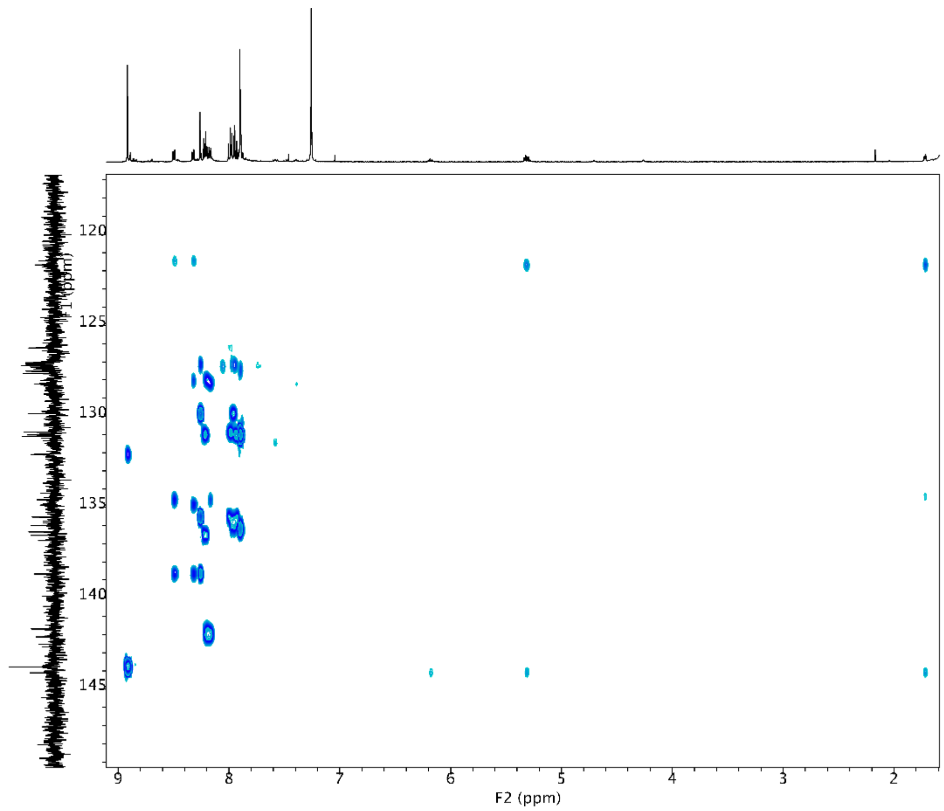

Figure S 73. ^{1}H-^{13}C gc2hmbc spectrum of compound **RuP-cor·py** (500 MHz, $CDCl_3$).

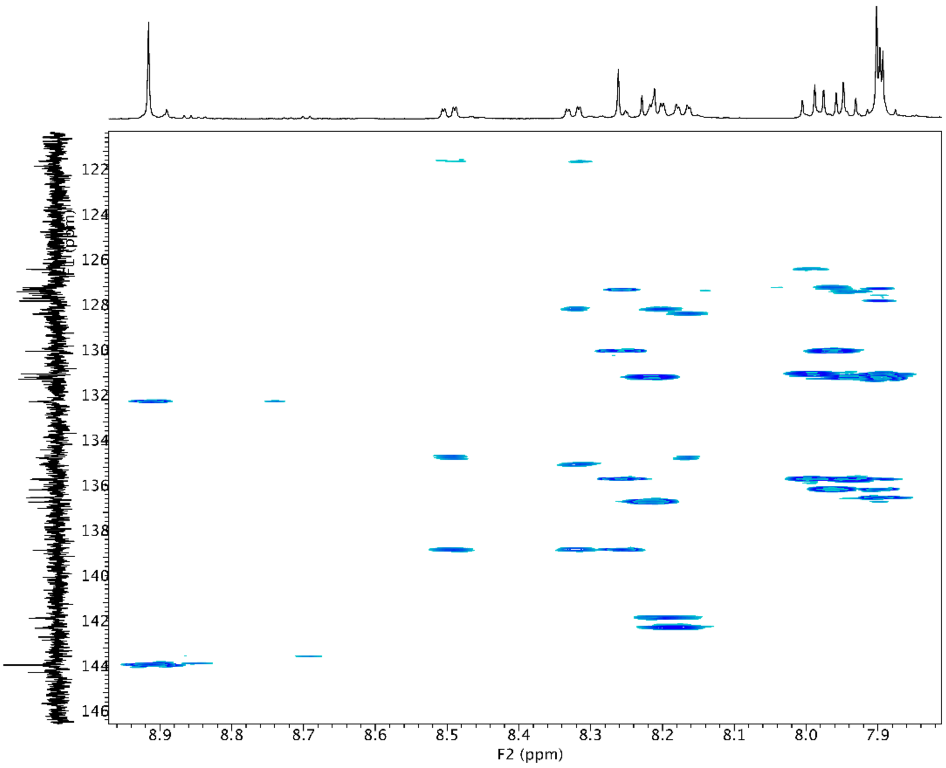

Figure S 74. ^{1}H-^{13}C bsgHMBC spectrum of compound **RuP-cor·py** (500 MHz, $CDCl_3$).

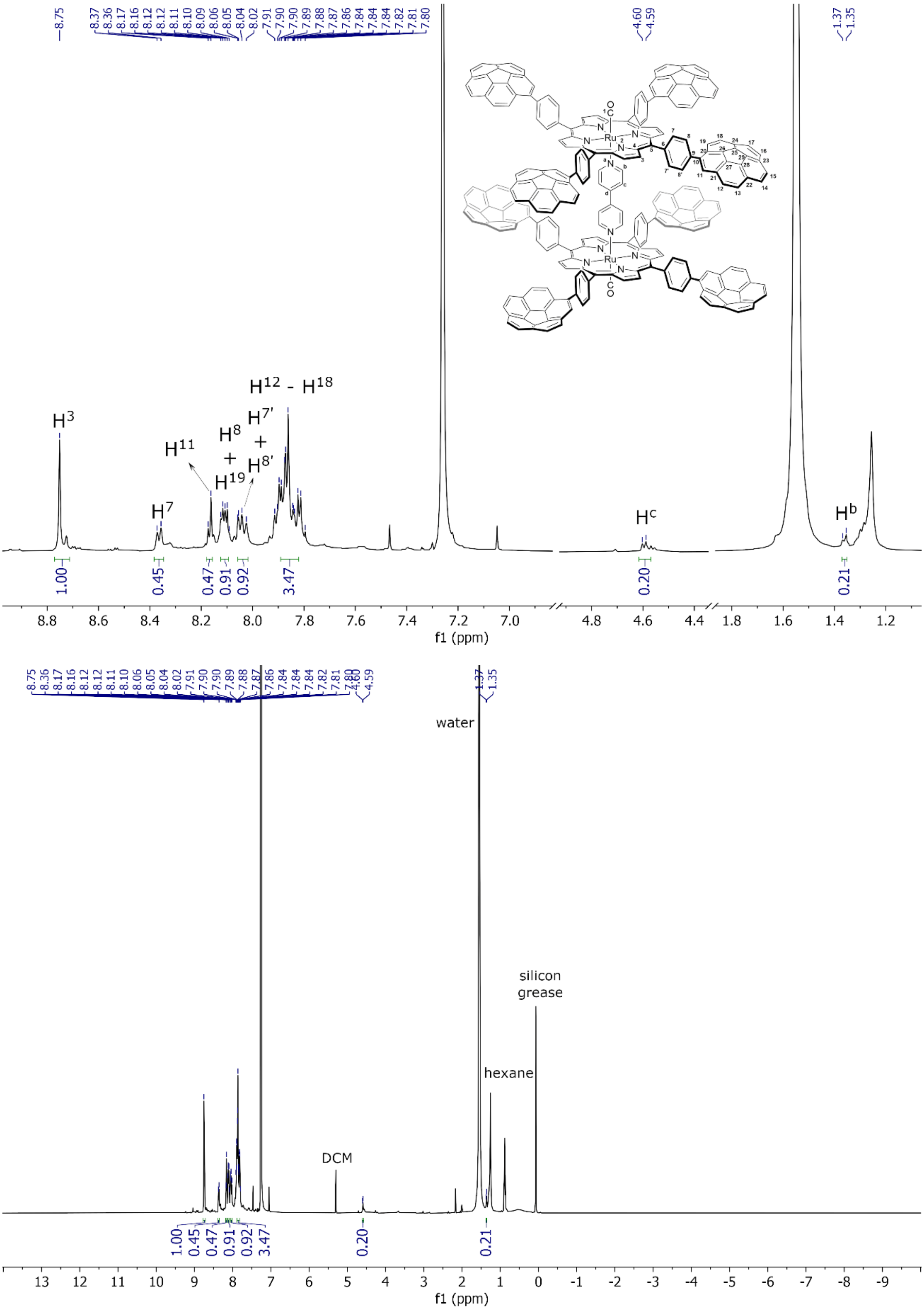

Figure S 75. ^{1}H NMR spectrum of compound **(RuP-cor)$_2$·bpy** (500 MHz, $CDCl_3$); selected regions (above) and full spectrum (below).

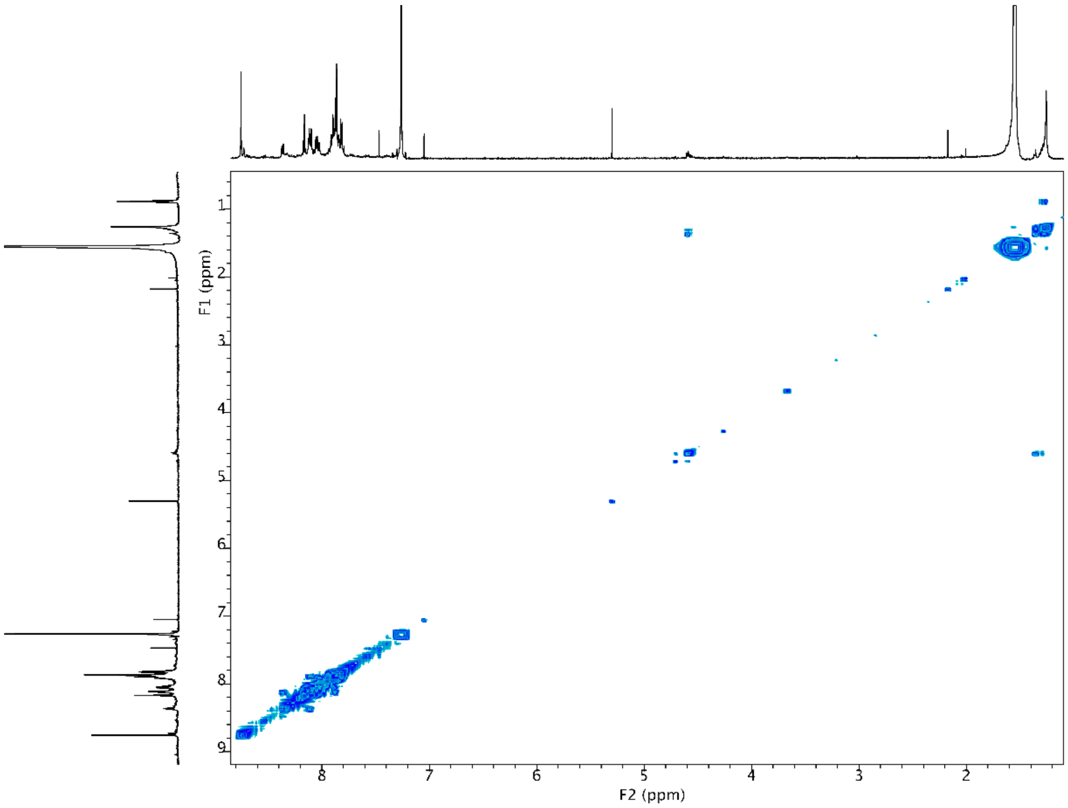

Figure S 76. ^{1}H-^{1}H gCOSY spectrum of compound **(RuP-cor)$_2$·bpy** (500 MHz, $CDCl_3$).

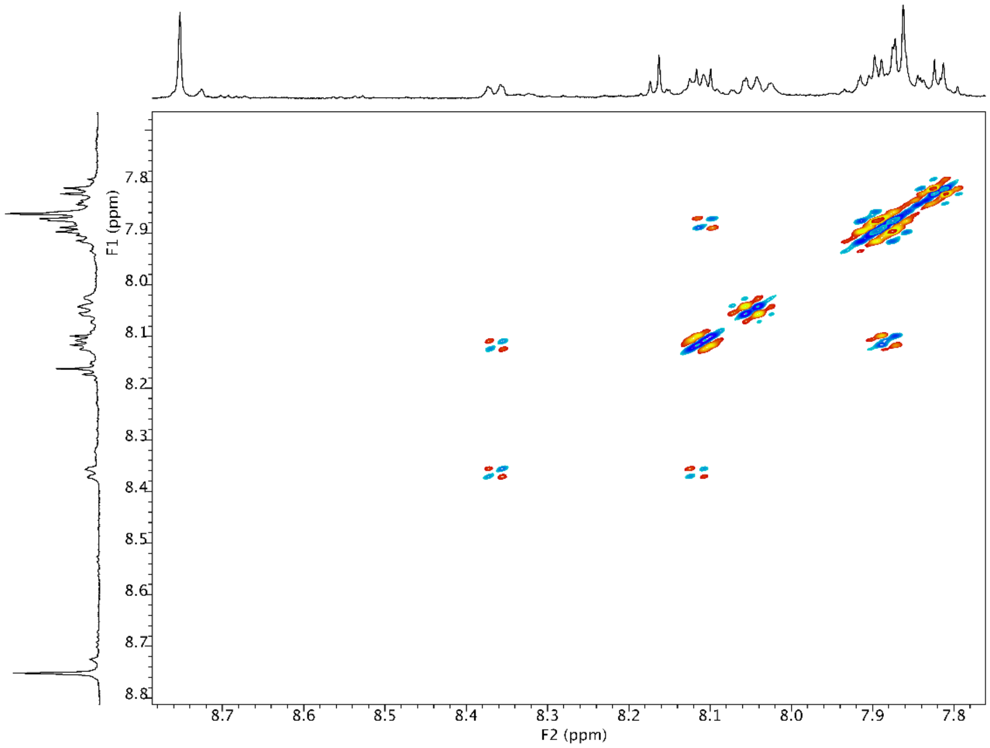

Figure S 77. ^{1}H-^{1}H gDQFCOSY spectrum of compound **(RuP-cor)$_2$·bpy** (500 MHz, $CDCl_3$).

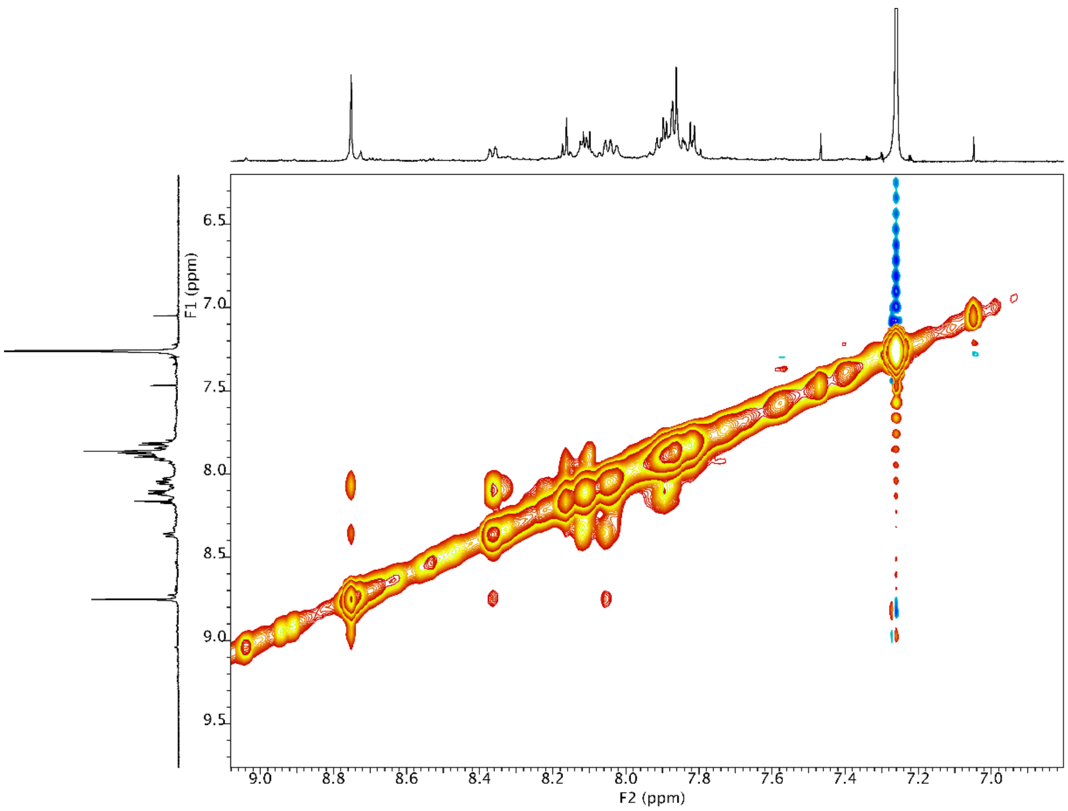

Figure S 78. ^{1}H-^{1}H ROESYAD spectrum of compound **(RuP-cor)$_2$·bpy** (500 MHz, $CDCl_3$).

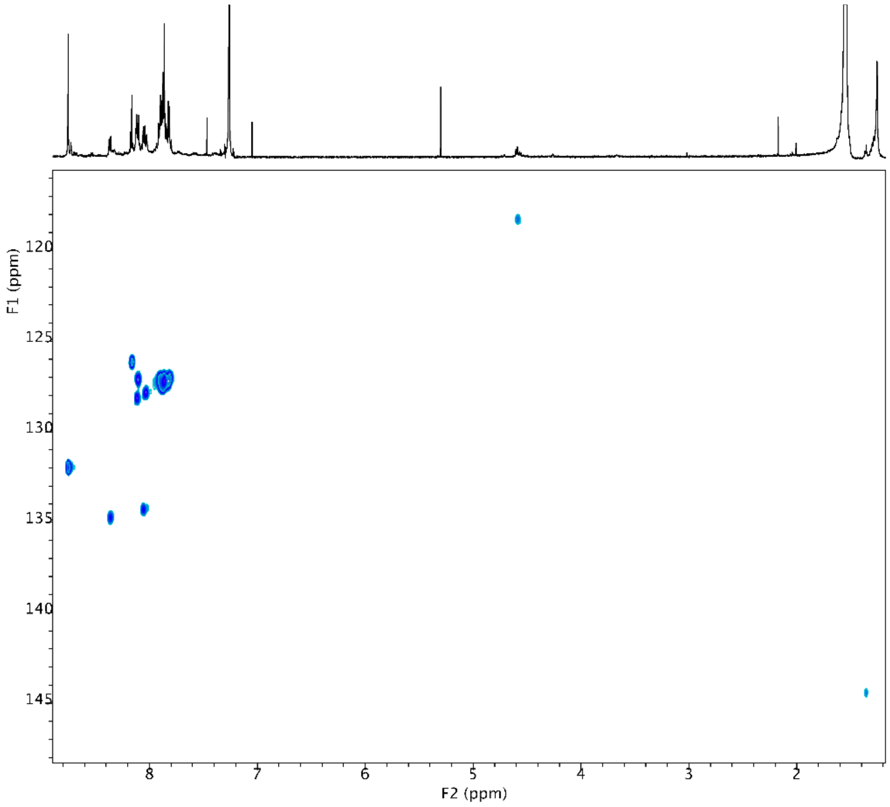

Figure S 79. ^{1}H-^{13}C gc2hsqc spectrum of compound **(RuP-cor)$_2$·bpy** (500 MHz, $CDCl_3$).

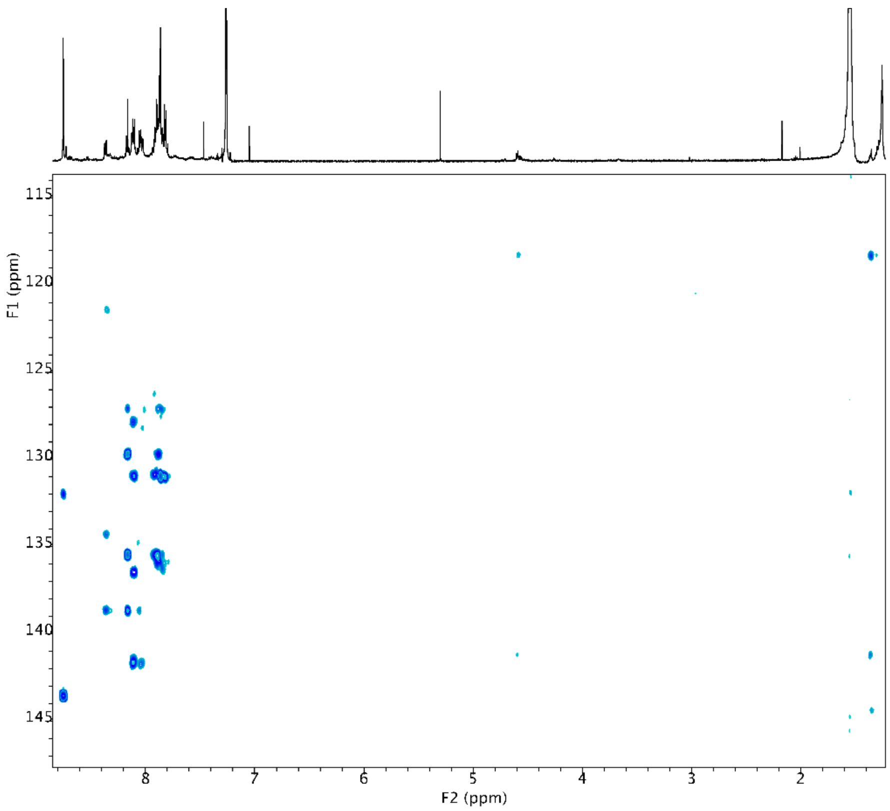

Figure S 80. ^{1}H-^{13}C gc2hmbc spectrum of compound **(RuP-cor)$_2$·bpy** (500 MHz, $CDCl_3$).

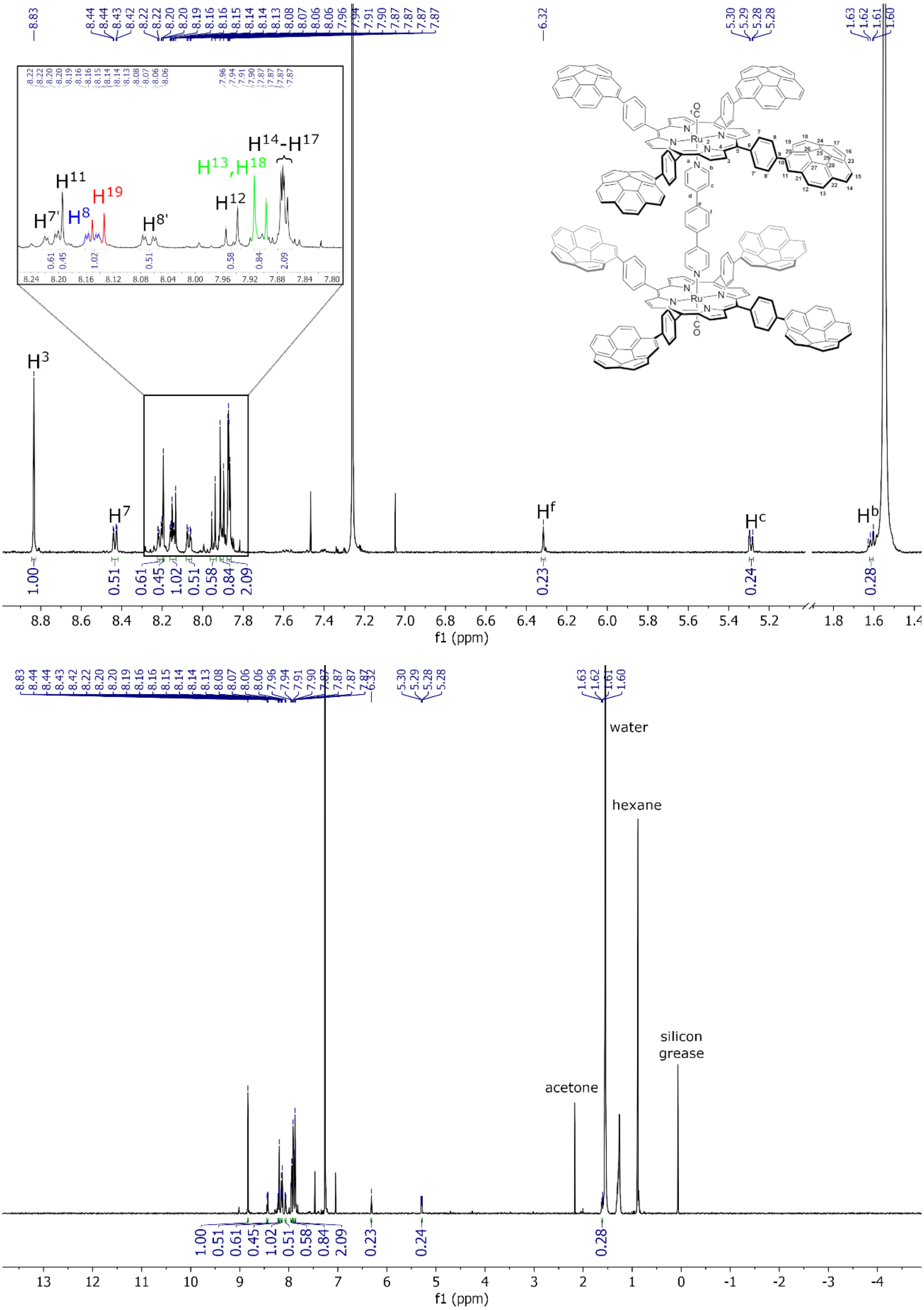

Figure S 81. ^{1}H NMR spectrum of compound **(RuP-cor)$_2$·dpyb** (500 MHz, $CDCl_3$); selected regions (above) and full spectrum (below). Some signals are depicted in different colors for clarity purposes.

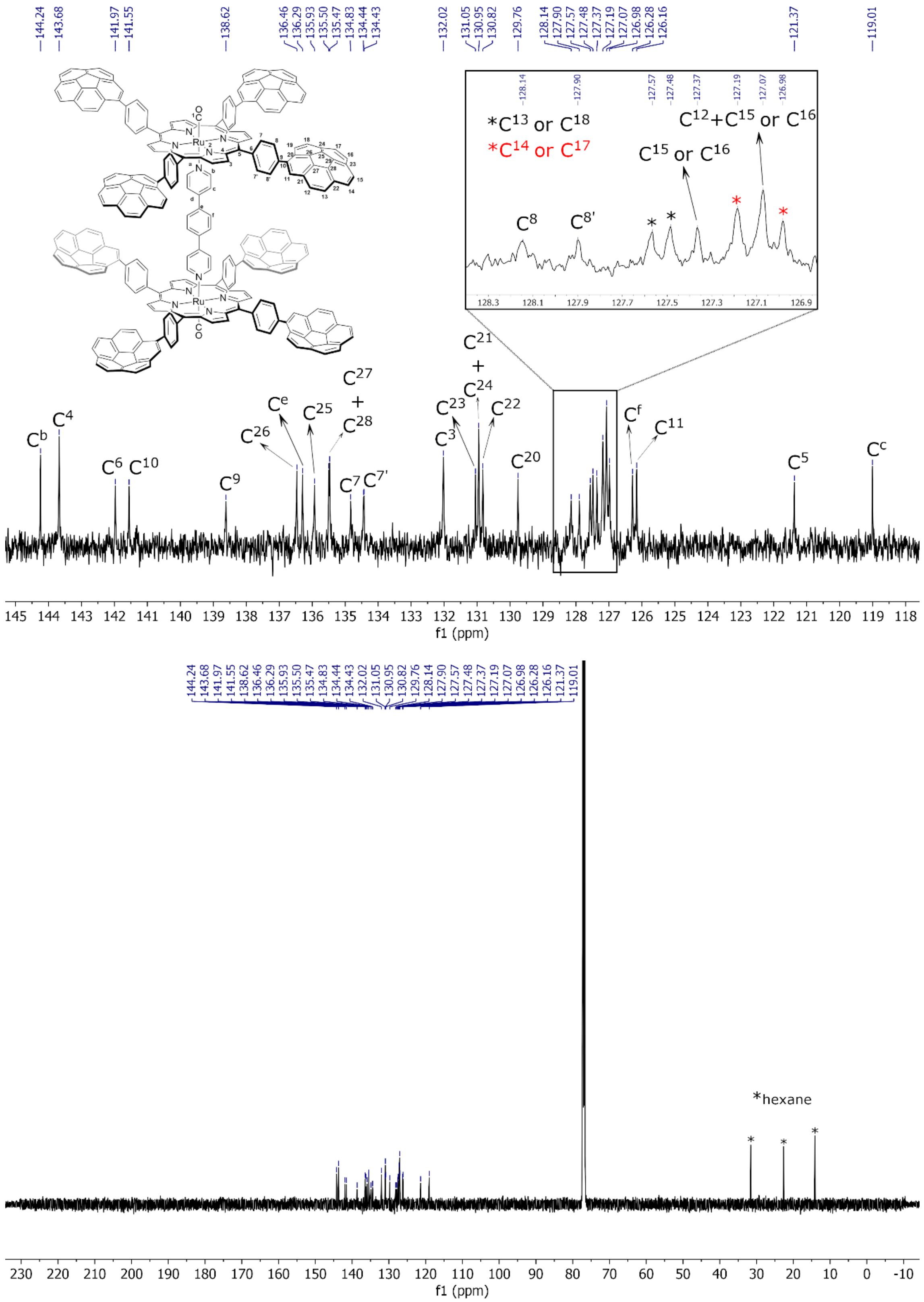

Figure S 82. $^{13}C\{^{1}H\}$ NMR spectrum of compound **(RuP-cor)₂·dpyb** (126 MHz, $CDCl_3$); selected regions (above) and full spectrum (below). Some signals are depicted in different colors for clarity purposes.

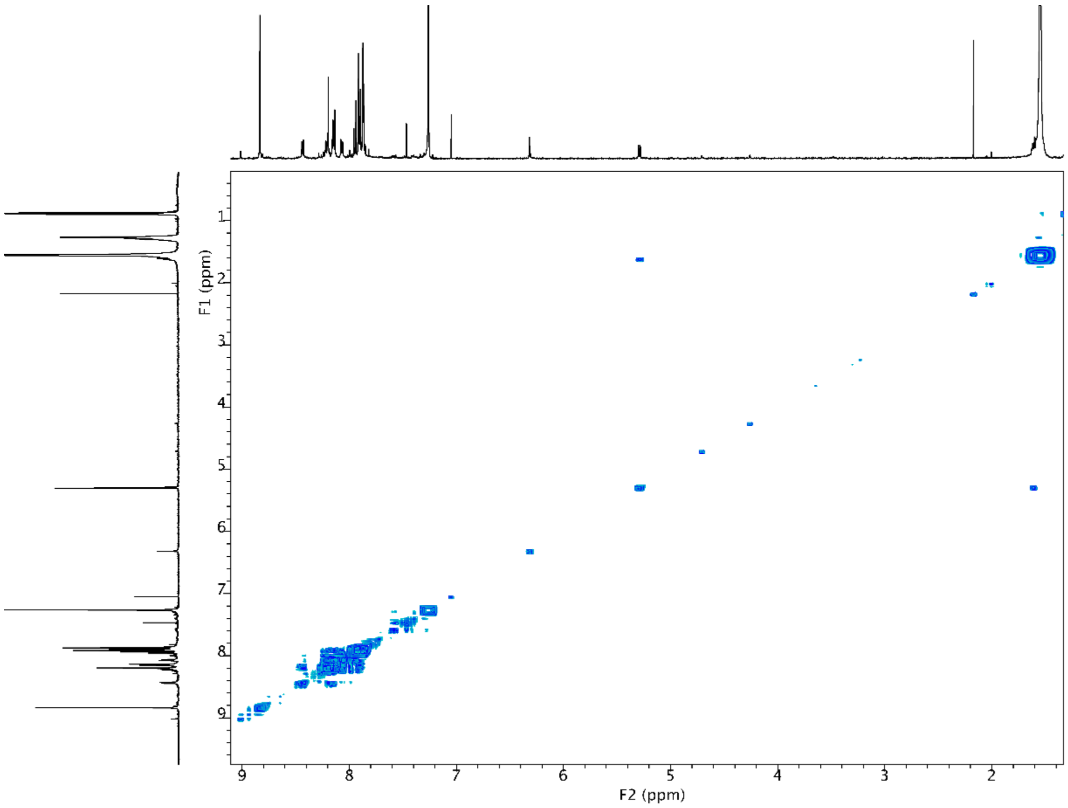

Figure S 83. ^1H-^1H gCOSY spectrum of compound **(RuP-cor)$_2$·dpyb** (500 MHz, $CDCl_3$).

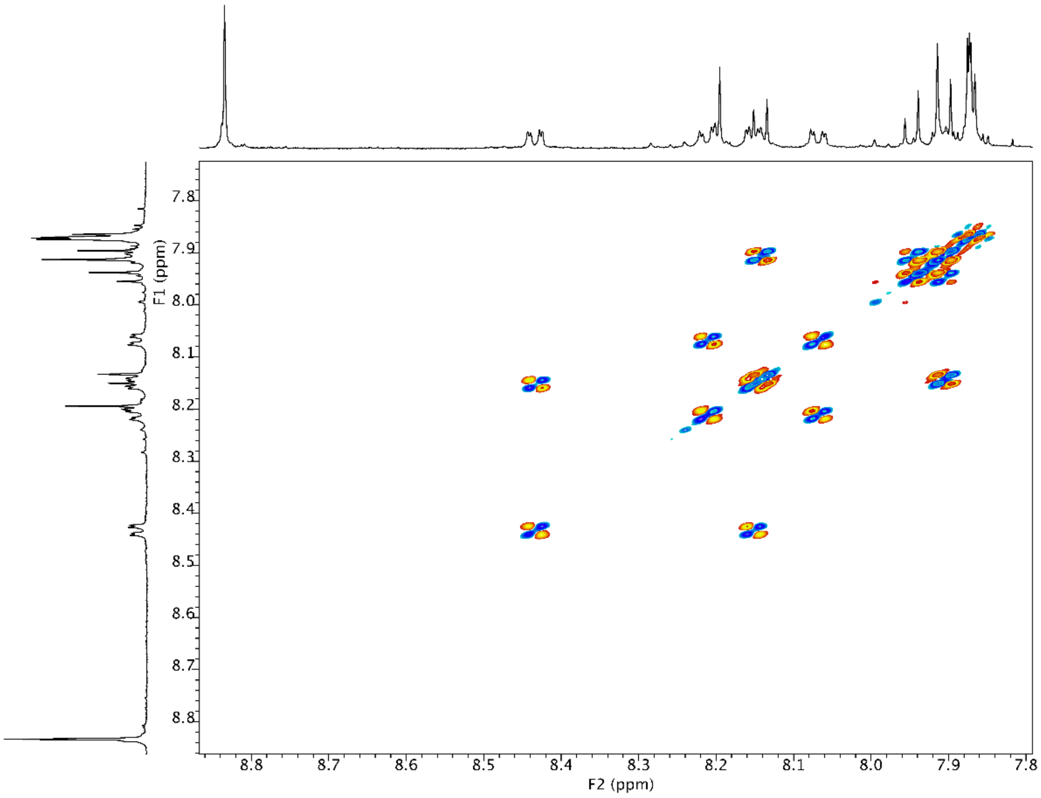

Figure S 84. ^1H-^1H gDQFCOSY spectrum of compound **(RuP-cor)$_2$·dpyb** (500 MHz, $CDCl_3$).

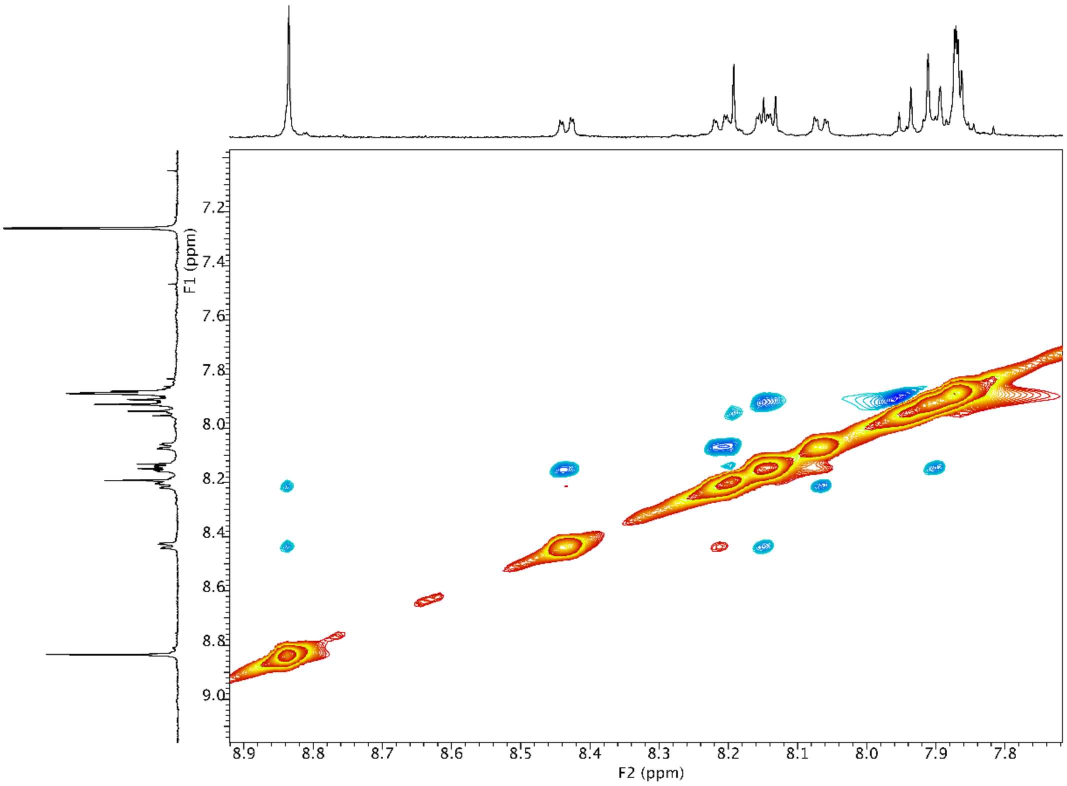

Figure S 85. ^{1}H-^{1}H ROESYAD spectrum of compound **(RuP-cor)$_2$·dpyb** (500 MHz, $CDCl_3$).

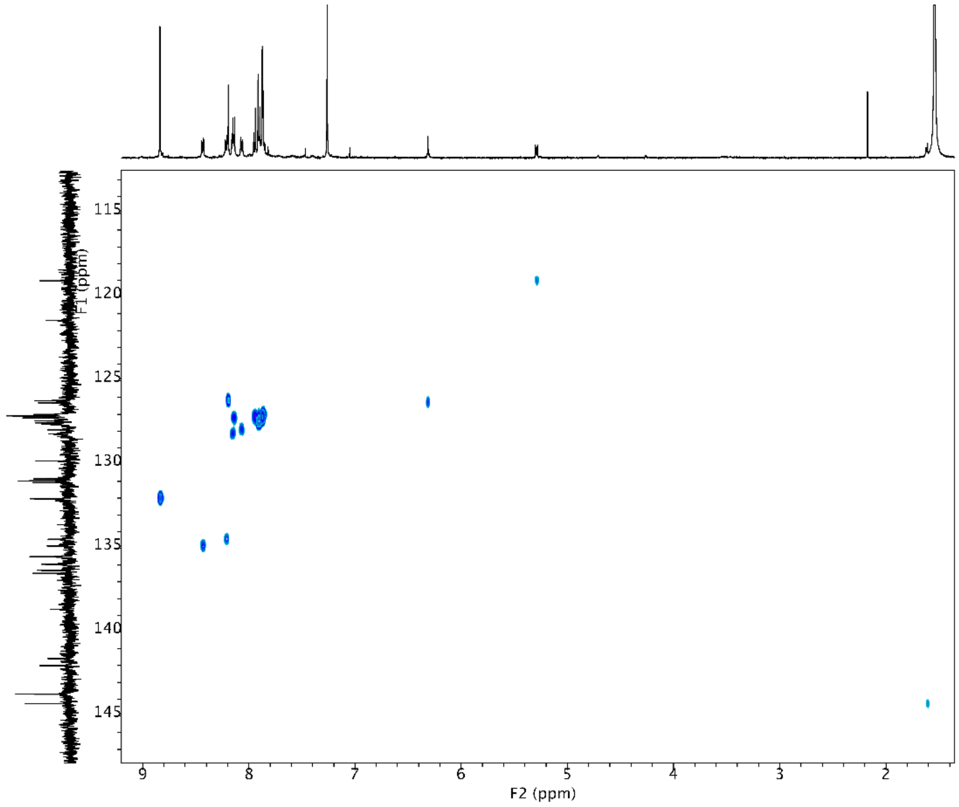

Figure S 86. ^{1}H-^{13}C gc2hsqc spectrum of compound **(RuP-cor)$_2$·dpyb** (500 MHz, $CDCl_3$).

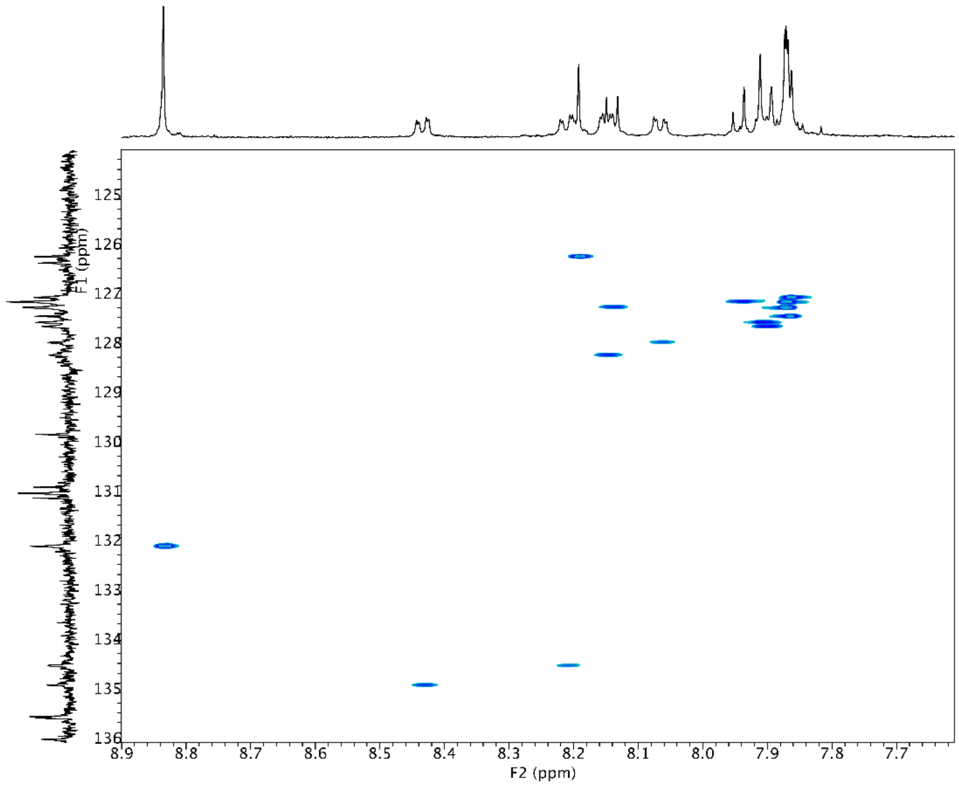

Figure S 87. ^{1}H-^{13}C selective gc2hsqc spectrum of compound **(RuP-cor)$_2$·dpyb** (500 MHz, $CDCl_3$).

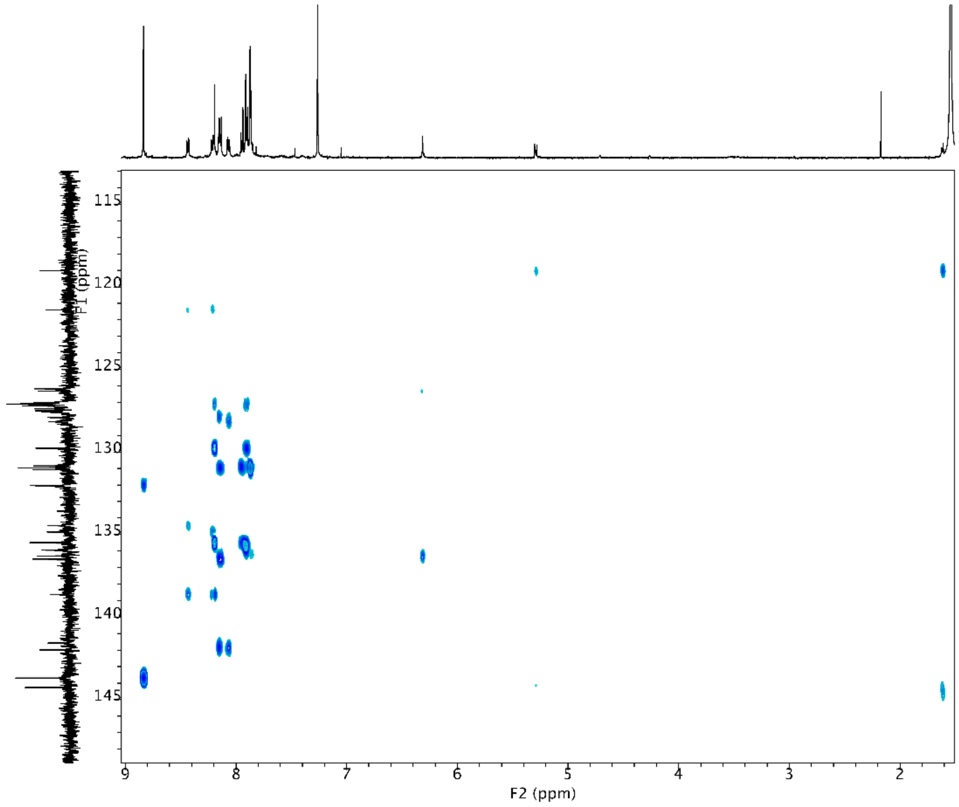

Figure S 88. ^{1}H-^{13}C gc2hmb spectrum of compound **(RuP-cor)$_2$·dpyb** (500 MHz, $CDCl_3$).

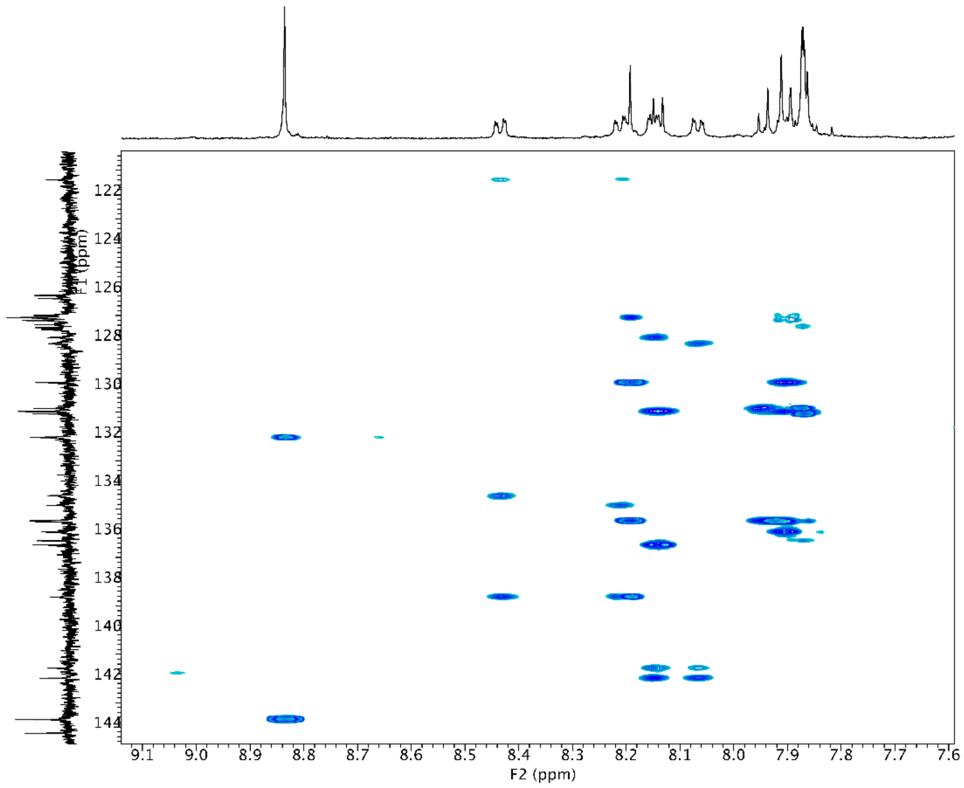

Figure S 89. ^{1}H-^{13}C selective gc2hmbc spectrum of compound **(RuP-cor)$_2$·dpyb** (500 MHz, $CDCl_3$).

High Resolution Mass Spectrometry

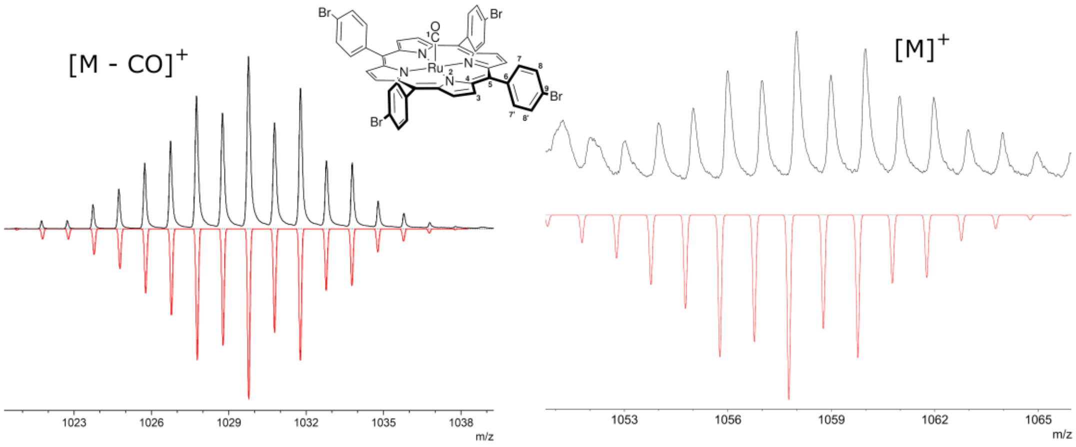

Figure S 90. HRMS (MALDI-TOF +, DCTB) of peaks of interest found for compound **RuP-Br**.

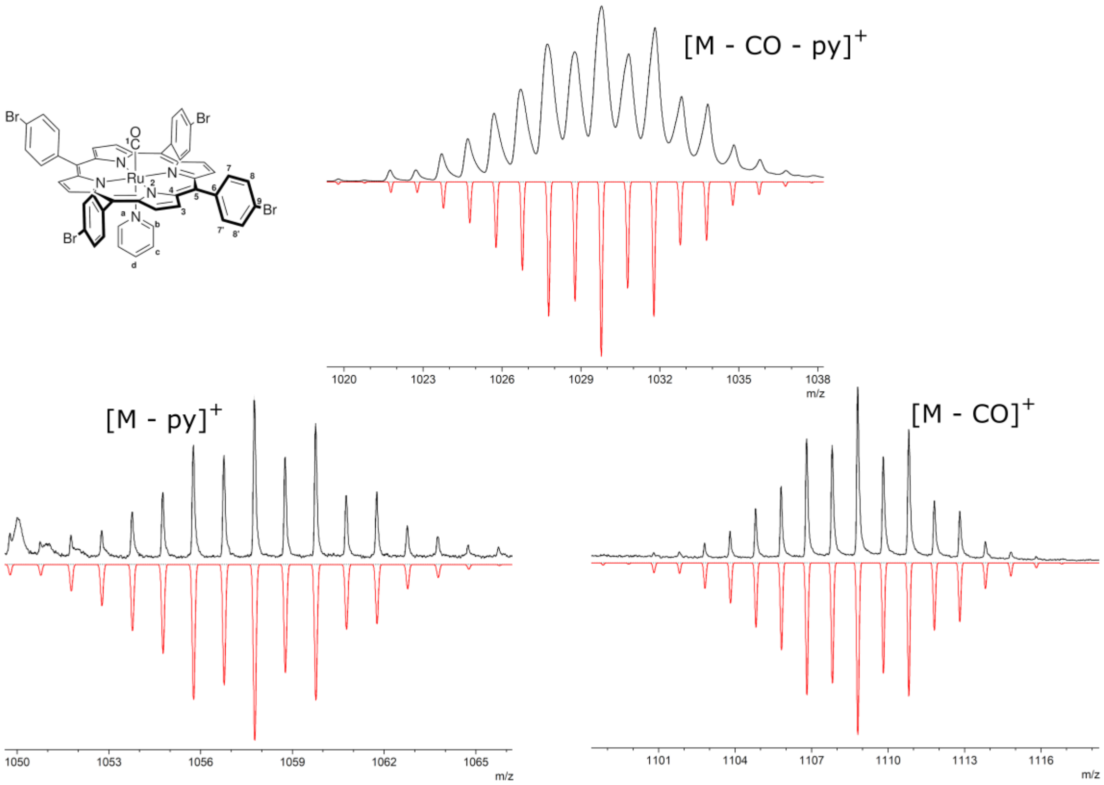

Figure S 91. HRMS (MALDI-TOF +, DCTB) of peaks of interest found for compound **RuP-Br·py**.

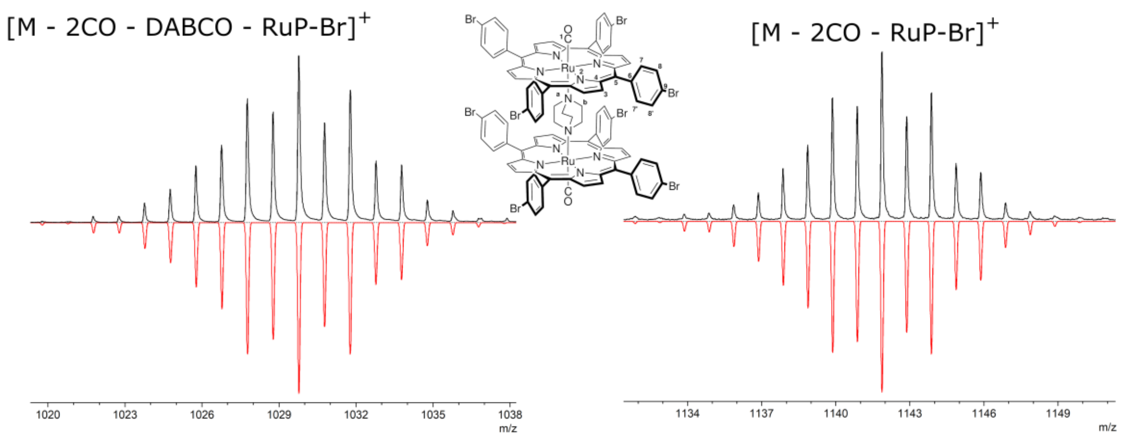

Figure S 92. HRMS (MALDI-TOF +, DCTB) of peaks of interest found for compound **(RuP-Br)$_2$·DABCO**.

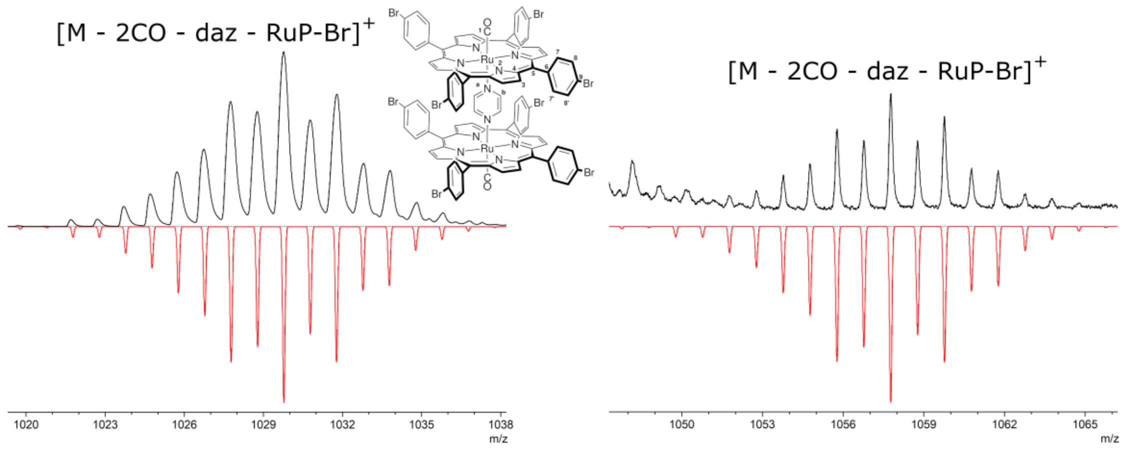

Figure S 93. HRMS (MALDI-TOF +, DCTB) of peaks of interest found for compound **(RuP-Br)$_2$·daz**.

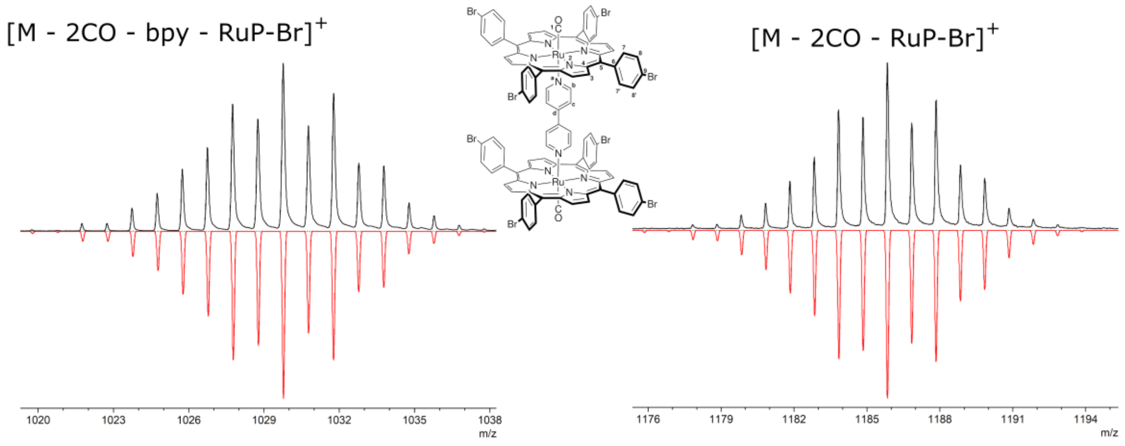

Figure S 94. HRMS (MALDI-TOF +, DCTB) of peaks of interest found for compound **(RuP-Br)$_2$·bpy**.

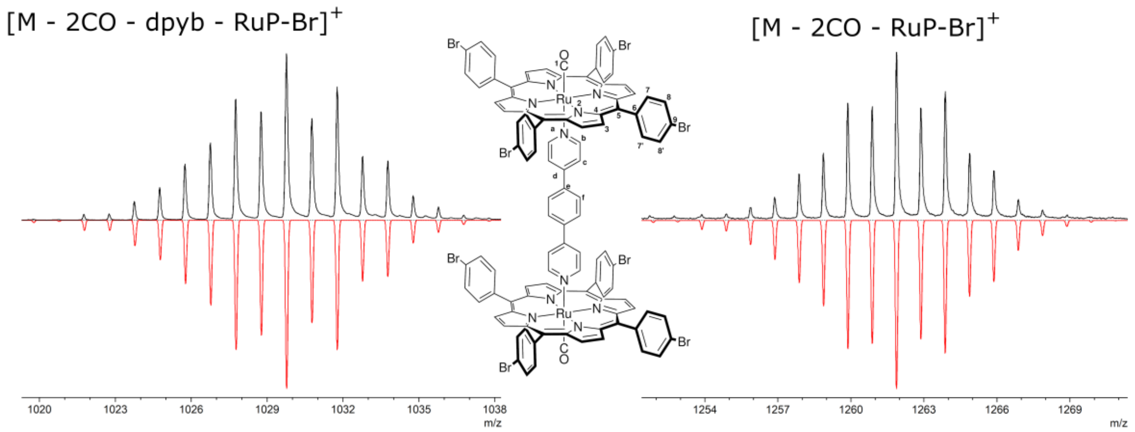

Figure S 95. HRMS (MALDI-TOF +, DCTB) of peaks of interest found for compound **(RuP-Br)$_2$·dpyb**.

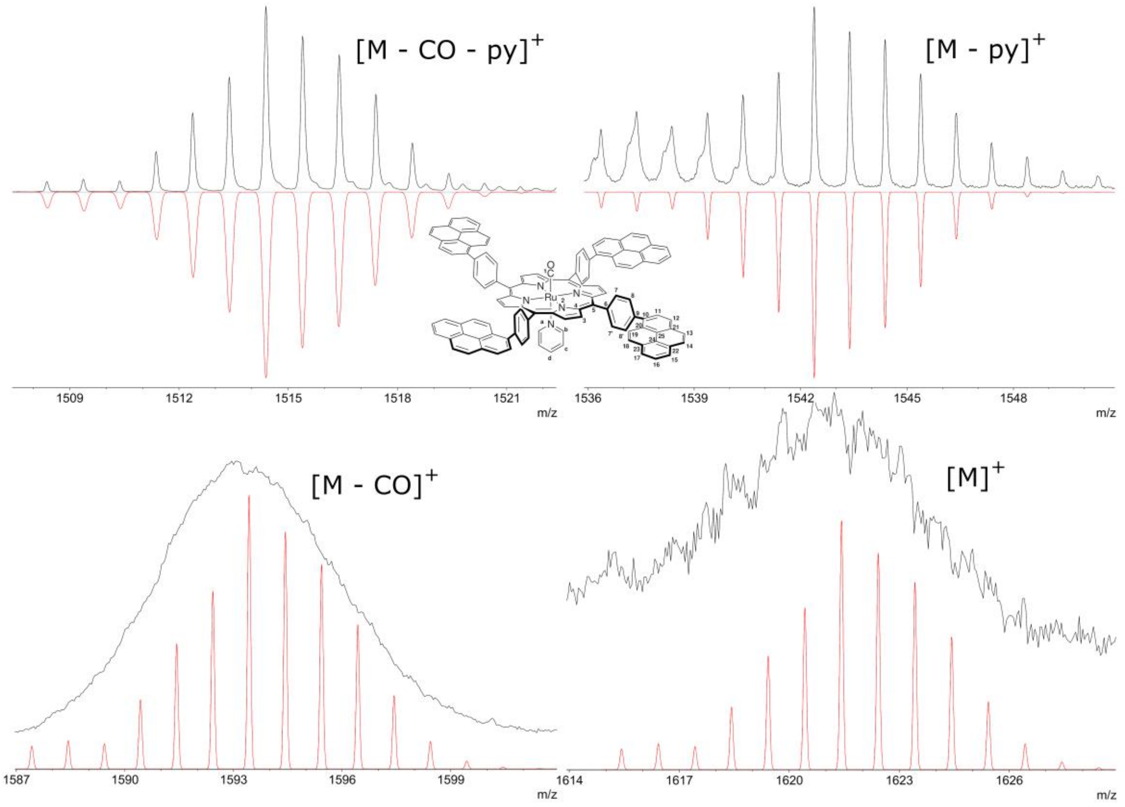

Figure S 96. HRMS and LRMS (MALDI-TOF +, DCTB) of peaks of interest found for compound **RuP-pyr·py**.

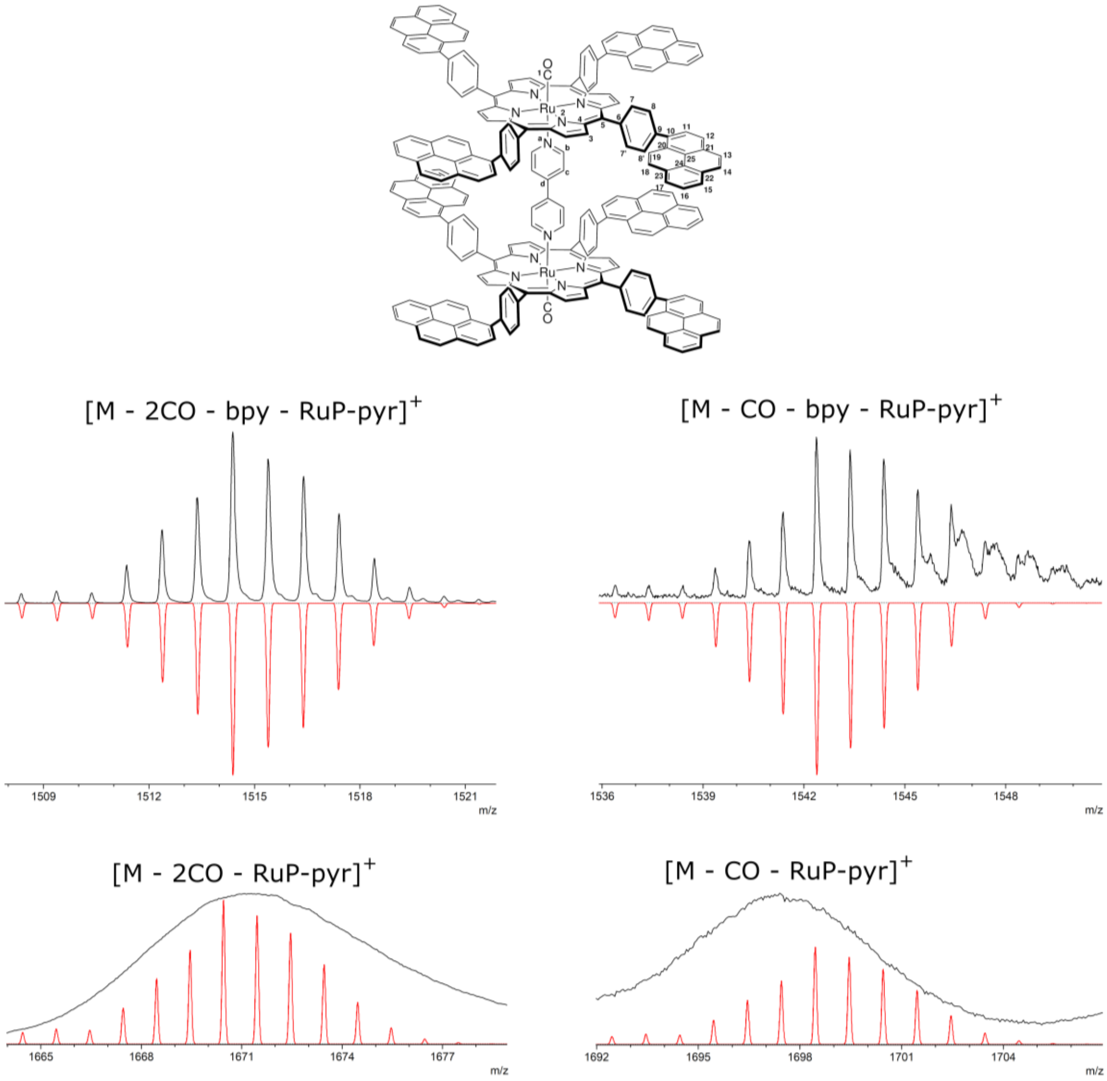

Figure S 97. HRMS and LRMS (MALDI-TOF +, DCTB) of peaks of interest found for compound **(RuP-pyr)$_2$·bpy**.

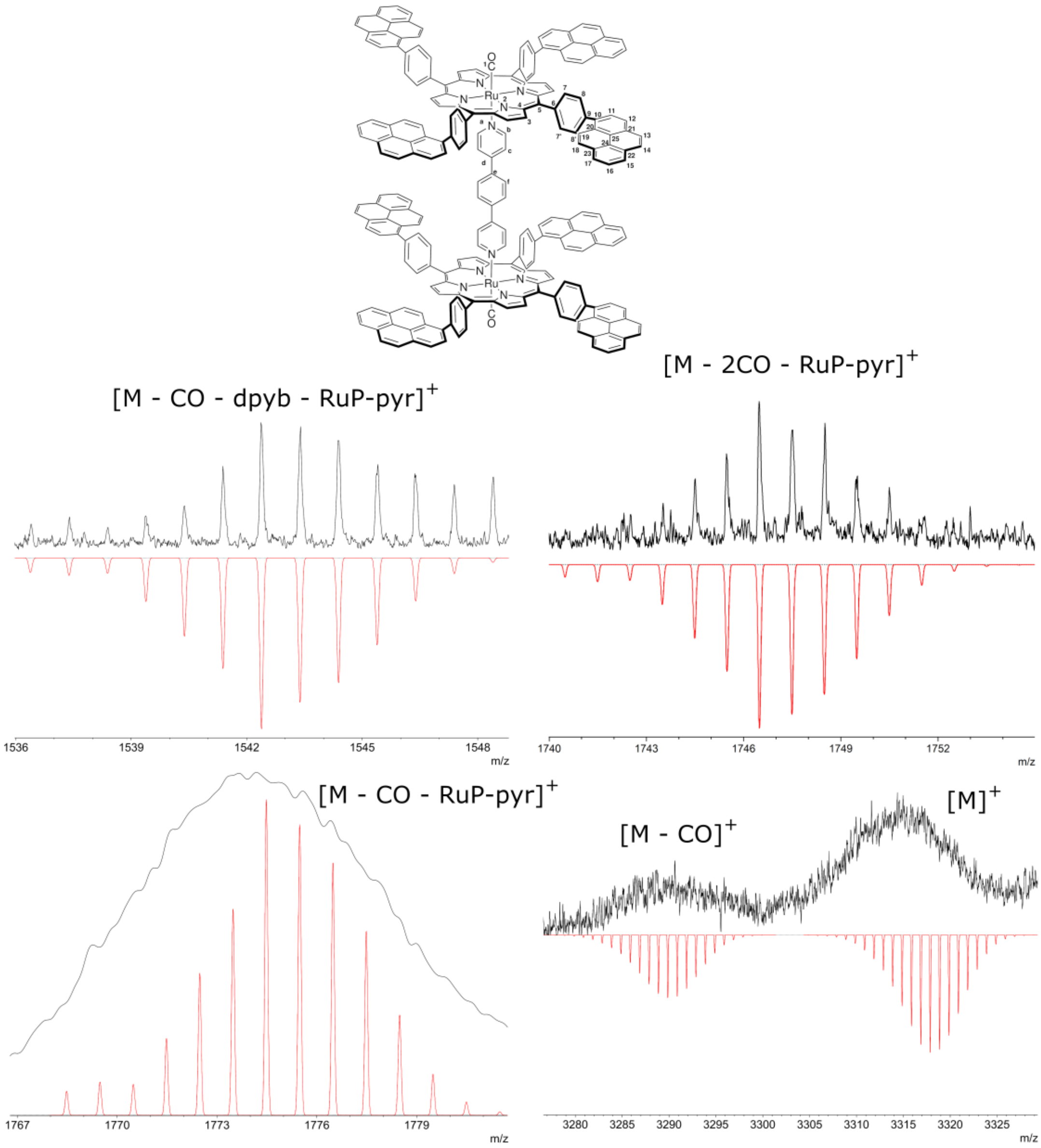

Figure S 98. HRMS and LRMS (MALDI-TOF +, DCTB) of peaks of interest found for compound **(RuP-pyr)$_2$·dpyb**.

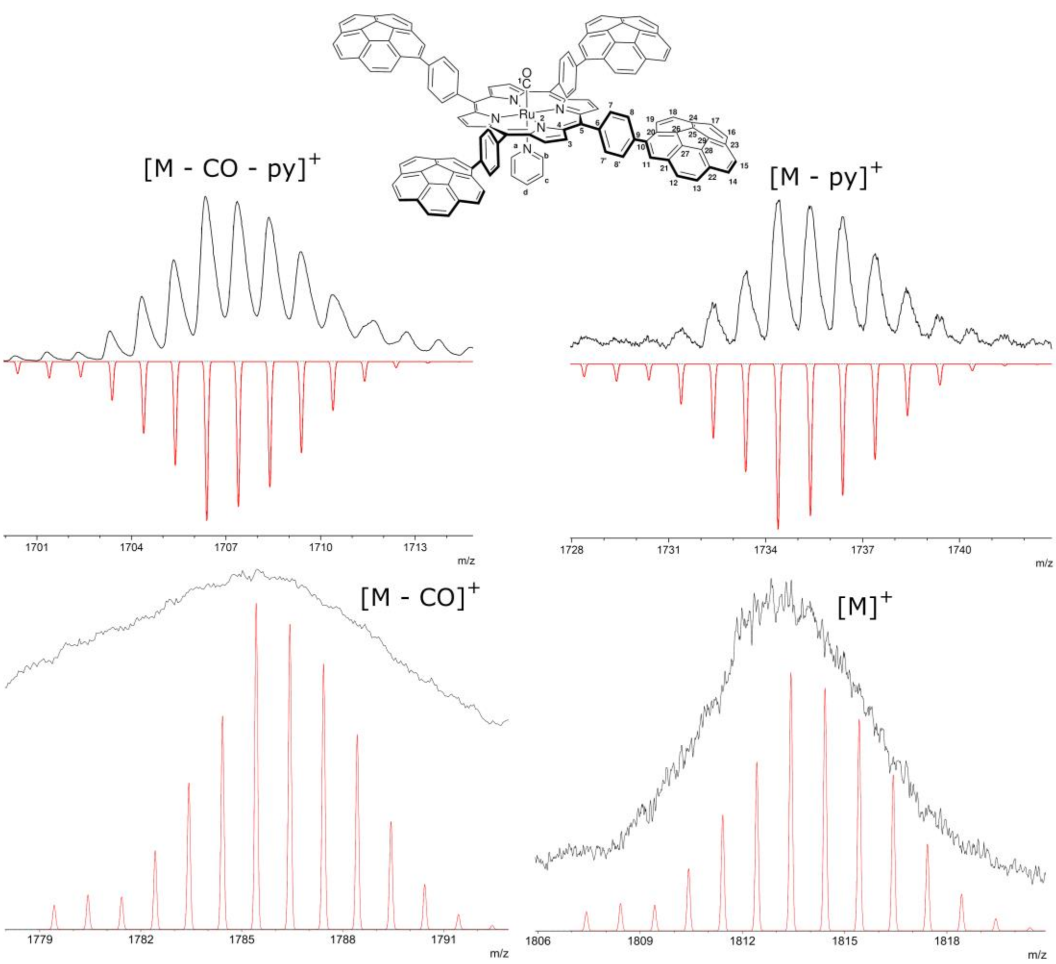

Figure S 99. HRMS and LRMS (MALDI-TOF +, DCTB) of peaks of interest found for compound **RuP-cor·py**.

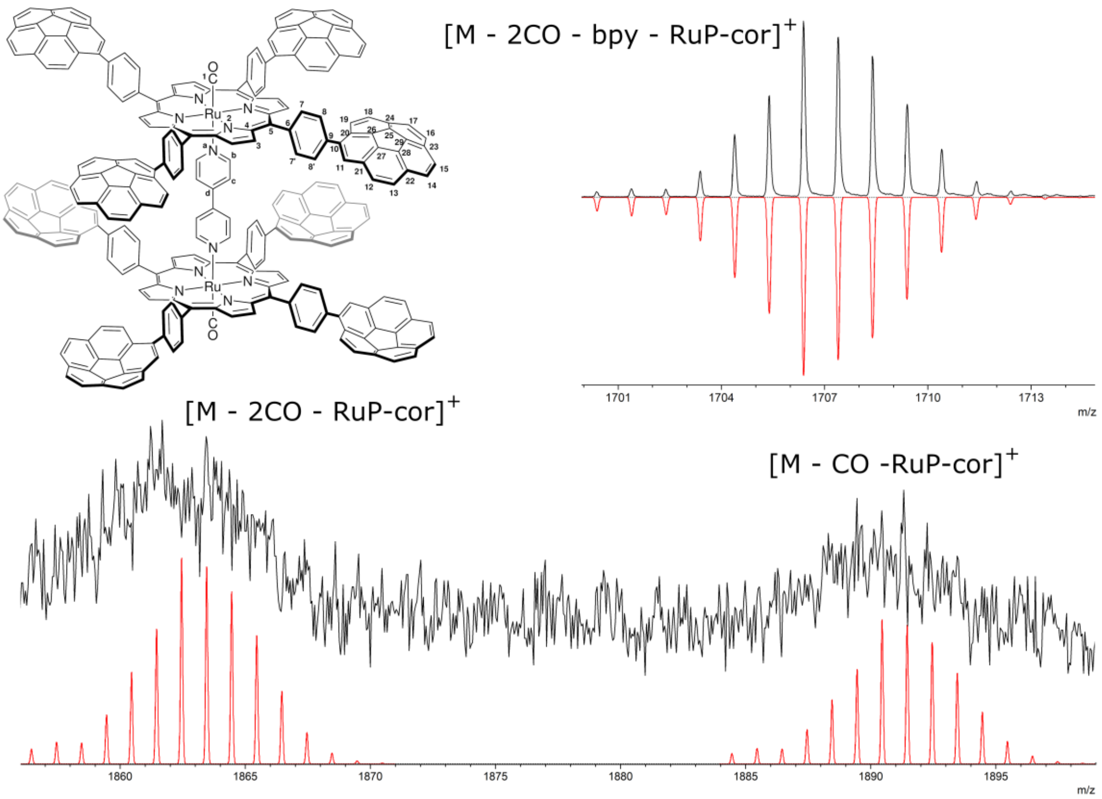

Figure S 100. HRMS and LRMS (MALDI-TOF +, DCTB) of peaks of interest found for compound (**RuP-cor**)$_2$·**bpy**.

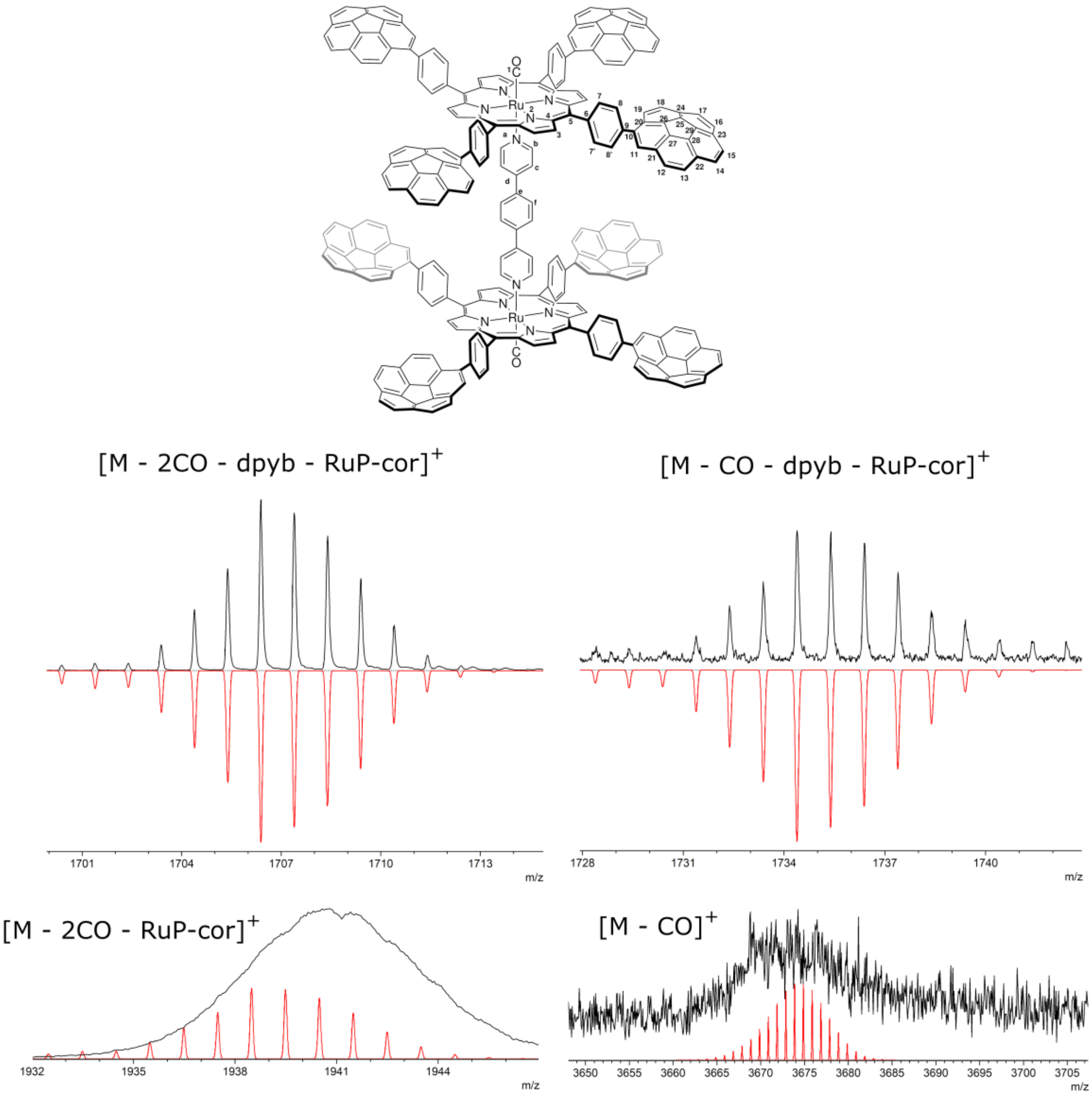

Figure S 101. HRMS and LRMS (MALDI-TOF +, DCTB) of peaks of interest found for compound **(RuP-cor)$_2$·dpyb**.

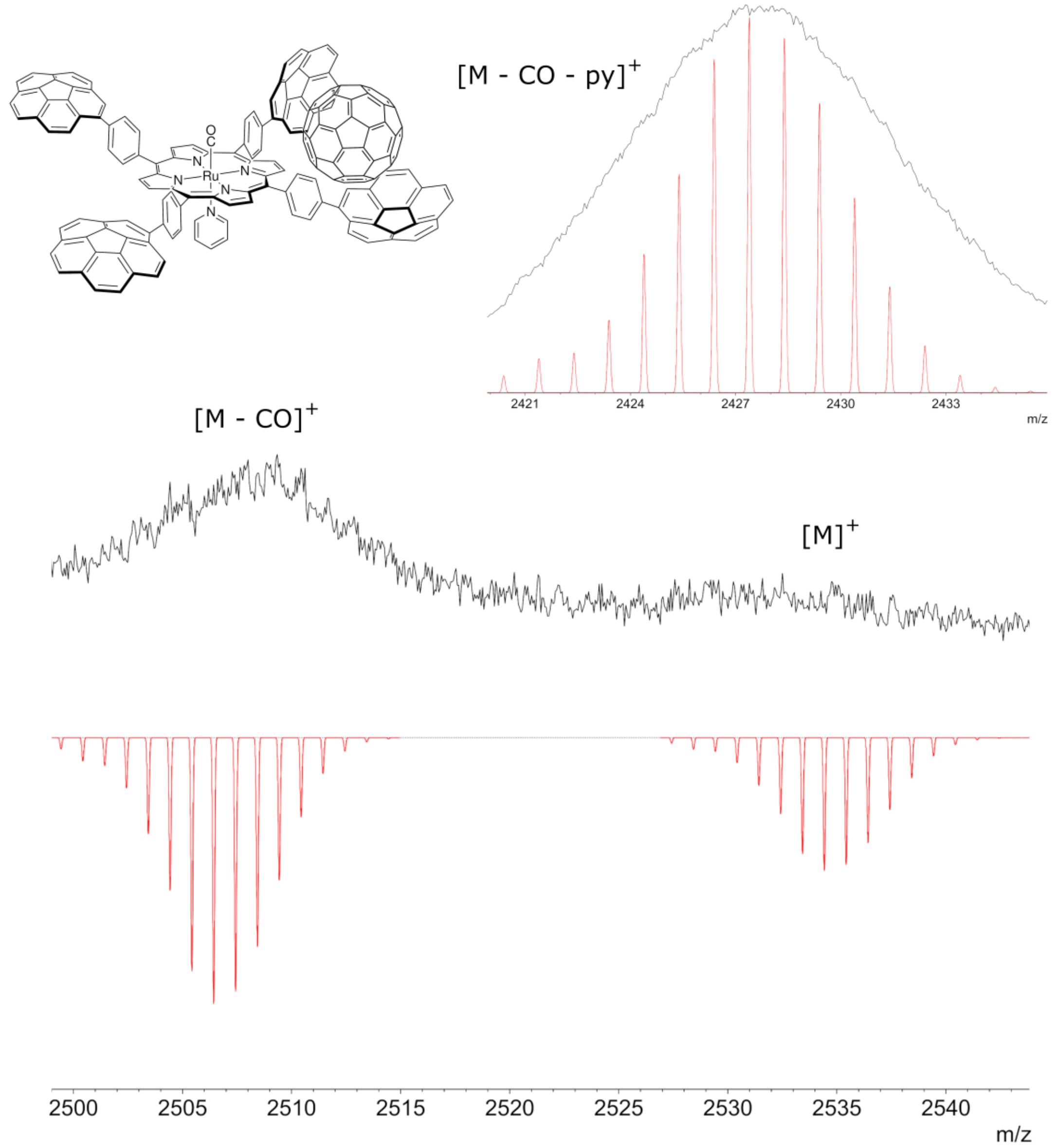

Figure S 102. LRMS (MALDI-TOF +, DCTB) of peaks of interest found for the adduct **C_{60}@RuP-cor·py**.

FT-IR spectra

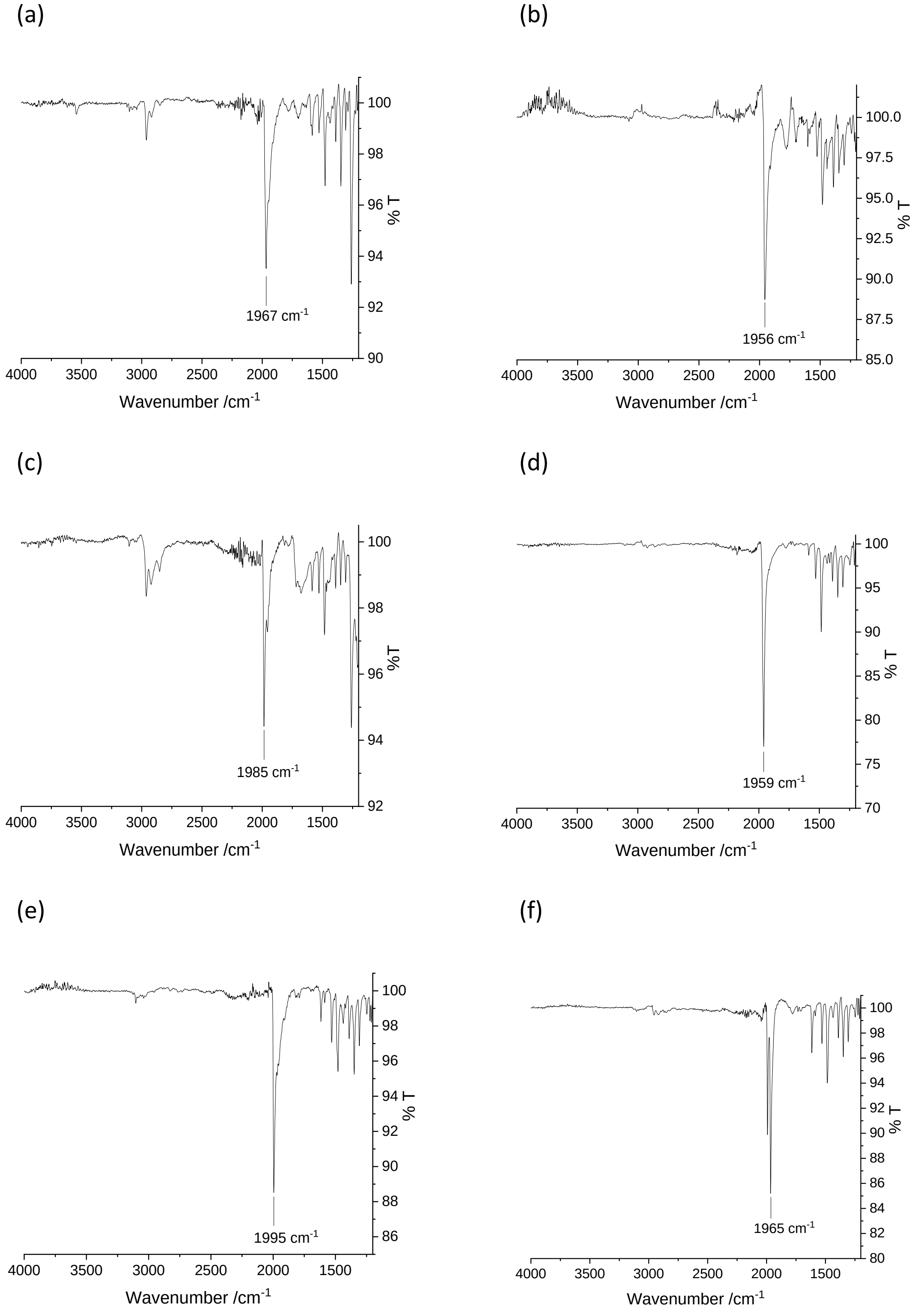

Figure S 103. FT-IR spectra of compounds (a) **RuP-Br**, (b) **RuP-Br·py**, (c) **$(RuP\text{-}Br)_2$·DABCO**, (d) **$(RuP\text{-}Br)_2$·daz**, (e) **$(RuP\text{-}Br)_2$·bpy** and (f) **$(RuP\text{-}Br)_2$·dpyb**.

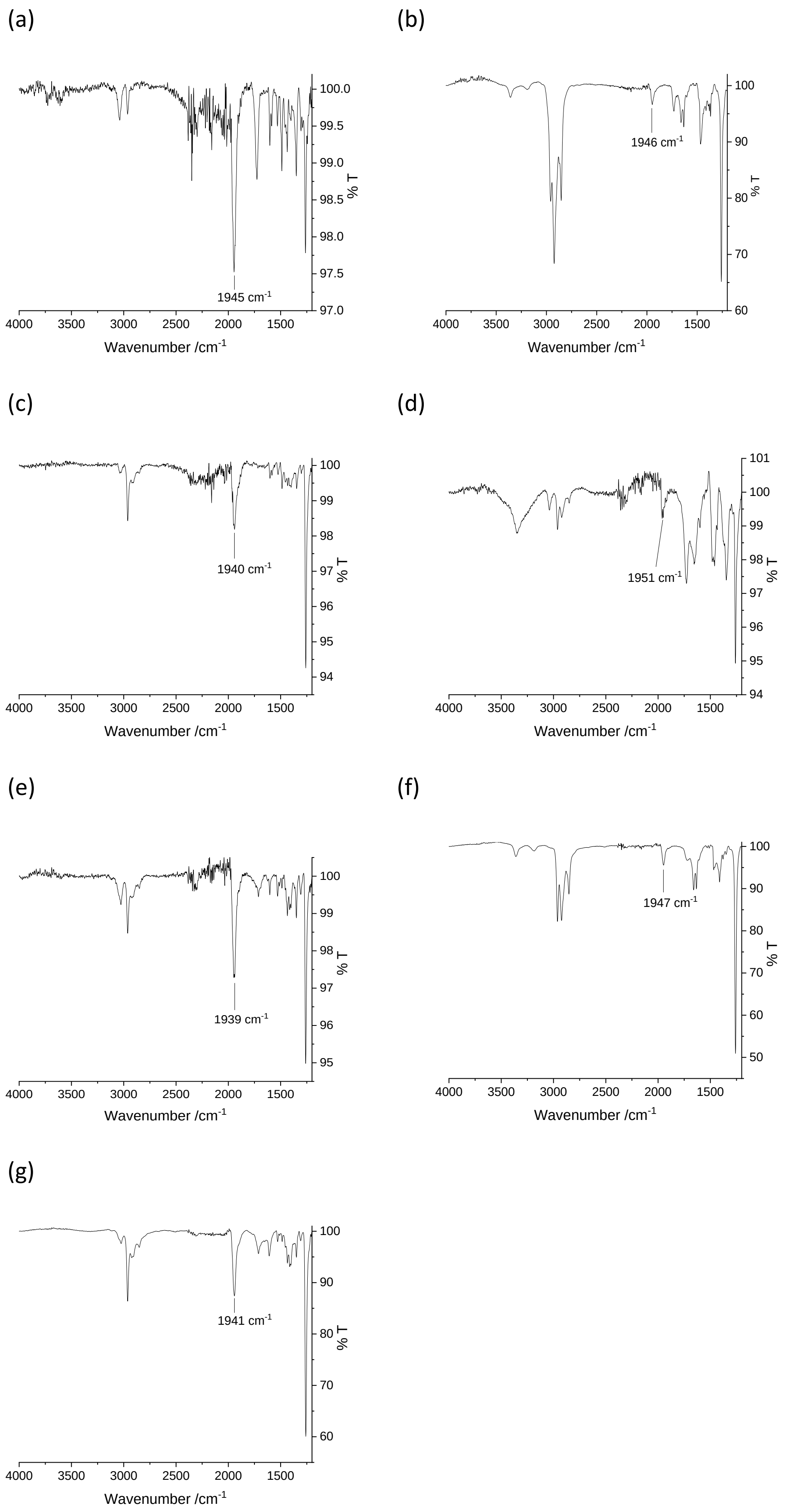

Figure S 104. FT-IR spectra of compounds (a) **RuP-pyr·py**, (b) **(RuP-pyr)$_2$·DABCO**, (c) **(RuP-pyr)$_2$·bpy**, (d) **(RuP-pyr)$_2$·dpyb**, (e) **RuP-cor·py**, (f) **(RuP-cor)$_2$·bpy** and (g) **(RuP-cor)$_2$·dpyb**.

UV-Vis absorption and emission spectra

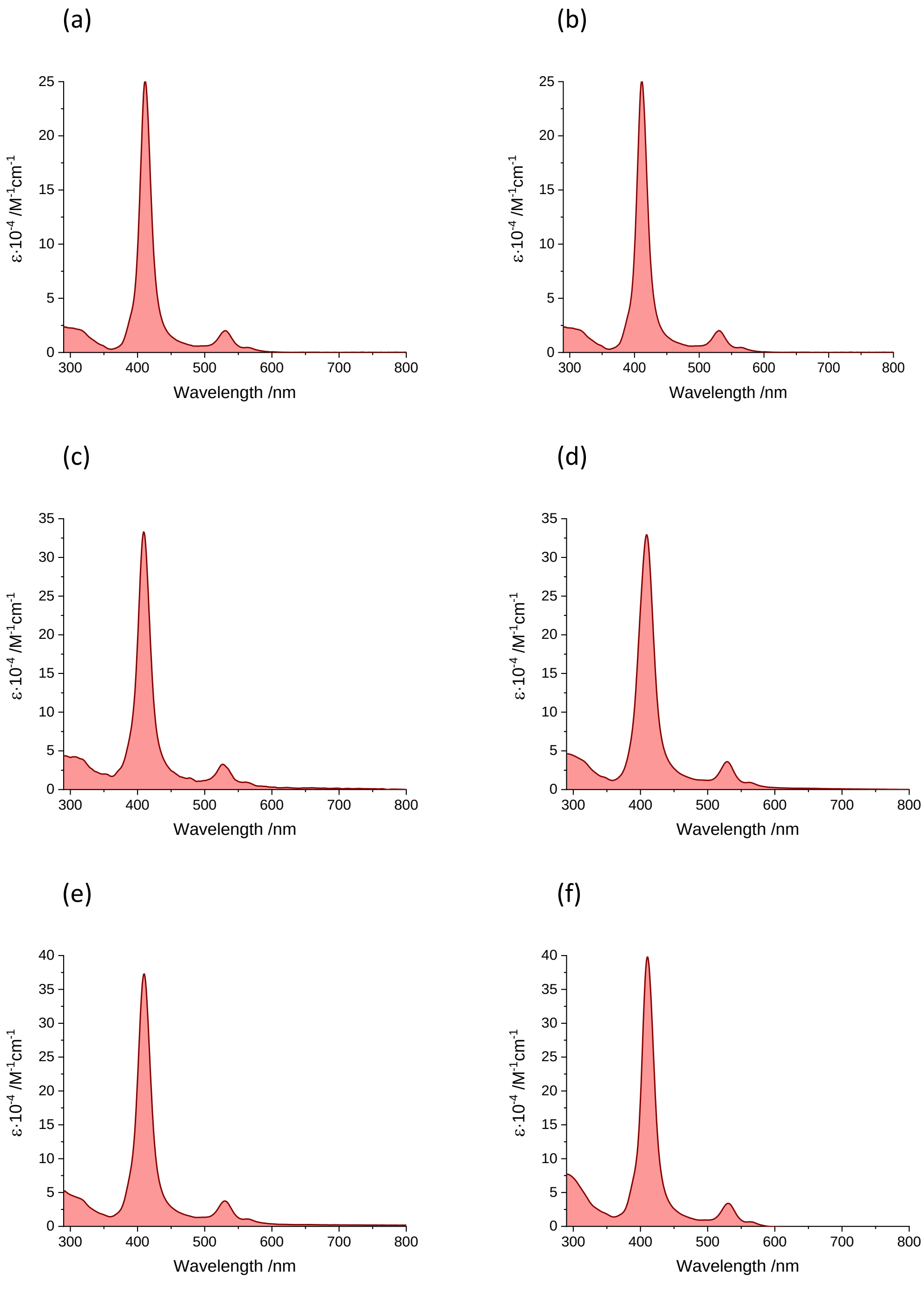

Figure S 105. UV-Vis absorption spectra of complexes (a) **RuP-Br** ($5 \cdot 10^{-6}$ M), (b) **RuP-Br·py** ($5 \cdot 10^{-6}$ M), (c) **(RuP-Br)$_2$·DABCO** ($5 \cdot 10^{-6}$ M), (d) **(RuP-Br)$_2$·daz** ($5 \cdot 10^{-6}$ M), (e) **(RuP-Br)$_2$·bpy** ($5 \cdot 10^{-6}$ M) and (f) **(RuP-Br)$_2$·dpyb** ($5 \cdot 10^{-6}$ M) in DCM.

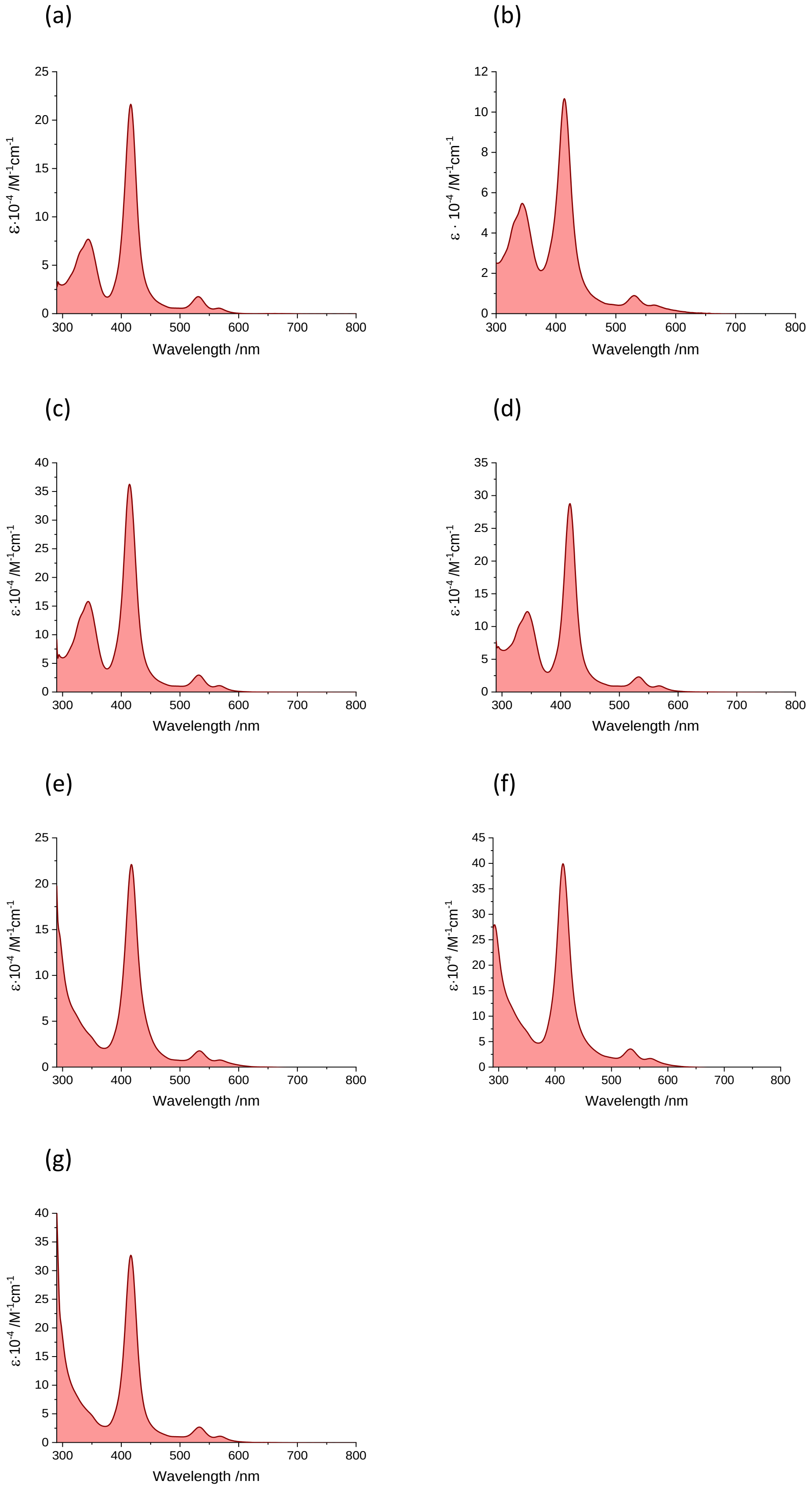

Figure S 106. UV-Vis absorption spectra of complexes (a) **RuP-pyr·py** ($5 \cdot 10^{-6}$ M), (b) **(RuP-pyr)$_2$·DABCO** ($5 \cdot 10^{-6}$ M, (c) **(RuP-pyr)$_2$·bpy** ($5 \cdot 10^{-6}$ M), (d) **(RuP-pyr)$_2$·dpyb** ($5 \cdot 10^{-6}$ M), (e) **RuP-cor·py** ($5 \cdot 10^{-6}$ M), (f) **(RuP-cor)$_2$·bpy** ($5 \cdot 10^{-6}$ M) and (g) **(RuP-cor)$_2$·dpyb** ($5 \cdot 10^{-6}$ M) in DCM.

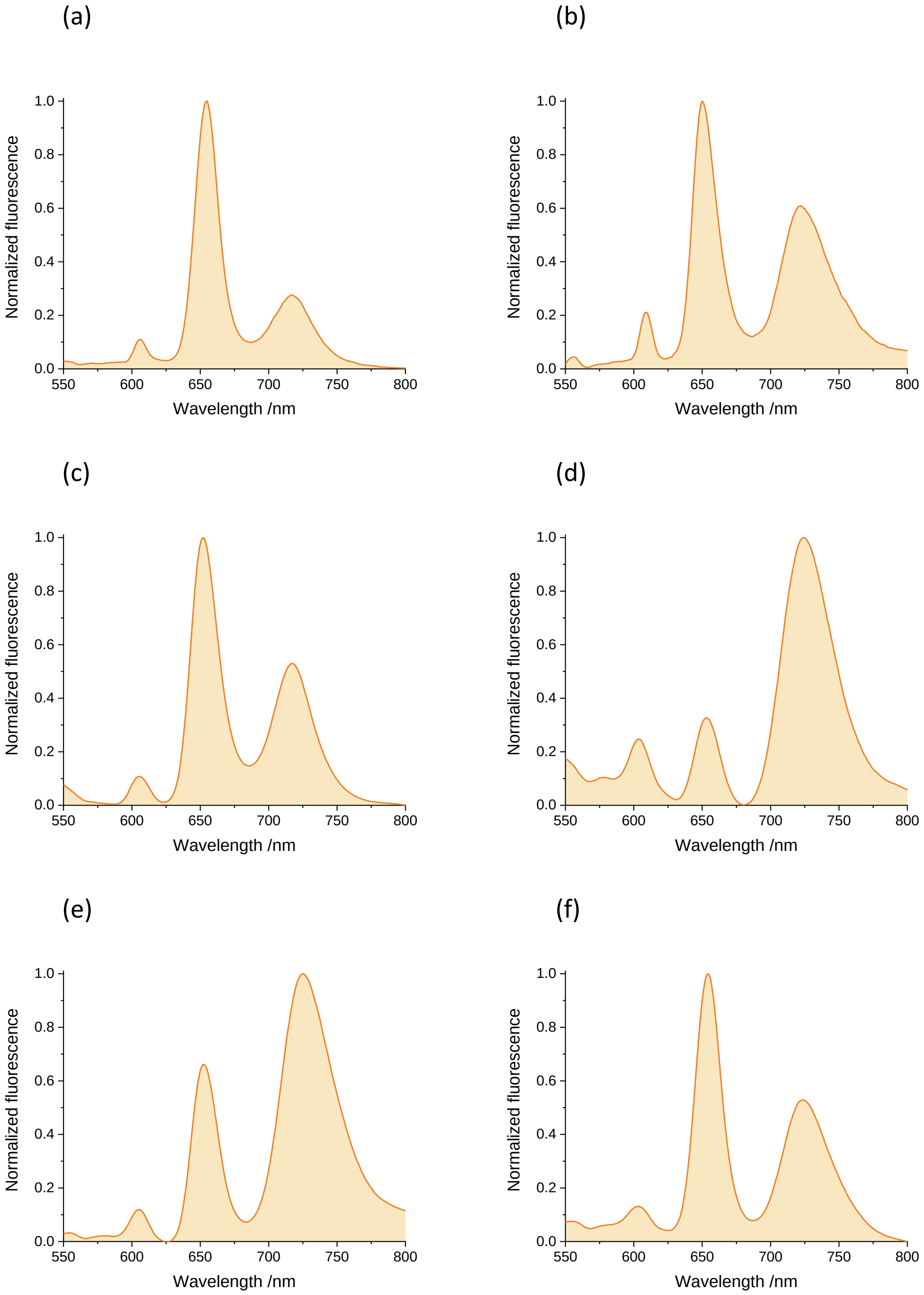

Figure S 107. Normalized emission spectra of complexes (a) **RuP-Br** ($5 \cdot 10^{-5}$ M) (λ_{ex} = 513 nm), (b) **RuP-Br·py** ($5 \cdot 10^{-5}$ M) (λ_{ex} = 515 nm), (c) **(RuP-Br)$_2$·DABCO** (10^{-5} M) (λ_{ex} = 513 nm), (d) **(RuP-Br)$_2$·daz** ($5 \cdot 10^{-5}$ M) (λ_{ex} = 512 nm), (e) **(RuP-Br)$_2$·bpy** ($5 \cdot 10^{-5}$ M) (λ_{ex} = 513 nm) and (f) **(RuP-Br)$_2$·dpyb** ($5 \cdot 10^{-5}$ M) (λ_{ex} = 512 nm) in DCM.

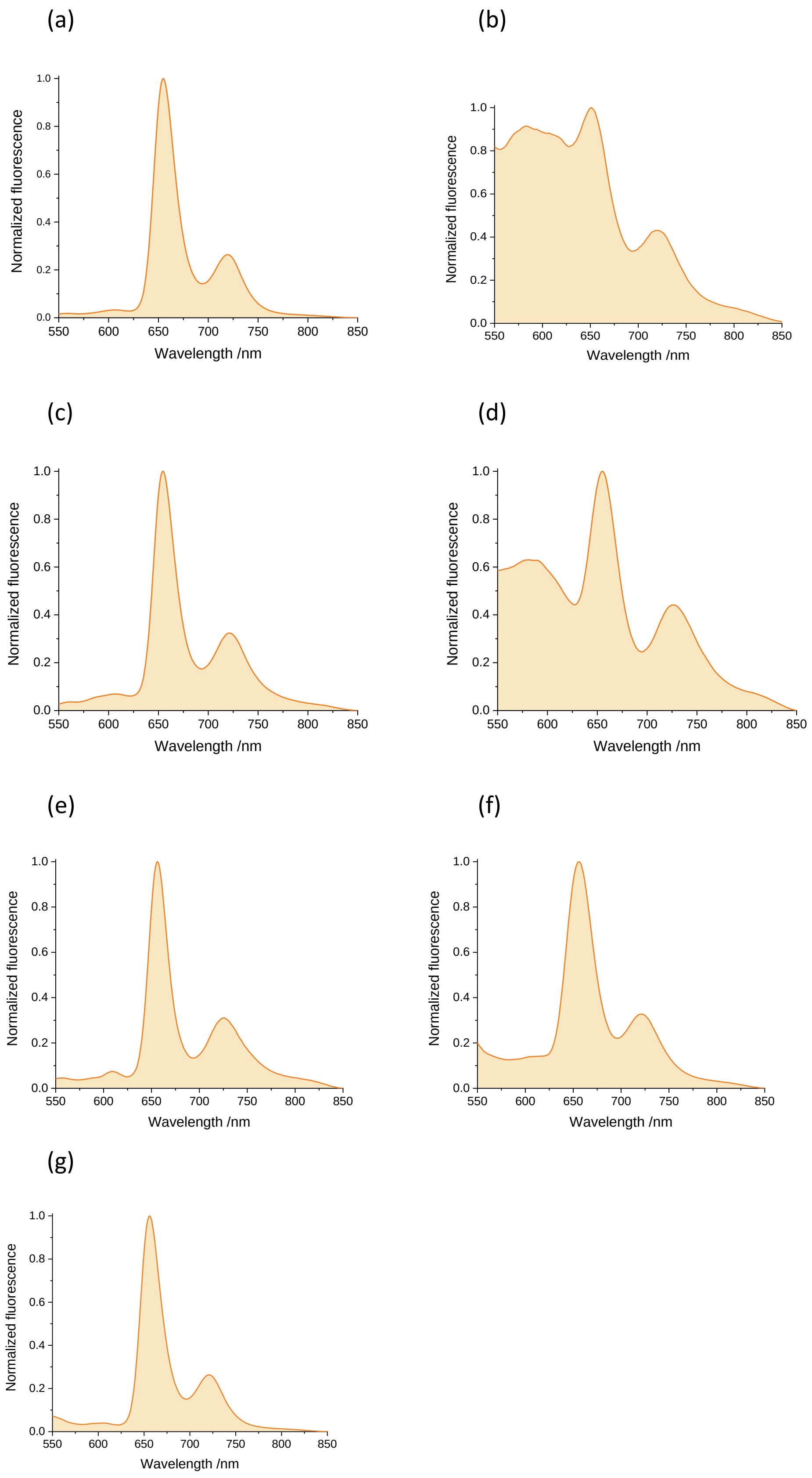

Figure S 108. Normalized emission spectra of complexes (a) **RuP-pyr·py** (5 · 10^{-5} M) (λ_{ex} = 514 nm), (b) **(RuP-pyr)$_2$·DABCO** (5 · 10^{-5} M) (λ_{ex} =518 nm), (c) **(RuP-pyr)$_2$·bpy** (5 · 10^{-5} M) (λ_{ex} = 514 nm), (d) **(RuP-pyr)$_2$·dpyb** (10^{-5} M) (λ_{ex} = 515 nm), (e) **RuP-cor·py** (5 · 10^{-5} M) (λ_{ex} = 515 nm), (f) **(RuP-cor)$_2$·bpy** (10^{-5} M) (λ_{ex} = 516 nm) and (g) **(RuP-cor)$_2$·dpyb** (5 · 10^{-5} M) (λ_{ex} = 516 nm) in DCM.

Table S 1. Fluorescence peaks of the different porphyrins prepared in this work.

**Compound**	***RuP-Br***	***RuP-Br·py***	***(RuP-Br)$_2$·daz***	***(RuP-Br)$_2$·DABCO***
Emission maxima	606 nm<br>655 nm<br>717 nm	609 nm<br>650 nm<br>722 nm	604 nm<br>653 nm<br>724 nm	605 nm<br>652 nm<br>717 nm
**Compound**	***(RuP-Br)$_2$·bpy***	***(RuP-Br)$_2$·dpyb***	***RuP-pyr·py***	***(RuP-pyr)$_2$·DABCO***
Emission maxima	605 nm<br>652 nm<br>725 nm	603 nm<br>654 nm<br>724 nm	655 nm<br>719 nm	583 nm<br>651 nm<br>721 nm
**Compound**	***(RuP-pyr)$_2$·bpy***	***(RuP-pyr)$_2$·dpyb***	***RuP-cor·py***	***(RuP-cor)$_2$·bpy***
Emission maxima	655 nm<br>721 nm	582 nm<br>655 nm<br>726 nm	656 nm<br>726 nm	656 nm<br>721 nm
**Compound**	***(RuP-cor)$_2$·dpyb***			
Emission maxima	656 nm<br>721 nm			

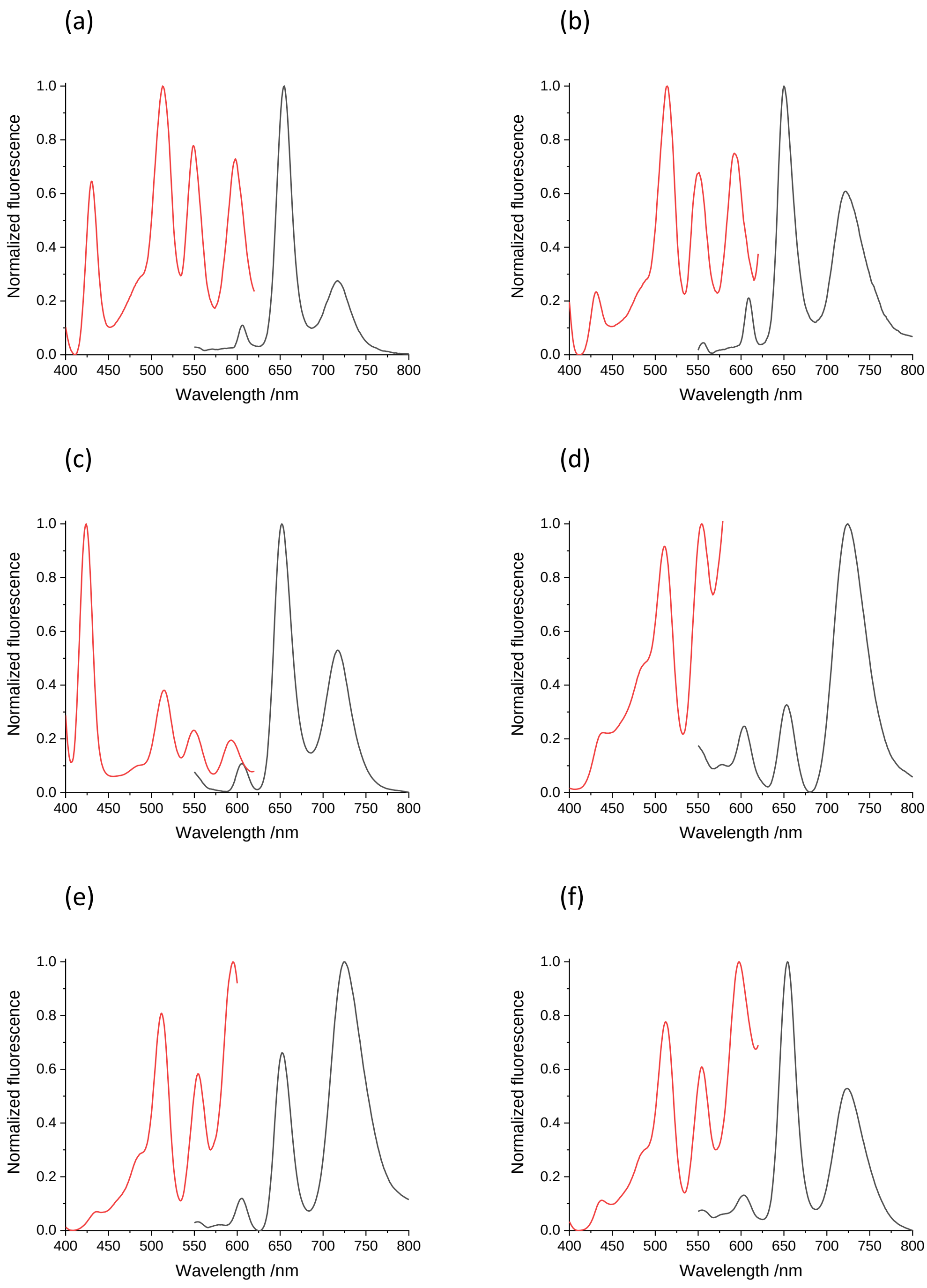

Figure S 109. Normalized excitation and emission spectra of complexes (a) **RuP-Br** ($5 \cdot 10^{-5}$ M) (λ_{em} = 655 nm, λ_{ex} = 513 nm), (b) **RuP-Br·py** ($5 \cdot 10^{-5}$ M) (λ_{em} = 650 nm, λ_{ex} = 515 nm), (c) **(RuP-Br)$_2$·DABCO** (10^{-5} M) (λ_{em} = 651 nm, λ_{ex} = 513 nm), (d) **(RuP-Br)$_2$·daz** ($5 \cdot 10^{-5}$ M) (λ_{em} = 653 nm, λ_{ex} = 512 nm), (e) **(RuP-Br)$_2$·bpy** ($5 \cdot 10^{-5}$ M) (λ_{em} = 652 nm, λ_{ex} = 513 nm) and (f) **(RuP-Br)$_2$·dpyb** ($5 \cdot 10^{-5}$ M) (λ_{em} = 654 nm, λ_{ex} = 512 nm) in DCM.

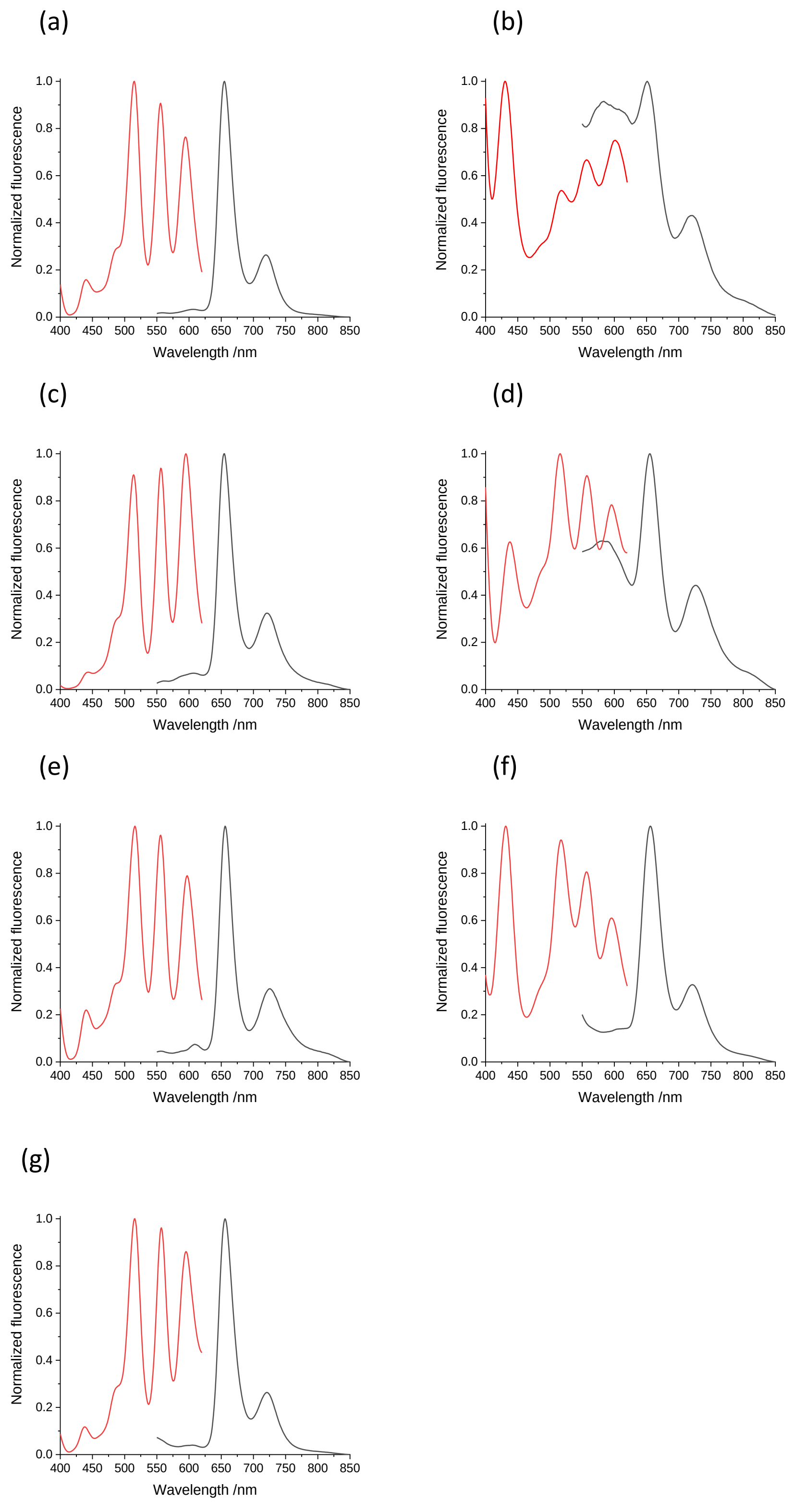

Figure S 110. Normalized excitation and emission spectra of complexes (a) **RuP-pyr·py** ($5 \cdot 10^{-5}$ M) (λ_{em} = 655 nm, λ_{ex} = 514 nm), (b) **(RuP-pyr)$_2$·DABCO** ($5 \cdot 10^{-5}$ M) (λ_{em} =655 nm, λ_{ex} = 518 nm), (c) **(RuP-pyr)$_2$·bpy** ($5 \cdot 10^{-5}$ M) (λ_{em} = 654 nm, λ_{ex} = 514 nm), (d) **(RuP-pyr)$_2$·dpyb** (10^{-5} M) (λ_{em} = 654 nm, λ_{ex} = 515 nm), (e) **RuP-cor·py** ($5 \cdot 10^{-5}$ M) (λ_{em} = 656 nm, λ_{ex} = 515 nm), (f) **(RuP-cor)$_2$·bpy** (10^{-5} M) (λ_{em} = 655 nm, λ_{ex} = 516 nm) and (g) **(RuP-cor)$_2$·dpyb** ($5 \cdot 10^{-5}$ M) (λ_{em} = 656 nm, λ_{ex} = 516 nm) in DCM.

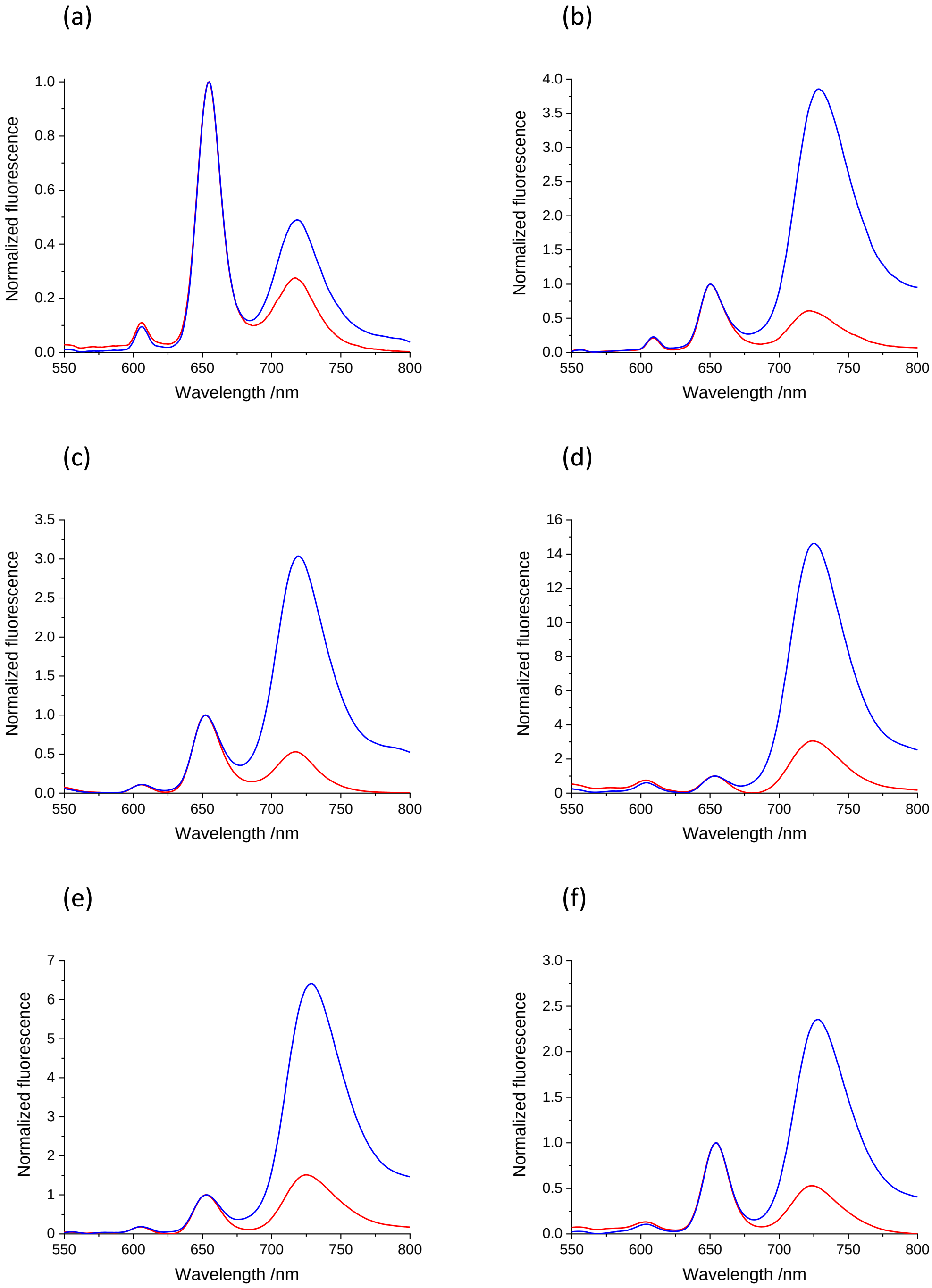

Figure S 111. Normalized emission spectra at the maximum around 650 nm of complexes (a) **RuP-Br** ($5 \cdot 10^{-5}$ M) (λ_{ex} = 513 nm), (b) **RuP-Br·py** ($5 \cdot 10^{-5}$ M) (λ_{ex} = 515 nm), (c) **(RuP-Br)$_2$·DABCO** (10^{-5} M) (λ_{ex} = 513 nm), (d) **(RuP-Br)$_2$·daz** ($5 \cdot 10^{-5}$ M) (λ_{ex} = 512 nm), (e) **(RuP-Br)$_2$·bpy** ($5 \cdot 10^{-5}$ M) (λ_{ex} = 513 nm) and (f) **(RuP-Br)$_2$·dpyb** ($5 \cdot 10^{-5}$ M) (λ_{ex} = 512 nm) in DCM (red line) and in deareated DCM (blue line).

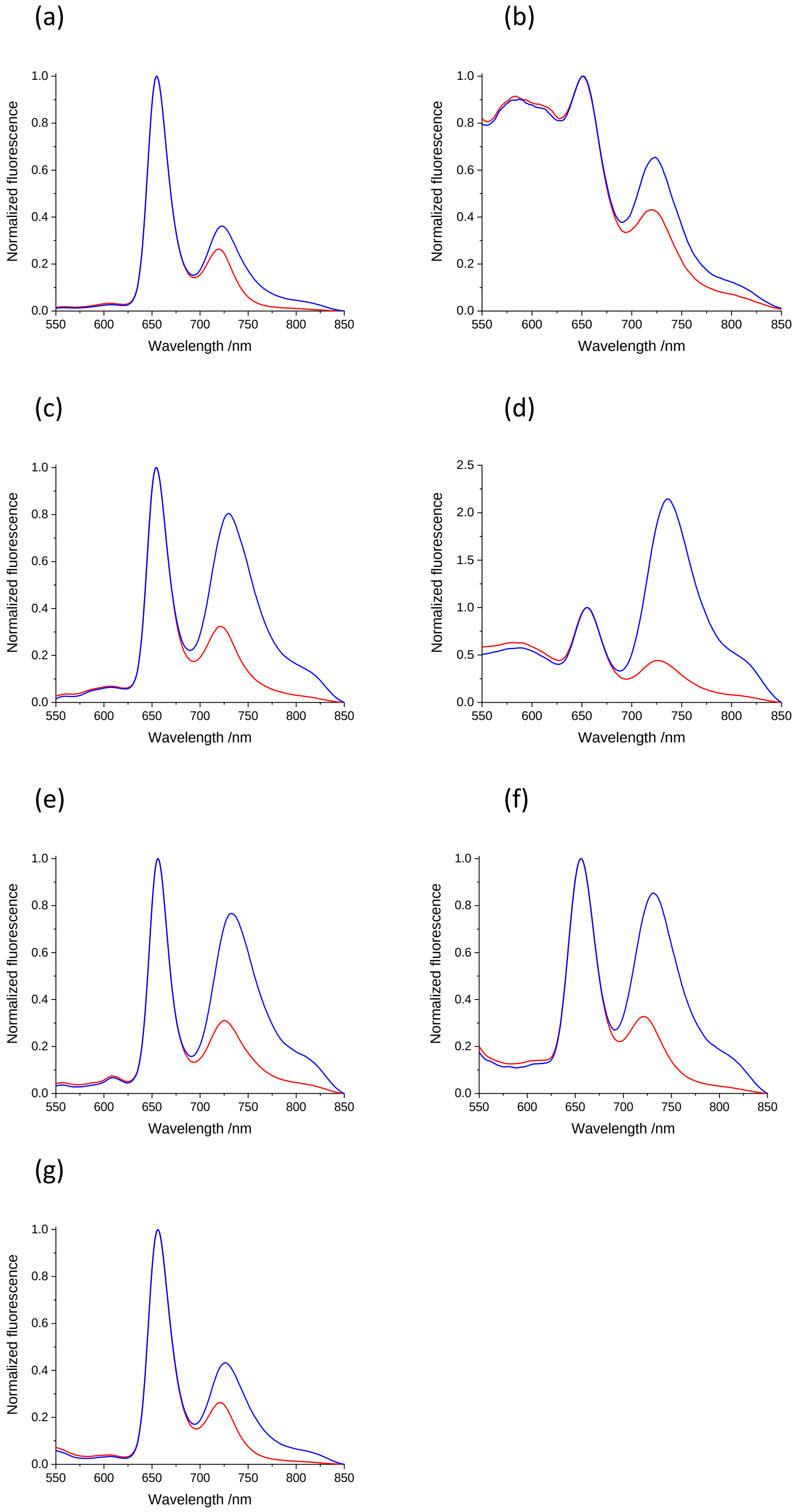

Figure S 112. Normalized emission spectra at the maximum around 650 nm of complexes (a) **RuP-pyr·py** ($5 \cdot 10^{-5}$ M) (λ_{ex} = 514 nm), (b) **(RuP-pyr)$_2$·DABCO** ($5 \cdot 10^{-5}$ M) (λ_{ex} = 518 nm) (b) **(RuP-pyr)$_2$·bpy** ($5 \cdot 10^{-5}$ M) (λ_{ex} = 514 nm), (c) **(RuP-pyr)$_2$·dpyb** (10^{-5} M) (λ_{ex} = 515 nm), (d) **RuP-cor·py** ($5 \cdot 10^{-5}$ M) (λ_{ex} = 515 nm), (e) **(RuP-cor)$_2$·bpy** (10^{-5} M) (λ_{ex} = 516 nm) and (f) **(RuP-cor)$_2$·dpyb** ($5 \cdot 10^{-5}$ M) (λ_{ex} = 516 nm) in DCM (red line) and in deareated DCM (blue line).

X-ray Crystallographic Tables

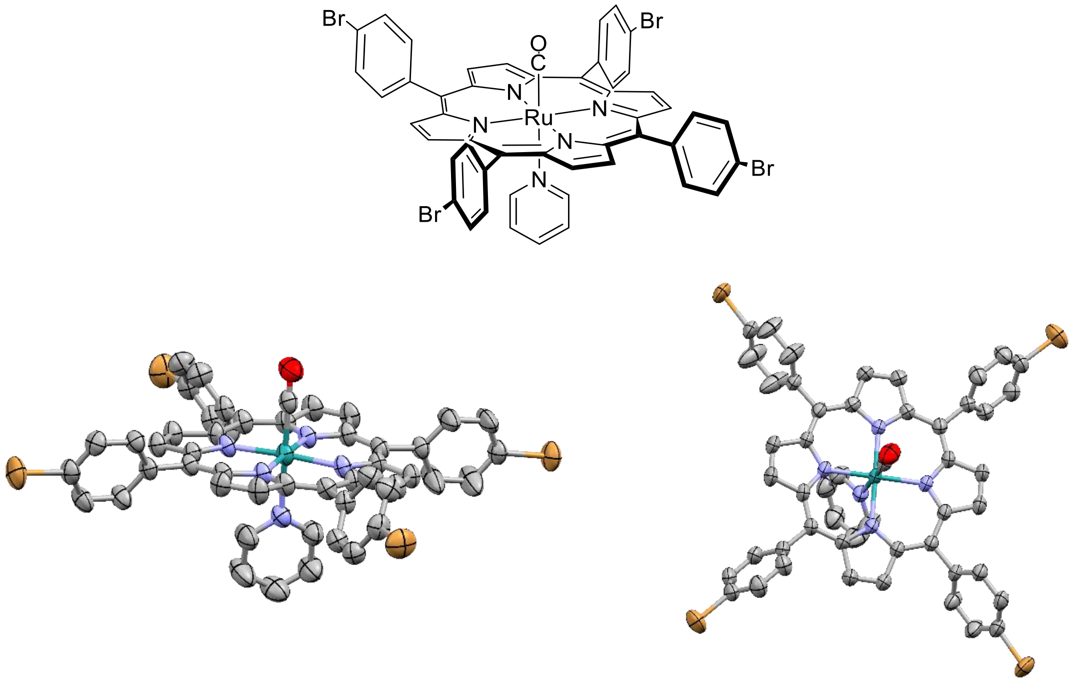

Figure S 113. Schematic representation and crystal structure of compound **RuP-Br·py** (50% ellipsoid contour probability). Solvent and hydrogen atoms were omitted for clarity. Crystallization conditions: slow evaporation of a solution of the compound in DCM at 25°C.

Table S 2. Crystallographic data of compound **RuP-Br·py**.

Identification code	*RuP-Br·py*
CCDC Number	2372292
Empirical formula	$C_{51}H_{31}Br_4Cl_2N_5ORu$
Formula weight	1221.42
Temperature [K]	298
Crystal system	monoclinic
Space group	C2/c
a [Å]	28.8029(10)
b [Å]	15.1487(3)
c [Å]	23.2307(8)
α [°]	90
β [°]	110.448(4)
γ [°+	90
V [Å^3]	9497.5(6)
Z	8
ρ_{calc} [g/cm^3]	1.708
μ [mm^{-1}]	7.998
F(000)	4784.0
Crystal size [mm^3]	0.351 × 0.215 × 0.063
Radiation	CuKα (λ = 1.54184)
2Θ range for data collection [°]	7.21 to 150.364
Index ranges	-35 ≤ h ≤ 36, -17 ≤ k ≤ 18, -17 ≤ l ≤ 28
Reflections collected	31809
Independent reflections	9636 [R_{int} = 0.0434, R_{sigma} = 0.0369]
Data/restraints/parameters	9636/0/577
Goodness-of-fit on F^2	1.035
Final R indexes [I>=2σ (I)]	R_1 = 0.0561, wR_2 = 0.1499
Final R indexes [all data]	R_1 = 0.0769, wR_2 = 0.1699
Max/min Δρ [e Å^{-3}]	1.40/-0.85
Flack parameter	-

Supramolecular titrations

Fluorescence titrations

In order to estimate the association constants (K_a) of complexes **RuP-cor·py**, **(RuP-cor)$_2$·bpy** and **(RuP-cor)$_2$·dpyb** with C_{60} the following procedure was carried out: a stock solution of each porphyrin complex (10^{-5} – 10^{-6} M) in toluene was prepared and a known volume (2 mL) was transferred to a quartz cuvette. The titration was carried out by adding portions of another stock solution containing the same concentration of the porphyrin complex and C_{60} ($5.0 \cdot 10^{-4}$ M) in toluene to cover a wide range of equivalents. An emission spectrum was recorded at 25°C of the resulting mixture after each addition. Once all the data had been obtained, the quenching of the selected emission band (ΔF) was plotted as a function of the concentration of the guest, and the resulting curve was fitted by a nonlinear method using the global analysis approach according to the following equations assuming different equilibria.

<u>Fitting the experimental data to 1:1 and 1:2 models</u>

The fluorescence titration data was fitted to 1:1 and 1:2 binding models using supramolecular.org. The equations for these models have been detailed previously[7–9] and will not be repeated here. Links to all the fittings are provided below.

<u>Fitting the experimental data to a 1:3 model</u>

The data was fitted to a 1:3 model using the same approach to that previously described by Miyake, Thordarson, and co-workers in their UV-Vis study on a 1:3 complexation of a Ni-morpholine complex.[10] The most important equations are summarized below in terms of fluorescence spectroscopy.

$$K_1 = \frac{[HG]}{[H][G]} \; eq.1 \qquad K_2 = \frac{[HG_2]}{[HG][G]} \; eq.2 \qquad K_3 = \frac{[HG_3]}{[HG_2][G]} \; eq.3$$

These equations together with the corresponding mass-balance equations form a system of equations that yields a fourth order equation for the concentration of the free guest [G]:

$$K_1K_2K_3[G]^4 + K_1K_2\big(1 + K_3(3[H]_0 - [G]_0)\big)[G]^3 + K_1\big(1 + K_2(2[H]_0 - [G]_0)\big)[G]^2 + \big(1 + K_1([H]_0 - [G]_0)\big)[G] - [G]_0 = 0 \; eq.4$$

The general equation to fit the quenching of the fluorescence intensity of the host upon addition of the guest according to a 1:3 model is:

$$\Delta F_{obs} = \frac{k_{\Delta HG}K_1[H]_0[G] + k_{\Delta HG_2}K_1K_2[H]_0[G]^2 + k_{\Delta HG_3}K_1K_2K_3[H]_0[G]^3}{1 + K_1[G] + K_1K_2[G]^2 + K_1K_2K_3[G]^3} \; eq.5$$

The data have been fitted to this model using Matlab, and the results are summarized below.

<u>Fitting the experimental data to a 1:4 model</u>

For 1:4 H:G_4 complexation occurs according to equations 6 to 9.

$$K_1 = \frac{[HG]}{[H][G]} \; eq.6 \qquad K_2 = \frac{[HG_2]}{[HG][G]} \; eq.7 \qquad K_3 = \frac{[HG_3]}{[HG_2][G]} \; eq.8 \qquad K_4 = \frac{[HG_4]}{[HG_3][G]} \; eq.9$$

Equations 6 to 9, together with the corresponding mass balance equations form a system of equations that is solved to yield a fifth-order equation (eq. 10) for the concentration of the free guest [G]:

$$K_1K_2K_3K_4[G]^5 + K_1K_2K_3\big(1 + K_4(4[H]_0 - [G]_0)\big)[G]^4 + K_1K_2\big(1 + K_3(3[H]_0 - [G]_0)\big)[G]^3 + K_1\big(1 + K_2(2[H]_0 - [G]_0)\big)[G]^2 + \big(1 + K_1([H]_0 - [G]_0)\big)[G] - [G]_0 = 0 \quad eq. 10$$

Following a similar approach to the previously described 1:3 model for fluorescence titrations, changes in fluorescence intensity of the host upon emission titration are expressed as in equation 11.

$$\Delta F_{obs} = \frac{k_{\Delta HG}K_1[H]_0[G] + k_{\Delta HG_2}K_1K_2[H]_0[G]^2 + k_{\Delta HG_3}K_1K_2K_3[H]_0[G]^3 + k_{\Delta HG_4}K_1K_2K_3K_4[H]_0[G]^4}{1 + K_1[G] + K_1K_2[G]^2 + K_1K_2K_3[G]^3 + K_1K_2K_3K_4[G]^4} \quad eq. 11$$

Where:

[G] is the concentration of free guest (C_{60}).

$[H]_0$ is the total concentration of the host (**RuP-cor·py**, **(RuP-cor)$_2$·bpy**, **(RuP-cor)$_2$·dpyb**).

$k_{\Delta HG}$ is the difference between the proportionality constants of the complex HG and the host H in the first equilibrium.

$k_{\Delta HG2}$ is the difference between the proportionality constants of the complex HG_2 and the host H in the second equilibrium.

$k_{\Delta HG3}$ is the difference between the proportionality constants of the complex HG_3 and the host H in the third equilibrium.

$k_{\Delta HG4}$ is the difference between the proportionality constants of the complex HG_4 and the host H in the fourth equilibrium.

K_1 is the estimated association constant for the first equilibrium.

K_2 is the estimated association constant for the second equilibrium.

K_3 is the estimated association constant for the third equilibrium.

K_4 is the estimated association constant for the fourth equilibrium.

In this analysis, four different binding models ("flavours") have been compared. The first one is the stepwise (non-degenerate) full 1:4 binding model. This assumes that each host has four non-identical binding sites that allow for cooperativity. In this model all the parameters in equation 11 are evaluated independently.

The second model considered is the stepwise (non-degenerate), additive 1:4 binding model. To reduce the number of parameters to fit in equation 11 the assumption that the proportionality constants of the 1:1, 1:2, 1:3 and 1:4 are additive is made. This means that the quenching in the fluorescence intensity from the free host to the 1:1 adduct is 1/2 of the change between the 1:2 complex and the free host, 1/3 of the change between the 1:3 complex and the free host and 1/4 of the change between the 1:4 complex and the free host. Then it follows that $k_{\Delta HG} = 1/2k_{\Delta HG2} = 1/3k_{\Delta HG3} = 1/4k_{\Delta HG4}$ and eq. 11 can be simplified to eq. 12.

$$\Delta F_{obs} = \frac{k_{\Delta HG}(K_1[H]_0[G] + 2K_1K_2[H]_0[G]^2 + 3K_1K_2K_3[H]_0[G]^3 + 4K_1K_2K_3K_4[H]_0[G]^4)}{1 + K_1[G] + K_1K_2[G]^2 + K_1K_2K_3[G]^3 + K_1K_2K_3K_4[G]^4} \quad eq. 12$$

The third model is the stepwise non-cooperative 1:4 binding model. In this model, the assumption that proportionality constants are not correlated is made. In this case, the assumption is that 1:4 complexation is non-cooperative and, after considering statistical factors, that $K_1 = 8/3K_2 = 6K_3 = 16K_4$. Then, K_{1n} is defined as $K_{1n} = K_1 = 8/3K_2 = 6K_3 = 16K_4$, and eq. 11 can be simplified to eq. 13.

$$\Delta F_{obs} = \frac{k_{\Delta HG}K_{1n}[H]_0[G] + k_{\Delta HG_2}K_{1n}^2[H]_0[G]^2 + k_{\Delta HG_3}K_{1n}^3[H]_0[G]^3 + k_{\Delta HG_4}K_{1n}^4[H]_0[G]^4}{1 + K_{1n}[G] + K_{1n}^2[G]^2 + K_{1n}^3[G]^3 + K_{1n}^4[G]^4} \quad eq. 13$$

By making this assumption, K_{1n} replaces the need to fit four parameters (each association constant) and once the value of K_{1n} is known after the fitting, the stepwise non-cooperative binding constants can be readily obtained with $K_{1n} = K_1 = 8/3K_2 = 6K_3 = 16K_4$.

The last model is the statistical 1:4 binding model. Here both assumptions are made, that the binding is non-cooperative ($K_1 = 8/3K_2 = 6K_3 = 16K_4$) and that the proportionality constants are additive ($k_{\Delta HG} = 1/2k_{\Delta HG2} = 1/3k_{\Delta HG3} = 1/4k_{\Delta HG4}$). This way eq. 11 gets simplified to eq. 14.

$$\Delta F_{obs} = \frac{k_{\Delta HG}(K_{1n}[H]_0[G] + 2K_{1n}^2[H]_0[G]^2 + 3K_{1n}^3[H]_0[G]^3 + 4K_{1n}^4[H]_0[G]^4)}{1 + K_{1n}[G] + K_{1n}^2[G]^2 + K_{1n}^3[G]^3 + K_{1n}^4[G]^4} \quad eq. 14$$

In this model the only parameters to fit are the proportionality constant $k_{\Delta HG}$ and K_{1n}.

All the analysis has been done using Matlab, and the results are summarised below.

RuP-pyr·py vs C_{60}

As it was expected for the planar pyrene substituents, this complex showed a negligible quenching upon addition of 10 equivalents of C_{60}, as it is shown in Figure S 107. Attempts to fit the data resulted in non-convergence or non-sensible association constants (like negative *K* values). The same results were obtained for dimers **(RuP-pyr)₂·bpy** and **(RuP-pyr)₂·dpyb**.

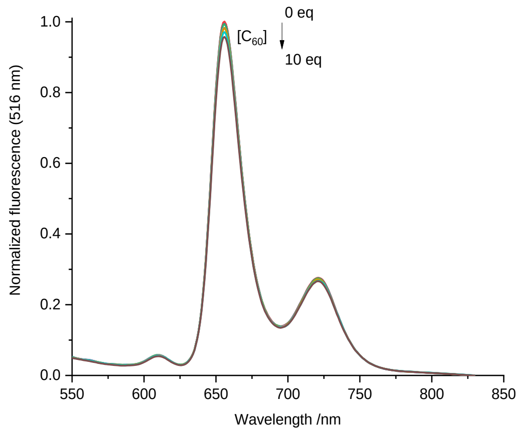

Figure S 114. Normalized emission spectra (λ_{ex} = 516 nm) of compound **RuP-pyr·py** upon addition of C_{60} in toluene at 298 K.

RuP-cor·py vs C_{60}

1 to 1

http://app.supramolecular.org/bindfit/view/c031bd1f-e8a3-47a8-80ab-54c34eef1a0e

1 to 2 full

http://app.supramolecular.org/bindfit/view/ec5bcad7-b995-40d2-bf36-f4170a030e30

1 to 2 additive

http://app.supramolecular.org/bindfit/view/d8c0c975-d78d-4e2b-83d1-6d598514d216

1 to 2 non cooperative

http://app.supramolecular.org/bindfit/view/219fadea-fa1e-48d7-9274-6d61752f686c

1 to 2 statistical

http://app.supramolecular.org/bindfit/view/890d0800-e336-4916-8dc7-6fe5704fce1f

Table S 3. Association constants of host **RuP-cor·py** with C_{60} according to different binding models.

Model	K_1	K_2	SSR	SE_y	Cov_{fit} ratio
**1 to 1**	373	-	3.96	0.57	1
**1 to 2 full**	-2394	-587	2.62	0.51	1.58
**1 to 2 additive**	3179	2508	3.00	0.52	1.38
**1 to 2 non coop**	6430	1608	3.10	0.53	1.33
**1 to 2 statistical**	761	190	3.96	0.57	1.00

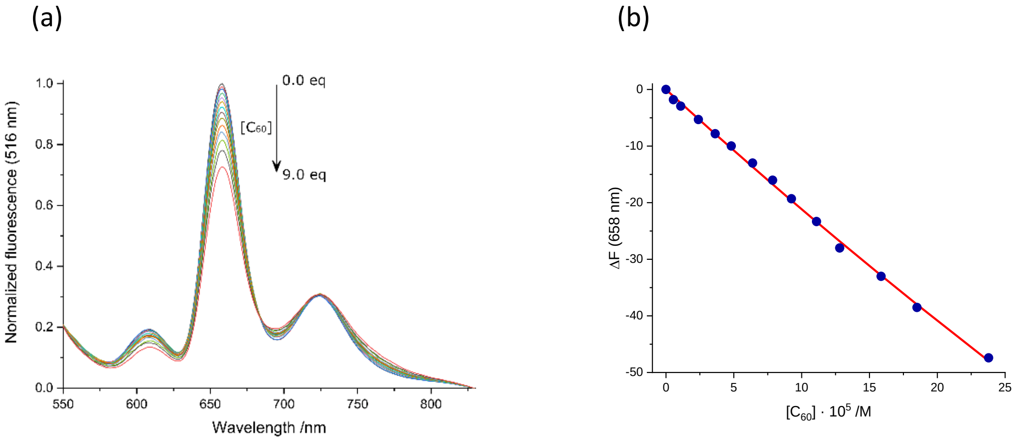

Figure S 115. (a) Normalized emission spectra (λ_{ex} = 516 nm) of complex **RuP-cor·py** upon addition of C_{60} in toluene at 298 K. (b) Binding isotherm of the quenching of the fluorescence at 658 nm. The blue dots represent the experimental data, and the red line corresponds to the fit obtained by non-linear regression with a 1 to 1 model.

(RuP-cor)$_2$·bpy vs C_{60}

1 to 1

http://app.supramolecular.org/bindfit/view/27eb2dd4-f48a-4ba8-841f-83b47e181750

1 to 2 full

http://app.supramolecular.org/bindfit/view/15ea46ae-45bb-4945-8e42-be37f230e51b

1 to 2 additive

http://app.supramolecular.org/bindfit/view/770b5d41-2ccc-49f5-942b-c694be6b297c

1 to 2 non cooperative

http://app.supramolecular.org/bindfit/view/a0ffaccc-bf8a-4671-8924-6cb8c2a93468

1 to 2 statistical

http://app.supramolecular.org/bindfit/view/655a1cd8-1096-4cc6-a938-f50f39cf94b7

Table S 4. Association constants of host **(RuP-cor)$_2$·bpy** with C_{60} according to different binding models. [a]The fit did not converge.

Model	K_1	K_2	K_3	K_4	SSR	SE_y	Cov_{fit} ratio
**1 to 1**	2050	-	-	-	103.67	3.39	1
**1 to 2 full**	117693	-511	-	-	4.86	0.83	17.97
**1 to 2 additive**	3177	476	-	-	101.11	3.56	1.02
**1 to 2 non coop**	4074	1019	-	-	103.75	3.60	1.00
**1 to 2 statistical**	4139	1035	-	-	103.76	0.57	1.00
**1 to 3 full**[a]	238188	-32	$-1.7 \cdot 10^{-7}$	-	6.63	1.15	13.12
**1 to 3 additive**	2050	$2.0 \cdot 10^{-6}$	$1.0 \cdot 10^{-17}$	-	103.73	3.85	1.00
**1 to 3 non coop**	29585	9862	3287	-	9.66	1.17	9.05
**1 to 3 statistical**	6269	2090	697	-	103.92	3.40	1.00
**1 to 4 full**[a]	178806	1645	$1.9 \cdot 10^{-6}$	13	415.65	11.77	0.26
**1 to 4 additive**	3176	476	$2.5 \cdot 10^{-8}$	$4.5 \cdot 10^{-2}$	101.17	4.11	1.01
**1 to 4 non coop**	21226	7960	3538	1327	8.53	1.19	10.49
**1 to 4 statistical**	8441	3165	1407	528	104.01	3.40	1.00

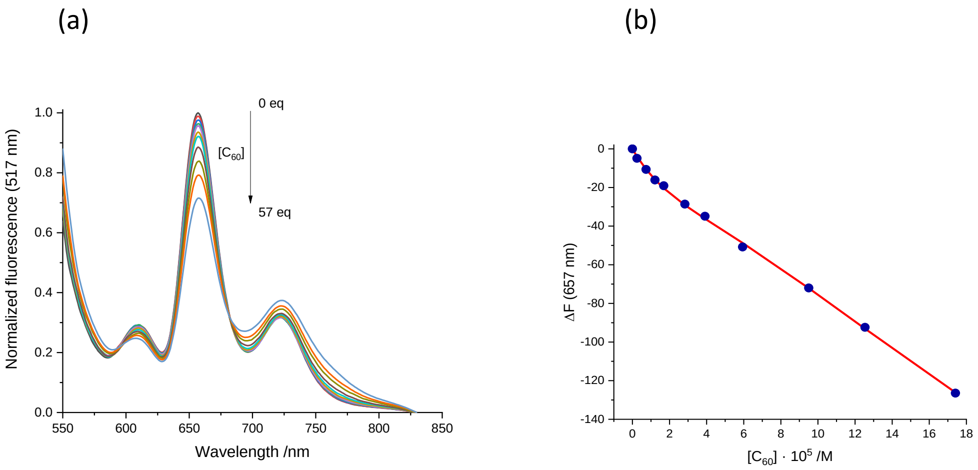

Figure S 116. (a) Normalized emission spectra (λ_{ex} = 517 nm) of complex **(RuP-cor)$_2$·bpy** upon addition of C_{60} in toluene at 298 K. (b) Binding isotherm of the quenching of the fluorescence at 657 nm. The blue dots represent the experimental data, and the red line corresponds to the fit obtained by non-linear regression with a 1 to 4 non cooperative model.

(RuP-cor)$_2$·dpyb vs C_{60}

1 to 1

http://app.supramolecular.org/bindfit/view/70ff294b-b6ae-403f-af6e-586b8758c0d5

1 to 2 full

http://app.supramolecular.org/bindfit/view/739feea7-11cb-4587-b650-21a0db69ee7d

1 to 2 additive

http://app.supramolecular.org/bindfit/view/4789329c-34da-4fe8-818e-28422b82346a

1 to 2 non cooperative

http://app.supramolecular.org/bindfit/view/6232f995-e190-4fc6-b7f7-19bd96a8c262

1 to 2 statistical

http://app.supramolecular.org/bindfit/view/1a7b498f-c940-46b2-95fa-00f8682ebce0

Table S 5. Association constants of host **(RuP-cor)$_2$·dpyb** with C_{60} according to different binding models. [a]The fit did not converge.

Model	K_1	K_2	K_3	K_4	SSR	SE_y	Cov_{fit} ratio
**1 to 1**	2422	-	-	-	42.73	2.07	1
**1 to 2 full**	1718388	697	-	-	13.83	1.31	2.77
**1 to 2 additive**	3724	572	-	-	42.15	2.16	1.01
**1 to 2 non coop**	4789	1197	-	-	42.77	2.18	1.00
**1 to 2 statistical**	4924	1233	-	-	42.77	2.07	1.00
**1 to 3 full[a]**	27713	5375	30949	-	8.87	1.22	4.32
**1 to 3 additive**	22213	2020	25770	-	17.54	1.48	2.23
**1 to 3 non coop**	57810	19270	6423	-	10.09	1.12	3.80
**1 to 3 statistical**	7523	2508	836	-	43.10	2.08	0.99
**1 to 4 full[a]**	16092	780	3.5 · 10-9	0.12	587.45	12.12	0.09
**1 to 4 additive**	23412	2551	20688	608	17.75	1.59	2.20
**1 to 4 non coop**	30812	11554	5135	1926	8.45	1.10	4.54
**1 to 4 statistical**	10228	3836	1705	639	43.14	2.08	0.99

(a) (b)

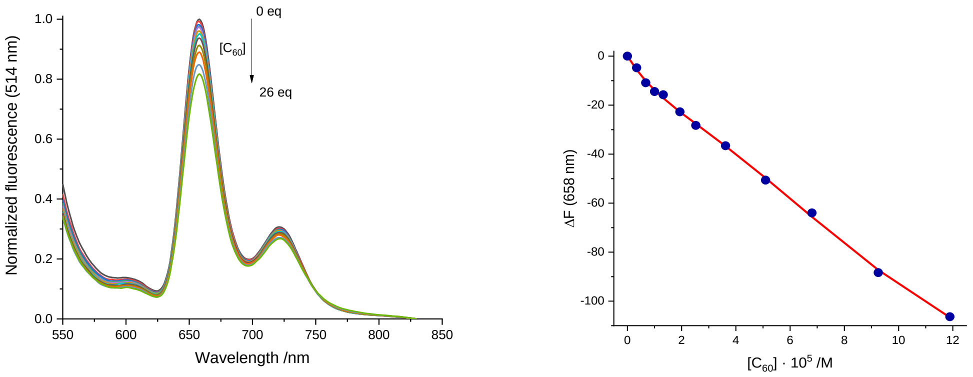

Figure S 117. (a) Normalized emission spectra (λ_{ex} = 514 nm) of complex **(RuP-cor)$_2$·dpyb** upon addition of C_{60} in toluene at 298 K. (b) Binding isotherm of the quenching of the fluorescence at 658 nm. The blue dots represent the experimental data, and the red line corresponds to the fit obtained by non-linear regression with a 1 to 4 non cooperative model.

Table S 6. Summary of the association constants (M^{-1}) obtained for the adducts formed between the complexes and C_{60}. [a]Uncertainties for the 1 to 4 non cooperative model were estimated with Monte Carlo simulations.[9,11] [b]In M^{-4}.

Compound	K_1	K_2	K_3	K_4	β [b]
RuP-cor·py	$(3.73 \pm 0.06) \times 10^2$	-	-	-	-
(RuP-cor)₂·bpy[a]	$(2.12 \pm 0.12) \times 10^4$	$(7.96 \pm 0.45) \times 10^3$	$(3.54 \pm 0.20) \times 10^3$	$(1.33 \pm 0.08) \times 10^3$	7.93×10^{14}
(RuP-cor)₂·dpyb[a]	$(3.08 \pm 0.29) \times 10^4$	$(1.16 \pm 0.11) \times 10^4$	$(5.14 \pm 0.48) \times 10^3$	$(1.93 \pm 0.18) \times 10^3$	3.52×10^{15}

UV-Vis titrations

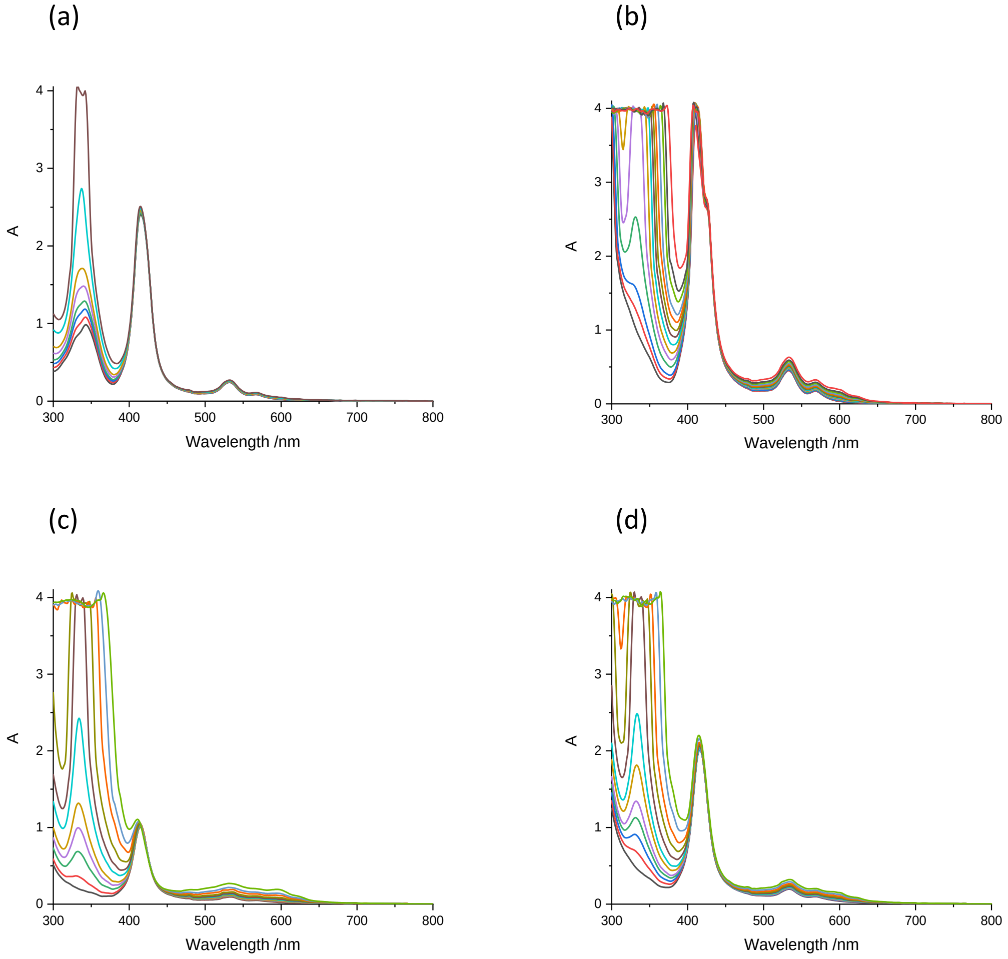

Figure S 118. UV-Vis stacked spectra of (a) **RuP-pyr·py**, (b) **RuP-cor·py**, (c) **(RuP-cor)$_2$·bpy** and (d) **(RuP-cor)$_2$·dpyb** in toluene solution upon addition of C_{60}.

^{1}H NMR titrations

The binding studies with C_{60} by ^{1}H NMR titration experiments were also performed for the porphyrin complexes. In this case, a solution of the compound (10^{-5}M) in deuterated toluene (tol-d_8) was prepared, and a known volume (0.5 mL) was transferred to an NMR tube covered with a septum. The titration was carried out by adding known portions of a stock solution of the fullerene (10^{-3}M) in tol-d_8 to cover a wide range of equivalents. A ^{1}H-NMR experiment was conducted at room temperature (298 K) after each addition. Once all data had been obtained, the changes in chemical shifts ($\Delta\delta$) of selected protons were plotted as a function of the molar fraction of the guest, and the resulting curve was fitted by a nonlinear method using the global

analysis approach according to the following equations assuming a 1:1 equilibrium as mentioned before[7–9].

<u>*RuP-cor·py* vs C_{60}</u>

1 to 1

http://app.supramolecular.org/bindfit/view/92bfa133-d210-47c5-b446-f2b0dcbc8467

1 to 2 full

http://app.supramolecular.org/bindfit/view/05ec9b59-ff40-4713-9024-d49c814166dd

1 to 2 additive

http://app.supramolecular.org/bindfit/view/ac09e8d6-182a-46d5-b6af-3c8634842b2a

1 to 2 non cooperative

http://app.supramolecular.org/bindfit/view/b59944db-1527-45c7-acfa-3b180a3256be

1 to 2 statistical

http://app.supramolecular.org/bindfit/view/6bd92a94-d6c1-4433-a936-adb18deddc3e

Table S 7. Association constants of host **RuP-cor·py** with C_{60} according to different binding models.

Model	K_1	K_2	SSR	SE_y	Cov_{fit} ratio
**1 to 1**	362	-	$1.56 \cdot 10^{-6}$	$2.05 \cdot 10^{-4}$	1
**1 to 2 full**	-789	339	$1.01 \cdot 10^{-6}$	$1.70 \cdot 10^{-4}$	1.54
**1 to 2 additive**	1337	1334	$1.46 \cdot 10^{-6}$	$2.01 \cdot 10^{-4}$	1.05
**1 to 2 non coop**	3429	857	$1.16 \cdot 10^{-6}$	$1.80 \cdot 10^{-4}$	1.32
**1 to 2 statistical**	735	184	$1.56 \cdot 10^{-6}$	$2.05 \cdot 10^{-4}$	1.00

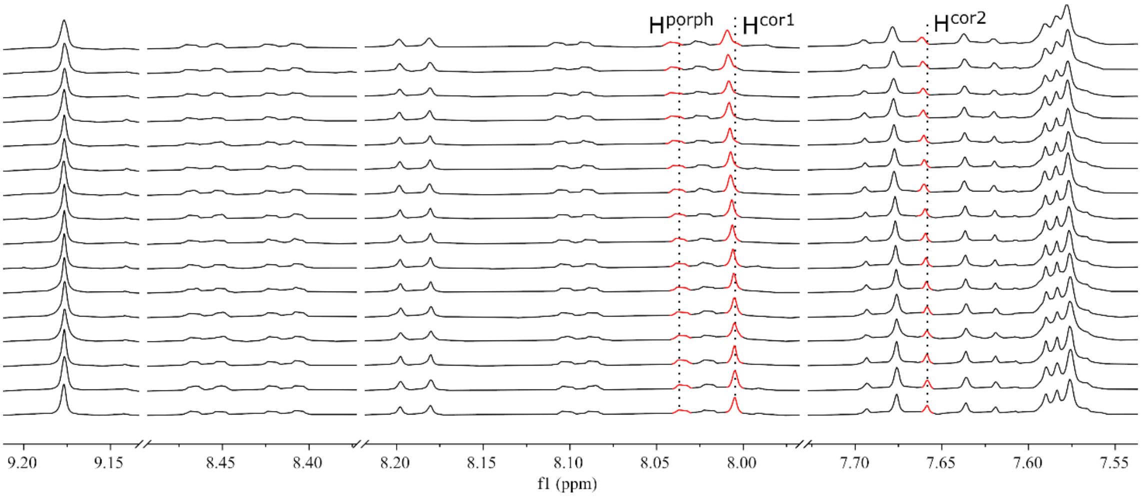

Figure S 119. Stacked ^{1}H-NMR spectra for the titration of **RuP-pyr·py** with variable concentrations of C_{60} in tol-d_8 at 298 K. The most significant chemical shifts of the compound (H^{porph}, H^{cor1}, H^{cor2}) have been labelled.

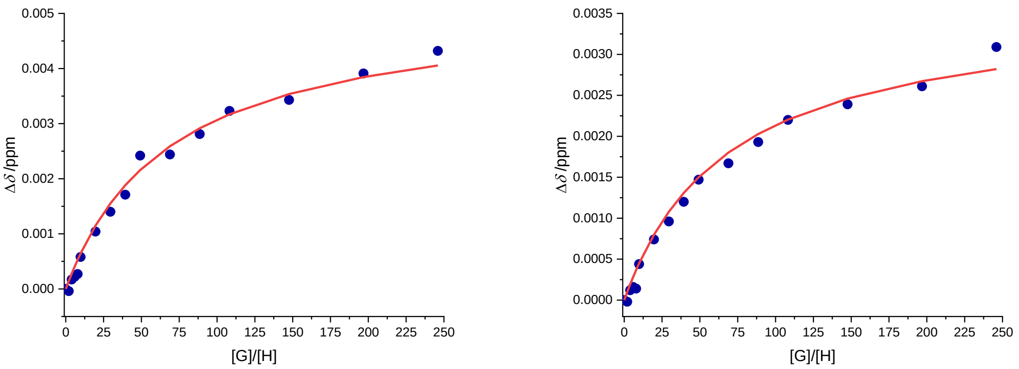

Figure S 120. Non-linear regressions for selected protons (left plot: H^{cor1}, right plot: H^{cor2}) for the titration of **RuP-cor·py** with C_{60}.

Porphyrin dimers vs C_{60}

(a) (b)

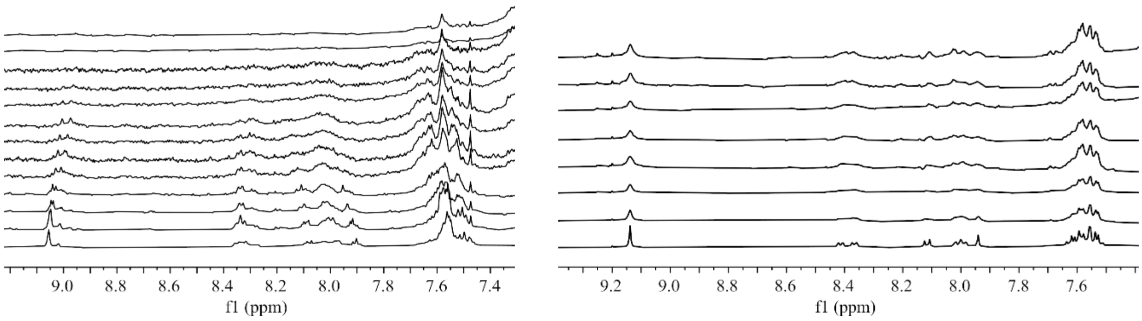

Figure S 121. Stacked ^{1}H NMR spectra for the titration of (a) **(RuP-cor)$_2$·bpy** and (b) **(RuP-cor)$_2$·dpyb** with variable concentrations of C_{60} in tol-d_8 at 298 K.

Variable temperature (VT) ^{1}H NMR experiments

(a) (b)

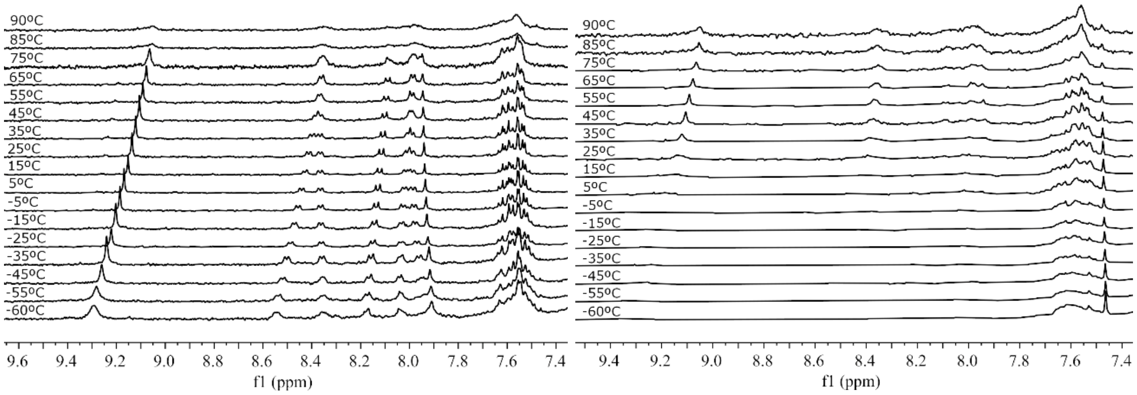

Figure S 122. VT ^{1}H NMR stacked spectra of (a) **(RuP-cor)$_2$·dpyb** in tol-d_8 (10^{-5} M) and (b) same sample after adding 10 equivalents of C_{60}.

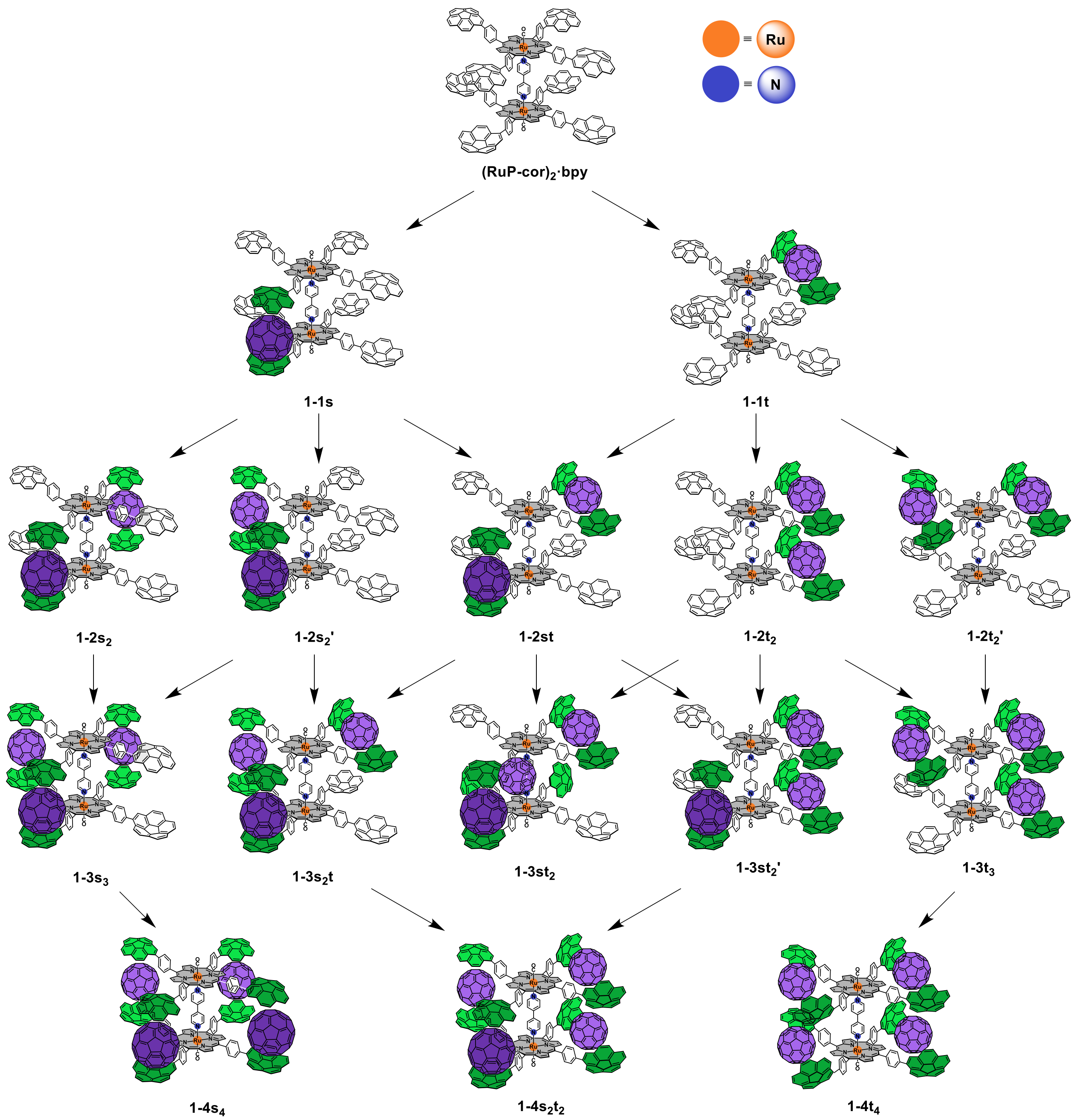

Scheme S 2. Tree diagram illustrating the possible isomers of the resulting supramolecular adducts, where C_{60} is recognized in a stepwise manner, differentiating between sandwich-like (s) and tweezer-like (t) assembly modes. It is important to note that not all statistical possibilities are represented.

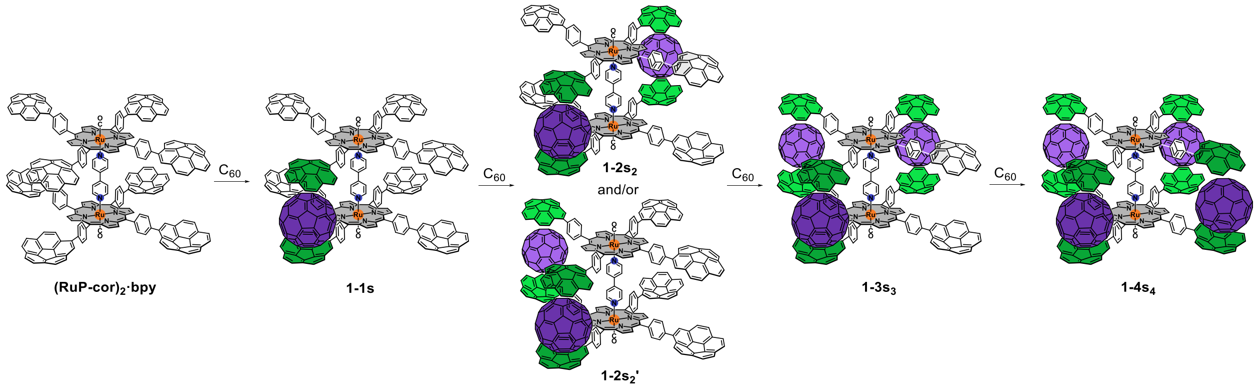

Scheme S 3. Most likely sequential binding mechanism of C_{60} by host **(RuP-cor)$_2$ bpy**.

Computational Calculations details

Quantum-chemical calculations

Geometry optimizations were performed using the self-consistent tight-binding GFN2-xTB method.[12,13] Energy decomposition analysis (EDA)[14,15] at BLYP(D3BJ)/TZP level[16-19] using the GFN2-xTB geometries was performed using the Amsterdam Density Functional (ADF) program.[20] Chemcraft 1.8. program[21] was utilized for molecular structures and NCI isosurfaces visualization.

Complexation, interaction and deformation energies

The interaction energies were calculated directly from the electronic energy of complex and electronic energies of its subsystems. For **C_{60}@(RuP-cor)$_2$·bpy**, the interaction energy can be expressed as follows:

$$E_{\text{int}} = E_{\mathbf{C_{60}@(RuP-cor)_2 \cdot bpy}} - \left(E_{\mathbf{(RuP-cor)_2 \cdot bpy}} + E_{\mathbf{C_{60}}}\right) \qquad eq.\ 15$$

Deformation energy for each of studied complexes has been determined using following equation:

$$E_{def} = \left(E_{Host}^{Complex\,geom.} - E_{Host}^{Eq.geom.}\right) + \left(E_{C_{60}}^{Complex\,geom.} - E_{C_{60}}^{Eq.geom.}\right) \qquad eq.\ 16$$

At the same time, complexation energy comprises both interaction ($\Delta E_{int.}$) and deformation ($E_{def.}$) energies. Thus $\Delta E_{Complex.}$ can be represented as following:

$$E_{Complex} = E_{\text{int}} + E_{def} \qquad eq.\ 17$$

Non-covalent interaction (NCI) index

The NCI method[22-24] relies on two scalar fields to map local bonding properties: the electron density (ρ) and the reduced-density gradient (RDG, s) defined as:

$$s = \frac{1}{2(3\pi)^{1/3}} \frac{|\nabla \rho|}{\rho^{4/3}} \qquad eq.\ 18$$

The combination of s and ρ allows a rough partition of real space into bonding regions: high-s low-ρ corresponds to non-interacting density tails, low-s high-ρ to covalent bonds, and low-s low-ρ to non-covalent interactions. The NCI analysis was carried out at the BLYP-D3(BJ)/def2-SVP//GFN2-xTB level using Multiwfn program.[25]

Table S8. EDA results (kcal/mol) for C_{60}@(RuP-cor)$_2$·bpy and (C_{60})$_4$@(RuP-cor)$_2$·bpy complexes at the BLYP(D3BJ)/TZP//GFN2-xTB level of theory.[a]

Complex	ΔE_{int} components				ΔE_{int}	ΔE_{def}	$\Delta E_{complex}$
	ΔE_{Pauli}	ΔE_{elstat}	ΔE_{oi}	ΔE_{disp}			
	**C_{60}@(RuP-cor)$_2$·bpy**						
**C_{60}···(RuP-cor)$_2$·bpy (1-1s)**	91.8	-38.1 (28.0%)	-19.2 (14.0%)	-78.9 (58.0%)	-44.6	1.9	42.7
**C_{60}···(RuP-cor)$_2$·bpy (1-1t)**	94.4	-36.9 (26.8 %)	-20.1 (14.6%)	-80.6 (58.6%)	-43.1	6.7	36.4
	**(C_{60})$_4$@(RuP-cor)$_2$·bpy**						
**(C_{60})$_4$···(RuP-cor)$_2$·bpy**	359.8	-146.9 (27.4%)	-74.3 (13.8%)	-315.4 (58.8%)	-176.9	4.7	172.2

[a] Relative values (in parentheses) are given as a percentage and express the contribution to the sum of all attractive energy terms: $\Delta E_{elstat} + \Delta E_{oi} + \Delta E_{disp}$. Complexation energy: $\Delta E_{complex} = \Delta E_{int} + \Delta E_{def}$.

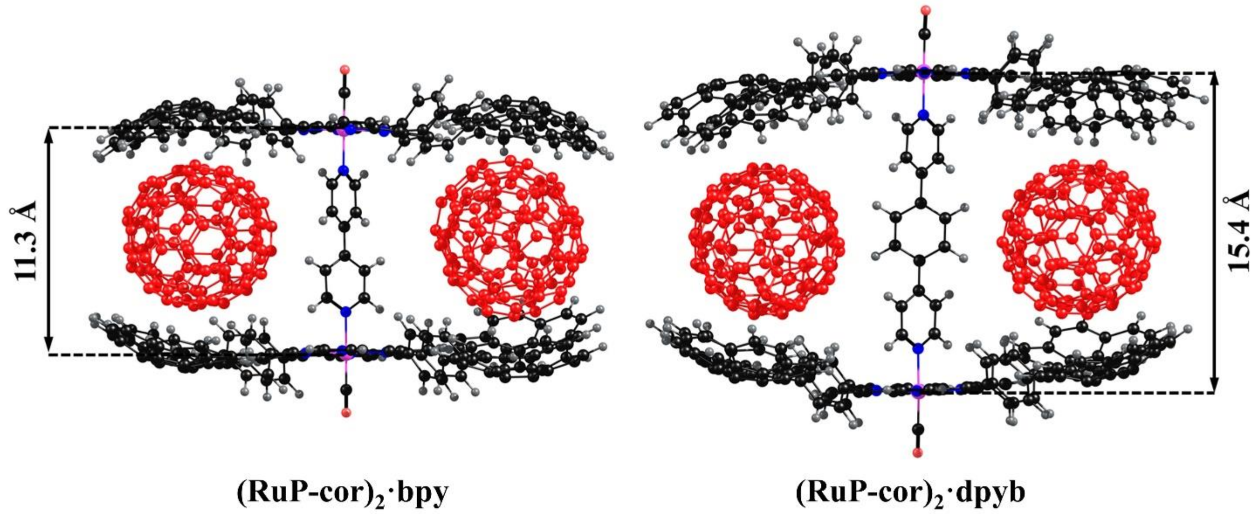

Figure S 123. Graphical representation of optimized structure of **(C_{60})$_4$@(RuP-cor)$_2$·bpy** and **(C_{60})$_4$@(RuP-cor)$_2$·dpyb** molecules.

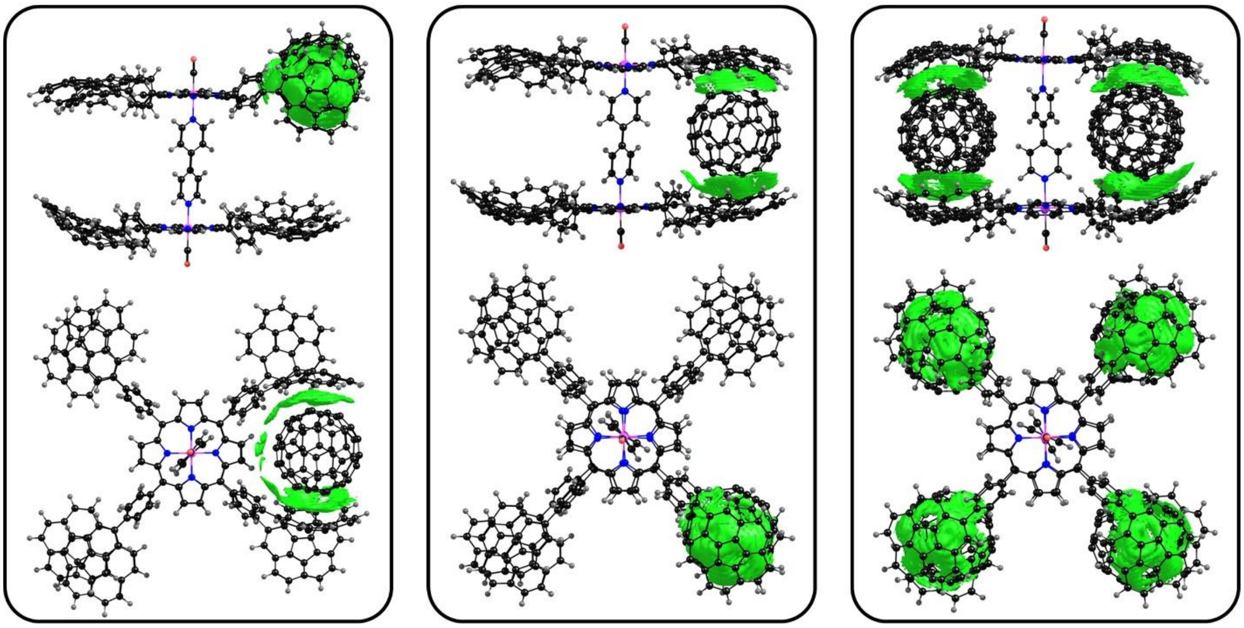

C_{60}@(RuP-cor)$_2$·bpy
Tweezer-like

C_{60}@(RuP-cor)$_2$·bpy
Sandwich-like

$(C_{60})_4$@(RuP-cor)$_2$·bpy

Figure S 124. NCI isosurfaces of van der Waals interactions for **C_{60}@(RuP-cor)$_2$·bpy** (tweezer-like), **C_{60}@(RuP-cor)$_2$·bpy** (sandwich-like), and **$(C_{60})_4$@(RuP-cor)$_2$·bpy** complexes.

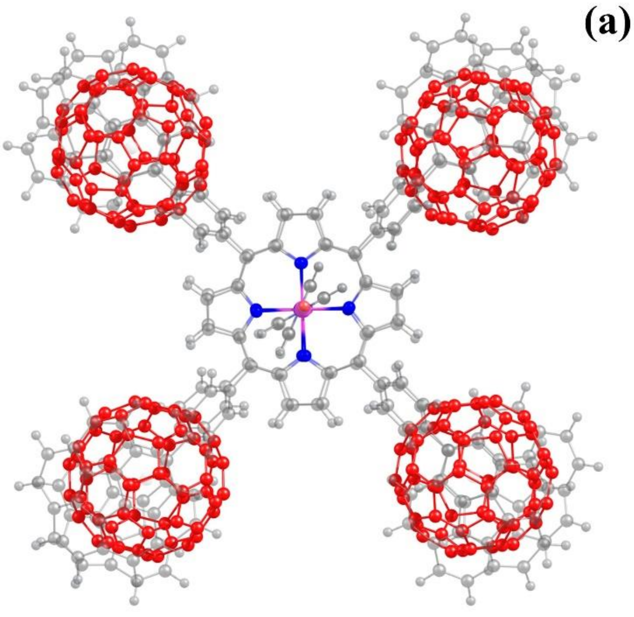

$(C_{60})_4$@(RuP-cor)$_2$·bpy

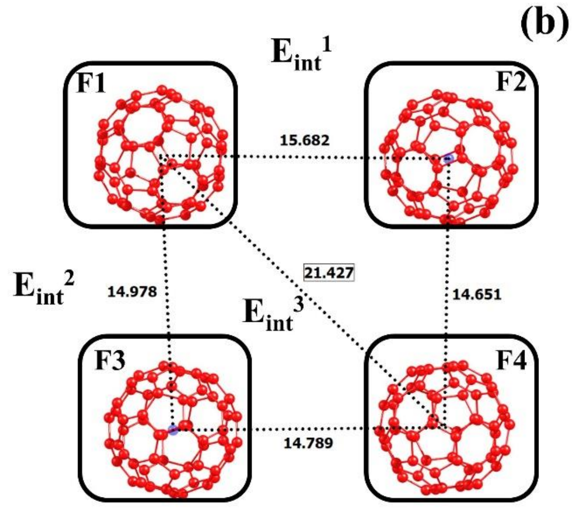

$$E^{1}_{\text{int}}(C^{F1}_{60}\cdots C^{F2}_{60}) = -0.22\,kcal/mol$$
$$E^{2}_{\text{int}}(C^{F1}_{60}\cdots C^{F3}_{60}) = -0.34\,kcal/mol$$
$$E^{3}_{\text{int}}(C^{F1}_{60}\cdots C^{F4}_{60}) = -0.02\,kcal/mol$$

Figure S 125. a) Graphical representation of **$(C_{60})_4$@(RuP-cor)$_2$·bpy** complex; (b) interaction energies between individual fullerenes in the complex.

Cartesian Coordinates

$(C_{60})_4$@(RuP-cor)$_2$·bpy

Gas-phase. GFN2-xTB

Atom	X	Y	Z
6	7.510100000	15.817700000	0.302900000
6	8.482800000	15.113400000	-0.415200000
6	8.814000000	13.814100000	-0.025500000
7	8.212800000	13.195800000	1.018800000
6	7.269300000	13.874900000	1.715100000
6	6.903000000	15.182000000	1.392800000
1	8.976500000	15.558600000	-1.278200000
1	9.581700000	13.219600000	-0.552200000
1	6.808300000	13.324300000	2.554800000
1	6.153500000	15.696100000	1.994300000
6	7.124100000	17.180600000	-0.072500000
6	8.068100000	18.099300000	-0.545000000
6	7.657300000	19.390200000	-0.882400000
7	6.372400000	19.799000000	-0.752500000
6	5.455800000	18.909200000	-0.301400000
6	5.792000000	17.597900000	0.036800000
1	9.118200000	17.825700000	-0.643800000
1	8.360200000	20.150000000	-1.267100000
1	4.423700000	19.292200000	-0.214600000
1	5.015100000	16.913200000	0.376500000
44	5.759500000	21.950500000	-1.172800000
6	8.722000000	22.696400000	-1.779500000
6	9.945900000	23.143500000	-1.107700000
1	10.880200000	23.284900000	-1.608600000
6	9.654000000	23.326900000	0.211500000
1	10.300400000	23.673700000	0.989600000
6	8.247800000	22.963900000	0.417100000
6	7.537900000	23.000300000	1.618800000
6	6.218200000	22.596400000	1.840300000
6	5.510600000	22.578000000	3.124800000
1	5.971100000	22.823300000	4.058800000
6	4.224100000	22.189800000	2.888200000
1	3.425900000	22.061500000	3.588000000
6	4.087700000	21.949200000	1.449200000
6	2.923100000	21.584600000	0.772900000
6	2.777200000	21.285500000	-0.583500000
6	1.549900000	20.848900000	-1.257700000
1	0.616300000	20.700600000	-0.757400000
6	1.835200000	20.687100000	-2.581100000
1	1.187000000	20.365900000	-3.368900000
6	3.243600000	21.037500000	-2.781900000
6	3.930100000	21.074600000	-3.993900000
6	5.266900000	21.422000000	-4.203400000
6	5.945500000	21.525100000	-5.497100000
1	5.470500000	21.334300000	-6.436600000
6	7.230500000	21.916000000	-5.262200000
1	8.001700000	22.109400000	-5.977600000
6	7.397300000	22.065100000	-3.812600000
6	8.557900000	22.473100000	-3.146700000
6	5.199200000	23.878200000	-1.500800000
7	7.724900000	22.583200000	-0.814100000
7	5.326500000	22.180100000	0.860500000
7	3.778800000	21.378200000	-1.543100000
7	6.180900000	21.764800000	-3.213900000
8	4.874300000	24.967200000	-1.680000000

$(C_{60})_4$@(RuP-cor)$_2$·dpyb

Gas-phase. GFN2-xTB

Atom	X	Y	Z
6	8.196103925	13.617034640	1.105136290
6	9.369038585	13.028137803	0.636766511
6	9.655149150	11.726075430	0.978229139
7	8.855698589	10.973862362	1.741760450
6	7.737459610	11.540660062	2.203368655
6	7.375578533	12.838750141	1.919306137
1	10.060331397	13.586801439	0.025357590
1	10.566710039	11.272338220	0.615560009
1	7.095782938	10.933840670	2.827590723
1	6.448870481	13.233853572	2.304709636
6	6.640606667	18.879813383	-0.380623490
6	7.418415288	19.686655093	-1.207901940
6	6.977624080	20.951517477	-1.527497810
7	5.826480259	21.463459907	-1.079985873
6	5.067556099	20.681154095	-0.307410134
6	5.428609057	19.404878145	0.061905133
1	8.371973019	19.341201437	-1.575352135
1	7.583923938	21.577965809	-2.166950865
1	4.125771854	21.087006355	0.034749596
1	4.766467352	18.815250131	0.675908762
44	5.301451183	23.403834964	-1.485310660
6	8.251369471	23.975767195	-2.043577303
6	9.480887946	24.332741931	-1.390688023
1	10.428779416	24.452572420	-1.877060252
6	9.202064188	24.474453524	-0.074114582
1	9.874199955	24.757743526	0.711459942
6	7.805220566	24.178273549	0.093717119
6	7.143771847	24.157909395	1.325903927
6	5.781919578	23.886541482	1.496958523
6	5.123435400	23.805216385	2.773459919
1	5.596352384	23.951457145	3.724675762
6	3.830141079	23.488448550	2.532678055
1	3.043964082	23.333128529	3.245658522
6	3.690705565	23.358792354	1.108033830
6	2.522906191	22.922583355	0.475082696
6	2.405619448	22.637504856	-0.888097918
6	1.223372896	22.097023595	-1.501971624
1	0.291966102	21.923700650	-0.999608954
6	1.528917864	21.857332838	-2.798292892
1	0.899665110	21.432834490	-3.555701762
6	2.890990669	22.275033329	-2.992054551
6	3.589852609	22.161321834	-4.195999099
6	4.915284056	22.555058450	-4.391613937
6	5.596607087	22.509287947	-5.656702290
1	5.153345161	22.206430736	-6.585109826
6	6.860049842	22.942267958	-5.439314509
1	7.647057575	23.063948437	-6.157436407
6	6.961619626	23.248662601	-4.037559363
6	8.138843871	23.660306562	-3.402939026
6	7.946150111	24.435493231	2.536690033
6	9.046429623	23.649644547	2.873406118
1	9.299145137	22.799814241	2.254660444
6	9.811996406	23.938879517	3.988061231
1	10.664027312	23.320861777	4.232970721
6	9.479601161	25.008059523	4.813198714

44	8.725900000	11.056400000	1.590000000
6	10.583100000	10.834300000	-0.899200000
6	10.548500000	10.476300000	-2.319700000
1	11.394300000	10.540300000	-2.970800000
6	9.280600000	10.070600000	-2.616800000
1	8.903900000	9.715400000	-3.552500000
6	8.472700000	10.193100000	-1.400100000
6	7.121200000	9.871600000	-1.252300000
6	6.331900000	10.018400000	-0.110200000
6	4.912700000	9.673200000	0.018600000
1	4.308300000	9.324600000	-0.792700000
6	4.549400000	9.879500000	1.316100000
1	3.596200000	9.706700000	1.768900000
6	5.721500000	10.386600000	2.038300000
6	5.795000000	10.703000000	3.396700000
6	6.888900000	11.219600000	4.097600000
6	6.912400000	11.630700000	5.505100000
1	6.050700000	11.629100000	6.138700000
6	8.188600000	12.005800000	5.808400000
1	8.576800000	12.371000000	6.735600000
6	9.002200000	11.838200000	4.602100000
6	10.376900000	12.037400000	4.486100000
6	11.158600000	11.921500000	3.334600000
6	12.611100000	12.097800000	3.246400000
1	13.233700000	12.343000000	4.081400000
6	12.979200000	11.877500000	1.952300000
1	13.961100000	11.904500000	1.529100000
6	11.768400000	11.554800000	1.189800000
6	11.706400000	11.220200000	-0.165100000
6	9.151700000	9.132100000	2.090900000
7	9.299700000	10.667600000	-0.388200000
7	6.772900000	10.465600000	1.130400000
7	8.167500000	11.392100000	3.582900000
7	10.691900000	11.588100000	2.068000000
8	9.383200000	8.040800000	2.373100000
6	8.231800000	23.600900000	2.773600000
6	9.396000000	23.037400000	3.297500000
1	9.789000000	22.113500000	2.876100000
6	10.049100000	23.659900000	4.356700000
1	10.974800000	23.238300000	4.745100000
6	9.516000000	24.817100000	4.926900000
6	8.346000000	25.378000000	4.407600000
1	7.937200000	26.286200000	4.850400000
6	7.713200000	24.777900000	3.327000000
1	6.809800000	25.217800000	2.906500000
6	1.678200000	21.556100000	1.565000000
6	1.041000000	20.344800000	1.837900000
1	1.496000000	19.408500000	1.521700000
6	-0.178500000	20.344700000	2.503400000
1	-0.687500000	19.402400000	2.698700000
6	-0.758200000	21.545600000	2.920500000
6	-0.104600000	22.754800000	2.673000000
1	-0.552900000	23.693100000	2.997000000
6	1.110200000	22.758800000	1.995100000
1	1.619800000	23.698600000	1.787900000
6	3.163500000	20.727900000	-5.205100000
6	3.526600000	19.601300000	-5.946900000
1	4.351800000	18.975300000	-5.610600000
6	2.836500000	19.289900000	-7.111000000
1	3.136500000	18.424900000	-7.700100000

6	8.360179736	25.776070141	4.493636548
1	8.096588359	26.611867733	5.126013891
6	7.614081564	25.504213380	3.365975228
1	6.773152803	26.131396596	3.108317651
6	1.351867822	22.627363742	1.334036029
6	0.937281574	21.312642618	1.526961093
1	1.480212831	20.508679197	1.050325771
6	-0.152217396	21.022393362	2.325881283
1	-0.445461245	19.995219441	2.482568343
6	-0.853977748	22.043796806	2.956717721
6	-0.430162646	23.358090505	2.773013061
1	-0.973361456	24.158439887	3.253378126
6	0.656469995	23.647605477	1.971451591
1	0.969354903	24.670974618	1.826161749
6	2.897022486	21.541540352	-5.345614109
6	3.360607561	20.338642586	-5.871626853
1	4.216736843	19.858877865	-5.418328075
6	2.734782688	19.751565885	-6.954005038
1	3.092030975	18.806758664	-7.335761116
6	1.625251924	20.355420231	-7.536961839
6	1.152003910	21.550578179	-6.999875819
1	0.293928749	22.027928288	-7.450809701
6	1.780307906	22.138874168	-5.920652749
1	1.419038593	23.076873906	-5.524968478
6	9.376122783	23.633112431	-4.212216611
6	9.791447478	22.431782461	-4.784460076
1	9.205499877	21.537448219	-4.624058544
6	10.939892222	22.369699500	-5.547145227
1	11.252453568	21.430542131	-5.980480295
6	11.719414182	23.505366102	-5.741379066
6	11.313577401	24.703416186	-5.155695550
1	11.905428982	25.594057724	-5.310777686
6	10.152566522	24.769969289	-4.411025897
1	9.830572800	25.710921418	-3.989409739
6	4.921617686	25.169712071	-1.874481689
7	7.256115948	23.901336342	-1.118037758
7	4.888613807	23.604169418	0.511545143
7	3.390600331	22.746638333	-1.819468602
7	5.759715174	23.033119168	-3.441376202
8	4.780708516	26.262477014	-2.192713437
44	9.310965461	9.018898936	2.125516801
6	11.082104384	9.028842262	-0.363257786
6	11.040735479	8.828311182	-1.786426540
1	11.875412926	8.921471311	-2.452338903
6	9.764143137	8.510910632	-2.104509236
1	9.370050199	8.268505230	-3.071765148
6	9.006458666	8.542786992	-0.883903537
6	7.625748911	8.329637028	-0.806531389
6	6.889047620	8.327486197	0.383784081
6	5.469185033	8.113804714	0.463986910
1	4.819146924	7.933144543	-0.368552855
6	5.128001714	8.197152627	1.770277347
1	4.156582881	8.061925529	2.203402942
6	6.329044339	8.507232176	2.496845215
6	6.366407472	8.786247803	3.867419678
6	7.503758964	9.185833413	4.580981170
6	7.478072958	9.640336337	5.945580165
1	6.619489387	9.622726753	6.587698089
6	8.715507993	10.109381629	6.228787552
1	9.061722671	10.543258980	7.146465331

6	1.760700000	20.079300000	-7.525800000
6	1.395600000	21.203100000	-6.781200000
1	0.567800000	21.829000000	-7.111300000
6	2.100000000	21.530000000	-5.626400000
1	1.830000000	22.412900000	-5.048300000
6	9.726500000	22.719600000	-4.011600000
6	10.223200000	21.680400000	-4.803800000
1	9.775400000	20.689900000	-4.738800000
6	11.272500000	21.919300000	-5.680400000
1	11.619300000	21.122600000	-6.337200000
6	11.881600000	23.176900000	-5.724400000
6	11.391400000	24.213300000	-4.926300000
1	11.849300000	25.201200000	-4.976300000
6	10.308800000	23.988000000	-4.081900000
1	9.908000000	24.800300000	-3.476400000
6	6.466900000	9.252900000	-2.420600000
6	6.349700000	9.949800000	-3.624700000
1	6.725200000	10.969200000	-3.699300000
6	5.755200000	9.338200000	-4.722300000
1	5.698300000	9.868200000	-5.671200000
6	5.226200000	8.050600000	-4.609500000
6	5.347800000	7.350500000	-3.406900000
1	4.950500000	6.339200000	-3.324900000
6	5.979400000	7.945200000	-2.320700000
1	6.093900000	7.398300000	-1.385600000
6	4.603600000	10.388500000	4.206500000
6	3.374100000	10.996400000	3.949400000
1	3.294800000	11.756200000	3.173800000
6	2.254800000	10.622300000	4.683000000
1	1.286900000	11.063300000	4.452700000
6	2.365000000	9.686700000	5.714700000
6	3.598900000	9.088900000	5.982200000
1	3.683900000	8.349800000	6.778700000
6	4.711000000	9.432300000	5.223100000
1	5.673000000	8.959100000	5.415300000
6	11.112200000	12.369400000	5.720400000
6	11.770100000	13.597200000	5.827000000
1	11.680600000	14.331100000	5.027600000
6	12.546500000	13.865100000	6.945800000
1	13.090600000	14.805400000	7.013400000
6	12.642300000	12.931200000	7.981600000
6	11.959100000	11.717900000	7.888200000
1	12.034800000	10.986500000	8.691300000
6	11.199000000	11.436300000	6.756500000
1	10.676500000	10.485100000	6.668800000
6	12.993100000	11.229100000	-0.885200000
6	13.735900000	12.408700000	-0.978200000
6	14.968100000	12.398400000	-1.619200000
6	15.438900000	11.228200000	-2.220100000
6	14.699200000	10.047600000	-2.124000000
1	15.077000000	9.130300000	-2.575600000
6	13.485700000	10.046500000	-1.445500000
1	12.912700000	9.125300000	-1.346500000
6	1.009500000	19.703700000	-8.730200000
6	1.717300000	19.611100000	-9.987800000
6	-0.322200000	19.347200000	-8.607800000
6	0.907300000	19.341500000	-11.068800000
6	3.128800000	19.523000000	-10.275100000
6	-1.089100000	18.841000000	-9.714200000
1	-0.801100000	19.380000000	-7.629700000

6	9.512181856	9.931553722	5.046226576
6	10.839925241	10.347891898	4.918100899
6	11.619433176	10.161392197	3.773278806
6	13.016351988	10.487058450	3.686564712
1	13.612885373	10.856703700	4.497650373
6	13.408082810	10.208611625	2.421274082
1	14.390082422	10.302374098	2.000689945
6	12.246985054	9.729464757	1.719986264
6	12.210454932	9.464535483	0.345299573
6	6.882443363	8.121524649	-2.069016089
6	6.906741523	9.076470974	-3.083798139
1	7.477779402	9.983282668	-2.941412139
6	6.206477475	8.884315828	-4.261102311
1	6.239384943	9.634606019	-5.038350242
6	5.435058221	7.741823270	-4.445641678
6	5.393139691	6.795148262	-3.423092345
1	4.804320839	5.899220998	-3.558922434
6	6.119073488	6.972086416	-2.262917228
1	6.110918193	6.208318747	-1.499515896
6	5.072852734	8.811816686	4.585391356
6	4.073704012	9.695942925	4.182193489
1	4.255095187	10.355777869	3.344830869
6	2.862506400	9.746219380	4.843057695
1	2.099809150	10.442453183	4.524856737
6	2.624388544	8.925403328	5.941022336
6	3.629481802	8.053192387	6.356094136
1	3.450853045	7.405957869	7.203156243
6	4.833215343	7.989609050	5.682296735
1	5.595915631	7.292267217	5.996648202
6	11.460733921	11.096007306	6.038154367
6	11.827460045	12.428661543	5.860131515
1	11.635152658	12.904870292	4.908028728
6	12.424658752	13.147867926	6.879839819
1	12.687797450	14.184633552	6.725953978
6	12.667845466	12.551269017	8.113257561
6	12.286331976	11.223528222	8.296364497
1	12.477290715	10.749166125	9.248919292
6	11.695428596	10.504078104	7.275267511
1	11.428112297	9.468412197	7.426162799
6	13.404252227	9.841727435	-0.443068481
6	13.846799368	11.163998063	-0.416115333
6	14.932485306	11.565984225	-1.168685090
6	15.591586467	10.658795917	-1.991789552
6	15.145617281	9.338784982	-2.026869078
1	15.661100779	8.624043192	-2.653070757
6	14.077180254	8.931299237	-1.252437245
1	13.763206045	7.897870119	-1.261706778
6	9.715874372	7.242984308	2.437687148
7	9.837224864	8.835693147	0.152000264
7	7.376927760	8.561043347	1.631617299
7	8.752177489	9.362768170	4.070508258
7	11.195684028	9.667728039	2.580126884
8	9.988887431	6.134575292	2.544716360
6	0.944264991	19.774140458	-8.707751197
6	1.682443933	19.514374111	-9.921461026
6	-0.397519563	19.458022600	-8.596307102
6	0.883341570	19.162769111	-10.990711903
6	3.084244105	19.361610523	-10.178257429
6	-1.147026605	18.854856862	-9.653308404
1	-0.867166564	19.594808168	-7.631114098

6	1.408700000	18.765100000	-12.281100000	6	1.376824995	18.504313052	-12.137146707
6	-0.466100000	18.962500000	-10.936800000	6	-0.484595013	18.843367419	-10.864136015
6	3.610100000	18.963700000	-11.438100000	6	3.564555247	18.710984802	-11.291066712
1	3.822400000	19.878400000	-9.512300000	1	3.793304670	19.704047679	-9.437254768
6	-2.273700000	18.017500000	-9.735000000	6	-2.346619312	18.070950928	-9.640694430
6	0.346400000	18.026900000	-12.893100000	6	0.315861914	17.774395313	-12.713478680
6	2.735000000	18.438800000	-12.456600000	6	2.701384675	18.145782101	-12.285384661
6	-0.813200000	18.149600000	-12.063100000	6	-0.834002473	17.981778993	-11.923441970
1	4.682500000	18.861200000	-11.591100000	1	4.630820632	18.549412368	-11.373900844
6	-2.605800000	17.233900000	-10.818900000	6	-2.682615800	17.223875102	-10.670719439
1	-2.882700000	17.985000000	-8.833500000	1	-2.964673792	18.081684093	-8.753081110
6	0.569600000	16.932200000	-13.699100000	6	0.510110958	16.636040808	-13.470142941
6	2.972500000	17.457400000	-13.485100000	6	2.915780886	17.103466668	-13.245615625
6	-1.794000000	17.184300000	-12.009900000	6	-1.862975302	17.065294319	-11.835474739
1	-3.476200000	16.581800000	-10.774700000	1	-3.554572227	16.593488783	-10.558902711
6	1.949100000	16.745100000	-14.072800000	6	1.881393060	16.389757290	-13.803906320
6	-0.562600000	16.049000000	-13.824300000	6	-0.646650553	15.793711547	-13.556343181
1	4.005900000	17.246400000	-13.753900000	1	3.930861735	16.805929094	-13.470728377
6	-1.680600000	16.168800000	-13.026500000	6	-1.767075715	15.996612874	-12.784620538
1	2.173500000	15.972200000	-14.805400000	1	2.117006129	15.553900060	-14.448510934
1	-0.489300000	15.232000000	-14.539900000	1	-0.604050452	14.914689630	-14.185642138
1	-2.491000000	15.447500000	-13.113900000	1	-2.567213492	15.270233295	-12.832179055
6	-2.043900000	21.522600000	3.627900000	6	-2.027379919	21.781046543	3.811263852
6	-3.173100000	20.882800000	2.989700000	6	-3.175390496	21.079616717	3.283610724
6	-2.110100000	22.016400000	4.919100000	6	-1.980845849	22.198220402	5.129250016
6	-4.350600000	20.990800000	3.695800000	6	-4.269282967	21.098625165	4.125673932
6	-3.240100000	19.967300000	1.875700000	6	-3.336861538	20.211428520	2.155135890
6	-3.297300000	21.913500000	5.723200000	6	-3.045024020	21.976020052	6.057946743
1	-1.217800000	22.436800000	5.382000000	1	-1.056914134	22.634932302	5.485548655
6	-5.440200000	20.077400000	3.517400000	6	-5.344112539	20.191426228	4.013348309
6	-4.413600000	21.495000000	5.032900000	6	-4.210848014	21.534076980	5.466005964
6	-4.285400000	19.086800000	1.706900000	6	-4.378506939	19.317937707	2.052635107
1	-2.403500000	19.960100000	1.176500000	1	-2.573581532	20.199906148	1.389318254
6	-3.462600000	21.936900000	7.156200000	6	-3.063435761	21.915919494	7.489810147
6	-6.170300000	20.014500000	4.746100000	6	-5.944428459	20.063933537	5.283437814
6	-5.394400000	19.028100000	2.626800000	6	-5.392875729	19.197683441	3.057056873
6	-5.538800000	20.893800000	5.682200000	6	-5.241551112	20.892939228	6.182141697
1	-4.270300000	18.373700000	0.885200000	1	-4.386938341	18.629007218	1.218614642
6	-4.548300000	21.362000000	7.780100000	6	-4.066409558	21.284689724	8.187905484
1	-2.665400000	22.382100000	7.749000000	1	-2.216148101	22.306371642	8.037243985
6	-6.876400000	18.897600000	5.135700000	6	-6.629132684	18.933280294	5.683032399
6	-6.320300000	17.963300000	2.918300000	6	-6.283872135	18.123410824	3.383720397
6	-5.592100000	20.692500000	7.043600000	6	-5.170060933	20.641847165	7.538028460
1	-4.613300000	21.356900000	8.866600000	1	-3.973710297	21.198129451	9.262143493
6	-7.020500000	17.901100000	4.104300000	6	-6.868415959	17.998934338	4.623364426
6	-7.135400000	18.827700000	6.552100000	6	-6.745062399	18.799362986	7.105483795
1	-6.414600000	17.161300000	2.188400000	1	-6.433744289	17.333492417	2.660014161
6	-6.527500000	19.675300000	7.453600000	6	-6.055278884	19.605854338	7.981182950
1	-7.667200000	17.050300000	4.310300000	1	-7.457282855	17.115572539	4.832079671
1	-7.794000000	18.037100000	6.907200000	1	-7.325516649	17.978449430	7.505257054
1	-6.704300000	19.552400000	8.520500000	1	-6.116515606	19.391406764	9.039735988
6	10.151200000	25.435300000	6.095100000	6	10.268044848	25.390623915	5.995711796
6	9.365200000	25.520000000	7.307400000	6	9.581670536	25.537037075	7.257937827
6	11.471800000	25.838500000	6.043800000	6	11.630913939	25.598807157	5.893706613
6	9.965700000	26.236200000	8.318100000	6	10.332520271	26.143137881	8.242522161
6	8.193000000	24.785500000	7.713000000	6	8.373630598	24.933921561	7.732466368
6	12.156100000	26.365800000	7.194700000	6	12.439153495	26.020466910	6.997883542
1	12.042300000	25.709100000	5.125100000	1	12.107138657	25.386601185	4.945273127
6	9.606300000	26.070800000	9.694500000	6	10.037785640	26.024927651	9.616983383
6	11.335300000	26.650400000	8.264100000	6	11.715707357	26.393355273	8.114258712

6	7.846600000	24.627700000	9.037000000	6	8.093253768	24.804983787	9.072981822
1	7.609700000	24.296900000	6.931600000	1	7.703349723	24.479486208	7.015233017
6	13.564700000	26.416000000	7.498000000	6	13.849574011	25.922220007	7.230212953
6	10.755400000	26.379800000	10.490700000	6	11.235267431	26.214661329	10.338599567
6	8.634700000	25.185400000	10.107200000	6	8.985750765	25.266781737	10.093015903
6	11.823500000	26.738200000	9.606900000	6	12.272211427	26.445982000	9.409231256
1	6.987700000	24.015900000	9.306500000	1	7.209179312	24.254507865	9.365292435
6	14.034100000	26.499600000	8.791000000	6	14.394809386	25.981551127	8.492392002
1	14.263600000	26.326200000	6.668300000	1	14.500847290	25.702373200	6.394725342
6	10.974400000	25.814200000	11.727500000	6	11.455597733	25.658964375	11.583463557
6	8.737700000	24.796100000	11.491600000	6	9.123993759	24.878801297	11.465624824
6	13.149200000	26.544800000	9.928400000	6	13.593748559	26.140629698	9.669696464
1	15.105000000	26.475600000	8.984500000	1	15.456035458	25.804885883	8.606376024
6	9.844100000	25.093000000	12.257600000	6	10.290782985	25.063069686	12.169061061
6	12.350400000	25.841000000	12.155900000	6	12.839861302	25.557041677	11.938016712
1	7.928800000	24.200100000	11.910600000	1	8.312932709	24.340065117	11.936985013
6	13.378500000	26.186800000	11.305500000	6	13.850399701	25.784518981	11.033631057
1	9.910900000	24.730800000	13.282000000	1	10.359809466	24.664136596	13.172200794
1	12.569100000	25.519300000	13.172400000	1	13.098246302	25.198458723	12.925429120
1	14.409900000	26.139000000	11.650500000	1	14.871288000	25.598821380	11.339851822
6	13.050500000	23.381600000	-6.585100000	6	12.944023177	23.474203493	-6.555390337
6	14.249700000	23.921900000	-5.980800000	6	14.164777042	23.995007869	-5.987350533
6	13.019100000	22.956400000	-7.901100000	6	12.922971604	22.908778070	-7.817245736
6	15.265800000	24.174600000	-6.875600000	6	15.203938212	24.103023993	-6.887492349
6	14.645000000	24.005600000	-4.596400000	6	14.557062828	24.160478069	-4.620585789
6	14.178200000	23.001800000	-8.751000000	6	14.078660036	22.834532668	-8.656976461
1	12.108400000	22.518000000	-8.307800000	1	11.999765492	22.456711861	-8.155142309
6	16.637700000	24.268700000	-6.474300000	6	16.556322245	24.180013821	-6.492520600
6	15.233200000	23.720300000	-8.232900000	6	15.161056322	23.551363023	-8.185580228
6	15.964200000	24.101300000	-4.210600000	6	15.876217067	24.225554462	-4.235239422
1	13.860700000	23.927000000	-3.842800000	1	13.793495702	24.139428050	-3.854944067
6	14.504700000	22.227700000	-9.922900000	6	14.388654022	21.982248534	-9.767265393
6	17.451000000	23.867500000	-7.582100000	6	17.349052971	23.685856979	-7.549471416
6	17.042800000	24.117200000	-5.166300000	6	16.955507850	24.134167655	-5.171434823
6	16.582900000	23.525700000	-8.667600000	6	16.486098518	23.297216986	-8.596320051
1	16.224300000	24.106200000	-3.153700000	1	16.104955785	24.257855472	-3.178816035
6	15.805000000	22.039200000	-10.338600000	6	15.680468813	21.741853300	-10.174400276
1	13.687400000	21.738900000	-10.450200000	1	13.584084012	21.441454906	-10.247576160
6	18.697200000	23.302400000	-7.423000000	6	18.594850787	23.122550637	-7.355706665
6	18.431900000	23.762900000	-5.012600000	6	18.325981189	23.754124177	-4.992706046
6	16.927600000	22.604400000	-9.632100000	6	16.814163594	22.320303918	-9.515515948
1	16.016600000	21.401300000	-11.194700000	1	15.849555487	21.019081727	-10.961419964
6	19.214700000	23.378900000	-6.079800000	6	19.100472039	23.277921639	-6.024093269
6	19.148700000	22.534400000	-8.556000000	6	19.026090463	22.284911197	-8.435375394
1	18.842000000	23.751300000	-4.004400000	1	18.739977429	23.761428775	-3.993489363
6	18.311500000	22.203200000	-9.599800000	6	18.184544116	21.905988709	-9.455906075
1	20.244100000	23.065200000	-5.916500000	1	20.098657084	22.925018262	-5.802437426
1	20.172900000	22.166200000	-8.540500000	1	20.021563712	21.862735231	-8.399948023
1	18.674400000	21.571900000	-10.408800000	1	18.546353286	21.197525827	-10.189083014
6	16.687700000	11.256000000	-2.987900000	6	16.760186216	11.037637572	-2.801888949
6	16.618200000	10.894100000	-4.387000000	6	16.736897793	10.768475217	-4.221288148
6	17.850700000	11.723100000	-2.404800000	6	17.847307586	11.646750892	-2.208758159
6	17.841100000	10.849300000	-5.017700000	6	17.963535434	10.892130007	-4.837413632
6	15.487500000	10.820800000	-5.278900000	6	15.635610296	10.667752954	-5.124807606
6	19.067200000	11.894500000	-3.152400000	6	19.034621412	11.992039213	-2.930705562
1	17.845300000	12.032100000	-1.360300000	1	17.774307956	11.907477823	-1.161352081
6	17.977200000	10.973200000	-6.438100000	6	18.112035326	11.062253107	-6.230041153
6	19.042300000	11.341300000	-4.413800000	6	19.087205190	11.467639245	-4.207197710
6	15.619200000	10.934100000	-6.645600000	6	15.777330586	10.857099665	-6.481373014
1	14.497800000	10.730600000	-4.830000000	1	14.642552344	10.538180572	-4.717672218

6	20.200800000	12.749600000	-2.901800000	6	20.072566069	12.937720470	-2.648196915
6	19.260700000	11.545500000	-6.710200000	6	19.336589597	11.722329405	-6.461791318
6	16.898100000	11.140700000	-7.277800000	6	17.036233044	11.158664557	-7.089184808
6	19.919100000	11.772700000	-5.459500000	6	19.937899318	11.975841879	-5.210781157
1	14.735900000	10.927500000	-7.281100000	1	14.888582013	10.873294581	-7.095694372
6	21.045100000	13.163800000	-3.908900000	6	20.912374399	13.424094961	-3.622446208
1	20.343400000	13.112700000	-1.885700000	1	20.143398577	13.348776743	-1.649599690
6	19.512500000	12.306400000	-7.830400000	6	19.567890365	12.504229743	-7.576406193
6	17.226900000	11.718600000	-8.557000000	6	17.347923805	11.780941755	-8.343554777
6	20.853000000	12.769900000	-5.282500000	6	20.804303094	13.030225898	-4.997058533
1	21.857000000	13.854900000	-3.689300000	1	21.619566614	14.199092790	-3.357159197
6	18.462700000	12.270600000	-8.817700000	6	18.545104377	12.412698258	-8.574253609
6	20.657000000	13.174400000	-7.710400000	6	20.641412411	13.436538814	-7.418608104
1	16.441900000	11.761500000	-9.309800000	1	16.579943140	11.833210962	-9.102182879
6	21.290600000	13.393500000	-6.506000000	6	21.227328586	13.685664268	-6.198898804
1	18.654500000	12.749800000	-9.775900000	1	18.683555138	12.938454624	-9.509847606
1	20.982800000	13.707100000	-8.601800000	1	20.939180023	14.032703922	-8.270907719
1	22.116500000	14.099900000	-6.445100000	1	21.970511093	14.466837014	-6.131041303
6	4.511100000	7.447000000	-5.739500000	6	4.706741109	7.452013497	-5.693227950
6	3.112600000	7.125200000	-5.549900000	6	3.295031545	7.146783595	-5.630700049
6	5.141400000	7.292400000	-6.959800000	6	5.381768938	7.454433663	-6.898643827
6	2.541800000	6.452100000	-6.606400000	6	2.788664011	6.593150446	-6.787435775
6	2.167600000	7.599800000	-4.569500000	6	2.292407617	7.547526939	-4.693141291
6	4.450700000	6.805500000	-8.124400000	6	4.764245115	7.099537366	-8.141160228
1	6.180700000	7.597500000	-7.074900000	1	6.415069310	7.774734170	-6.898669490
6	1.128200000	6.439400000	-6.837200000	6	1.412688819	6.570140731	-7.096877874
6	3.196900000	6.294900000	-7.869800000	6	3.505833666	6.548351026	-8.001896791
6	0.807600000	7.586600000	-4.791200000	6	0.951085155	7.548114375	-5.003734334
1	2.569500000	8.034300000	-3.653600000	1	2.605578670	7.958138035	-3.742571076
6	4.744700000	6.983500000	-9.524700000	6	5.122088363	7.388211628	-9.498003918
6	0.910800000	6.275800000	-8.242600000	6	1.279364465	6.490286901	-8.499081666
6	0.235100000	7.097900000	-6.020700000	6	0.452801489	7.135552223	-6.281687586
6	2.189400000	6.186400000	-8.880700000	6	2.574963839	6.477477629	-9.058234558
1	0.131000000	8.008000000	-4.050200000	1	0.256981916	7.960941171	-4.285154198
6	3.773700000	6.880300000	-10.497300000	6	4.217396824	7.311713538	-10.531467636
1	5.760900000	7.264000000	-9.796200000	1	6.116019747	7.761685411	-9.705903791
6	-0.205600000	6.765600000	-8.883800000	6	0.174525197	6.965404873	-9.176650267
6	-1.034200000	7.384200000	-6.641500000	6	-0.780212429	7.423375903	-6.954386996
6	2.398100000	6.583600000	-10.183100000	6	2.846543156	6.944496413	-10.329411969
1	4.021600000	7.080900000	-11.537900000	1	4.530611616	7.627937529	-11.518076877
6	-1.242300000	7.228900000	-7.995200000	6	-0.910787192	7.344790974	-8.320907649
6	-0.041500000	6.943700000	-10.304700000	6	0.412914630	7.222809674	-10.565480886
1	-1.825100000	7.788800000	-6.012500000	1	-1.607588099	7.812748062	-6.377330055
6	1.189300000	6.857100000	-10.919200000	6	1.675413095	7.211823761	-11.110522965
1	-2.196600000	7.512600000	-8.435700000	1	-1.839740597	7.667338616	-8.772016245
1	-0.922700000	7.209800000	-10.885700000	1	-0.417026347	7.528062775	-11.188684994
1	1.280200000	7.054300000	-11.985800000	1	1.795952987	7.507113115	-12.143980855
6	1.193100000	9.368000000	6.536000000	6	1.343784135	8.953922057	6.664321680
6	1.308600000	9.538800000	7.968100000	6	1.362719221	9.144531505	8.095229571
6	-0.004300000	9.034400000	5.929600000	6	0.152243225	8.856301978	5.968967867
6	0.194600000	9.144500000	8.675100000	6	0.138590202	8.964571594	8.704388769
6	2.275200000	10.274500000	8.745800000	6	2.352921424	9.743316336	8.937686477
6	-1.220400000	8.862300000	6.676900000	6	-1.127362963	8.889525762	6.607394326
1	-0.058500000	8.956400000	4.844600000	1	0.198002382	8.804088393	4.888819392
6	-0.094800000	9.638800000	9.988400000	6	-0.170091013	9.495292864	9.975461245
6	-1.047400000	8.817300000	8.042700000	6	-1.068708151	8.825315361	7.986519724
6	2.000400000	10.741600000	10.012000000	6	2.048975400	10.269613254	10.171637859
1	3.229600000	10.507700000	8.272500000	1	3.355689446	9.869696050	8.552335840
6	-2.593700000	8.983800000	6.254600000	6	-2.430802656	9.198653048	6.095604970
6	-1.515800000	9.624900000	10.162100000	6	-1.567700803	9.672873355	10.046407011

6	0.721700000	10.534000000	10.643800000	6	0.720610958	10.256918630	10.705363230
6	-2.104500000	9.117600000	8.960000000	6	-2.123997965	9.256129776	8.817769121
1	2.738800000	11.337600000	10.545300000	1	2.824490781	10.789749426	10.716854340
6	-3.610500000	9.272200000	7.138700000	6	-3.465303065	9.602297773	6.907329701
1	-2.804100000	8.896700000	5.190400000	1	-2.582887778	9.196083662	5.023612458
6	-2.171700000	10.504300000	10.995200000	6	-2.162860259	10.621923852	10.854473681
6	0.067300000	11.275500000	11.692800000	6	0.100721834	11.080730380	11.701008598
6	-3.369800000	9.471800000	8.546100000	6	-3.313148660	9.757695891	8.324727884
1	-4.626300000	9.412000000	6.773900000	1	-4.396666170	9.907338253	6.447972277
6	-1.300600000	11.261700000	11.858900000	6	-1.261171422	11.253326698	11.770638411
6	-3.569900000	10.677300000	10.691200000	6	-3.495654488	10.967239415	10.460650560
1	0.687000000	11.905300000	12.328900000	1	0.730971367	11.655749871	12.364894062
6	-4.136200000	10.189600000	9.533000000	6	-4.040622989	10.557901535	9.265380153
1	-1.762600000	11.879600000	12.626600000	1	-1.659701272	11.957626138	12.488465345
1	-4.169800000	11.263000000	11.385400000	1	-4.060030213	11.649163270	11.083681069
1	-5.182700000	10.390900000	9.311400000	1	-5.017587532	10.928813444	8.986988766
6	13.458500000	13.250000000	9.159500000	6	13.342399736	13.248165618	9.229806078
6	14.859800000	13.547600000	8.959800000	6	14.714231569	13.690197229	9.085170791
6	12.849600000	13.368400000	10.396200000	6	12.647049269	13.444519267	10.408847628
6	15.561300000	13.756000000	10.126700000	6	15.308535205	14.040172594	10.280684665
6	15.591800000	13.890500000	7.764300000	6	15.503003279	14.019215998	7.935393197
6	13.560400000	13.816600000	11.563700000	6	13.223693088	14.058636530	11.567248338
1	11.779300000	13.187900000	10.489800000	1	11.596383240	13.179579094	10.423181906
6	16.787100000	14.497100000	10.164300000	6	16.482321119	14.818915619	10.363470445
6	14.926800000	13.889000000	11.402000000	6	14.594272148	14.208866502	11.485475771
6	16.764500000	14.611500000	7.800900000	6	16.636387999	14.798074269	8.014294926
1	15.162000000	13.596600000	6.806200000	1	15.154179016	13.716422765	6.957424308
6	13.076200000	14.430800000	12.776200000	6	12.637563815	14.723201972	12.693719742
6	16.906700000	15.088800000	11.461500000	6	16.495968706	15.461452195	11.618995810
6	17.352900000	15.056100000	9.039700000	6	17.128027985	15.323872065	9.253080880
6	15.757900000	14.712100000	12.227500000	6	15.325847936	15.085698214	12.312295966
1	17.252300000	14.907300000	6.874200000	1	17.128593839	15.087578187	7.094898425
6	13.876900000	15.223700000	13.569800000	6	13.352299570	15.580053562	13.501127916
1	12.025600000	14.296400000	13.026700000	1	11.573407090	14.616938898	12.864603646
6	17.593700000	16.262500000	11.682600000	6	17.157532662	16.651476069	11.850798496
6	18.263300000	16.139700000	9.309600000	6	17.980587595	16.440166400	9.539286111
6	15.255600000	15.493200000	13.245000000	6	14.735885336	15.879543440	13.276183598
1	13.459700000	15.717100000	14.445900000	1	12.822625419	16.120310797	14.274875805
6	18.378000000	16.709900000	10.559400000	6	17.996407117	17.065138695	10.766041515
6	17.220000000	16.947600000	12.894500000	6	16.686952058	17.359051468	13.006521784
1	18.829200000	16.544800000	8.472700000	1	18.565847356	16.865921780	8.735732202
6	16.115000000	16.583400000	13.633800000	6	15.542960481	16.994933885	13.678470165
1	19.034500000	17.564800000	10.710400000	1	18.591744046	17.960854388	10.882538804
1	17.811000000	17.813700000	13.186900000	1	17.196808777	18.265966676	13.304765694
1	15.834500000	17.160700000	14.512900000	1	15.193074819	17.629427387	14.481699306
1	13.350300000	13.329000000	-0.541900000	1	13.325670018	11.884903886	0.199145670
1	15.565400000	13.307800000	-1.656400000	1	15.261277261	12.595046952	-1.141159140
6	16.934628228	20.766295081	-6.254670711	6	16.741601317	20.696537812	-6.485891344
6	17.031771764	20.854875002	-4.802316672	6	16.845070986	20.853952274	-5.058200832
6	18.039369138	20.187564978	-4.138059349	6	17.873652138	20.229703578	-4.364860443
6	19.005759614	19.394641772	-4.890058729	6	18.838548846	19.426511480	-5.072695963
6	18.914929292	19.313813052	-6.264884382	6	18.738811451	19.275839991	-6.448785042
6	17.849587861	20.020243938	-6.967017445	6	17.670622128	19.922702033	-7.167543385
6	15.522660961	20.720868342	-6.610400205	6	15.344487893	20.608300292	-6.824031479
6	14.746436421	20.779555470	-5.377573332	6	14.582613897	20.712728462	-5.606318129
6	15.677789147	20.864219046	-4.259396032	6	15.510482670	20.863741567	-4.513349056
6	15.400207008	20.203318125	-3.080782717	6	15.254507003	20.246011379	-3.296375070
6	17.746129612	19.485936404	-2.893177568	6	17.607370862	19.589430019	-3.101160692
6	19.311334225	18.201227322	-4.108840453	6	19.167909686	18.290750483	-4.247217713
6	19.510212930	16.993251291	-4.745531760	6	19.386087960	17.047309010	-4.825971653

6	19.414002748	16.908986977	-6.199408800
6	19.124707173	18.037148664	-6.939022291
6	18.190423152	17.956833295	-8.057571820
6	17.402539550	19.184080719	-8.075253071
6	16.065186240	19.141975623	-8.412164931
6	15.099094877	19.933317556	-7.659700000
6	13.589406556	20.040078699	-5.257801567
6	13.141947443	19.202555938	-6.365714093
6	13.873661079	19.152139502	-7.533675320
6	14.081902131	17.876180773	-8.208001164
6	15.436497858	17.869748913	-8.750904893
6	16.180596119	16.708125186	-8.733013170
6	17.595424802	16.753325417	-8.377031134
6	17.900412127	15.561013993	-7.594669639
6	18.783156251	15.637976981	-6.537075779
6	18.532854218	18.257642495	-2.875492321
6	16.553556362	14.259560806	-3.941662205
6	16.979274575	15.048006964	-2.893193213
6	16.011702071	15.839404417	-2.141498562
6	14.673661158	15.795807251	-2.477398021
6	14.224805298	14.957046695	-3.584018941
6	15.042876715	14.122322433	-5.748229617
6	16.397318614	14.120204934	-6.289846215
6	17.329625420	14.203106759	-5.173081332
6	18.489085448	14.939150149	-5.291823276
6	18.939875512	15.774078167	-4.184331206
6	18.205019146	15.826506172	-3.017688012
6	16.640404324	17.110760015	-1.802652356
6	15.895796207	18.272155081	-1.820376348
6	14.480978170	18.226845116	-2.175458845
6	13.886225478	17.022658400	-2.494308702
6	12.951171244	16.941723167	-3.612096032
6	13.160226972	15.664283259	-4.285775563
6	13.070752430	15.584075600	-5.661125181
6	14.036309825	14.790307727	-6.413777251
6	16.674999197	14.780294945	-7.468174073
6	15.611186295	15.487528775	-8.171853718
6	14.329895294	15.492856240	-7.658512254
6	13.544446648	16.721635505	-7.676027104
6	12.766700799	16.777588746	-6.442284429
6	12.569457396	17.985291132	-5.804771405
6	12.663082628	18.070673213	-4.350850478
6	13.292395985	19.342950337	-4.012710087
6	14.174655654	19.421344285	-2.954519786
6	17.995602642	17.103228535	-2.344315964
6	16.464541441	19.493343683	-2.380091686
6	15.139427069	14.209483229	-4.296246305
6	2.579039694	15.831395727	-10.554680196
6	2.795310954	16.420347836	-9.239354757
6	3.915234235	16.086149434	-8.506766646
6	4.881177150	15.138725280	-9.049736633
6	4.676011107	14.580349009	-10.295417647
6	3.493485903	14.937485172	-11.071127838
6	1.150610069	15.583964868	-10.706684256
6	0.484181060	16.021555503	-9.486947615
6	1.499891621	16.537947982	-8.579394999
6	1.390889972	16.317003841	-7.222384992
6	3.800743931	15.847864716	-7.071729581
6	5.363663819	14.312133093	-7.948901428
6	5.616050384	12.971500020	-8.154433222

6	19.283810004	16.890818994	-6.254689857
6	18.966439472	17.984544523	-7.049389721
6	18.038805828	17.833551691	-8.141806202
6	17.238524847	19.029888277	-8.213318247
6	15.893148406	18.945246742	-8.539352668
6	14.928806367	19.749488681	-7.831861900
6	13.433362802	19.951112864	-5.439797956
6	13.002084944	19.058108238	-6.486689016
6	13.735905093	18.960159060	-7.660882472
6	13.962997435	17.669134497	-8.261665313
6	15.297237635	17.660537290	-8.805562063
6	16.067511652	16.507010563	-8.735714717
6	17.465092920	16.594773535	-8.397188450
6	17.795015254	15.460020492	-7.571211929
6	18.687453487	15.605230586	-6.519151366
6	18.407038846	18.392044843	-3.028054322
6	16.513791964	14.303413390	-3.868628799
6	16.929335809	15.164607382	-2.861543624
6	15.964219123	15.969054278	-2.154164388
6	14.617170325	15.884161957	-2.479298128
6	14.185295662	14.991869437	-3.525933219
6	15.012941901	14.062028813	-5.637175995
6	16.347122289	14.053216770	-6.180903301
6	17.274706045	14.202499014	-5.088686266
6	18.419785437	14.967307804	-5.255237240
6	18.852481971	15.858837958	-4.209599970
6	18.121666619	15.955999617	-3.034404853
6	16.560093122	17.255232097	-1.889659423
6	15.789790964	18.408321658	-1.960492849
6	14.391880966	18.320504480	-2.298664018
6	13.817572347	17.082097366	-2.553063847
6	12.890250917	16.931327034	-3.645540187
6	13.117736157	15.640639039	-4.246503113
6	13.019434515	15.491085904	-5.622859456
6	13.984547149	14.687359658	-6.330524429
6	16.602860634	14.669787489	-7.398315209
6	15.535268284	15.317587360	-8.118448828
6	14.250066850	15.326401648	-7.594791880
6	13.450233880	16.523899345	-7.667776328
6	12.689267644	16.625577568	-6.448725777
6	12.469528201	17.868624274	-5.869828925
6	12.571893891	18.024880332	-4.440924067
6	13.166933618	19.311228821	-4.175472435
6	14.061096918	19.455706701	-3.123636337
6	17.893795791	17.246649390	-2.434098495
6	16.322412678	19.597595508	-2.576626433
6	15.115210231	14.216501548	-4.207179671
6	2.555731669	15.837875759	-10.377496376
6	2.778484321	16.409672311	-9.074269833
6	3.897423831	16.042044546	-8.338695947
6	4.836921028	15.091550647	-8.879300210
6	4.622162978	14.542969788	-10.135916471
6	3.460591980	14.923981816	-10.898625308
6	1.141033067	15.621745041	-10.533104388
6	0.487474754	16.059092965	-9.326932793
6	1.499079755	16.545714088	-8.423625882
6	1.387204695	16.307083834	-7.060488555
6	3.780914720	15.794576114	-6.922997155
6	5.299983058	14.256876579	-7.799493743
6	5.533034118	12.905216656	-8.015642795

6	5.399761920	12.380893416	-9.471508452	6	5.310985924	12.335893755	-9.320794770
6	4.942070415	13.162538850	-10.512279840	6	4.864468044	13.140057230	-10.360929497
6	3.924531856	12.645600524	-11.422014787	6	3.852214461	12.654212087	-11.264373640
6	3.028302029	13.743496732	-11.767610323	6	2.985249402	13.757109105	-11.596882637
6	1.675756751	13.510258176	-11.912895223	6	1.621489579	13.548051216	-11.746604308
6	0.709892572	14.457561823	-11.368108916	6	0.682952639	14.497602199	-11.204517103
6	-0.589164846	15.311616117	-8.991586701	6	-0.600564648	15.352265214	-8.834404499
6	-1.053950727	14.118265615	-9.688872349	6	-1.076335379	14.183062184	-9.530631838
6	-0.422920858	13.703137169	-10.843796177	6	-0.446701813	13.763814034	-10.693957747
6	-0.156274039	12.286040093	-11.062440827	6	-0.205948241	12.360580537	-10.919193090
6	1.140081826	12.166899968	-11.722699507	6	1.073296984	12.226635785	-11.569855534
6	1.988384812	11.128693657	-11.396501543	6	1.908041379	11.164139295	-11.248495191
6	3.418977363	11.374381532	-11.242105665	6	3.324543826	11.381694977	-11.093209619
6	3.902961181	10.548886708	-10.141483652	6	3.787731874	10.547463089	-10.012687321
6	4.865639483	11.036902866	-9.281315155	6	4.763693951	11.014301763	-9.143433631
6	4.696590387	14.750549469	-6.726865050	6	4.647234826	14.691054833	-6.590259840
6	3.012199035	10.525662035	-6.127885562	6	2.906912554	10.494369350	-6.024975812
6	3.452966364	11.652501193	-5.466246947	6	3.365127178	11.618584960	-5.352377207
6	2.487046346	12.600486750	-4.922894531	6	2.424645014	12.569488053	-4.812893724
6	1.134270742	12.367956459	-5.070060218	6	1.060640819	12.360159714	-4.964511654
6	0.668555763	11.173557891	-5.766099360	6	0.584518870	11.191927615	-5.661242756
6	1.367863201	9.693582637	-7.599112858	6	1.270746236	9.714590946	-7.488788420
6	2.661345553	9.575312417	-8.258580015	6	2.547177927	9.578098803	-8.137892320
6	3.677704397	10.090307610	-7.350741892	6	3.559434794	10.060498646	-7.234302805
6	4.750677859	10.800170138	-7.847715735	6	4.647460334	10.766149025	-7.728143733
6	5.214941910	11.994001168	-7.149623209	6	5.122903522	11.935212988	-7.031086976
6	4.584578012	12.408326096	-5.993474252	6	4.494513786	12.353388617	-5.865348071
6	3.021744706	13.944225779	-5.115119926	6	2.972318914	13.890585645	-4.992041484
6	2.173969930	14.982696305	-5.441942363	6	2.137857992	14.953243555	-5.313376540
6	0.743374374	14.737234908	-5.596650155	6	0.721965451	14.735564866	-5.469801837
6	0.238399687	13.465962061	-5.416764029	6	0.194284259	13.462953289	-5.298651446
6	-0.779491801	12.949156415	-6.326124935	6	-0.818000756	12.976710392	-6.201547430
6	-0.513498665	11.531319262	-6.542328974	6	-0.576926089	11.573311264	-6.425435768
6	-0.718706497	10.974319617	-7.788302610	6	-0.790251305	11.025707429	-7.682555591
6	0.248750011	10.027819748	-8.331492602	6	0.150746192	10.077411154	-8.223248095
6	2.770064404	9.796273769	-9.615212796	6	2.658635696	9.815878011	-9.499447443
6	1.587490532	10.152309821	-10.390151390	6	1.497739764	10.196332233	-10.263204312
6	0.362075209	10.265063243	-9.765999958	6	0.266878325	10.325934513	-9.636902447
6	-0.535676256	11.361035685	-10.111364809	6	-0.601209513	11.426411812	-9.971088438
6	-1.202787557	11.799557628	-8.889349396	6	-1.254571057	11.860731595	-8.762042355
6	-1.455186198	13.140335551	-8.683727073	6	-1.487187106	13.212747195	-8.546596665
6	-1.237826520	13.730874981	-7.366794143	6	-1.264543430	13.781415250	-7.241334637
6	-0.703578022	15.074818372	-7.556943265	6	-0.716734498	15.103220770	-7.419316727
6	0.258702857	15.564008185	-6.696814025	6	0.258076109	15.571588942	-6.548928651
6	4.318065568	13.825016606	-5.775699903	6	4.251660438	13.756900069	-5.642324135
6	2.574533358	15.959691713	-6.447887267	6	2.548777413	15.924148175	-6.296365478
6	1.583697662	10.279585796	-6.281333663	6	1.490578688	10.279058761	-6.183220232
6	-0.472835857	18.892681688	5.582845469	6	-0.344476458	18.798109827	5.150940992
6	-0.424202836	19.058193847	7.031150330	6	-0.365585392	19.018478148	6.575305989
6	0.603734955	18.488347441	7.753871627	6	0.629501545	18.472359513	7.375807723
6	1.641473958	17.724488701	7.068898147	6	1.682997991	17.686100795	6.784503302
6	1.596534053	17.571825752	5.697865152	6	1.703659444	17.474366281	5.413012840
6	0.508433972	18.171535916	4.934420838	6	0.671328909	18.040906782	4.581197678
6	-1.869679898	18.730045581	5.195910582	6	-1.705831086	18.631851615	4.707173352
6	-2.683291756	18.795983442	6.402801274	6	-2.567648682	18.748987493	5.855699411
6	-1.791062524	18.999269677	7.538435057	6	-1.739313961	18.988315487	7.010321099
6	-2.058634417	18.377781659	8.740593234	6	-2.065854705	18.412289523	8.230653603
6	0.321844462	17.829428486	9.024746975	6	0.289802537	17.875438212	8.642918846
6	2.001929399	16.593809494	7.917345517	6	1.993156999	16.604149685	7.685286225
6	2.299429258	15.371930109	7.349616265	6	2.312253991	15.349305266	7.184082440

6	2.251046367	15.210760223	5.899564695	6	2.333425884	15.128875851	5.760249319
6	1.909265323	16.279534247	5.096503297	6	2.034667377	16.171373985	4.892107892
6	1.014461139	16.082340953	3.960560364	6	1.207128855	15.931783613	3.736910840
6	0.148036623	17.249967818	3.861919043	6	0.364839560	17.086551673	3.544889848
6	-1.173986596	17.099497056	3.494495879	6	-0.946613835	16.925016388	3.118169933
6	-2.211147993	17.860948627	4.181326330	6	-2.000480901	17.711391629	3.710003490
6	-3.792816567	17.987647138	6.530152031	6	-3.691042982	17.942603886	5.964515808
6	-4.154043491	17.069053605	5.457638675	6	-3.996318575	16.987759057	4.929989957
6	-3.385198275	17.008352540	4.314221576	6	-3.166704172	16.874238911	3.824106204
6	-3.076401862	15.717896719	3.709373937	6	-2.835530721	15.572265398	3.302368137
6	-1.708911916	15.775530527	3.202395192	6	-1.462715514	15.602629760	2.866194675
6	-0.889895364	14.669750097	3.298372814	6	-0.651038177	14.490961297	3.050747854
6	0.507905691	14.827792682	3.688458414	6	0.709525210	14.658337185	3.494590517
6	0.868263229	13.700170022	4.538429336	6	1.020917100	13.576297615	4.395581632
6	1.716052802	13.884600762	5.611783002	6	1.818559134	13.805950057	5.508331752
6	1.186162268	16.658748959	9.125902745	6	1.131821793	16.721096696	8.834191696
6	-0.493386585	12.490736133	8.216617257	6	-0.506497432	12.516983425	8.036911819
6	-0.152032662	13.355724975	9.234239284	6	-0.212036149	13.436603402	9.034642360
6	-1.190528911	14.115640425	9.920360946	6	-1.265700791	14.224819571	9.624343701
6	-2.512272365	13.966974968	9.553058959	6	-2.575964296	14.065841586	9.195300440
6	-2.872143601	13.048481535	8.478441291	6	-2.881028975	13.112732544	8.159006071
6	-1.939578910	12.167541680	6.381343732	6	-1.847005070	12.134738114	6.166877823
6	-0.573933536	12.227918338	5.876166599	6	-0.473716381	12.163378287	5.730947491
6	0.321532461	12.425108783	7.010174609	6	0.355523837	12.399781958	6.886853176
6	1.436387016	13.226930667	6.883604025	6	1.479741334	13.207173568	6.776250611
6	1.797011525	14.144730735	7.958230846	6	1.784860186	14.162967215	7.811709173
6	1.024623724	14.207307530	9.100319101	6	0.954947804	14.275321102	8.918874019
6	-0.656354957	15.441952914	10.208893632	6	-0.750204426	15.548008146	9.873924792
6	-1.474629965	16.548475335	10.112189480	6	-1.561454452	16.660119729	9.688683724
6	-2.872491328	16.391608846	9.723807045	6	-2.922132752	16.493971884	9.244761454
6	-3.377897724	15.136405519	9.452223862	6	-3.418613045	15.220225268	9.003305154
6	-4.272703817	14.938280172	8.316159179	6	-4.246393401	14.981217174	7.847945509
6	-3.960864076	13.646451860	7.713703736	6	-3.914027341	13.678516437	7.327640330
6	-4.007581247	13.492409483	6.342870805	6	-3.894574725	13.466625634	5.956294778
6	-2.969405485	12.730892822	5.657200274	6	-2.840661628	12.681652590	5.365585827
6	-0.308784737	12.848122563	4.673321589	6	-0.147713602	12.739613404	4.509894007
6	-1.394734460	13.443973265	3.906419288	6	-1.179969739	13.305836727	3.677431029
6	-2.688287251	13.388354684	4.385490304	6	-2.502240309	13.277281979	4.098185893
6	-3.553837546	14.558084248	4.284408868	6	-3.345247326	14.430624590	3.905803722
6	-4.368749832	14.622500002	5.493671220	6	-4.206193737	14.548192963	5.055340413
6	-4.662071302	15.844182325	6.064054836	6	-4.524756578	15.802651491	5.557056269
6	-4.613727920	16.007028785	7.513397035	6	-4.546194360	16.023735201	6.980511104
6	-4.077788320	17.333094084	7.801607938	6	-4.031197760	17.346481921	7.231842813
6	-3.233001570	17.522344055	8.875809205	6	-3.233641312	17.576167578	8.343895978
6	0.711244290	15.498612384	9.701987230	6	0.622747983	15.579168276	9.437923849
6	-0.971301908	17.775653205	9.504230155	6	-1.032891500	17.845672070	9.061688149
6	-1.889339729	12.332073090	7.829125456	6	-1.866087944	12.355957826	7.590376965
6	13.803407494	22.998379119	7.569963760	6	13.828686210	22.767423617	7.361190298
6	13.266519903	23.412838422	8.861269877	6	13.305690215	23.247890678	8.613995385
6	13.904589044	23.047427707	10.027923237	6	13.920099034	22.890353948	9.804557163
6	15.117557122	22.239851820	9.970298315	6	15.081966151	22.038514129	9.788227813
6	15.625079206	21.844198845	8.749197367	6	15.587255684	21.575604770	8.580652184
6	14.950554702	22.234313559	7.515798526	6	14.947843428	21.947418725	7.343519531
6	12.679768303	22.691132905	6.693161140	6	12.714255398	22.488186070	6.492293180
6	11.448371288	22.916114121	7.441859180	6	11.502226522	22.798508963	7.208583848
6	11.812063506	23.361815126	8.780434282	6	11.869014545	23.269515915	8.519862518
6	11.073198671	22.949371026	9.868213545	6	11.099272652	22.925043000	9.620703091
6	13.123158487	22.610099791	11.179268679	6	13.120795452	22.535928573	10.948825847
6	15.084999790	21.301981185	11.087723357	6	14.999579490	21.153231182	10.922655559
6	15.562629470	20.018329090	10.922722095	6	15.421826624	19.835980276	10.805121592

6	16.099165630	19.600026134	9.631725065
6	16.129707527	20.486739495	8.574922756
6	15.766552090	20.038713222	7.234258568
6	15.036750394	21.118704934	6.579609912
6	13.973239886	20.829688392	5.748353138
6	12.761028170	21.638092766	5.805954657
6	10.363812367	22.083280870	7.262809117
6	10.450519430	20.969588597	6.324983777
6	11.615854731	20.753348464	5.617540065
6	12.120675739	19.396987635	5.443146192
6	13.577172446	19.443988714	5.524047977
6	14.268000664	18.422422755	6.142681079
6	15.392946156	18.727859765	7.021086689
6	15.358585149	17.791365651	8.138626412
6	15.700706663	18.215568654	9.405964284
6	13.852628715	21.530993548	11.835175708
6	12.605207135	17.169363716	11.128259057
6	12.524022134	18.222511942	12.015115627
6	11.311721679	19.031479763	12.072120534
6	10.248300165	18.742440352	11.240899682
6	10.334653582	17.626969829	10.304840570
6	12.017682387	16.447460717	8.960037051
6	13.473267767	16.499288713	9.039938060
6	13.835644109	16.944767263	10.378347437
6	14.917630448	17.780151789	10.556076135
6	14.832931618	18.892399678	11.495211948
6	13.668417346	19.107770498	12.203238517
6	11.707591521	20.417723797	12.296757436
6	11.017164296	21.438832034	11.677257594
6	9.892557597	21.133406028	10.798786293
6	9.518772255	19.822562988	10.585908415
6	9.155349150	19.375429064	9.245075442
6	9.660190392	18.018021873	9.071224877
6	10.167032644	17.622034129	7.850002081
6	11.379869309	16.815014540	7.793467041
6	14.213632318	16.910051884	7.951280364
6	13.539144014	17.298296676	6.717427935
6	12.161816907	17.253385517	6.641602222
6	11.431910979	18.330967422	5.985453749
6	10.199160210	18.559996072	6.732495055
6	9.720741943	19.843479850	6.896983254
6	9.183739735	20.262308857	8.188113718
6	9.580914331	21.647739987	8.412855530
6	9.926480962	22.068602879	9.680456318
6	13.163886522	20.465058003	12.377540924
6	11.746544646	22.563226103	11.102125279
6	11.481049225	16.861388918	10.250822819

6	15.943878985	19.355554226	9.550606508
6	16.026884166	20.208660537	8.459561064
6	15.660475062	19.735809326	7.147594395
6	14.994132804	20.811255187	6.458008179
6	13.919861571	20.539782349	5.621473355
6	12.759099114	21.394402601	5.638785862
6	10.378865131	22.000226700	7.045991947
6	10.425292846	20.862166310	6.161728174
6	11.591913013	20.564775540	5.470236731
6	12.031968434	19.197419657	5.350303437
6	13.470240350	19.182294934	5.443110757
6	14.111688326	18.145864365	6.107415375
6	15.226531133	18.428182141	6.975893523
6	15.140737754	17.543529373	8.109222581
6	15.491844863	17.999948823	9.370472034
6	13.787507595	21.461746347	11.640229554
6	12.361088629	17.148619630	11.070095228
6	12.316878566	18.243316828	11.922005374
6	11.156999410	19.097275471	11.941376554
6	10.082486620	18.824747266	11.106043967
6	10.128572281	17.687597024	10.221296543
6	11.768057354	16.380354712	8.950314002
6	13.205576941	16.367383930	9.042806667
6	13.570747367	16.842989176	10.350839780
6	14.690964943	17.642376912	10.510790926
6	14.647544158	18.776019146	11.398329650
6	13.482350811	19.072464143	12.090382540
6	11.605363847	20.455774612	12.120473064
6	10.964412939	21.492732540	11.455620405
6	9.849538933	21.209937100	10.586649538
6	9.417749616	19.901253550	10.415689843
6	9.052232838	19.428867148	9.104085464
6	9.491024294	18.061083247	8.983875198
6	9.993685230	17.595966911	7.776257029
6	11.152919156	16.739136507	7.758908233
6	13.976989914	16.712454055	7.942131111
6	13.339609673	17.084623412	6.704554829
6	11.953792784	17.098352501	6.614594822
6	11.288530784	18.175380544	5.925239028
6	10.077376632	18.483215456	6.643353314
6	9.654474363	19.800827695	6.759108554
6	9.132035554	20.283223325	8.012768279
6	9.579579321	21.642100702	8.190197184
6	9.933065481	22.096295745	9.453203885
6	13.043714036	20.439384008	12.213969975
6	11.735831268	22.553012003	10.858361081
6	11.246082316	16.863824992	10.204455568
6	7.067690436	17.542543882	0.006639507
6	6.826268950	17.083656443	1.299976908
6	7.708849708	16.715453757	-0.912232206
6	7.204639489	15.811886910	1.664024943
6	8.086277138	15.443005313	-0.548469038
6	7.830209792	14.978165514	0.739829823
1	6.361680722	17.737642643	2.022245020
1	7.878978373	17.062674790	-1.920070170
1	7.037894878	15.465424088	2.672726902
1	8.551524548	14.790216845	-1.271417557

$(C_{60})_4$@$(RuP$-$cor)_2$·bpy (sandwich-like)

Gas-phase. GFN2-xTB

Atom	X	Y	Z
6	-1.883181488	-0.147934675	-0.666487103
6	-0.722316891	-0.504907064	-1.361851409
6	-0.771599168	-0.741424423	-2.718931227
7	-1.892625715	-0.662031960	-3.447206653
6	-3.013463875	-0.352487611	-2.783399832
6	-3.052966427	-0.085324540	-1.431603320
1	0.219276235	-0.615515163	-0.847825034
1	0.135072493	-1.011887973	-3.245495927
1	-3.928180617	-0.310339702	-3.360836459
1	-3.993476327	0.193269290	-0.983718280
6	-1.873112304	0.139921344	0.764015529
6	-0.713456876	0.551455082	1.430478819
6	-0.743545894	0.801165888	2.785655715
7	-1.845029396	0.686872598	3.538840573
6	-2.963962518	0.314984820	2.904426903
6	-3.020785571	0.029522799	1.556811949
1	0.211427478	0.695843740	0.894963085
1	0.162882874	1.113414862	3.288640807
1	-3.862383247	0.233732828	3.503096766
1	-3.956690571	-0.299669372	1.134197769
44	-1.775863284	0.955972459	5.590070234
6	1.095851469	-0.067241495	5.551643499
6	1.868170847	-1.279867565	5.558569371
1	2.937131644	-1.332273576	5.488216843
6	0.990356280	-2.305739576	5.658136187
1	1.205085824	-3.355371646	5.707683582
6	-0.325300072	-1.728545313	5.696082429
6	-1.505555841	-2.456985313	5.859912531
6	-2.787511011	-1.905041801	5.914862825
6	-3.996964663	-2.668949906	6.059990744
1	-4.044929819	-3.736651791	6.149870442
6	-5.024822147	-1.788340225	6.039703676
1	-6.074697638	-1.996539193	6.109742931
6	-4.450596039	-0.480058488	5.879496851
6	-5.192455975	0.697718086	5.752119125
6	-4.653474130	1.966865579	5.521310001
6	-5.430599495	3.168838782	5.384231397
1	-6.500927158	3.221144249	5.422728513
6	-4.554882587	4.187044073	5.216111785
1	-4.773160794	5.226629183	5.068123663
6	-3.235955391	3.618117146	5.271128756
6	-2.053036273	4.358465751	5.204836076
6	-0.766163988	3.817765667	5.267632482
6	0.440767606	4.597019913	5.324572483
1	0.485541293	5.668335550	5.318274216
6	1.470195688	3.724246289	5.421983961
1	2.514824916	3.948043285	5.512706108
6	0.901119004	2.403666472	5.417560568
6	1.642195757	1.220143563	5.496860954
6	-1.392178557	-3.921348939	6.035916884
6	-0.964129726	-4.744518017	4.999723271
1	-0.718161262	-4.306607439	4.042451152
6	-0.869993327	-6.110909767	5.179832981
1	-0.562470965	-6.739300341	4.356346396
6	-1.196111507	-6.692050105	6.403092868
6	-1.620869914	-5.862817185	7.441008953

$(C_{60})_4$@$(RuP$-$cor)_2$·bpy (tweezer-like)

Gas-phase. GFN2-xTB

Atom	X	Y	Z
6	2.040699563	-0.109973616	-0.180793936
6	2.547439697	-0.754167540	-1.316476047
6	1.905426513	-0.638218455	-2.530123959
7	0.802629063	0.096955856	-2.722583079
6	0.310945081	0.719024879	-1.643600722
6	0.872677314	0.634591474	-0.387767329
1	3.451757577	-1.338880354	-1.269012552
1	2.303641388	-1.159107200	-3.391604412
1	-0.584244492	1.311534061	-1.785315977
1	0.384763190	1.143932514	0.427393709
6	2.684159519	-0.209087985	1.125258378
6	3.650192166	-1.181306186	1.414115600
6	4.238223980	-1.233918046	2.659255908
7	3.926025807	-0.407735833	3.666062721
6	3.002582403	0.524976111	3.400605717
6	2.382644906	0.664276730	2.177658573
1	3.941708504	-1.913260982	0.678366603
1	4.994094412	-1.983295474	2.857694731
1	2.746175917	1.202466926	4.204964660
1	1.672937251	1.465481248	2.049954344
44	4.768132023	-0.614681180	5.545170074
6	5.778710811	-3.400452785	4.828919142
6	5.401923380	-4.787368674	4.858157244
1	6.023801900	-5.602580514	4.543601057
6	4.140154206	-4.842382470	5.345086039
1	3.540972628	-5.713598196	5.523650023
6	3.731260004	-3.488306791	5.603633047
6	2.488043255	-3.120488915	6.124421249
6	2.102630764	-1.811881204	6.427057718
6	0.806237523	-1.440530541	6.924827239
1	-0.008418152	-2.118379609	7.089970758
6	0.825445599	-0.100233579	7.114584380
1	0.029920740	0.527913045	7.465399262
6	2.133092324	0.357232744	6.730805459
6	2.546907345	1.692398273	6.774815014
6	3.780653938	2.166607765	6.319680246
6	4.196294620	3.541874346	6.375739696
1	3.618387555	4.345719988	6.787814782
6	5.432941634	3.601928555	5.829094835
1	6.049900392	4.467829226	5.688790367
6	5.790676567	2.260893921	5.453391160
6	7.005389183	1.902478995	4.864229023
6	7.370662776	0.601862919	4.508649429
6	8.655163288	0.236389827	3.976191811
1	9.451649132	0.921412951	3.760950552
6	8.656829138	-1.109874666	3.838577679
1	9.455283522	-1.735498173	3.490575904
6	7.372641898	-1.577239250	4.285561531
6	7.003913837	-2.923823895	4.353956102
6	1.524177059	-4.203226313	6.411970346
6	1.013664950	-4.999509483	5.391149861
1	1.305772908	-4.801542687	4.369345635
6	0.132171014	-6.024826916	5.671632324
1	-0.271666370	-6.620477294	4.865568371
6	-0.262924891	-6.284838687	6.981860375
6	0.242476953	-5.479219243	8.001699766

1	-1.854909665	-6.295792126	8.402829879
6	-1.719410655	-4.498718889	7.259541374
1	-2.042102412	-3.864307336	8.072245972
6	-6.663145914	0.596627769	5.872192089
6	-7.487876595	0.883922331	4.789054633
1	-7.044548829	1.152941227	3.840300681
6	-8.862025554	0.822319749	4.914527850
1	-9.491163856	1.024220978	4.059541461
6	-9.446442028	0.473627926	6.128983816
6	-8.619903149	0.157725512	7.203696770
1	-9.064740128	-0.118750021	8.148768178
6	-7.246136256	0.219667826	7.078010823
1	-6.611825776	-0.008338775	7.922102511
6	-2.159463872	5.830788732	5.132176336
6	-1.645662926	6.532954023	4.046138459
1	-1.207058597	5.987383392	3.222519581
6	-1.696589886	7.913039822	4.011322830
1	-1.314574184	8.444971438	3.151829438
6	-2.259572036	8.627247600	5.065424330
6	-2.802446509	7.922572950	6.136511362
1	-3.245791738	8.467993295	6.957151502
6	-2.751375613	6.543386224	6.171250261
1	-3.151456368	6.006431127	7.018904900
6	3.112231633	1.341194446	5.563466907
6	3.836041333	1.957571109	4.546524762
1	3.317555052	2.326645782	3.672536146
6	5.206897751	2.093311953	4.638451846
1	5.749026613	2.574075305	3.838242710
6	5.898271002	1.599554125	5.742297595
6	5.172008566	0.976135700	6.756708156
1	5.690628200	0.611042557	7.631521230
6	3.800756852	0.854508013	6.672077368
1	3.246192325	0.393478740	7.476296609
6	-1.696799970	1.174145889	7.421803523
7	-0.226112676	-0.375508409	5.610216541
7	-3.097856004	-0.588333974	5.794780337
7	-3.331623758	2.272160105	5.431703888
7	-0.453642488	2.497229772	5.333103350
8	-1.641791995	1.307273655	8.560324670
44	-1.852957530	-0.845377589	-5.508518729
6	0.953268892	0.341564335	-5.462646470
6	1.650230255	1.597182882	-5.391010687
1	2.713781803	1.710617846	-5.311215520
6	0.711683603	2.572365533	-5.431348281
1	0.861520670	3.634301503	-5.414419901
6	-0.566040265	1.919815900	-5.511237112
6	-1.785411229	2.581160459	-5.672898645
6	-3.031163796	1.956534451	-5.760079009
6	-4.278002234	2.645251232	-5.955481942
1	-4.383643475	3.705120236	-6.081577472
6	-5.253375389	1.706566994	-5.945549285
1	-6.307647814	1.849217305	-6.080775239
6	-4.611159000	0.439267918	-5.724772303
6	-5.280340674	-0.781043394	-5.594927552
6	-4.660852448	-2.017325275	-5.383170397
6	-5.364070273	-3.247775094	-5.141652313
1	-6.429587185	-3.345023055	-5.065622772
6	-4.428041976	-4.217676556	-5.013403561
1	-4.581542537	-5.259701174	-4.810732379
6	-3.147072134	-3.587807460	-5.182724405

1	-0.041649425	-5.679774774	9.024702578
6	1.122692397	-4.454202911	7.721209365
1	1.522093688	-3.847396418	8.520773963
6	1.601688297	2.676529312	7.344054685
6	1.077469968	3.700845013	6.561625905
1	1.353661333	3.767730371	5.518368305
6	0.199513052	4.621227656	7.100749040
1	-0.223065144	5.393375051	6.474070883
6	-0.174458626	4.542430517	8.439278020
6	0.337313454	3.506931185	9.216998711
1	0.052752092	3.437551814	10.257036267
6	1.214180793	2.587075602	8.677815249
1	1.617626057	1.795470889	9.292117009
6	8.006438548	2.971392167	4.658033521
6	8.417484914	3.337396553	3.380668964
1	7.977404180	2.849437978	2.522368867
6	9.368546608	4.324041183	3.202431483
1	9.656464541	4.619970860	2.203932224
6	9.936444814	4.966228997	4.299174086
6	9.511749380	4.610175260	5.576689459
1	9.947235268	5.102551935	6.434238653
6	8.561345982	3.625158907	5.754511006
1	8.250189760	3.343356676	6.749845786
6	7.995210994	-3.921042545	3.895780682
6	8.390513279	-3.969844329	2.562376783
1	7.951040040	-3.282019212	1.853342242
6	9.327577176	-4.891101173	2.138734941
1	9.607368534	-4.930567742	1.095817601
6	9.899125647	-5.788592138	3.037755756
6	9.496041282	-5.741716559	4.371924845
1	9.944570860	-6.418794615	5.084495878
6	8.557701009	-4.822214467	4.794792502
1	8.263723468	-4.783899917	5.833556858
6	5.515322890	-0.815724083	7.221830616
7	4.743993073	-2.642441830	5.281075172
7	2.870571730	-0.698469518	6.296844660
7	4.766572008	1.420164096	5.752607556
7	6.617549886	-0.516842657	4.677387650
8	5.984985690	-0.945029603	8.260822518
44	-0.044658326	0.305361202	-4.598208974
6	2.547954057	-0.672322870	-5.857447094
6	3.829864776	-0.212252465	-6.319183878
1	4.597319428	-0.836660981	-6.733307409
6	3.863226227	1.126553274	-6.121495958
1	4.655771288	1.808553209	-6.360117854
6	2.606660981	1.494068990	-5.526965318
6	2.222077182	2.804648945	-5.233418527
6	0.986056186	3.179585843	-4.699799364
6	0.580017953	4.538424646	-4.459477221
1	1.186223830	5.404515290	-4.640099704
6	-0.684756948	4.494095997	-3.978648251
1	-1.318711821	5.315427771	-3.707175560
6	-1.047994795	3.107208154	-3.889933802
6	-2.269458655	2.631531254	-3.412866225
6	-2.603194341	1.277019004	-3.280634355
6	-3.798498853	0.800232419	-2.640581243
1	-4.519306001	1.413607590	-2.137008764
6	-3.804738382	-0.546649623	-2.772218846
1	-4.536261898	-1.237490522	-2.401430698
6	-2.609666708	-0.899887079	-3.488754475

6	-1.924246701	-4.264420899	-5.197812836	6	-2.289605491	-2.199267526	-3.896599319
6	-0.675257687	-3.651097059	-5.332189461	6	-1.089982364	-2.570093246	-4.501451031
6	0.565383556	-4.357312674	-5.501618257	6	-0.776526337	-3.897344930	-4.952559290
1	0.666927908	-5.423252928	-5.559154112	1	-1.427828699	-4.745110696	-4.863549560
6	1.539569238	-3.424231893	-5.610982261	6	0.458656972	-3.843933482	-5.504353574
1	2.586316233	-3.585442567	-5.777531275	1	1.012109698	-4.641346975	-5.960435570
6	0.903986086	-2.139395883	-5.496305847	6	0.905994843	-2.480515180	-5.399352108
6	1.575553291	-0.912093611	-5.516940585	6	2.124016405	-2.005280143	-5.895868537
6	-1.752836665	4.053133124	-5.825189507	6	3.169725154	3.887612302	-5.577188473
6	-1.437730135	4.886657672	-4.757834233	6	4.374997248	4.035942801	-4.899137259
1	-1.220839431	4.452039106	-3.792015902	1	4.619345372	3.352895096	-4.097653335
6	-1.416478906	6.258646529	-4.919613773	6	5.247931742	5.054471420	-5.229855048
1	-1.196798379	6.893791523	-4.073492690	1	6.168387412	5.172141389	-4.676227164
6	-1.703962384	6.834511500	-6.154937843	6	4.940708895	5.951426857	-6.250097142
6	-2.014729661	5.994355303	-7.224051044	6	3.733673582	5.796391152	-6.931338236
1	-2.216711854	6.424395427	-8.194378494	1	3.492259216	6.470187781	-7.740740768
6	-2.040936635	4.624629207	-7.061111548	6	2.861594767	4.780052798	-6.600155830
1	-2.276501475	3.981934442	-7.896903620	1	1.933942287	4.662000763	-7.141083989
6	-6.755328591	-0.772869722	-5.684622567	6	-3.358921367	3.565465068	-3.065254452
6	-7.529081510	-0.088165692	-4.752306448	6	-3.288593710	4.488556569	-2.027713025
1	-7.042778202	0.443943728	-3.946504967	1	-2.360790415	4.617084198	-1.487967048
6	-8.907349585	-0.099679002	-4.836177867	6	-4.411742787	5.197660433	-1.643372118
1	-9.493785400	0.413447772	-4.087656648	1	-4.344924105	5.895076888	-0.820317342
6	-9.552247315	-0.792976870	-5.857838156	6	-5.636637303	5.011929273	-2.284462947
6	-8.774105929	-1.473349055	-6.794212006	6	-5.677807293	4.147071611	-3.376086818
1	-9.259150853	-1.995646471	-7.606171523	1	-6.608636953	3.973679806	-3.890098906
6	-7.397222711	-1.465350012	-6.707715339	6	-4.562080456	3.435192742	-3.755846637
1	-6.803417574	-1.989029109	-7.442602306	1	-4.623253875	2.740161289	-4.580651403
6	-1.944239018	-5.739072789	-5.123210486	6	-3.383517525	-3.179373114	-3.731104426
6	-1.281042339	-6.408716435	-4.098597917	6	-3.323087071	-4.250432099	-2.847461501
1	-0.788312702	-5.836877974	-3.324990521	1	-2.400412561	-4.459310001	-2.324658192
6	-1.254594060	-7.788996970	-4.059472668	6	-4.450854278	-5.008494383	-2.591513283
1	-0.756042944	-8.295698474	-3.245664735	1	-4.397684135	-5.826223519	-1.886737560
6	-1.888076378	-8.537369490	-5.047970477	6	-5.662698754	-4.727240380	-3.221570766
6	-2.577778487	-7.867531637	-6.054973086	6	-5.688225206	-3.719839301	-4.185523218
1	-3.075959179	-8.439739463	-6.824341771	1	-6.604879795	-3.502993577	-4.712420242
6	-2.604131514	-6.487656451	-6.094004141	6	-4.569993244	-2.952332790	-4.426419518
1	-3.118879687	-5.977405226	-6.894967884	1	-4.613242371	-2.143441443	-5.141221563
6	3.045082745	-0.937231647	-5.647705575	6	3.028318939	-2.982369420	-6.538602990
6	3.847498549	-1.621679555	-4.738361040	6	3.592955297	-4.027726266	-5.814234480
6	5.217959672	-1.667214199	-4.900636418	6	4.443044680	-4.928867495	-6.424751564
6	5.829353603	-1.005239390	-5.962134229	6	4.749206527	-4.813084572	-7.778654201
6	5.027258479	-0.306866712	-6.863497183	6	4.176279020	-3.768057932	-8.503362973
1	5.487106100	0.195641062	-7.702452677	1	4.384137727	-3.680208687	-9.559952665
6	3.656306809	-0.282980709	-6.715527583	6	3.330407709	-2.865649852	-7.892539495
1	3.042081781	0.232641665	-7.439154716	1	2.884334043	-2.065161840	-8.464507604
6	-1.801963893	-0.967646638	-7.350151336	6	-0.776226294	0.503062881	-6.281743529
7	-0.384908714	0.572624289	-5.497295408	7	1.842539617	0.381989772	-5.367968193
7	-3.267781169	0.625796548	-5.625833091	7	-0.014267148	2.338679272	-4.329398045
7	-3.322686484	-2.252791159	-5.365375754	7	-1.881568556	0.219108486	-3.732399323
7	-0.437755000	-2.313653951	-5.349832230	7	-0.051285213	-1.737754584	-4.783948072
8	-1.766449750	-1.034307096	-8.495239573	8	-1.220014973	0.625108617	-7.333248357
6	-2.276524759	10.101086144	5.068840699	6	10.962727467	6.010896325	4.132526645
6	-1.036851910	10.827110980	4.929220986	6	12.162404041	5.730451433	3.381132523
6	-3.493568930	10.754705774	5.129251573	6	10.706303518	7.272409377	4.638793165
6	-1.168677371	12.193966289	5.072985215	6	13.089228888	6.753966489	3.402704434
6	0.257359005	10.417435712	4.467765195	6	12.480687223	4.683590665	2.454384936
6	-3.618864344	12.175443498	5.037308614	6	11.583107356	8.383280484	4.442514444
1	-4.390688412	10.149228574	5.139244556	1	9.746283912	7.440312027	5.109615922
6	-0.217220911	13.110204416	4.573130206	6	14.106435092	6.884479590	2.431582689

6	-2.416870503	12.850201112	5.124073574
6	1.181291369	11.309593628	3.977585663
1	0.492281953	9.362609239	4.435157822
6	-4.719493225	12.997045682	4.627435434
6	-0.880240844	14.326808312	4.306942408
6	0.923875742	12.716737847	3.902508641
6	-2.240281938	14.165870164	4.647312390
1	2.102977905	10.924563204	3.562108497
6	-4.546376063	14.277530268	4.158058918
1	-5.710290309	12.563620410	4.600948525
6	-0.449503793	15.219428200	3.344583838
6	1.494467990	13.735904124	3.073363564
6	-3.254044257	14.887015456	4.046962388
1	-5.406879654	14.810648300	3.776392473
6	0.844426657	14.918238501	2.808256875
6	-1.467142864	16.118482883	2.887380516
1	2.427978462	13.531631652	2.566163047
6	-2.792414896	15.961238922	3.219098450
1	1.286788674	15.606021814	2.100006574
1	-1.197188912	16.894907089	2.184094427
1	-3.521966169	16.618754354	2.765493533
6	-10.911895759	0.438214855	6.283308835
6	-11.688938044	1.613428094	5.971299050
6	-11.515600744	-0.754569185	6.636771720
6	-13.034669655	1.487816761	6.253828371
6	-11.361584235	2.798906313	5.234037135
6	-12.933747616	-0.920746717	6.694788597
1	-10.880930952	-1.621840954	6.765499907
6	-14.019732046	2.304591791	5.655864429
6	-13.641026348	0.262064449	6.602032821
6	-12.320791025	3.591495826	4.649994789
1	-10.321230230	3.042201375	5.068290354
6	-13.745720891	-2.096002819	6.577009088
6	-15.229421183	1.579016756	5.626020420
6	-13.719643566	3.297610891	4.744449224
6	-14.995197278	0.316178115	6.210825311
1	-12.001994542	4.420390073	4.032156009
6	-15.063973916	-2.043036939	6.190894379
1	-13.281063578	-3.064129951	6.707568228
6	-16.208062007	1.793873303	4.674828548
6	-14.818878594	3.673403997	3.906669562
6	-15.724754901	-0.809627432	5.881255307
1	-15.594720175	-2.971739307	6.029228083
6	-15.994694036	2.961360025	3.872460899
6	-17.103627825	0.689113135	4.500705950
1	-14.686781665	4.491387138	3.211066068
6	-16.875106863	-0.541254661	5.070697582
1	-16.749811979	3.241114869	3.150079449
1	-17.940606313	0.800024982	3.824345986
1	-17.539529117	-1.358531880	4.823885608
6	-1.097891447	-8.140924712	6.625443623
6	-2.178040639	-8.819433135	7.305785116
6	-0.014188830	-8.843387931	6.129380699
6	-1.912541830	-10.137392466	7.619307819
6	-3.552534622	-8.448752939	7.463358225
6	0.125466867	-10.259250874	6.264897700
1	0.729339073	-8.299488971	5.562190002
6	-2.927641909	-11.081847912	7.886912754
6	-0.791331658	-10.837178577	7.121403358
6	-4.541293845	-9.369067318	7.722302645

6	12.812145330	8.039463971	3.914514085
6	13.468068997	4.813344398	1.506754427
1	11.877824158	3.786144152	2.450527763
6	11.329842596	9.793959607	4.459177707
6	14.448491033	8.250087100	2.337684419
6	14.260989245	5.999938123	1.382627966
6	13.648008312	8.964221851	3.254520516
1	13.596156880	4.021711638	0.780566605
6	12.142187883	10.694265794	3.811719817
1	10.419425948	10.153268313	4.919887886
6	14.954217263	8.817940326	1.184315617
6	14.990758010	6.533463092	0.271578527
6	13.303380570	10.289892353	3.075467044
1	11.844612465	11.733959915	3.782983376
6	15.316846556	7.865964743	0.176999206
6	14.786907821	10.239184473	1.113276746
1	15.214587286	5.883738736	-0.563990762
6	14.006612154	10.934698006	2.006830103
1	15.785669552	8.222233326	-0.730528663
1	15.215749888	10.771914618	0.275065276
1	13.846502169	11.991869264	1.842235340
6	-1.102012679	5.521836532	9.033283716
6	-0.806317958	6.932151052	8.956245563
6	-2.287417872	5.061048564	9.577062553
6	-1.697061912	7.730012228	9.646924004
6	0.108325758	7.667580426	8.132427184
6	-3.304746317	5.931197998	10.076507978
1	-2.489621262	3.999356015	9.517570859
6	-1.860755486	9.106098505	9.374027826
6	-2.907927939	7.249315553	10.189286233
6	-0.054418472	9.005940069	7.863970274
1	0.915857648	7.139113333	7.645003078
6	-4.714652311	5.730864192	10.239029953
6	-3.174127909	9.470767248	9.738515061
6	-1.143589607	9.768356424	8.397384116
6	-3.821707509	8.322518765	10.242109765
1	0.619624754	9.479114490	7.162399777
6	-5.605201000	6.777061901	10.285286395
1	-5.100185984	4.720110775	10.233530743
6	-3.855536752	10.512743181	9.139754708
6	-1.758205168	10.979065815	7.941140192
6	-5.190110132	8.144554339	10.178017404
1	-6.663846545	6.556123520	10.314091629
6	-3.040898540	11.330116710	8.290785063
6	-5.279608293	10.431755778	9.275666475
1	-1.228734226	11.593517860	7.225261801
6	-5.910282267	9.312448528	9.765943905
1	-3.480068560	12.208908138	7.837876015
1	-5.885135235	11.243584588	8.895494956
1	-6.991546965	9.279848034	9.755020628
6	-1.179814435	-7.385360674	7.308908320
6	-2.279338923	-7.138280408	8.213277530
6	-1.018981594	-8.616936582	6.699666024
6	-2.975851544	-8.271237785	8.583820140
6	-2.930113166	-5.911961347	8.565158704
6	-1.898850956	-9.719786419	6.926968819
1	-0.225137402	-8.725743362	5.972655331
6	-4.294386090	-8.229719067	9.087899041
6	-2.788214287	-9.527612526	7.966484607
6	-4.215019028	-5.871828544	9.053842781

1	-3.840858433	-7.423281515	7.277018511
6	0.885475466	-11.202164297	5.499215793
6	-2.432686552	-12.362370069	7.558218539
6	-4.272696702	-10.769031660	7.845904692
6	-1.112349628	-12.210152763	7.082925821
1	-5.569424384	-9.032924934	7.737193164
6	0.573550878	-12.540360424	5.462810515
1	1.674235852	-10.834705732	4.856460701
6	-3.251472985	-13.405187923	7.169916211
6	-5.132226079	-11.901923949	7.669340469
6	-0.533342932	-13.089546923	6.187789696
1	1.126801458	-13.184335269	4.792316481
6	-4.649537051	-13.148328461	7.349815838
6	-2.579550173	-14.432019332	6.431077878
1	-6.203041113	-11.751029585	7.698528828
6	-1.294429994	-14.282278575	5.965801957
1	-5.355317152	-13.940176597	7.137478175
1	-3.133528372	-15.317666252	6.150043483
1	-0.878122548	-15.054678917	5.333030853
6	7.357036461	1.718239586	5.864514256
6	8.114965393	0.558930240	6.280430111
6	7.989603832	2.897245967	5.511649341
6	9.452395272	0.809519480	6.513550152
6	7.786019819	-0.833921960	6.224226195
6	9.408984216	3.061413684	5.546191386
1	7.381262196	3.708060344	5.133315655
6	10.436690885	-0.203139504	6.510032711
6	10.081170029	2.024920556	6.163902106
6	8.745520303	-1.820091389	6.213564861
1	6.753346822	-1.120757725	6.080285750
6	10.264999326	3.982222844	4.858480042
6	11.671355600	0.386860849	6.163324244
6	10.146423934	-1.529960437	6.257677077
6	11.450546868	1.764326691	5.947770242
1	8.430873627	-2.845510954	6.072107845
6	11.600380760	3.729097257	4.648704126
1	9.826544754	4.862794424	4.408007325
6	12.689703240	-0.312376505	5.544464681
6	11.273242327	-2.303322295	5.825629783
6	12.234547834	2.527218776	5.103551022
1	12.170024193	4.419287796	4.040663062
6	12.476427346	-1.728405610	5.491306180
6	13.629672850	0.517515241	4.851066269
1	11.144643788	-3.366310798	5.670841204
6	13.414000841	1.859234101	4.641490382
1	13.255695879	-2.356631081	5.081027121
1	14.493671128	0.053454840	4.394328093
1	14.115437456	2.407506711	4.026966368
6	7.286223549	-1.021978325	-6.152457060
6	7.969029950	0.231399157	-6.378158036
6	7.986753683	-2.209245692	-6.034673846
6	9.308363808	0.100154203	-6.683271583
6	7.569958865	1.572698909	-6.070592281
6	9.410442100	-2.288275669	-6.140088600
1	7.431536680	-3.103911909	-5.785286897
6	10.239183414	1.148700930	-6.514623323
6	10.008832558	-1.121664206	-6.576332270
6	8.476890881	2.594204149	-5.906965913
1	6.526702852	1.771815607	-5.865960497
6	10.332749890	-3.267952093	-5.648540087

1	-2.431592214	-4.976833709	8.349175539
6	-2.175857254	-10.875024805	6.125845265
6	-4.918109851	-9.459558215	8.785395396
6	-5.001380654	-7.052624074	9.241130251
6	-3.986693862	-10.261250106	8.090844436
1	-4.678938668	-4.907181282	9.210672397
6	-3.342463425	-11.591286849	6.248666247
1	-1.485078269	-11.137767130	5.335824908
6	-6.283762869	-9.585574369	8.618046266
6	-6.422780908	-7.225846542	9.295191528
6	-4.365646157	-11.234668480	7.185959807
1	-3.533814977	-12.395953774	5.551387907
6	-7.029213272	-8.423352960	9.000578974
6	-6.676862189	-10.737676850	7.863169629
1	-7.044557296	-6.361078311	9.484710010
6	-5.769604729	-11.517343854	7.185424809
1	-8.109701138	-8.464509721	8.967145027
1	-7.731582297	-10.947495100	7.745952105
1	-6.139128144	-12.316080818	6.556353183
6	10.914914186	-6.761709940	2.613783707
6	10.803332929	-8.133274926	3.054782564
6	11.919351343	-6.369655745	1.746835266
6	11.891195938	-8.922022083	2.737539745
6	9.662665969	-8.869414985	3.511271497
6	12.910495039	-7.268071711	1.244624890
1	11.913263688	-5.349022589	1.388048329
6	11.825929333	-10.331626446	2.682417996
6	12.919142324	-8.502466547	1.864614246
6	9.598942974	-10.242122994	3.456102122
1	8.781014338	-8.325372618	3.821411699
6	13.724937984	-7.198377480	0.067841052
6	12.813734979	-10.781191624	1.779602980
6	10.665288577	-11.038707795	2.930756117
6	13.488320081	-9.649542902	1.272949631
1	8.672517655	-10.728388202	3.730900693
6	14.280896364	-8.315890290	-0.507650319
1	13.830020415	-6.246980817	-0.436274362
6	12.700289960	-11.962503102	1.072219471
6	10.655482923	-12.360924886	2.378456468
6	14.086684610	-9.630648099	0.027371164
1	14.806533947	-8.209259487	-1.447143140
6	11.617520282	-12.797806214	1.499715312
6	13.510346026	-12.017568228	-0.107809810
1	9.812667610	-13.005538448	2.589121931
6	14.165372208	-10.914492961	-0.602359287
1	11.502443810	-13.772924835	1.045690757
1	13.532955653	-12.934946203	-0.680644711
1	14.682435003	-10.998632143	-1.548859474
6	5.649403175	-5.757853290	-8.453179507
6	6.669654794	-5.240095686	-9.336717655
6	5.556620941	-7.111972890	-8.185590447
6	7.348779709	-6.208390566	-10.048610812
6	7.273564042	-3.942674253	-9.395419287
6	6.432079081	-8.082490315	-8.763701246
1	4.824515858	-7.439107090	-7.459327482
6	8.619753921	-5.988012055	-10.623139080
6	7.230197967	-7.589384962	-9.777676823
6	8.512302364	-3.728655394	-9.953398066
1	6.783900987	-3.116375091	-8.898496979
6	6.791141191	-9.398273669	-8.324570385

6	11.512156329	0.574912451	-6.309601007
6	9.889319464	2.391279491	-6.021907445
6	11.369921952	-0.828809578	-6.350297912
1	8.114380123	3.560488685	-5.582682920
6	11.659884096	-2.983063171	-5.429185534
1	9.953384307	-4.235941109	-5.349848106
6	12.512303976	1.205255161	-5.595177853
6	10.987613085	3.133756447	-5.478491095
6	12.218932872	-1.686891655	-5.677710983
1	12.282548444	-3.737492654	-4.966813208
6	12.226111161	2.572116820	-5.273972555
6	13.519614036	0.317877832	-5.095218068
1	10.810421788	4.141982513	-5.128523425
6	13.381335259	-1.049728909	-5.134772480
1	12.984381941	3.157544552	-4.771090123
1	14.375867276	0.737807496	-4.584407503
1	14.133542197	-1.661183166	-4.654323459
6	-1.680757961	8.289229282	-6.358994411
6	-2.754121514	8.908550817	-7.103732542
6	-0.676198564	9.050623267	-5.788790579
6	-2.548972724	10.243046408	-7.391497542
6	-4.090604165	8.457868546	-7.353146479
6	-0.614543064	10.473743347	-5.905687421
1	0.061527439	8.546690948	-5.178720121
6	-3.599583242	11.127469341	-7.720074280
6	-1.507074051	11.003986359	-6.816892014
6	-5.114111283	9.319683968	-7.671676258
1	-4.328292593	7.415361849	-7.191620945
6	0.035107735	11.452973146	-5.085991862
6	-3.205557333	12.432133093	-7.352186552
6	-4.923066654	10.734284294	-7.769914310
6	-1.912456103	12.354743037	-6.791627504
1	-6.116817502	8.922617936	-7.756209677
6	-0.359373795	12.769506344	-5.061952927
1	0.800887012	11.127310058	-4.394727093
6	-4.109561411	13.420280058	-7.013138455
6	-5.859137815	11.812067006	-7.644915235
6	-1.447677079	13.258391508	-5.855007058
1	0.108353275	13.438630867	-4.352116682
6	-5.474656147	13.082029961	-7.287191415
6	-3.550946561	14.478131309	-6.225317359
1	-6.914615176	11.597736991	-7.746348988
6	-2.292551950	14.401019600	-5.676741001
1	-6.239386065	13.828262488	-7.118167439
1	-4.174671909	15.326230456	-5.976450590
1	-1.966250675	15.190652070	-5.013179719
6	-11.016884525	-0.821920749	-5.967118020
6	-11.673898826	-2.083295157	-6.224632603
6	-11.747503148	0.332645581	-5.750247954
6	-13.028927553	-1.978920766	-6.467708056
6	-11.223087965	-3.424406522	-6.001680174
6	-13.175737726	0.368069236	-5.779377574
1	-11.210257534	1.233815386	-5.486193799
6	-13.919933450	-3.064676062	-6.320537513
6	-13.759692758	-0.788486801	-6.258687805
6	-12.091081002	-4.481375105	-5.857624368
1	-10.167824392	-3.600633659	-5.844197554
6	-14.099902928	1.286824659	-5.183699679
6	-15.198684981	-2.545175877	-6.024113056
6	-13.511715537	-4.319454804	-5.911060687

6	9.282862228	-7.231136198	-10.710152224
6	9.295623337	-4.788293676	-10.511060926
6	8.423866262	-8.220674370	-10.185438585
1	8.948343359	-2.741291082	-9.880981747
6	7.953486427	-10.014214291	-8.723339838
1	6.172440910	-9.886078253	-7.583227985
6	10.659214681	-7.349172358	-10.690974841
6	10.709303953	-4.890863573	-10.721781953
6	8.890078827	-9.385818953	-9.606795435
1	8.213021408	-10.967807569	-8.283335306
6	11.354078914	-6.101506287	-10.806983194
6	11.129157945	-8.646286339	-10.305696761
1	11.301359101	-3.985465113	-10.722577306
6	10.292597508	-9.609080951	-9.792182630
1	12.433831223	-6.111667549	-10.872433953
1	12.192454959	-8.843480008	-10.333269013
1	10.724268027	-10.533210698	-9.431727725
6	5.849295118	7.042888271	-6.626772387
6	5.303291091	8.365656236	-6.830640568
6	7.209786013	6.810313282	-6.718123726
6	6.201050878	9.276838603	-7.349508386
6	4.093460664	8.954177538	-6.339391040
6	8.155921596	7.833929411	-7.034214527
1	7.574506614	5.819914139	-6.480161713
6	6.028906873	10.673705141	-7.234745187
6	7.585445767	9.018912849	-7.456980052
6	3.927110621	10.314632977	-6.225178185
1	3.318032899	8.306785796	-5.953294852
6	9.561079812	7.923787941	-6.768783452
6	7.304389675	11.277180993	-7.275577562
6	4.953645162	11.245375853	-6.582234184
6	8.266585259	10.253333614	-7.411362856
1	3.024798852	10.687994515	-5.759680010
6	10.225442798	9.126375519	-6.726774716
1	10.098691088	7.023531496	-6.502615762
6	7.580648246	12.486735719	-6.667803732
6	5.178382506	12.594199976	-6.153835567
6	9.561479870	10.377014508	-6.944821143
1	11.265420028	9.134949778	-6.428995036
6	6.420298537	13.181238524	-6.194360490
6	8.970599496	12.700191004	-6.395498645
1	4.361354000	13.137324221	-5.698050440
6	9.907272331	11.702167475	-6.525858286
1	6.542555944	14.168500175	-5.769376124
1	9.279799350	13.652054401	-5.984912664
1	10.923869982	11.900268682	-6.213526760
6	-6.872766781	5.608247473	-1.751568227
6	-7.908642359	6.120445541	-2.619868187
6	-7.083504212	5.481259221	-0.384670055
6	-9.011445629	6.612674576	-1.947931617
6	-8.112637543	6.028650751	-4.040251362
6	-8.308483926	5.821764143	0.260421883
1	-6.320577201	4.979190135	0.195831037
6	-10.285701270	6.735380726	-2.547859864
6	-9.206594557	6.478553015	-0.557527391
6	-9.355215045	6.126653319	-4.618798203
1	-7.269714278	5.824366107	-4.684593985
6	-8.870818363	5.376309614	1.502409353
6	-11.259498006	6.673501913	-1.530165758
6	-10.542523160	6.367617354	-3.853226893

6	-15.098593750	-1.137744315	-5.984209387
1	-11.686932991	-5.450961178	-5.599142978
6	-15.404796344	0.946947341	-4.917596020
1	-13.734262616	2.248246056	-4.848580194
6	-16.143645790	-3.248039683	-5.301686097
6	-14.561980502	-5.129368521	-5.368578863
6	-15.935366917	-0.349767640	-5.216866392
1	-16.026024505	1.651663267	-4.381176980
6	-15.806468335	-4.622869693	-5.080303736
6	-17.146450965	-2.425296592	-4.694268933
1	-14.339441360	-6.151065324	-5.091336023
6	-17.047469948	-1.054555535	-4.653472542
1	-16.525588466	-5.261424175	-4.584982189
1	-17.962433816	-2.902880118	-4.168620238
1	-17.788630840	-0.496824940	-4.096796597
6	-1.829503336	-10.010117929	-5.045086461
6	-0.546703070	-10.671525460	-5.032054413
6	-3.011287898	-10.724322533	-4.970952962
6	-0.622925232	-12.045286003	-5.148231044
6	0.766357138	-10.191016114	-4.714434820
6	-3.053558577	-12.147996132	-4.853661354
1	-3.934037022	-10.164408552	-4.889850727
6	0.421373789	-12.905366253	-4.742614566
6	-1.834455377	-12.763455202	-5.060328353
6	1.781176224	-11.028574050	-4.316411506
1	0.949196873	-9.125428821	-4.717077531
6	-4.061641765	-13.016664399	-4.321103191
6	-0.147101221	-14.149043915	-4.395138264
6	1.605122128	-12.445450368	-4.200489938
6	-1.541837821	-14.061126936	-4.591268286
1	2.720654228	-10.591988003	-4.004792783
6	-3.774969516	-14.279334762	-3.859258985
1	-5.064912616	-12.632465841	-4.194204370
6	0.427355609	-15.003850384	-3.474343391
6	2.311031878	-13.421940087	-3.425959128
6	-2.448690989	-14.822009206	-3.879283052
1	-4.562135491	-14.848345829	-3.382858699
6	1.753522479	-14.630666121	-3.081189038
6	-0.489379416	-15.944642865	-2.902197479
1	3.281064047	-13.163678306	-3.022559903
6	-1.848472753	-15.858534614	-3.093346885
1	2.302289870	-15.284423866	-2.416507487
1	-0.107731492	-16.695125214	-2.222899524
1	-2.491861269	-16.543720724	-2.557714732
1	3.391340352	-2.115586611	-3.891782748
1	5.823424172	-2.204452934	-4.186500079
6	11.206467566	-0.282841535	2.927230209
6	10.441937677	-1.501189070	3.008308630
6	10.710634305	-2.547727570	2.137505662
6	11.755816523	-2.416391597	1.153623336
6	12.493428752	-1.242751073	1.077475260
6	12.213851712	-0.156326897	1.982149276
6	10.317243409	0.819355827	3.184461682
6	9.002595140	0.283104007	3.421052067
6	9.078076453	-1.150682699	3.313945534
6	8.032541803	-1.859069527	2.738688059
6	9.625212920	-3.283417828	1.538553218
6	11.316098804	-3.070261527	-0.053393984
6	11.630345951	-2.527103415	-1.291941803
6	12.396699914	-1.308545797	-1.371324740
6	-10.592099130	6.516838459	-0.297719883
1	-9.444766653	5.954049988	-5.682957246
6	-10.221857622	5.414551666	1.754516803
1	-8.219813398	4.909636136	2.229716385
6	-12.546772994	6.221525804	-1.747781964
6	-11.922096715	6.101220531	-4.125952676
6	-11.169770712	5.903891393	0.796473144
1	-10.589951729	4.976905148	2.673117706
6	-12.868954095	6.028299377	-3.130279223
6	-13.221792551	5.797602968	-0.557033139
1	-12.215045535	5.858654676	-5.138618540
6	-12.571146369	5.648455419	0.645635932
1	-13.875577284	5.731560060	-3.393325296
1	-14.258671449	5.496445020	-0.625849995
1	-13.117754188	5.232940151	1.481802662
6	-6.902681700	-5.405839292	-2.820904741
6	-7.175601162	-5.564607813	-1.408914846
6	-7.856093471	-5.728137870	-3.772136301
6	-8.342549182	-6.249497584	-1.128548802
6	-6.605700020	-4.904058607	-0.272103471
6	-9.153336571	-6.224875496	-3.441053909
1	-7.631500592	-5.520224129	-4.809692545
6	-9.012077467	-6.150496988	0.111646345
6	-9.303079444	-6.571707790	-2.112537492
6	-7.255475176	-4.813611632	0.937525869
1	-5.674264981	-4.367648035	-0.390861958
6	-10.381825788	-6.188635321	-4.178257085
6	-10.382787832	-6.405953071	-0.108183409
6	-8.559826714	-5.360295123	1.150315979
6	-10.562649887	-6.661996541	-1.484274449
1	-6.804258965	-4.220966451	1.722311605
6	-11.608958194	-6.272922144	-3.565221646
1	-10.344519850	-5.993668430	-5.241698034
6	-11.380609744	-5.884469001	0.693061326
6	-9.567827880	-5.019124496	2.110112272
6	-11.749354210	-6.406720907	-2.145373998
1	-12.499326962	-6.140436270	-4.165295946
6	-10.901894838	-5.267955280	1.894568854
6	-12.665841579	-5.827318916	0.062788997
1	-9.277893782	-4.464537743	2.992629108
6	-12.839882156	-6.073222292	-1.279014002
1	-11.622294519	-4.899130909	2.612544903
1	-13.513966657	-5.487500867	0.642249198
1	-13.819086025	-5.917999259	-1.712136585
1	3.371774553	-4.123527986	-4.760272682
1	4.894586864	-5.718847106	-5.842051740
6	-9.170008300	-2.049976041	0.581386935
6	-10.585060014	-2.279909905	0.719634491
6	-11.275167473	-2.970622743	-0.266221665
6	-10.575779833	-3.457601282	-1.426797333
6	-9.213074332	-3.239051607	-1.558647665
6	-8.495453360	-2.521849215	-0.535513088
6	-8.878695166	-0.737274615	1.099531367
6	-10.114771726	-0.154890710	1.558004186
6	-11.169597671	-1.109242828	1.323492408
6	-12.423366743	-0.671646052	0.919017895
6	-12.576402158	-2.517340095	-0.685745980
6	-11.444844454	-3.305989354	-2.564857037
6	-10.918574505	-2.938143960	-3.794826363
6	-9.503088865	-2.709041962	-3.931929309

6	12.820109448	-0.678974099	-0.208568333
6	12.743358638	0.756180530	-0.097648549
6	12.369412889	1.079019928	1.256597525
6	11.511583230	2.141624248	1.504538233
6	10.466503144	2.008631422	2.486956854
6	7.883997516	0.954931567	2.953090043
6	8.038213525	2.190305380	2.227253594
6	9.305037929	2.707074481	1.998185992
6	9.631334442	3.272174398	0.713721464
6	10.995691505	2.922114652	0.407798805
6	11.355222466	2.611399235	-0.896677090
6	12.245639918	1.507365098	-1.154124997
6	11.805100917	0.852868093	-2.359494989
6	11.879686333	-0.528211501	-2.466741225
6	9.999483054	-3.606201132	0.184771762
6	8.361104253	-1.125397551	-3.295629122
6	8.212100111	-2.315163694	-2.597136849
6	7.166619966	-2.447042602	-1.613506941
6	6.306770438	-1.385002384	-1.366306552
6	6.461527266	-0.149621163	-2.093454831
6	8.236980437	1.193867795	-3.119016215
6	9.600707250	0.842510507	-3.423801143
6	9.677045831	-0.590281875	-3.533958384
6	10.794651777	-1.263255633	-3.064510429
6	10.640545307	-2.498110852	-2.338461251
6	9.373491240	-3.015454111	-2.109797412
6	7.682603239	-3.228192890	-0.517633247
6	7.321790611	-2.917354703	0.786256319
6	6.430611143	-1.814138674	1.044327597
6	5.932695040	-1.062827104	-0.012147793
6	5.855795002	0.372639739	0.098217414
6	6.182244447	0.936478098	-1.188074892
6	6.922243267	2.108748053	-1.264620381
6	7.967070936	2.240696284	-2.248822330
6	10.645144916	1.551911239	-2.849575564
6	10.365940945	2.638594183	-1.944816350
6	9.052486500	2.976521482	-1.649988950
6	8.678394169	3.298660249	-0.295740231
6	7.362146726	2.762365566	-0.057652803
6	7.047548206	2.218925403	1.180781771
6	6.279601948	1.001320832	1.261441084
6	6.795913840	0.221316742	2.358513365
6	6.869533508	-1.160655573	2.252020148
6	9.046379265	-3.579166525	-0.824377190
6	8.311402338	-2.945449781	1.833658965
6	7.470120581	-0.022883212	-3.038373306

6	-8.665951624	-2.856894615	-2.834754897
6	-7.609865180	-1.903600851	-2.601395668
6	-7.505108722	-1.695878672	-1.179528680
6	-7.225143795	-0.429883488	-0.681925849
6	-7.924408666	0.058547642	0.480404569
6	-10.351331949	1.201141337	1.378346922
6	-9.361848811	2.027432561	0.733695260
6	-8.170620552	1.466842821	0.294274410
6	-7.624234988	1.849787758	-0.983572757
6	-7.040948836	0.677306661	-1.586083389
6	-7.142415399	0.475967418	-2.955511028
6	-7.431115984	-0.837052572	-3.472146777
6	-8.300258924	-0.684715672	-4.612067597
6	-9.316832232	-1.602672037	-4.837298664
6	-12.681630112	-2.724777577	-2.108300899
6	-12.092224554	0.791337479	-4.981686264
6	-13.045433650	-0.004675281	-4.361378343
6	-13.744385248	0.483438343	-3.199181276
6	-13.463768374	1.748778405	-2.701432922
6	-12.473897349	2.575776610	-3.345322040
6	-10.385438481	2.335261846	-4.602176216
6	-9.801323108	1.162506106	-5.204037019
6	-10.855838954	0.208604847	-5.438819735
6	-10.618178573	-1.147345178	-5.258887299
6	-11.607913069	-1.973582955	-4.614645220
6	-12.799179958	-1.412579928	-4.174806849
6	-13.930723903	-0.623427950	-2.294557003
6	-13.829610302	-0.424117044	-0.924624528
6	-13.538163816	0.889586115	-0.407481299
6	-13.358120253	1.955632204	-1.278778518
6	-12.302970981	2.908486221	-1.045131417
6	-11.757361191	3.294464403	-2.321630240
6	-10.394303258	3.516477542	-2.453178941
6	-9.696649732	3.026352516	-3.614780683
6	-8.548687533	0.722958253	-4.798243796
6	-7.834320473	1.438563060	-3.772726333
6	-8.398350923	2.567024018	-3.192227358
6	-8.291239627	2.777789495	-1.770709098
6	-9.525985835	3.362406528	-1.314304304
6	-10.051510174	2.992058126	-0.085138269
6	-11.466118967	2.759515417	0.051020624
6	-11.652829634	1.655119324	0.956618055
6	-12.669281761	0.736452273	0.732026235
6	-13.346147088	-1.795451309	-2.897215275
6	-13.140280205	-1.389299833	-0.105100423
6	-11.801848064	2.105722218	-4.464993894

References

(1) Williams, D. B. G.; Lawton, M. Drying of Organic Solvents: Quantitative Evaluation of the Efficiency of Several Desiccants. *J. Org. Chem.* **2010**, *75*, 8351–8354.

(2) Ferrero, S.; Barbero, H.; Miguel, D.; García-Rodríguez, R.; Álvarez, C. M. Porphyrin-Based Systems Containing Polyaromatic Fragments: Decoupling the Synergistic Effects in Aromatic-Porphyrin-Fullerene Systems. *RSC Adv.* **2020**, *10*, 36164–36173.

(3) Pan, M.; Lu, Y.; Lu, S.; Yu, B.; Wei, J.; Liu, Y.; Jin, Z. The Dual Role of Bridging Phenylene in an Extended Bipyridine System for High-Voltage and Stable Two-Electron Storage in Redox Flow Batteries. *ACS Appl. Mater. Interfaces* **2021**, *13*, 44174–44183.

(4) Ferrero, S.; Barbero, H.; Miguel, D.; García-Rodríguez, R.; Álvarez, C. M. Dual-Tweezer Behavior of an Octapodal Pyrene Porphyrin-Based System as a Host for Fullerenes. *J. Org. Chem.* **2019**, *84*, 6183–6190.

(5) Ferrero, S.; Barbero, H.; Miguel, D.; García-Rodríguez, R.; Álvarez, C. M. Octapodal Corannulene Porphyrin-Based Assemblies: Allosteric Behavior in Fullerene Hosting. *J. Org. Chem.* **2020**, *85*, 4918–4926.

(6) Rillema, D. P.; Nagle, J. K.; Barringer, L. F.; Meyer, T. J. Redox Properties of Metalloporphyrin Excited States, Lifetimes, and Related Properties of a Series of Para-Substituted Tetraphenylporphine Carbonyl Complexes of Ruthenium(II). *J. Am. Chem. Soc.* **1981**, *103*, 56–62.

(7) Thordarson, P. Determining Association Constants from Titration Experiments in Supramolecular Chemistry. *Chem. Soc. Rev.* **2011**, *40*, 1305–1323.

(8) Thordarson, P. Binding Constants and Their Measurement. In *Supramolecular Chemistry*; Wiley, 2012.

(9) Brynn Hibbert, D.; Thordarson, P. The Death of the Job Plot, Transparency, Open Science and Online Tools, Uncertainty Estimation Methods and Other Developments in Supramolecular Chemistry Data Analysis. *Chem. Commun.* **2016**, *52*, 12792–12805.

(10) Kudisch, M.; Lim, C.-H.; Thordarson, P.; Miyake, G. M. Energy Transfer to Ni-Amine Complexes in Dual Catalytic, Light-Driven C–N Cross-Coupling Reactions. *J. Am. Chem. Soc.* **2019**, *141*, 19479–19486.

(11) Hu, W.; Xie, J.; Chau, H. W.; Si, B. C. Evaluation of Parameter Uncertainties in Nonlinear Regression Using Microsoft Excel Spreadsheet. *Environ. Syst. Res.* **2015**, *4*.

(12) Bannwarth, C.; Ehlert, S.; Grimme, S., GFN2-xTB—An Accurate and Broadly Parametrized Self-Consistent Tight-Binding Quantum Chemical Method with Multipole Electrostatics and Density-Dependent Dispersion Contributions. *J. Chem. Theory Comput.* **2019**, *15*, 1652-1671.

(13) Bannwarth, C., Caldeweyher, E., Ehlert, S., Hansen, A., Pracht, P., Seibert J., Spicher, S., Grimme, S., Extended tight-binding quantum chemistry methods. *WIREs Comput. Mol. Sci.* **2020**, *11*, 1-49.

(14) Ziegler, T.; Rauk, A., On the calculation of bonding energies by the Hartree Fock Slater method. *Theor. Chim. Acta* **1977**, *46*, 1-10.

(15) Ziegler, T.; Rauk, A. A theoretical study of the ethylene-metal bond in complexes between copper(1+), silver(1+), gold(1+), platinum(0) or platinum(2+) and ethylene, based on the Hartree-Fock-Slater transition-state method. *Inorg. Chem.* **1979**, *18*, 1558–1565.

(16) Becke, A. D., Density-functional exchange-energy approximation with correct asymptotic behavior. *Phys. Rev. A* **1988**, *38*, 3098-3100.

(17) Lee, C.; Yang, W.; Parr, R. G., Development of the Colle-Salvetti correlation-energy formula into a functional of the electron density. *Phys. Rev. B* **1988**, *37*, 785-789.

(18) Grimme, S.; Antony, J.; Ehrlich, S.; Krieg, H., A consistent and accurate ab initio parametrization of density functional dispersion correction (DFT-D) for the 94 elements H-Pu. *J. Chem. Phys.* **2010**, *132*, 154104.

(19) Grimme, S.; Ehrlich, S.; Goerigk, L., Effect of the damping function in dispersion corrected density functional theory. *J. Comput. Chem.* **2011**, *32*, 1456-1465.

(20) ADF 2018, S., Theoretical Chemistry, Vrije Universiteit, Amsterdam, The Netherlands, http://www.scm.com

(21) G. A. Zhurko, Chemcraft 1.80 (build 523b) - graphical program for visualization of quantum chemistry computations. (https://chemcraftprog.com).

(22) Johnson, E., Keinan, S., Mori-Sánchez, P., Contreras-García, J., Cohen, A., Yang, W. Revealing Noncovalent Interactions. *J. Am. Chem. Soc.* **2010**, *132*, 6498-6506.

(23) J. Contreras-García, J., Johnson, E., Keinan, S., Chaudret, R., Piquemal, J., Beratan, D., Yang, NCIPLOT: A Program for Plotting Noncovalent Interaction Regions. *J. Chem. Theory Comput.* **2011**, *7*, 625–632.

(24) Contreras-García, J., Yang, W., Johnson, E. Analysis of Hydrogen-Bond Interaction Potentials from the Electron Density: Integration of Noncovalent Interaction Regions. *J. Phys. Chem. A*, **2011**, *115*, 12983–12990.

(25) Lu, T., Chen, F. Multiwfn: A multifunctional wavefunction analyzer. *J. Comput. Chem.* **2012**, *33*, 580-592.
